# Cycloartane and Oleanane Glycosides from the Tubers of *Eranthis cilicica*

**DOI:** 10.3390/molecules24010069

**Published:** 2018-12-25

**Authors:** Kazuki Watanabe, Yoshihiro Mimaki, Haruhiko Fukaya, Yukiko Matsuo

**Affiliations:** 1 Faculty of Pharmacy, Takasaki University of Health and Welfare, 37-1, Nakaorui, Takasaki, Gunma 370-0033, Japan; watanabe-k@takasaki-u.ac.jp; 2 Department of Medicinal Pharmacognosy, School of Pharmacy, Tokyo University of Phramacy and Life Sciences, 1432-1, Horinouchi, Hachioji, Tokyo 192-0392, Japan; mimakiy@toyaku.ac.jp (Y.M.); fukayah@toyaku.ac.jp (H.F.)

**Keywords:** *Eranthis cilicica*, *Ranunculaceae*, cycloartane glycoside, oleanane glycoside, spectroscopic analysis, X-ray crystallographic analysis, apoptosis

## Abstract

Phytochemical analysis of the tubers of *Eranthis cilicica* was performed as part of our continuous study on the plants of the family *Ranunculaceae*, which resulted in the isolation of eleven new cycloartane glycosides (**1**–**11**) and one new oleanane glycoside (**13**), together with one known oleanane glycoside (**12**). The structures of the new compounds were determined by extensive spectroscopic analysis, including two-dimensional (2D) NMR, and enzymatic hydrolysis followed by either X-ray crystallographic or chromatographic analysis. The aglycone (**1a**) of **2** and its C-23 epimer (**8a**), and the oleanane glycosides (**12** and **13**) showed cytotoxic activity against HL-60 leukemia cells with IC_50_ values ranging from 10.6 μM to 101.6 μM. HL-60 cells were much more sensitive to **8a** (IC_50_ 14.8 μM) than **1a** (IC_50_ 101.1 μM), indicating that the C-23 configuration is associated with the cytotoxicity of these cycloartane derivatives. Compound **12** was revealed so as to partially induce apoptotic cell death in HL-60 cells, as was evident from morphology of HL-60 cells treated with **12**.

## 1. Introduction

We carried out systematic phytochemical screenings of the plants belonging to the family *Ranunculaceae*, such as the *Adonis* [1,2,3,4], *Anemone* [5,6], *Cimicifuga* [7,8], *Clematis* [9], *Helleborus* [10,11,12,13,14], and *Pulsatilla* species [15,16], and isolated various triterpene and steroidal glycosides, including cardiac and pregnane glycosides. Among them, cycloartane glycosides had a unique structure and showed relationships between chemical structure and cytotoxic activity [8]. The genus *Eranthis* also belongs to the family *Ranunculaceae* and is taxonomically related to the genus *Helleborus* [17]. Previously, we have isolated two new oleanane bisdesmosides, eranthisaponins A and B [18], and eight new chromone derivatives from the tubers of *Eranthis cilicica* [19]. Further phytochemical examination of the *E. cilicica* tubers resulted in the isolation of eleven new cycloartane glycosides and one new oleanane glycoside, together with one known oleanane glycoside. We report herein the structural determination of the new compounds by extensive spectroscopic analysis, including two-dimensional (2D) NMR, and enzymatic hydrolysis, followed by either X-ray crystallographic or chromatographic analysis. As part of our ongoing phytochemical study of *Ranunculaceae* plants, the cytotoxic activity of the cycloartane-type glycosides **2** and **8**, the aglycone **1a** and its C-23 epimer **8a**, and the oleanane-type triterpene glycosides **12** and **13** against HL-60 human promyelocytic leukemia cells is evaluated and briefly discussed. 

## 2. Results and Discussion

### 2.1. Isolation and Structure Elucidation of 1–13 

**Figure 1:**
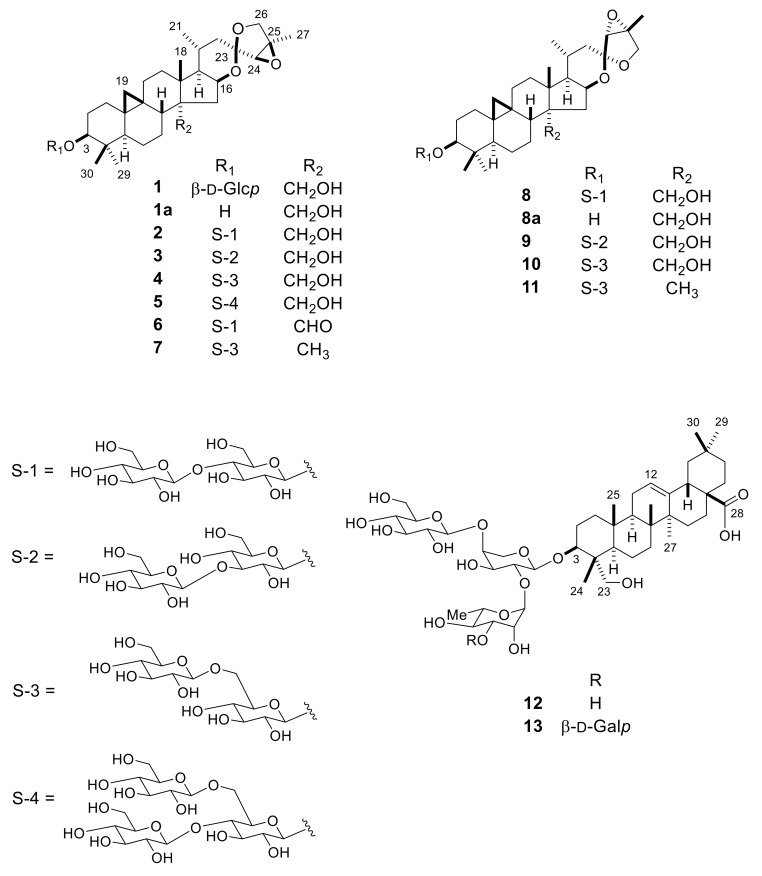
Structures of **1**, **1a**, **2**–**8**, **8a**, and **9**–**13.**

The MeOH extract of the *E. cilicica* tubers was allowed to pass through the porous-polymer polystyrene resin (Diaion HP-20) column, and a series of chromatographic separations of the glycoside-enriched fraction using column chromatography (CC) on silica gel and octadecylsilanized (ODS) silica gel were performed to obtain compounds **1**–**13** (Figure 1). The known compound **12** was identified as 3β-[(*O*-β-d-glucopyranosyl-(1→4)-*O*-[α-l-rhamnopyranosyl-(1→2)]-α-l-arabinopyranosyl)oxy]-23-hydroxyolean-12-en-28-oic acid by direct comparison with an authentic sample isolated from *Anemone coronaria* [6]. 

**Figure 2:**
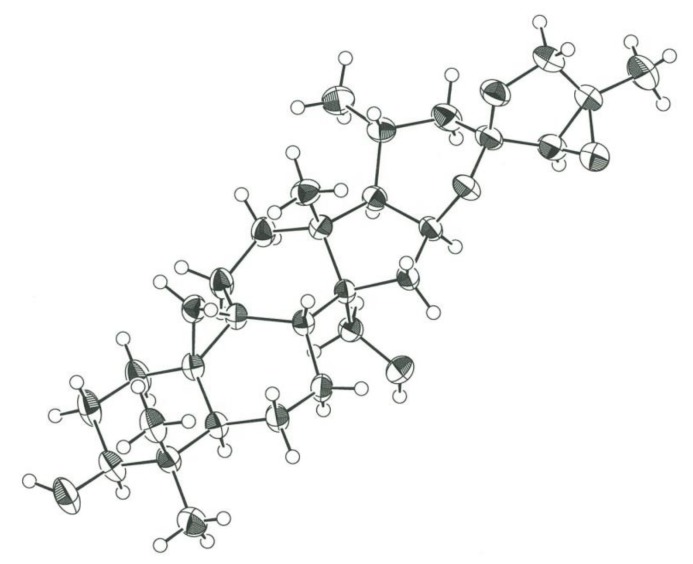
Perspective drawing of **1a.**

Compound **1** was obtained as an amorphous solid, and its molecular formula was determined to be C_36_H_56_O_10_, based on high-resolution (HR)-electrospray ionization (ESI)-time-of-flight (TOF)-MS (*m*/*z* 671.3794 [M + Na]^+^) data and ^13^C-NMR spectrum with 36 carbon signals (Table 1). The IR spectrum of **1** showed an absorption band attributed to hydroxy groups at 3388 cm^−1^. The ^1^H-NMR spectrum of **1** was typical of a triterpene glycoside based on a cycloartane derivative, showing signals for a cyclopropane methylene group at δ_H_ 0.48 and 0.20 (each d, *J* = 4.0 Hz); four tertiary methyl groups at δ_H_ 1.42, 1.36, 1.29, and 1.03 (each s); a secondary methyl group at δ_H_ 0.97 (d, *J* = 6.5 Hz); and an anomeric proton δ_H_ 4.94 (d, *J* = 7.8 Hz). Enzymatic hydrolysis of **1** using naringinase gave a new triterpene aglycone (**1a**: C_30_H_46_O_5_) as colorless needles and d-glucose as a carbohydrate moiety. Based on X-ray crystallographic analysis, the structure of **1a** was unambiguously established as (23*R*,24*R*,25*R*)-16β,23:23,26:24,25-triepoxy-9,19-cycloartane-3β,28-diol (Figure 2). Linkage of a β-d-glucopyranosyl group to the C-3 hydroxy group of the aglycone in **1** was ascertained by long-range correlations between the anomeric proton (H-1′) at δ_H_ 4.94 and the C-3 carbon of the aglycone at δ_C_ 88.6 in the ^1^H-detected heteronuclear multiple-bond connectivity (HMBC) spectrum of **1**. Thus, **1** was established as (23*R*,24*R*,25*R*)-16β,23:23,26:24,25-triepoxy-28-hydroxy-9,19-cycloartan-3β-yl β-d-glucopyranoside.

Compound **2** had the molecular formula C_42_H_66_O_15_, as determined by HR-ESI-TOF-MS (*m*/*z* 833.4297 [M + Na]^+^) and ^13^C-NMR (42 carbon signals) data. The molecular formula was larger than that of **1** by C_6_H_10_O_5_, which corresponded to one hexosyl unit. The ^1^H-NMR spectrum of **2** showed signals due to two anomeric protons at δ_H_ 5.20 (d, *J* = 7.9 Hz) and 4.87 (d, *J* = 7.8 Hz), as well as a cyclopropane methylene group at δ_H_ 0.47 and 0.20 (each d, *J* = 4.0 Hz), four tertiary methyl groups at δ_H_ 1.42, 1.35, 1.27, and 1.02 (each s), a secondary methyl group at δ_H_ 0.97 (d, *J* = 6.6 Hz). Enzymatic hydrolysis of **2** with naringinase gave **1a** and d-glucose. The ^1^H- and ^13^C-NMR signals of the diglycoside moiety, which were assigned based on ^1^H-^1^H correlation spectroscopy (COSY) and ^1^H-detected heteronuclear multiple coherence (HMQC) spectra, indicated the presence of a terminal β-d-glucopyranosyl unit [δ_H_ 5.20 (d, *J* = 7.9 Hz, H-1′′ of Glc (II)); δ_C_ 104.9 (CH), 74.8 (CH), 78.2 (CH), 71.5 (CH), 78.4 (CH), 62.3 (CH_2_)] and a C-4 glycosylated β-d-glucopyranosyl unit [δ_H_ 4.87 (d, *J* = 7.8 Hz, H-1′ of Glc (I)); δ_C_ 106.4 (CH), 75.2 (CH), 76.9 (CH), 81.6 (CH), 76.2 (CH), 62.3 (CH_2_)] in the molecule of **2 [20]**. In the HMBC spectrum of **2**, long-range correlations were observed between H-1′′ of Glc (II) and C-3′ of Glc (I), and between H-1′ of Glc (I) and C-3 of the aglycone moiety at δ_C_ 88.7. The molecular formula of **2** was assigned as (23*R*,24*R*,25*R*)-16β,23:23,26:24,25-triepoxy-28-hydroxy-9,19-cylcoartan-3β-yl *O*-β-d-glucopyranosyl-(1→4)-β-d-glucopyranoside.

Compounds **3** and **4**, which had the same molecular formula as **2** (C_42_H_66_O_15_), gave **1a** and d-glucose upon enzymatic hydrolysis. The ^1^H- and ^13^C-NMR spectra of **3** and **4** suggested that they were constitutional isomers of **2** based on the linkage position of the terminal d-glucosyl unit (Glc (II)) at the inner d-glucosyl unit (Glc (I)) in the diglucosyl residue. In the HMBC spectrum of **3**, H-1′′ of Glc (II) at δ_H_ 5.28 (d, *J* = 7.9 Hz) showed a long-range correlation with C-3′ of Glc (I) at δ_C_ 88.7, and H-1′ of Glc (I) at δ_H_ 4.86 (d, *J* = 7.8 Hz), in turn, showed a long-range correlation with C-3 of the aglycone at δ_C_ 88.6. On the other hand, HMBC correlations were observed between H-1′′ of Glc (II) at δ_H_ 5.28 (d, *J* = 7.8 Hz) and C-6′ of Glc (I) at δ_C_ 70.2, and between H-1′ of Glc (I) at δ_H_ 4.86 (d, *J* = 7.7 Hz) and C-3 of the aglycone at δ_C_ 88.4 in **4**. Compounds **3** and **4** were established as *O*-β-d-glucopyranosyl-(1→3)-β-d-glucopyranoside and *O*-β-d-glucopyranosyl-(1→6)-β-d-glucopyranoside of (23*R*,24*R*,25*R*)-16β,23:23,26:24,25-triepoxy-28-hydroxy-9,19-cycloartan-3β-yl, respectively.

Compound **5** (C_48_H_76_O_20_) also yielded **1a** and d-glucose upon enzymatic hydrolysis. The ^1^H- and ^13^C-NMR spectra of **5** showed signals for two terminal β-d-glucopyranosyl units [δ_H_ 5.45 (d, *J* = 7.9 Hz, H-1′′ of Glc (II)); δ_C_ 104.7 (CH), 75.1 (CH), 78.2 (CH), 71.6 (CH), 78.1 (CH), 62.3 (CH_2_); and δ_H_ 5.34 (d, *J* = 7.8 Hz, H-1′′′ of Glc (III)); δ_C_ 105.0 (CH), 75.1 (CH), 78.2 (CH), 71.5 (CH), 78.1 (CH), 62.5 (CH_2_)] and a C-4 and C-6 diglycosylated β-d-glucopyranosyl unit [δ_H_ 4.80 (d, *J* = 7.5 Hz, H-1′ of Glc (I)); δ_C_ 106.3 (CH), 75.0 (CH), 76.5 (CH), 81.1 (CH), 74.9 (CH), 68.7 (CH_2_)] [21]. In the HMBC spectrum of **5**, long-range correlations were observed between H-1′′ of Glc (II) and C-4′ of Glc (I), H-1′′′ of Glc (III) and C-6′ of Glc (I), and between H-1′ of Glc (II) and C-3 of the aglycone at δ_C_ 88.7. Thus, **5** was deduced to be (23*R*,24*R*,25*R*)-16β,23:23,26:24,25-triepoxy-28-hydroxy-9,19-cylcoartan-3β-yl *O*-β-d-glucopyranosyl-(1→4)-*O*-[β-d-glucopyranosyl(1→6)]-β-d-glucopyranoside.

Compounds **6** and **7** had the molecular formula C_42_H_64_O_15_ and C_42_H_66_O_14_, respectively, based on HR-ESI-TOF-MS and ^13^C-NMR data. The ^1^H- and ^13^C-NMR spectral properties of **6** were essentially analogous to those of **2**; however, the hydroxymethyl signals at δ_H_ 4.02 and 3.94 (each d, *J* = 11.5 Hz) and δ_C_ 63.4 assignable to H_2_-28 and C-28 in **2** were replaced by those due to an aldehyde group at δ_H_ 10.14 (s) and δ_C_ 210.2 in **6**. Treatment of **6** with NaBH_4_ in EtOH afforded **2**. On the other hand, comparison of the ^1^H- and ^13^C-NMR spectra of **7** with those **4** revealed that the H_2_-28/C-28 hydroxymethyl proton and carbon signals observed for **4** disappeared in the case of **7**. Instead, the signals from a tertiary methyl group were detected at δ_H_ 0.80 (s) and δ_C_ 19.7 in the spectrum of **7**. The methyl proton signal showed HMBC correlations with the C-8 (δ_C_ 47.5), C-13 (δ_C_ 46.3), C-14 (δ_C_ 44.7) and C-15 (δ_C_ 44.4) carbon signals. Compounds **6** and **7** were determined to be (23*R*,24*R*,25*R*)-16β,23:23,26:24,25-triepoxy-28-oxo-9,19-cycloartan-3β-yl *O*-β-d-glucopyranosyl-(1→4)-β-d-glucopyranoside and (23*R*,24*R*,25*R*)-16β,23:23,26:24,25-triepoxy-9,19-cycloartan-3β-yl *O*-β-d-glucopyranosyl-(1→6)-β-d-glucopyranoside.

Compound **8** had the same molecular formula as **2** (C_42_H_66_O_15_), and its ^1^H- and ^13^C-NMR spectral features were closely related to those of **2**, except for the signals from the ring E and F parts. Enzymatic hydrolysis of **8** gave an aglycone (**8a**; C_30_H_46_O_5_) and d-glucose. The phase-sensitive NOE correlation spectroscopy (PHNOESY) spectrum of **8a** showed NOE correlations between H-16 (δ_H_ 4.80) and H-22 α (δ_H_ 1.56)/H-26a (δ_H_ 3.82), H-20 (δ_H_ 1.85) and H-24 (δ_H_ 3.69), and between H-22β(δ_H_ 2.16) and H-24, indicating that **8a** was a new aglycone, the C-23 epimer of **1a**. Compound **8** was assigned as (23*S*,24*R*,25*R*)-16β,23:23,26:24,25-triepoxy-28-hydroxy-9,19-cycloartan-3β-yl *O*-β-d-glucopyranosyl-(1→4)-β-d-glucopyranoside.

Compounds **9** and **10** had the same molecular formula C_42_H_66_O_15_, and their ^1^H- and ^13^C-NMR spectra were suggestive of cycloartane diglucosides closely related to **8**. Indeed, enzymatic hydrolysis of **9** and **10** gave **8a** and d-glucose. Assignments of the ^1^H- and ^13^C-NMR signals from the sugar moieties of **9** and **10**, which were established by ^1^H-^1^H COSY and HMQC spectral analysis, implied that the diglucoside sequences of **9** and **10** corresponded to those of **3** and **4**, respectively. Furthermore, HMBC correlations were observed from H-1′′ of Glc (II) at δ_H_ 5.31 (d, *J* = 7.9 Hz) to C-3′ of Glc (I) at δ_C_ 88.9, and from H-1′ at δ_H_ 4.89 (d, *J* = 7.8 Hz) to C-3 of the aglycone at δ_C_ 88.7 in **9**, and from H-1′′ of Glc (II) at δ_H_ 5.16 (d, *J* = 7.8 Hz) to C-6′ of Glc (I) at δ_C_ 70.3, and from H-1′ at δ_H_ 4.89 (d, *J* = 7.7 Hz) to C-3 of the aglycone at δ_C_ 88.4 in **10**. Compounds **9** and **10** were formulated as *O*-β-d-glucopyranosyl-(1→3)-β-d-glucopyranoside and *O*-β-d-glucopyranosyl-(1→6)-β-d-glucopyranoside of (23*S*,24*R*,25*R*)-16β,23:23,26:24,25-triepoxy-28-hydroxy-9,19-cylcoartan-3β-yl, respectively.

Compound **11** (C_42_H_66_O_14_) bore close similarity to **10** in terms of the ^1^H- and ^13^C-NMR spectral features. However, the hydroxymethyl signals at δ_H_ 3.98 and 3.89 (each d, *J* = 11.5 Hz) and δ_C_ 63.2 assignable to H_2_-28 and C-28 in **10** were replaced by the signals due to a methyl group at δ_H_ 0.79 (s) and δ_C_ 19.6 in **11**. In addition, the methyl proton signal showed HMBC correlations with the C-8 (δ_C_ 47.6), C-13 (δ_C_ 46.3), C-14 (δ_C_ 44.5), and C-15 (δ_C_ 43.8) carbon signals. Compound **11** was established to be (23*S*,24*R*,25*R*)-16β,23:23,26:24,25-triepoxy-9,19-cycloartan-3β-yl *O*-β-d-glucopyranosyl-(1→6)-β-d-glucopyranoside.

Compound **13** had the molecular formula C_53_H_86_O_22_, as revealed by HR-ESI-TOF-MS (*m*/*z* 1075.5710 [M + H]^+^) and ^13^C-NMR (53 carbon signals) data. The molecular formula was larger than that of **12** by C_6_H_10_O_5_, which corresponded to one hexosyl unit. The ^1^H-NMR spectrum of **13** displayed signals for four anomeric protons at δ_H_ 6.02 (br s), 5.29 (d, *J* = 7.8 Hz), 5.06 (d, *J* = 7.9 Hz) and 4.92 (d, *J* = 6.9 Hz), as well as the signals for six tertiary methyl groups at δ_H_ 1.25, 1.05, 1.02, 1.00, 0.94 and 0.92 (each s), and an olefinic proton at δ_H_ 5.46 (t-like, *J* = 3.0 Hz), suggesting that this compound was a tetraglycoside of an oleanoic acid derivative. Acid hydrolysis of **13** with 1M HCl in dioxane-H_2_O (1:1) gave 23-hydroxyolean-12-en-28-oic acid (hederagenin) as the aglycone [22], and l-arabinose, d-galactose, d-glucose and l-rhamnose as the carbohydrate moieties. When the ^13^C-NMR spectrum of **13** was compared with that of **12 [6]**, six signals assignable to a terminal β-d-galactopyraosyl group (Gal) were observed in addition to signals for a 2,4-disubstituted α-l-arabinopyranosyl group (Ara), C-3 substituted α-l-rhamnopyranosyl group (Rha), and terminal β-d-glucopyranosyl group (Glc). The anomeric configurations of Ara, Gal and Glc were confirmed to be α, β, and β, respectively, based on the relatively large ^3^*J*_H-1,H-2_ values (6.9–7.9 Hz). In the case of the Rha moiety, the large ^1^*J*_C-1,H-1_ (172 Hz) was indicative of the α-anomeric configuration [6]. In the HMBC spectrum of **13**, long-range couplings were observed between H-1′′′ of Glc (δ_H_ 5.29) and C-4′ of Ara at δ_C_ 80.0, H-1′′′′ of Gal (δ_H_ 5.06) and C-3′′ of Rha at δ_C_ 83.2, H-1′′ of Rha (δ_H_ 6.02) and C-2′ of Ara at δ_C_ 76.5, and between H-1′ of Ara (δ_H_ 4.92) and C-3 of the aglycone at δ_C_ 81.3. Compound **13** was determined to be 3 β-[(*O*-β-d-galactopyranosyl-(1→3)-*O*-α-l-rhamnopyranosyl-(1→2)-*O*-[β-d-glucopyranosyl-(1→4)]-α-l-arabinopyranosyl)oxy]-23-hydroxyolean-12-en-28-oic acid.

### 2.2. Cytotoxic Activity of ***1a***, ***2***, ***8***, ***8a***, ***12***, and ***13***

**Figure 3:**
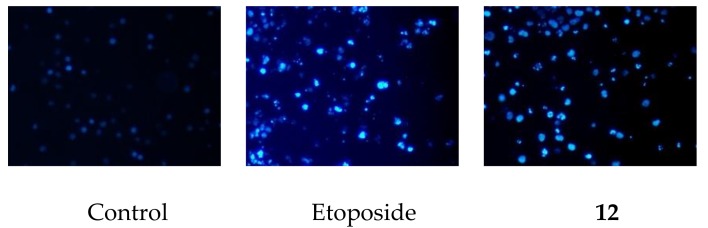
Morphology of HL-60 cells treated with either etoposide or **12**. HL-60 cells were incubated with either etoposide (17 μM) or **12** (20 μM) for 72 h, stained with DAPI and observed under a fluorescence microscope (magnification, 200×).

We previously reported that the slight differences in the structure of cycloartane glycosides effected on cytotoxic activity [8]. In this study, some selected compounds, the new cycloartane glycosides (**2** and **8**), and the aglycone (**1a**) of **2** and its C-23 epimer (**8a**), as well as oleanane glycosides (**12** and **13**) were evaluated for their cytotoxic activity against HL-60 cells using a modified 3-(4,5-dimethylthiazol-2-yl)-2,5-diphenyl-2*H*-tetrazolium bromide (MTT) assay (Table 2). Etoposide was used as a positive control, and it gave an IC_50_ value of 0.32 ± 0.01 μM. The cytotoxic activity of **8a** against HL-60 cells (IC_50_ 14.8 ± 1.00 μM) was much more potent than that of **1a** (IC_50_ 101.1 ± 0.44 μM), indicating that the C-23 configuration is associated with the cytotoxicity of these cycloartane derivatives. On the other hand, **2** and **8** were not cytotoxic to HL-60 cells at sample concentrations up to 200 μM. Compounds **12** and **13** were cytotoxic to HL-60 cells, with IC_50_ values of 10.6 ± 0.40 μM and 10.8 ± 0.53 μM, respectively. After HL-60 cells were exposed to **12** at a sample concentration of 20 μM for 72 h, they were stained with 4′,6-diamidino-2-phenylindole dihydrochloride (DAPI) and observed under a fluorescence microscope. The cells showed nuclear chromatin condensation and nuclear disassembly, illustrated in Figure 3. Therefore, **12** partially induced apoptotic cell death in HL-60 cells. 

## 3. Materials and Methods

### 3.1. General Experimental Procedures and Plant Material

The instruments, experimental conditions, and plant material used (except for those mentioned below) were the same as those described in previous papers [18,19]. Melting point was determined on an MP-3 melting point apparatus (Yanaco, Kyoto, Japan). X-ray diffraction experiments were carried out on a DIP image plate diffractometer (Bruker AXS, Karlsruhe, Germany).

### 3.2. Extraction and Isolation

The MeOH extract (135 g) of *Eranthis cilicica* tubers (1.3 kg) was subjected to Diaion HP-20 CC [19]. The 80% MeOH eluted portion (30 g) was chromatographed on silica gel eluted with gradient mixtures of CHCl_3_-MeOH (20:1; 9:1, 4:1, 2:1) and finally with MeOH to give 9 subfractions (Frs. A–I). Fr. E was subjected silica gel CC eluted with CHCl_3_-MeOH (9:1) and ODS silica gel CC eluted with MeCN-H_2_O (1:2) to yield **1** (17.0 mg) and **7** (13.4 mg). Fr. G was repeatedly subjected to silica gel CC eluted with CHCl_3_-MeOH-H_2_O (60:10:1, 50:10:1, 40:10:1) and ODS silica gel CC eluted with MeCN-H_2_O (5:8, 5:9, 1:2) to yield **2** (121 mg), **3** (7.8 mg), **8** (100 mg), **9** (4.1 mg), **10** (35.0 mg), and **11** (5.1 mg). Fr. H was subjected to CC on silica gel eluted with CHCl_3_-MeOH-H_2_O (30:10:1) and ODS silica gel eluted with MeCN-H_2_O (1:2) and MeOH-H_2_O (7:5) to yield **13** (15.2 mg). Fr. I was subjected to CC on silica gel eluted with CHCl_3_-MeOH-H_2_O (14:8:1; 10:10:1) and ODS silica gel eluted with MeCN-H_2_O (5:8, 2:5, 4:11, 1:3) to yield **12** (13.7 mg). The 50% MeOH eluted portion (35 g) was chromatographed on silica gel eluted with CHCl_3_-MeOH-H_2_O (40:10:1) to give 6 subfractions (Frs. a–f). Fr. b was subjected to silica gel column eluted with CHCl_3_-MeOH (20:1) and CHCl_3_-MeOH-H_2_O (40:10:1) and ODS silica gel column eluted with MeCN-H_2_O (1:2, 2:5, 1:3) to yield **4** (15.2 mg) and **6** (10.0 mg). Fr. c was subjected to silica gel CC eluted with CHCl_3_-MeOH (10:1) and CHCl_3_-MeOH-H_2_O (40:10:1) and ODS silica gel CC eluted with MeCN-H_2_O (1:2) to yield **5** (33.8 mg).

### 3.3. Structural Characterization

Compound **1**: Amorphous solid. [α]_D_^25^ −46.0 (*c* 0.10, MeOH). HR-ESI-TOF-MS *m*/*z*: 671.3794 [M + Na]^+^ (Calcd for C_36_H_56_NaO_10_, 671.3771). IR ν_max_ (film) cm^−1^: 3388 (OH), 2928 and 2870 (CH). ^1^H-NMR spectral data for **1** are provided in the Supplementary Materials. ^13^C-NMR, see Table 1.

Enzymatic Hydrolysis of **1** Compound **1** (4.5 mg) was treated with naringinase (EC 232-96-4, Sigma; 22.5 mg) in a mixture of HOAc/KOAc buffer (pH 4.3, 5 mL) and EtOH (2 mL) at room temperature for 72 h. The reaction mixture was purified by silica gel CC eluted with CHCl_3_-MeOH (22:1) followed by MeOH to give **1a** (2.4 mg) and a sugar fraction (0.6 mg). The sugar fraction was analyzed by HPLC under the following conditions: Capcell Pak NH_2_ UG80 column (4.6 mm i.d. × 250 mm, 5 μm, Shiseido, Tokyo, Japan); mobile phase of MeCN-H_2_O (7:3); detection by refractive index and optical rotation, and at a flow rate of 1.0 mL/min. d-glucose was identified by comparing its retention time and optical rotation with those of an authentic sample; *t*_R_ 11.62 min (d-glucose, positive optical rotation).

Compound **1a**: Colorless needles from MeOH-MeCN (1:1). mp 281–285 °C. [α]_D_^24^ −48.0 (*c* 0.10, MeOH). HR-ESI-TOF-MS *m*/*z*: 509.3255 [M + Na]^+^ (Calcd for C_30_H_46_NaO_5_, 509.3243). IR ν_max_ (film) cm^−1^: 3476 (OH), 2953, 2925 and 2868 (CH). ^1^H-NMR spectral data for **1a** are provided in the Supplementary Materials. ^13^C-NMR, see Table 1.

X-Ray Crystallography of **1a** Monoclinic, space group *C*2, unit cell dimension *a* = 32.945(2) Å, *b* = 6.9250(2) Å, *c* = 13.5690(6), β = 111.270(2)°, *V* = 2884.8(2) Å^3^, *Z* = 4; *T* = 296 K, *d*_calc_ = 1.204 Mg/m^3^; μ (Mo Kα, λ = 0.71073 Å) = 0.084 mm^−1^; *R* [*I* > 2σ(*I*)] = 0.0370, *wR* [*I* > 2σ(*I*)] = 0.1054, *R* [for all data] = 0.0409, *wR* [for all data] = 0.1081. Crystallographic data of **1a** have been deposited with the Cambridge Crystallographic Data Centre (CCDC) as supplementary publication no. CCDC 1877743. Copies of the data can be obtained free of charge from the CCDC via http://beta-www.ccdc.cam.ac.uk.

Compound **2**: Amorphous solid. [α]_D_^26^ −34.0 (*c* 0.10, MeOH). HR-ESI-TOF-MS *m*/*z*: 833.4297 [M + Na]^+^ (Calcd for C_42_H_66_NaO_15_, 833.4299). IR ν_max_ (film) cm^−1^: 3397 (OH), 2930 and 2870 (CH). ^1^H-NMR spectral data for **2** are provided in the Supplementary Materials. ^13^C-NMR, see Table 1.

Compound **3**: Amorphous solid. [α]_D_^24^ −48.0 (*c* 0.10, MeOH). HR-ESI-TOF-MS *m*/*z*: 833.4359 [M + Na]^+^ (Calcd for C_42_H_66_NaO_15_, 833.4299). IR ν_max_ (film) cm^−1^: 3450 (OH), 2932 and 2872 (CH). ^1^H-NMR spectral data for **3** are provided in the Supplementary Materials. ^13^C-NMR, see Table 1.

Compound **4**: Amorphous solid. [α]_D_^26^ −44.0 (*c* 0.10, MeOH). HR-ESI-TOF-MS *m*/*z*: 833.4340 [M + Na]^+^ (Calcd for C_42_H_66_NaO_15_, 833.4299). IR ν_max_ (film) cm^−1^: 3408 (OH), 2933 and 2871 (CH). ^1^H-NMR spectral data for **4** are provided in the Supplementary Materials. ^13^C-NMR, see Table 1.

Compound **5**: Amorphous solid. [α]_D_^28^ −16.0 (*c* 0.10, MeOH). HR-ESI-TOF-MS *m*/*z*: 973.5001 [M + H]^+^ (Calcd for C_48_H_77_O_20_, 973.5008). IR ν_max_ (film) cm^−1^: 3363 (OH), 2932 and 2872 (CH). ^1^H-NMR spectral data for **5** are provided in the Supplementary Materials. ^13^C-NMR, see Table 1.

Enzymatic Hydrolysis of **2**–**5** Compounds **2** (54.1 mg), **3** (2.5 mg), **4** (5.1 mg), and **5** (4.8 mg) were independently subjected to enzymatic hydrolysis with naringinase as described for **1** to give **1a** (25.2 mg from **2**; 1.3 mg from **3**; 2.5 mg from **4**; 2.6 mg from **5**) and a sugar fraction (5.9 mg from **2**; 0.4 mg from **3**; 0.8 mg from **4**; 1.2 mg from **5**). HPLC analysis of the sugar fraction under the same conditions as in the case of that of **1** showed the presence of d-glucose.

Compound **6**: Amorphous solid. [α]_D_^24^ −84.0 (*c* 0.10, MeOH). HR-ESI-TOF-MS *m*/*z*: 831.4100 [M + Na]^+^ (Calcd for C_42_H_64_NaO_15_, 831.4143). IR ν_max_ (film) cm^−1^: 3387 (OH), 2925 and 2870 (CH), 1712 (C=O). ^1^H-NMR spectral data for **6** are provided in the Supplementary Materials. ^13^C-NMR, see Table 1.

Preparation of **2** from **6** Compound **6** (1.7 mg) was dissolved in NaBH_4_ (2.2 mg) ethanolic solution (1 mL) and it was stirred at room temperature for 5 h. After Me_2_CO was added to the reaction mixture, the solvent was removed under reduced pressure. The residue was suspended with H_2_O (3 mL) and extracted with EtOAc (3 mL × 3). The EtOAc extract was chromatographed on silica gel eluted with CHCl_3_-MeOH-H_2_O (30:10:1) to yield **2** (1.2 mg).

Compound **7**: Amorphous solid. [α]_D_^25^ −70.0 (*c* 0.10, MeOH). HR-ESI-TOF-MS *m*/*z*: 817.4393 [M + Na]^+^ (Calcd for C_42_H_66_NaO_14_, 817.4350). IR ν_max_ (film) cm^−1^: 3418 (OH), 2931 and 2868 (CH). ^1^H-NMR spectral data for **7** are provided in the Supplementary Materials. ^13^C-NMR, see Table 1.

Compound **8**: Amorphous solid. [α]_D_^26^ −50.0 (*c* 0.10, C_5_H_5_N). HR-ESI-TOF-MS *m*/*z*: 833.4359 [M + Na]^+^ (Calcd for C_42_H_66_NaO_15_, 833.4299). IR ν_max_ (film) cm^−1^: 3348 (OH), 2926 and 2867 (CH). ^1^H-NMR spectral data for **8** are provided in the Supplementary Materials. ^13^C-NMR, see Table 1.

Compound **8a**: Amorphous solid. [α]_D_^24^ −58.0 (*c* 0.10, MeOH). HR-ESI-TOF-MS *m*/*z*: 487.3423 [M + H]^+^ (Calcd for C_30_H_47_O_5_, 487.3424). IR ν_max_ (film) cm^−1^: 3442 (OH), 2955, 2927 and 2870 (CH). ^1^H-NMR spectral data for **8a** are provided in the Supplementary Materials. ^13^C-NMR, see Table 1.

Compound **9**: Amorphous solid. [α]_D_^26^ −36.0 (*c* 0.10, C_5_H_5_N). HR-ESI-TOF-MS *m*/*z*: 833.4276 [M + Na]^+^ (Calcd for C_42_H_66_NaO_15_, 833.4299). IR ν_max_ (film) cm^−1^: 3347 (OH), 2919 and 2871 (CH). ^1^H-NMR spectral data for **9** are provided in the Supplementary Materials. ^13^C-NMR, see Table 1.

Compound **10**: Amorphous solid. [α]_D_^26^ −72.0 (*c* 0.10, MeOH). HR-ESI-TOF-MS *m*/*z*: 833.4313 [M + Na]^+^ (Calcd for C_42_H_66_NaO_15_, 833.4299). IR ν_max_ (film) cm^−1^: 3388 (OH), 2933 and 2872 (CH). ^1^H-NMR spectral data for **10** are provided in the Supplementary Materials. ^13^C-NMR, see Table 1.

Enzymatic Hydrolysis of **8**–**10** Compounds **8** (31.3 mg), **9** (1.9 mg), and **10** (5.3 mg) were independently subjected to enzymatic hydrolysis with naringinase as described for **1** to give **8a** (19.4 mg from **8**; 0.9 mg from **9**; 2.8 mg from **10**) and a sugar fraction (3.2 mg from **8**; 0.3 mg from **9**; 1.1 mg from **10**). HPLC analysis of the sugar fraction under the same conditions as in the case of that of **1** showed the presence of d-glucose.

Compound **11**: Amorphous solid. [α]_D_^26^ −62.0 (*c* 0.10, MeOH). HR-ESI-TOF-MS *m*/*z*: 795.4583 [M + H]^+^ (Calcd for C_42_H_67_O_14_, 795.4531). IR ν_max_ (film) cm^−1^: 3387 (OH), 2927 and 2871 (CH). ^1^H-NMR spectral data for **11** are provided in the Supplementary Materials. ^13^C-NMR, see Table 1.

Compound **13**: Amorphous solid. [α]_D_^27^ +6.0 (*c* 0.10, MeOH). HR-ESI-TOF-MS *m*/*z*: 1075.5710 [M + H]^+^ (Calcd for C_53_H_87_O_22_, 1075.5689). IR ν_max_ (film) cm^−1^: 3376 (OH), 2926 and 2858 (CH), 1696 (C=O). ^1^H-NMR (C_5_D_5_N) δ_H_ 6.02 (1H, br s, H-1′′), 5.46 (1H, t-like, *J* = 3.0 Hz, H-12), 5.29 (1H, d, *J* = 7.8 Hz, H-1′′′), 5.06 (1H, d, *J* = 7.9 Hz, H-1′′′′), 4.92 (1H, d, *J* = 6.9 Hz, H-1′), 4.48 (1H, dd, *J* = 9.3, 7.8 Hz, H-2′′′), 4.46 (1H, br s, H-4′′′), 4.36 (1H, m, H-6a′′′), 4.34 (1H, m, H-6b′′′), 4.24 (1H, d, *J* = 11.0 Hz, H-23a), 4.18 (1H, dd, *J* = 10.5, 4.6 Hz, H-3), 4.10 (1H, dd, *J* = 9.3, 3.3 Hz, H-3′′′), 4.07 (1H, m, H-5′′′), 3.86 (1H, d, *J* = 11.0 Hz, H-23b), 3.26 (1H, dd, *J* = 13.8, 3.9 Hz, H-18), 1.54 (3H, d, *J* = 6.1 Hz, Me-6′′), 1.25 (3H, s, Me-27), 1.05 (3H, s, Me-24), 1.02 (3H, s, Me-26), 1.00 (3H, s, Me-30), 0.94 (3H, s, Me-25), 0.92 (3H, s, Me-29). ^13^C-NMR (C_5_D_5_N) δ_C_: 39.0, 26.3, 81.3, 43.5, 47.6, 18.2, 32.9, 39.8, 48.2, 36.9, 23.8, 122.6, 144.8, 42.2, 28.3, 23.7, 46.7, 42.0, 46.5, 30.9, 34.2, 32.0, 64.0, 13.9, 16.0, 17.5, 26.1, 180.1, 33.2, 23.8 (aglycone C-1–C-30), 104.7, 76.5, 74.2, 80.0, 65.5 (Ara C-1–C-5), 101.6, 71.2, 83.2, 72.6, 69.9, 18.5 (Rha C-1–C-6), 107.1, 73.3, 75.2, 70.0, 76.8, 62.1 (Gal C-1–C-6), 106.5, 75.5, 78.3, 71.4, 78.5, 62.5 (Glc C-1–C-6).

Acid Hydrolysis of **13** A solution of **13** (5.0 mg) in 1 M HCl (dioxane-H_2_O, 1:1, 3 mL) was heated at 95 °C for 1 h under an Ar atmosphere. After the reaction mixture was diluted with H_2_O (2 mL), it was extracted with Et_2_O (5 mL × 2). The Et_2_O extract was chromatographed on silica gel eluted with CHCl_3_-MeOH (19:1) to give hederagenin (2.6 mg). The H_2_O residue was neutralized using an Amberlite IRA-93ZU (Organo, Tokyo, Japan) column and passed through a Sep-Pak C_18_ cartridge (Waters, Milford, MA) eluted with H_2_O-MeOH (3:2) to give a sugar fraction (1.8 mg). HPLC analysis of the sugar fraction under the same conditions as in the case of that of **1** (flow rate, 0.9 mL/min) showed the presence of l-arabinose, d-galactose, d-glucose, and l-rhamnose. *t*_R_ (min): 7.08 (negative optical rotation, l-rhamnose), 8.14 (positive optical rotation, l-arabinose), 12.86 (positive optical rotation, d-galactose), and 13.17 (positive optical rotation, d-glucose).

### 3.4. Cytotoxic Activity

HL-60 cells were maintained in an RPMI-1640 medium. The cell media contained heat-inactivated 10% (*v/v*) FBS supplemented with l-glutamine, penicillin G sodium salt (100 units/mL), and streptomycin sulfate (100 μg/mL). HL-60 (4 × 10^4^ cells/mL) cells were continuously treated with each compound for 72 h, and cell growth was measured using an MTT reduction assay as previously described [23]. Data represented as mean ± S.E.M. of three experiments performed in triplicate. The concentration, resulting in a 50% inhibition value (IC_50_), was calculated from the dose response curve.

### 3.5. DAPI Staininig

The cells (1 × 10^5^ cells/mL) were plated on coverslips in 96-well plates. After 24 h, HL-60 cells were treated with either 20 μM of **12** or 17 μM of etoposide for 72 h. The cells were fixed with 1% glutaraldehyde for 30 min at room temperature before staining with DAPI (0.5 μg/mL in H_2_O). They were observed under a CKX41 fluoroscence microscope (Olympus, Tokyo, Japan).

## 4. Conclusions

Further phytochemical examination of *E. cilicica* tubers gave eleven new cycloartane glycosides (**1**–**11**) and one new oleanane glycoside (**13**), together with one known oleanane glycosides (**12**). The structures of the new compounds were established by extensive spectroscopic analysis, including 2D NMR, and enzymatic hydrolysis, followed by either X-ray crystallographic or chromatographic analysis. The new cycloartane glycosides **2** and **8**, the aglycone **1a** and its C-23 epimer **8a**, and oleanane-type triterpene glycosides **12** and **13** were evaluated for their cytotoxic activity against HL-60 leukemia cells. The cytotoxic activity of **8a** was much more potent than that of **1a**, indicating that the C-23 configuration is associated with the cytotoxicity of these cycloartane derivatives. This is the first report of structure–activity relationships of cycloartane-type triterpenoids on C-23 epimer. Compounds **12** and **13** were moderately cytotoxic to HL-60 cells, and **12** partially induced apoptotic cell death in HL-60 cells.

## Figures and Tables

**Table 1:** ^13^C-NMR spectral data for **1**, **1a**, **2**–**8**, **8a**, and **9**–**11** in C_5_D_5_N.

C	1	1a	2	3	4	5	6	7	8	8a	9	10	11
1	32.1	32.5	32.1	32.0	32.1	32.0	31.3	32.1	32.1	32.4	32.1	32.1	32.0
2	29.8	31.2	29.7	29.6	29.9	29.7	29.5	30.2	29.8	31.1	29.8	30.0	29.9
3	88.6	77.9	88.7	88.6	88.4	88.7	88.3	88.5	88.7	77.8	88.7	88.4	88.5
4	41.2	41.0	41.1	41.0	41.1	41.1	41.0	41.2	41.2	41.0	41.2	41.2	41.1
5	47.5	47.5	47.5	47.4	47.3	47.4	46.4	47.3	47.5	47.4	47.5	47.4	47.2
6	21.1	21.5	21.1	21.0	21.1	21.0	20.0	20.8	21.2	21.5	21.2	21.2	20.8
7	27.4	27.6	27.4	27.3	27.4	27.4	26.7	26.2	27.5	27.6	27.5	27.5	26.2
8	48.3	48.5	48.3	48.3	48.4	48.3	41.9	47.5	48.5	48.7	48.5	48.5	47.6
9	19.7	19.8	19.8	19.7	19.7	19.7	19.4	19.6	19.8	19.8	19.8	19.8	19.5
10	26.6	27.0	26.6	26.5	26.5	26.5	27.6	26.5	26.7	26.9	26.6	26.7	26.5
11	27.3	27.4	27.2	27.2	27.1	27.1	27.6	26.3	27.2	27.2	27.2	27.1	26.1
12	32.6	32.7	32.6	32.5	32.5	32.5	33.6	33.2	32.6	32.6	32.6	32.6	33.1
13	45.5	45.5	45.4	45.4	45.4	45.4	46.5	46.3	45.3	45.3	45.3	45.3	46.3
14	51.5	51.6	51.5	51.5	51.5	51.5	64.0	44.7	51.6	51.6	51.6	51.6	44.5
15	38.2	38.3	38.2	38.1	38.1	38.1	36.1	44.4	37.6	37.6	37.6	37.6	43.8
16	75.3	75.4	75.3	75.2	75.3	75.3	74.2	75.2	73.7	73.7	73.7	73.7	73.1
17	56.9	57.0	56.9	56.7	56.8	56.8	57.1	56.6	57.1	57.1	57.1	57.1	56.7
18	22.0	22.1	22.0	21.9	22.0	22.0	20.5	20.6	22.0	22.0	22.0	22.0	20.5
19	30.6	30.9	30.6	30.5	30.6	30.5	28.1	30.0	30.6	30.8	30.6	30.7	30.1
20	23.9	23.9	23.9	23.8	23.8	23.8	23.1	23.7	26.5	26.4	26.5	26.4	26.2
21	20.7	20.7	20.7	20.6	20.6	20.6	20.9	20.7	20.5	20.4	20.5	20.4	20.3
22	37.7	37.8	37.7	37.6	37.6	37.6	36.8	37.6	36.8	36.7	36.8	36.7	36.5
23	106.1	106.1	106.1	106.0	106.1	106.1	106.2	106.1	106.1	106.1	106.1	106.1	106.0
24	62.0	62.1	62.0	62.0	62.0	62.0	62.1	62.0	63.4	63.4	63.4	63.4	63.3
25	62.4	62.4	62.4	62.4	62.4	62.4	62.4	62.5	63.1	63.1	63.1	63.1	63.1
26	67.9	68.0	67.9	67.9	67.9	67.9	68.1	68.0	68.7	68.6	68.7	68.6	68.6
27	14.2	14.2	14.2	14.1	14.2	14.1	14.2	14.2	13.7	13.7	13.7	13.7	13.7
28	63.5	63.6	63.4	63.3	63.4	63.3	210.2	19.7	63.2	63.2	63.2	63.2	19.6
29	25.7	26.1	25.6	25.5	25.5	25.5	25.6	25.7	25.7	26.0	25.6	25.6	25.6
30	15.4	14.8	15.4	15.3	15.3	15.3	15.1	15.4	15.4	14.8	15.4	15.4	15.3
1′	106.8		106.4	106.1	106.6	106.3	106.4	106.8	106.4		106.2	106.7	106.6
2′	75.8		75.2	74.4	75.5	75.0	75.3	75.6	75.3		74.4	75.6	75.5
3′	78.7		76.9	88.7	78.2	76.5	77.0	78.5	76.9		88.9	78.5	78.5
4′	71.8		81.6	69.7	71.6	81.1	81.6	71.7	81.6		69.8	71.6	71.6
5′	78.3		76.2	77.7	77.0	74.9	76.3	77.1	76.2		77.9	77.1	77.0
6′	63.0		62.3	62.4	70.2	68.7	62.4	70.3	62.4		62.5	70.3	70.1
1′′			104.9	105.7	105.2	104.7	105.0	105.4	105.0		105.9	105.3	105.2
2′′			74.8	75.4	75.1	75.1	74.8	74.7	74.8		75.5	75.2	75.1
3′′			78.2	78.1	78.4	78.2	78.2	78.4	78.2		78.2	78.3	78.2
4′′			71.5	71.4	71.6	71.6	71.5	71.7	71.5		71.6	71.7	71.5
5′′			78.4	78.6	78.3	78.1	78.5	78.4	78.4		78.7	78.4	78.3
6′′			62.3	62.3	62.5	62.3	62.5	62.7	62.4		62.5	62.6	62.5
1′′′						105.0							
2′′′						75.1							
3′′′						78.2							
4′′′						71.5							
5′′′						78.1							
6′′′						62.5							

**Table 2:** Cytotoxic activity of **1a**, **2**, **8**, **8a**, **12**, **13**, and etoposide against HL-60 cells ^a^.

Compound	IC_50_ (µM)
**1a**	101.1 ± 0.44
**2**	>200
**8**	>200
**8a**	14.8 ± 1.00
**12**	10.6 ± 0.40
**13**	10.8 ± 0.53
etoposide	0.32 ± 0.01

^a^ Data represent the mean value ± standard error of the mean (SEM) of three experiments performed in triplicate.

## Supplementary Materials

All NMR spectra of **1**, **1a**, **2**–**8**, **8a**, and **9**–**13** are available online.

## References

[1] Kuroda M., Kubo S., Masatani D., Matsuo Y., Sakagami H., Mimaki Y. (2018). Aestivalosides A–L, twelve pregnane glycosides from the seeds of *Adonis aestivalis*.. Phytochemistry.

[2] Kubo S., Kuroda M., Yokosuka A., Sakagami H., Mimaki Y. (2015). Amurensiosides L–P, five new cardenolide glycosides from the roots of *Adonis amurensis*.. Nat. Prod. Commun..

[3] Kubo S., Kuroda M., Matsuo Y., Masatani D., Sakagami H., Mimaki Y. (2012). New cardenolides from the seeds of *Adonis aestivalis*.. Chem. Pharm. Bull..

[4] Kuroda M., Kubo S., Uchida S., Sakagami H., Mimaki Y. (2010). Amurensiosides A–K, 11 new pregnane glycosides from the roots of *Adonis amurensis*.. Steroids.

[5] Yokosuka A., Sano T., Hashimoto K., Sakagami H., Mimaki Y. (2009). Triterpene glycosides from the whole plant of *Anemone hupehensis* var. japonica and their cytotoxic activity. Chem. Pharm. Bull..

[6] Mimaki Y., Watanabe K., Matsuo Y., Sakagami H. (2009). Triterpene glycosides from the tubers of *Anemone coronaria*.. Chem. Pharm. Bull..

[7] Mimaki Y., Nadaoka I., Yasue M., Ohtake Y., Ikeda M., Watanabe K., Sashida Y. (2006). Neocimicigenosides A and B, cycloartane glycosides from the rhizomes of *Cimicifuga racemosa* and their effects on CRF-stimulated ACTH secretion from AtT-20 cells. J. Nat. Prod..

[8] Watanabe K., Mimaki Y., Sakagami H., Sashida Y. (2002). Cycloartane glycosides from the rhizomes of *Cimicifuga racemosa* and their cytotoxic activities. Chem. Pharm. Bull..

[9] Mimaki Y., Yokosuka A., Hamanaka M., Sakuma C., Yamori T., Sashida Y. (2004). Triterpene saponins from the roots of *Clematis chinensis*.. J. Nat. Prod..

[10] Yokosuka A., Iguchi T., Kawahata R., Mimaki Y. (2018). Cytotoxic bufadienolides from the whole plants of *Helleborus foetidus*.. Phytochem. Lett..

[11] Mimaki Y., Matsuo Y., Watanabe K., Sakagami H. (2010). Furostanol glycosides from the rhizomes of *Helleborus orientalis*.. J. Nat. Med..

[12] Watanabe K., Sakagami H., Mimaki Y. (2005). Four new steroidal saponins from the rhizomes of *Helleborus orientalis*.. Heterocycles.

[13] Mimaki Y., Watanabe K., Sakuma C., Sakagami H., Sashida Y. (2003). Novel polyoxygenated spirostanol glycosides from the rhizomes of *Helleborus orientalis*.. Helv. Chim. Acta.

[14] Watanabe K., Mimaki Y., Sakagami H., Sashida Y. (2003). Bufadienolide and spirostanol glycosides from the rhizomes of *Helleborus orientalis*.. J. Nat. Prod..

[15] Mimaki Y., Yokosuka A., Kuroda M., Hamanaka M., Sakuma C., Sashida Y. (2001). New bisdesmosidic triterpene saponins from the roots of *Pulsatilla chinensis*.. J. Nat. Prod..

[16] Mimaki Y., Kuroda M., Asano T., Sashida Y. (1999). Triterpene saponins and lignans from the roots of *Pulsatilla chinensis* and their cytotoxic activity against HL-60 cells. J. Nat. Prod..

[17] Tsukamoto Y. (1988). The Grand Dictionary of Horticulture.

[18] Watanabe K., Mimaki Y., Sakuma C., Sashida Y. (2003). Eranthisaponins A and B, two new bisdesmosidic triterpene saponins from the tubers of *Eranthis cilicica*.. J. Nat. Prod..

[19] Kuroda M., Uchida S., Watanabe K., Mimaki Y. (2009). Chromones from the tubers of *Eranthis cilicica* and their antioxidant activity. Phytochemistry.

[20] Malinow M.R., Gardner J.O., Nelson J.T., McLaughlin P., Upson B., Rosemarie A.H. (1986). Effects of α-and β-tigogenin cellobiosides on cholesterol absorption. Steroids.

[21] Sautour M., Miyamoto T., Lacaille-Dubois M.A. (2005). Steroidal saponins from *Smilax medica* and their antifungal activity. J. Nat. Prod..

[22] Matsuo Y., Watanabe K., Mimaki Y. (2009). Triterpene glycosides from the underground parts of *Caulophyllum thalictroides*.. J. Nat. Prod..

[23] Matsuo Y., Mimaki Y. (2010). Lignans from *Santalum album* and their cytotoxic activities. Chem. Pharm. Bull..

