# Supplementary material for: Cycloartane and Oleanane Glycosides from the Tubers of Eranthis cilicica
Source: Molecules. 2018 Dec 25;24(1):69. doi: 10.3390/molecules24010069 (PMC6337325; doi:10.3390/molecules24010069)

Cycloartane and Oleanane Glycosides from the Tubers of *Eranthis cilicica*

Kazuki Watanabe [1], Yoshihiro Mimaki [2], Haruhiko Fukaya [2], and Yukiko Matsuo [2,*]

Supplementary Materials: All NMR spectra of **1**, **1a**, **2-8**, **8a**, and **9-13** are available online.

Table 1. ^{1}H-NMR spectral data for compounds **1-7**, and **1a** in C_5D_5N

Table 2. ^{1}H-NMR spectral data for compounds **8-11**, and **8a** in C_5D_5N

NMR spectra of **1** in C_5D_5N_ page 3

NMR spectra of **1a** in C_5D_5N_ page 9

NMR spectra of **2** in C_5D_5N_ page 15

NMR spectra of **3** in C_5D_5N_ page 21

NMR spectra of **4** in C_5D_5N_ page 27

NMR spectra of **5** in C_5D_5N_ page 34

NMR spectra of **6** in C_5D_5N_ page 40

NMR spectra of **7** in C_5D_5N_ page 47

NMR spectra of **8** in C_5D_5N_ page 53

NMR spectra of **8a** in C_5D_5N_ page 60

NMR spectra of **9** in C_5D_5N_ page 66

NMR spectra of **10** in C_5D_5N_ page 73

NMR spectra of **11** in C_5D_5N_ page 79

NMR spectra of **13** in C_5D_5N_ page 86

Table 1. ^{1}H-NMR spectral data for compounds **1**－**7**, and **1a** in C_5D_5N

	1		1a		2		3		4		5		6		7	
	^{1}H	J (Hz)	^{1}H	J (Hz)	^{1}H	J (Hz)	^{1}H	J (Hz)	^{1}H	J (Hz)	^{1}H	J (Hz)	^{1}H	J (Hz)	^{1}H	J (Hz)
1	1.42 m		1.53 m		1.42 m		1.38 m		1.60 m		1.44 m		1.47 m		1.65 ddd	13.4, 13.0, 3.0
	1.08 m		1.22 m		1.08 m		1.04 m		1.20 m		1.06 m		1.18 m		1.23 m	
2	2.37 m		1.97 m		2.27 m		2.28 m		2.48 m		2.28 m		2.32 m		2.54 m	
	1.85 m		1.86 m		1.81 m		1.78 m		1.89 m		1.77 m		1.81 m		1.93 m	
3	3.46 dd	11.7, 4.3	3.48 dd	11.5, 4.5	3.40 dd	11.7, 4.3	3.40 dd	11.7, 4.3	3.44 dd	11.6, 4.3	3.38 dd	11.6, 4.2	3.41 dd	11.7, 4.3	3.52 dd	11.6, 4.3
5	1.23 dd	12.7, 3.7	1.28 dd	12.9, 3.6	1.24 dd	12.7, 3.8	1.20 dd	12.6, 4.0	1.17 dd	12.4, 3.6	1.19 m		1.17 dd	11.9, 4.3	1.20 m	
6	1.44 m		1.52 m		1.44 m		1.28 m		1.39 m		1.42 m		1.31 m		1.39 m	
	0.56 qd-like	11.1, 2.9	0.66 qd-like	12.1, 2.1	0.57 qd-like	11.2, 3.5	0.54 qd-like	10.9, 2.7	0.53 qd-like	12.1, 2.1	0.53 qd-like	11.3, 3.0	0.69 qd-like	11.9, 2.4	0.58 qd-like	12.5, 1.6
7	1.48 m		1.51 m		1.47 m		1.46 m		1.43 m		1.42 m		1.30 m		1.88 m	
	1.44 m		1.47 m		1.43 m		1.41 m		1.37 m		1.38 m		0.75 m		1.03 m	
8	1.65 m		1.70 m		1.64 m		1.60 m		1.60 m		1.60 m		1.84 m		1.49 m	
11	1.99 m		2.06 m		1.99 m		1.95 m		1.94 m		1.94 m		1.90 m		1.16 m	
	1.09 m		1.18 m		1.10 m		1.05 m		1.04 ddd	12.7, 10.8, 2.7	1.05 m		1.53 m		0.92 m	
12	1.74 m		1.78 m		1.74 m		1.71 m		1.66 m		1.68 m		1.63 m		1.49 m	
	1.56 m		1.61 m		1.56 m		1.53 m		1.49 m		1.51 m		1.58 m		1.44 m	
15	2.97 dd	12.7, 7.9	2.99 dd	12.8, 7.8	2.95 dd	12.7, 7.8	2.93 dd	12.7, 7.8	2.93 dd	12.7, 7.8	2.93 dd	12.7, 7.5	2.59 dd	13.0, 8.0	1.87 m	
	1.65 dd	12.7, 6.4	1.69 dd	12.8, 6.5	1.63 dd	12.7, 6.4	1.60 dd	12.7, 6.9	1.59 m		1.60 m		1.47 m		1.60 m	
16	4.50 ddd	8.3, 7.9, 6.4	4.50 ddd	8.4, 7.8, 6.5	4.49 ddd	8.4, 7.8, 6.4	4.50 ddd	8.4, 7.8, 6.9	4.49 m		4.50 q-like	7.5	4.42 q-like	8.0	4.06 m	
17	1.87 dd	10.3, 8.3	1.89 dd	9.9, 8.4	1.87 dd	10.2, 8.4	1.85 dd	10.3, 8.4	1.84 dd	9.6, 8.3	1.85 dd	9.3, 7.5	1.25 m		1.55 dd	10.0, 8.3
18	1.36 s		1.40 s		1.35 s		1.32 s		1.32 s		1.32 s		1.29 s		1.21 s	
19	0.48 d	4.0	0.56 d	4.1	0.47 d	4.0	0.44 d	3.9	0.44 d	3.9	0.43 d	3.7	0.62 d	4.4	0.43 d	4.0
	0.20 d	4.0	0.32 d	4.1	0.20 d	4.0	0.16 d	3.9	0.20 d	3.9	0.18 d	3.7	0.19 d	4.4	0.16 d	4.0
20	2.27 m		2.29 m		2.26 m		2.23 m		2.23 m		2.23 m		2.20 m		2.20 m	
21	0.97 d	6.5	0.99 d	6.5	0.97 d	6.6	0.95 d	6.5	0.96 d	6.5	0.95 d	6.5	0.91 d	6.6	0.97 d	6.6
22	1.56 dd	13.3, 2.6	1.57 dd	13.3, 2.6	1.55 dd	13.1, 2.2	1.54 dd	13.3, 2.4	1.54 dd	13.4, 2.5	1.54 dd	13.1, 1.6	1.34 m		1.57 dd	13.2, 3.1
	1.34 dd	13.3, 13.3	1.35 dd	13.3, 13.3	1.35 dd	13.1, 13.1	1.26 dd	13.3, 13.3	1.34 dd	13.4, 13.4	1.37 dd	13.1, 13.1	1.29 m		1.38 dd	13.2, 13.2
24	3.51 s		3.50 s		3.51 s		3.54 s		3.51 s		3.52 s		3.58 s		3.66 s	
26	4.02 d	10.3	4.03 d	10.1	4.02 d	10.2	4.01 d	10.3	4.00 d	10.3	4.01 d	10.2	4.03 d	10.3	4.02 d	10.3
	3.59 d	10.3	3.60 d	10.1	3.59 d	10.2	3.58 d	10.3	3.58 d	10.3	3.58 d	10.2	3.58 d	10.3	3.60 d	10.3
27	1.42 s		1.43 s		1.42 s		1.41 s		1.41 s		1.41 s		1.44 s		1.45 s	
28	4.02 d	11.3	4.05 d	11.4	4.02 d	11.5	4.00 d	11.3	3.96 d	11.4	3.98 d	11.4	10.14 s		0.80 s	
	3.94 d	11.3	3.96 d	11.4	3.94 d	11.5	3.93 d	11.3	3.89 d	11.4	3.90 d	11.4				
29	1.29 s		1.17 s		1.27 s		1.24 s		1.21 s		1.22 s		1.27 s		1.28 s	
30	1.03 s		1.07 s		1.02 s		0.99 s		1.00 s		0.99 s		1.00 s		1.02 s	
1'	4.94 d	7.8			4.87 d	7.8	4.86 d	7.8	4.86 d	7.7	4.80 d	7.5	4.90 d	7.8	4.90 d	7.7
2'	4.05 dd	8.2, 7.8			4.03 dd	8.7, 7.8	4.03 dd	8.3, 7.8	3.97 dd	8.1, 7.7	3.87 dd	8.4, 7.5	4.05 dd	8.6, 7.8	3.99 dd	8.7, 7.7
3'	4.27 dd	8.7, 8.2			4.26 dd	8.7, 8.7	4.20 dd	9.0, 8.3	4.19 dd	8.9, 8.1	4.17 dd	9.1, 8.4	4.29 dd	8.6, 8.4	4.18 dd	9.1, 8.7
4'	4.25 dd	9.1, 8.7			4.30 dd	9.8, 8.7	4.07 dd	9.4, 9.0	4.07 dd	8.9, 8.9	4.37 dd	9.1, 9.1	4.33 dd	8.9, 8.4	4.09 dd	9.1, 7.9
5'	3.98 ddd	9.1, 5.3, 2.4			3.92 ddd	9.8, 3.9, 2.1	3.88 ddd	9.4, 5.0, 2.0	4.09 m		3.96 m		3.97 m		4.10 m	
6' a	4.58 dd	11.7, 2.4			4.56 dd	12.0, 3.9	4.44 dd	11.9, 2.0	4.82 br d	11.0	4.82 br d	11.2	4.59 dd	11.9, 4.0	4.85 br d	10.7
b	4.42 dd	11.7, 5.3			4.48 dd	12.0, 2.1	4.27 dd	11.9, 5.0	4.31 dd	11.0, 5.3	4.63 dd	11.2, 3.8	4.30 br d	11.9	4.34 dd	12.1, 5.5
1"					5.20 d	7.9	5.28 d	7.9	5.28 d	7.8	5.45 d	7.9	5.21 d	7.9	5.16 d	7.8
2"					4.09 dd	8.3, 7.9	4.04 dd	8.3, 7.9	4.03 dd	8.8, 7.8	4.03 dd	8.9, 7.9	4.12 dd	8.6, 7.9	4.19 dd	8.7, 7.8
3"					4.24 dd	8.8, 8.3	4.25 dd	9.0, 8.3	4.24 dd	8.8, 8.8	4.35 dd	9.1, 8.9	4.24 dd	8.6, 8.6	4.22 dd	9.1, 8.7
4"					4.20 dd	9.1, 8.8	4.16 dd	9.5, 9.0	4.21 dd	8.8, 8.4	4.19 dd	9.5, 9.1	4.20 dd	8.6, 8.6	4.23 dd	9.1, 8.1
5"					3.99 ddd	9.1, 5.7, 2.1	3.98 ddd	9.5, 5.5, 2.1	3.90 ddd	8.4, 5.2, 2.0	4.13 m		4.01 ddd	8.6, 4.8, 2.3	3.92 ddd	8.1, 5.2, 2.5
6" a					4.50 dd	11.7, 2.1	4.49 dd	11.7, 2.1	4.47 dd	11.8, 2.0	4.45 br d	11.7	4.53 dd	12.0, 2.3	4.51 dd	11.9, 2.5
b					4.28 dd	11.7, 5.7	4.28 dd	11.7, 5.5	4.34 dd	11.8, 5.2	4.25 dd	11.7, 4.7	4.32 dd	12.0, 4.8	4.37 dd	11.9, 5.2
1'''											5.34 d	7.8				
2'''											4.01 dd	8.4, 7.8				
3'''											4.25 dd	9.3, 8.4				
4'''											4.22 dd	9.3, 8.8				
5'''											3.93 m					
6''' a											4.43 br d	12.0				
b											4.31 dd	12.0, 4.1				

Table 2. ^{1}H-NMR spectral data for compounds **8**－**11**, and **8a** in C_5D_5N

	8		8a		9		10		11	
	^{1}H	J (Hz)	^{1}H	J (Hz)	^{1}H	J (Hz)	^{1}H	J (Hz)	^{1}H	J (Hz)
1	1.44 ddd	13.5, 12.2, 3.0	1.53 ddd	13.1, 11.5, 2.9	1.43 m		1.65 m		1.62 ddd	13.2, 11.6, 3.1
	1.11 m		1.21 m		1.09 m		1.23 m		1.21 m	
2	2.29 m		1.98 m		2.33 m		2.53 m		2.51 m	
	1.84 m		1.88 qd-like	11.5, 3.6	1.82 m		1.93 m		1.92 m	
3	3.42 dd	11.7, 4.3	3.49 dd	11.5, 4.4	3.45 dd	11.7, 4.3	3.48 dd	11.5, 4.0	3.51 ddd	11.6, 4.3
5	1.28 dd	11.9, 2.9	1.30 dd	12.5, 3.6	1.27 dd	11.7, 3.3	1.25 m		1.21 m	
6	1.51 m		1.54 m		1.53 m		1.46 m		1.41 m	
	0.65 qd-like	11.9, 2.7	0.70 qd-like	11.9, 2.6	0.65 qd-like	11.8, 2.8	0.62 qd-like	12.5, 2.5	0.61 qd-like	12.4, 2.1
7	1.56 m		1.57 m		1.56 m		1.52 m		1.19 m	
	1.51 m		1.54 m		1.54 m		1.48 m		0.94 m	
8	1.70 m		1.71 dd	12.5, 4.6	1.70 m		1.66 m		1.51 m	
11	2.00 m		2.03 m		1.99 m		1.95 m		1.88 m	
	1.12 ddd	12.6, 10.7, 4.2	1.15 m		1.10 m		1.07 m		1.02 m	
12	1.71 m		1.72 m		1.72 m		1.65 m		1.44 m	
	1.57 m		1.58 m		1.57 m		1.51 m		1.42 m	
15	2.99 dd	12.6, 7.9	3.01 dd	12.6, 8.0	3.00 dd	12.5, 8.0	2.99 dd	12.4, 8.0	1.88 m	
	1.57 m		1.58 dd	12.6, 6.4	1.57 m		1.54 m		1.52 m	
16	4.79 q-like	7.9	4.80 q-like	7.6	4.79 q-like	8.0	4.78 q-like	8.0	4.48 q-like	7.6
17	1.83 m		1.84 m		1.85 m		1.82 m		1.53 m	
18	1.32 s		1.34 s		1.32 s		1.30 s		1.15 s	
19	0.55 d	4.0	0.60 d	3.8	0.55 d	3.9	0.53 d	3.6	0.46 d	4.0
	0.26 d	4.0	0.34 d	3.8	0.25 d	3.9	0.26 d	3.6	0.13 d	4.0
20	1.84 m		1.85 m		1.84 m		1.82 m		1.76 m	
21	0.90 d	5.8	0.90 d	5.8	0.90 d	5.6	0.90 d	5.3	0.88 d	6.4
22	2.17 dd	12.4, 1.9	2.16 dd	13.3, 2.0	2.17 br d	12.7	2.16 br d	13.6	2.14 dd	13.9, 2.5
	1.57 dd	12.4, 12.4	1.56 dd	13.3, 13.3	1.57 dd	12.7, 12.7	1.56 dd	13.6, 13.6	1.56 dd	13.9, 13.9
24	3.68 s		3.69 s		3.68 s		3.67 s		3.64 s	
26	3.89 d	10.0	3.88 d	10.0	3.89 d	10.0	3.88 d	9.9	3.91 d	10.0
	3.83 d	10.0	3.82 d	10.0	3.83 d	10.0	3.82 d	9.9	3.83 d	10.0
27	1.42 s		1.42 s		1.42 s		1.42 s		1.41 s	
28	4.02 d	11.6	4.03 d	11.4	4.03 d	11.3	3.98 d	11.5	0.79 s	
	3.93 d	11.6	3.94 d	11.4	3.94 d	11.3	3.89 d	11.5		
29	1.29 s		1.17 s		1.29 s		1.25 s		1.28 s	
30	1.05 s		1.07 s		1.05 s		1.04 s		1.02 s	
1'	4.88 d	7.8			4.89 d	7.8	4.89 d	7.7	4.90 d	7.8
2'	4.04 dd	8.2, 7.8			4.07 dd	9.4, 7.8	4.00 dd	8.7, 7.7	3.99 dd	8.7, 7.8
3'	4.27 dd	8.9, 8.2			4.22 dd	9.4, 8.6	4.21 dd	8.7, 8.7	4.22 dd	8.7, 8.3
4'	4.31 dd	9.1, 8.9			4.11 dd	9.3, 8.6	4.12 dd	9.8, 8.7	4.11 dd	8.3, 8.3
5'	3.93 ddd	9.1, 3.9, 2.7			3.91 ddd	9.3, 4.6, 1.8	4.11 m		4.10 m	
6' a	4.57 dd	12.0, 3.9			4.48 dd	11.8, 1.8	4.85 br d	11.3	4.82 br d	11.1
b	4.49 dd	12.0, 2.7			4.30 dd	11.8, 4.6	4.33 dd	11.3, 5.2	4.33 dd	11.1, 5.1
1"	5.20 d	7.9			5.31 d	7.9	5.16 d	7.8	5.15 d	8.0
2"	4.10 dd	8.4, 7.9			4.08 dd	8.3, 7.9	4.06 dd	8.4, 7.8	4.03 dd	9.7, 8.0
3"	4.23 dd	8.8, 8.4			4.27 dd	8.9, 8.3	4.25 dd	8.9, 8.4	4.25 dd	9.7, 8.8
4"	4.20 dd	9.5, 8.8			4.20 dd	8.9, 8.9	4.24 dd	8.9, 8.4	4.22 dd	8.8, 8.8
5"	3.99 ddd	9.5, 5.4, 2.5			4.03 ddd	8.9, 5.7, 2.2	3.93 ddd	8.4, 4.9, 2.1	3.90 ddd	8.8, 5.1, 2.4
6" a	4.52 dd	11.6, 2.5			4.53 dd	11.7, 2.2	4.50 dd	11.4, 2.1	4.47 dd	12.1, 2.4
b	4.30 dd	11.6, 5.4			4.32 dd	11.7, 5.7	4.37 dd	11.4, 4.9	4.36 dd	12.1, 5.1

^{1}H-NMR spectrum of **1** in C_5D_5N

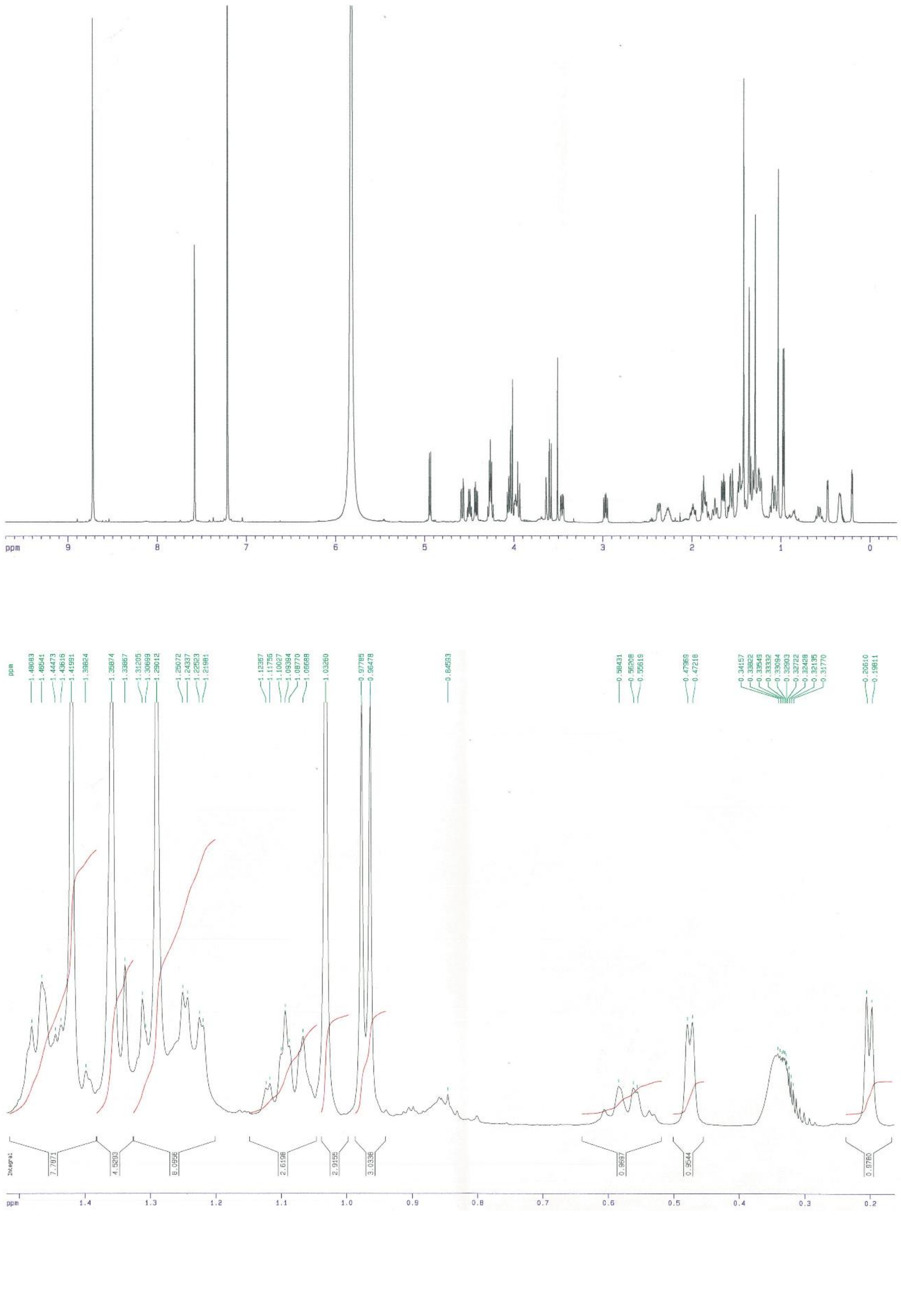

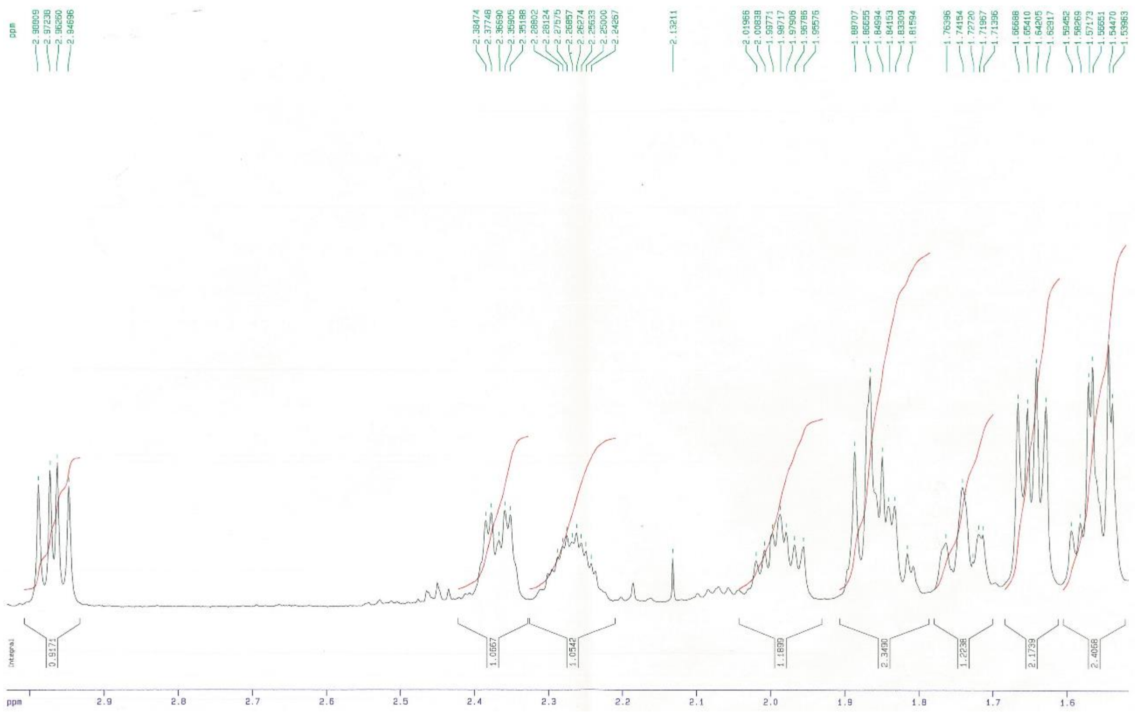

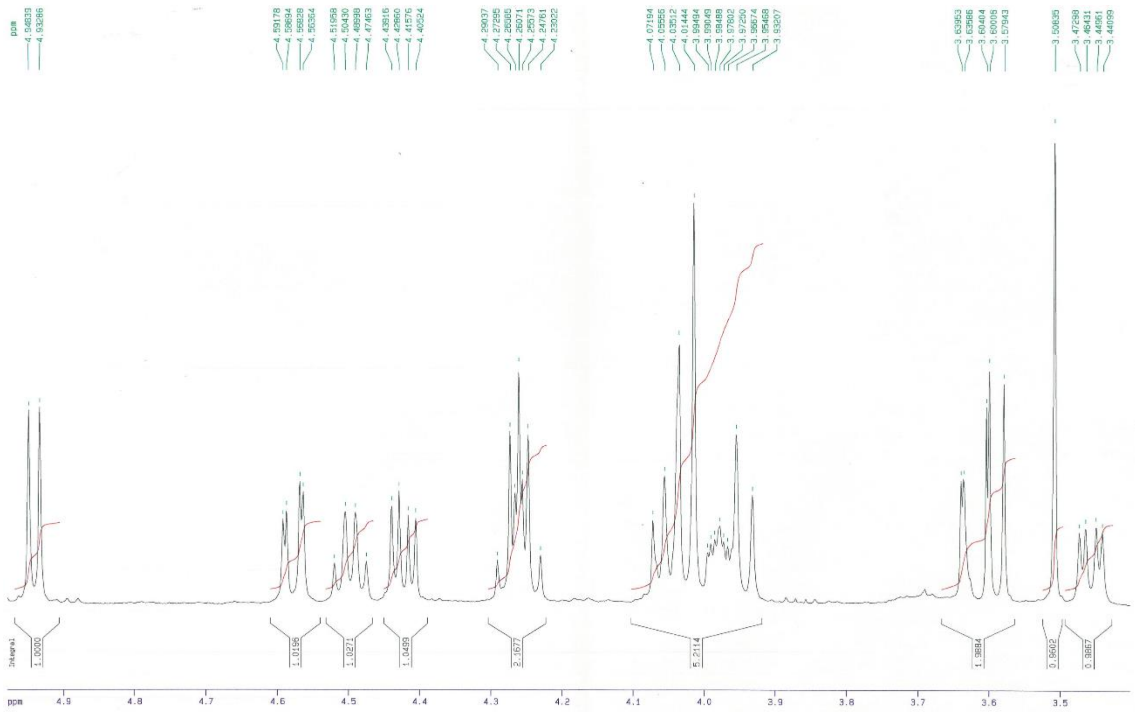

^{13}C-NMR spectrum of **1** in C_5D_5N

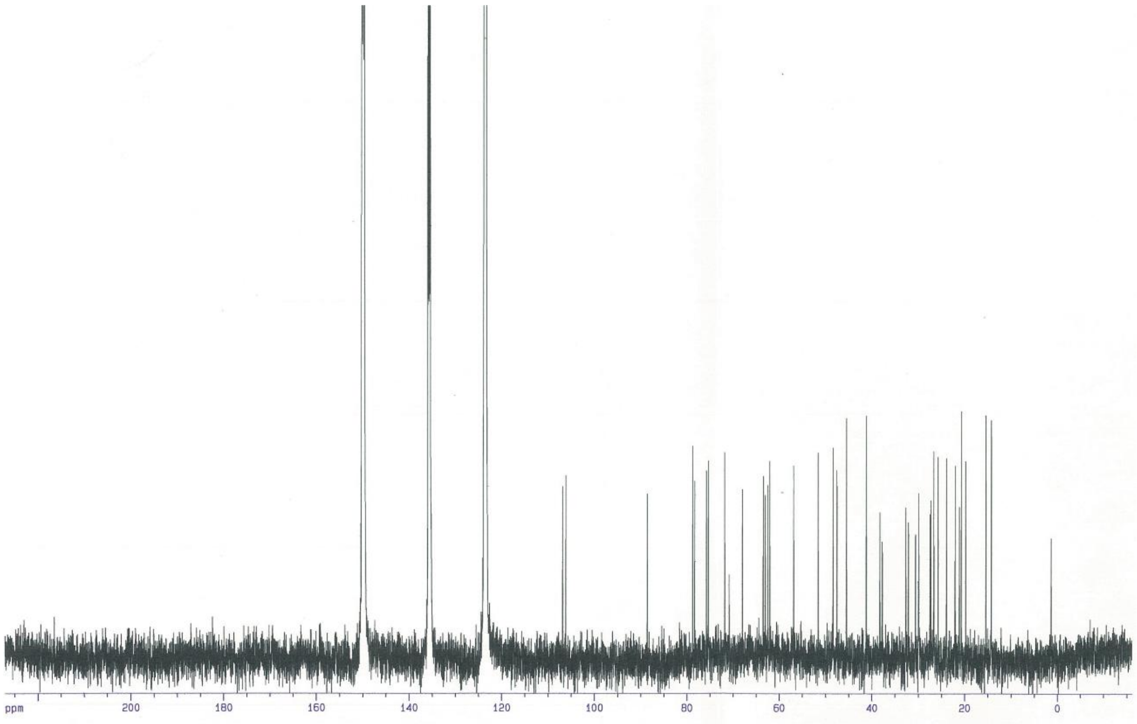

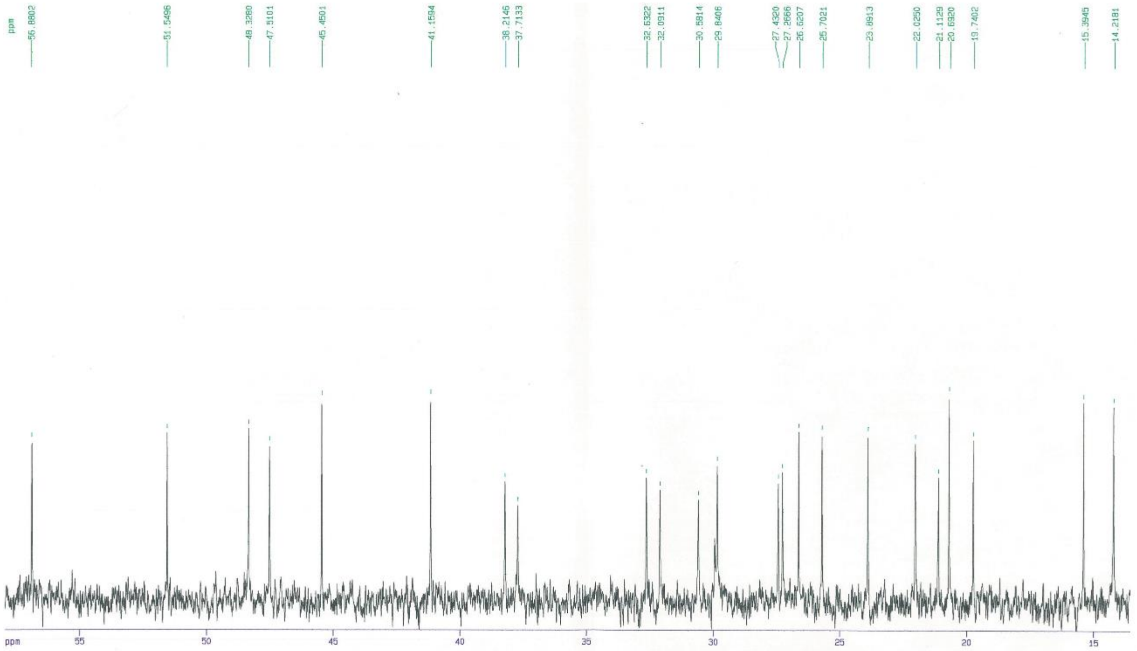

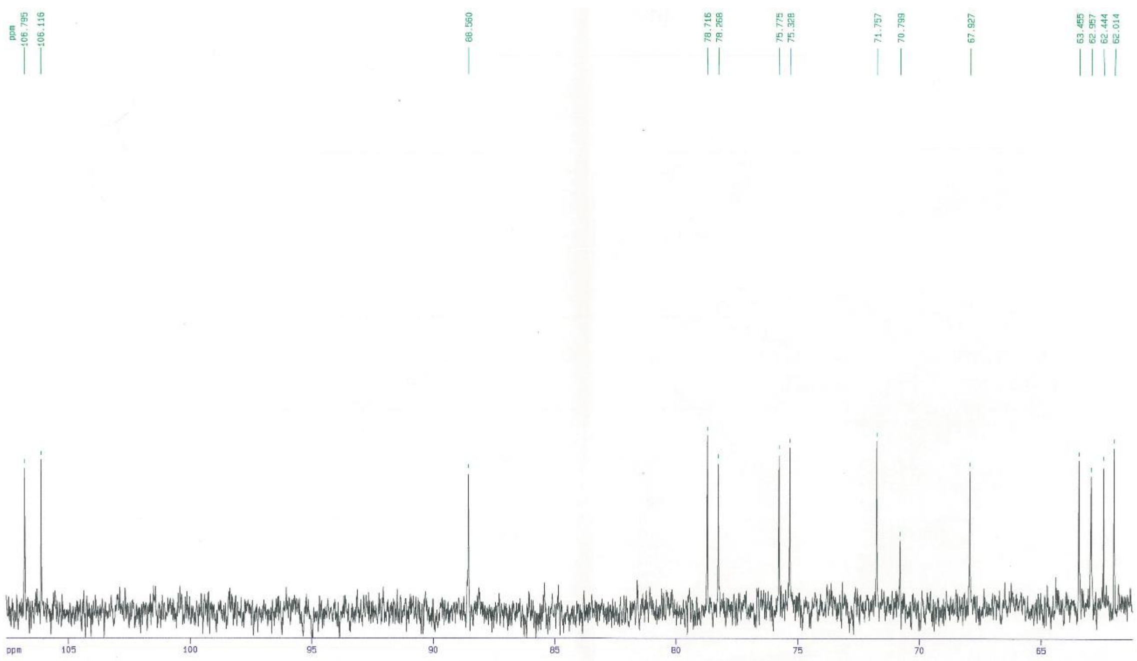

COSY spectrum of **1** in C_5D_5N

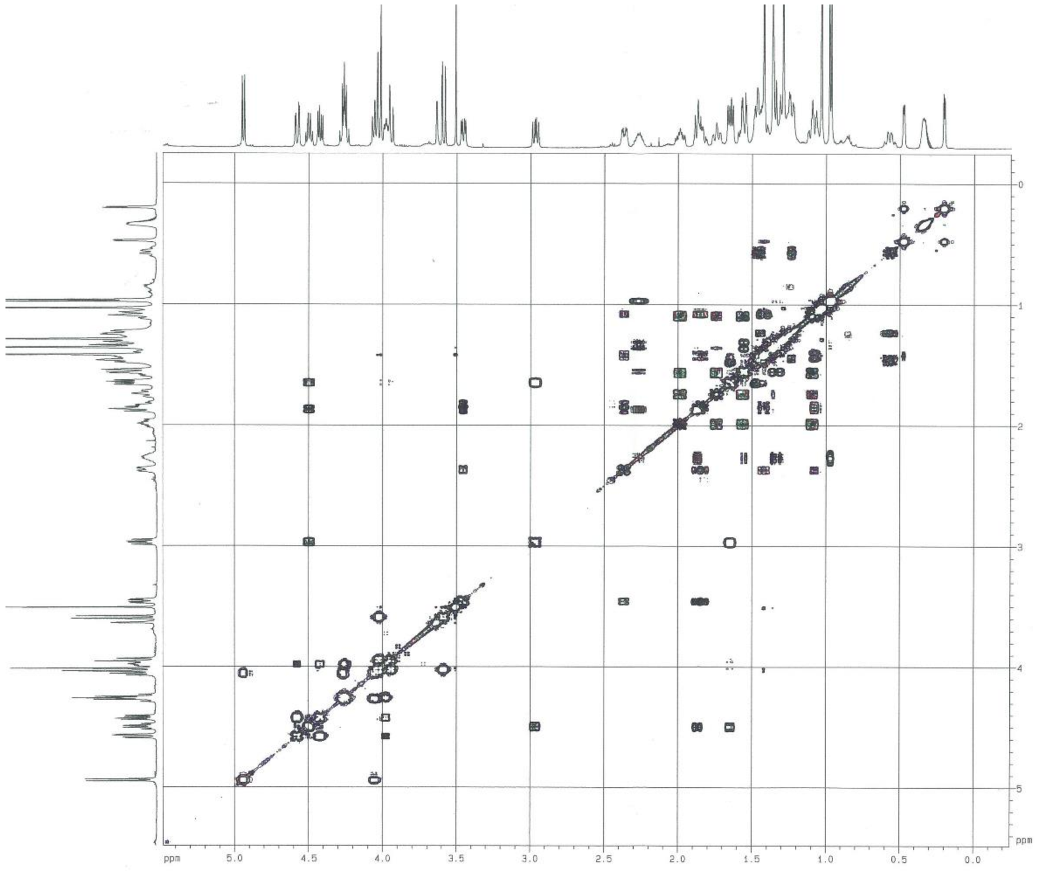

HMQC spectrum of **1** in C_5D_5N

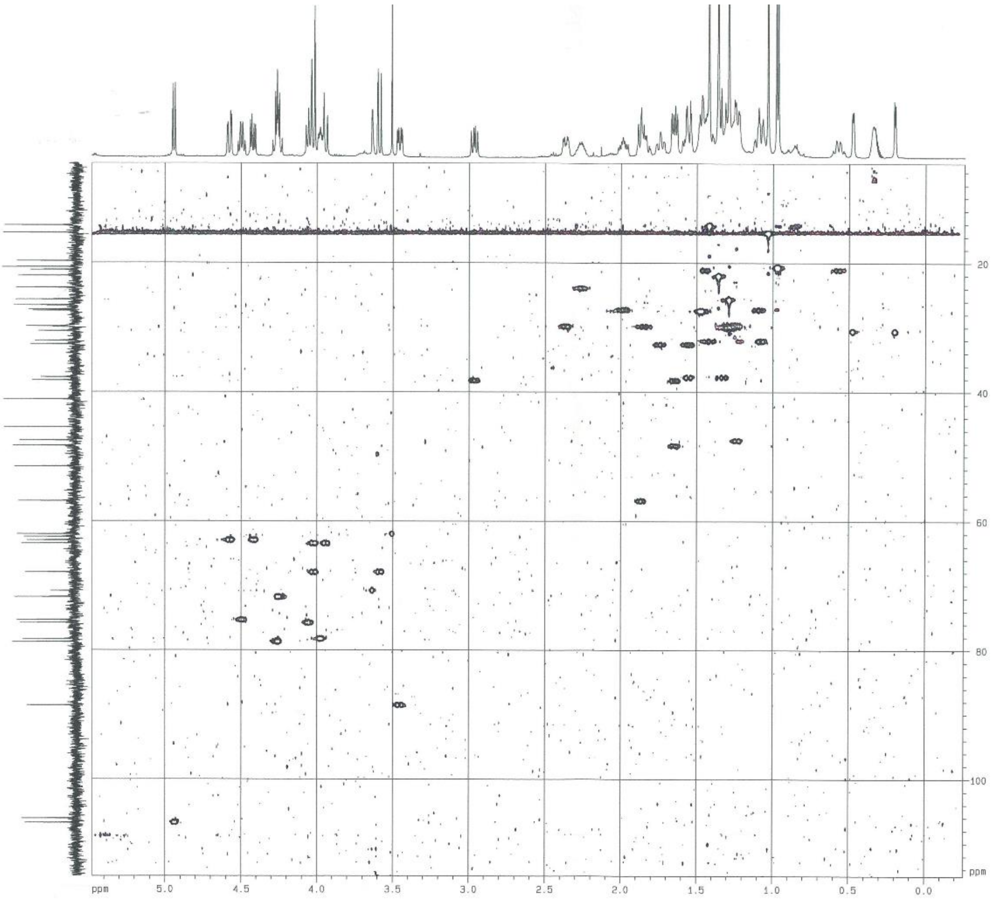

HMBC spectrum of **1** in C_5D_5N

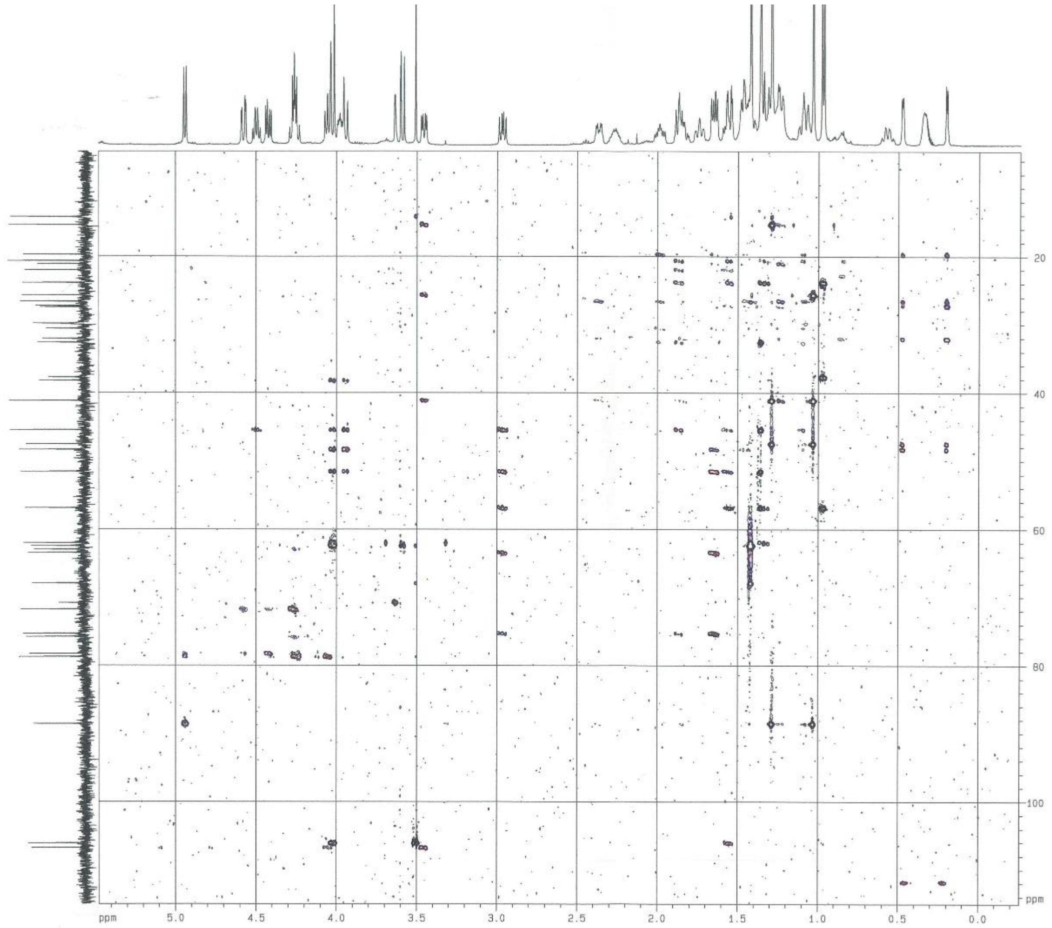

NOESY spectrum of **1** in C_5D_5N

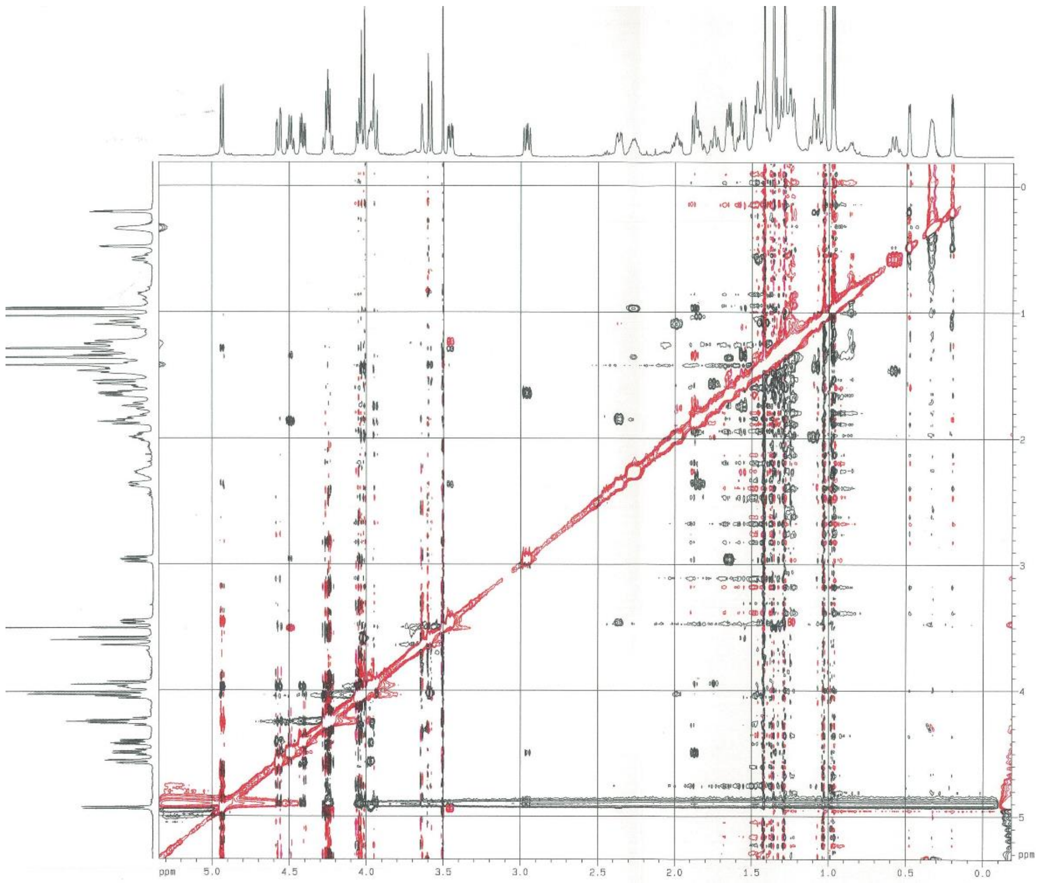

^{1}H-NMR spectrum of **1a** in C_5D_5N

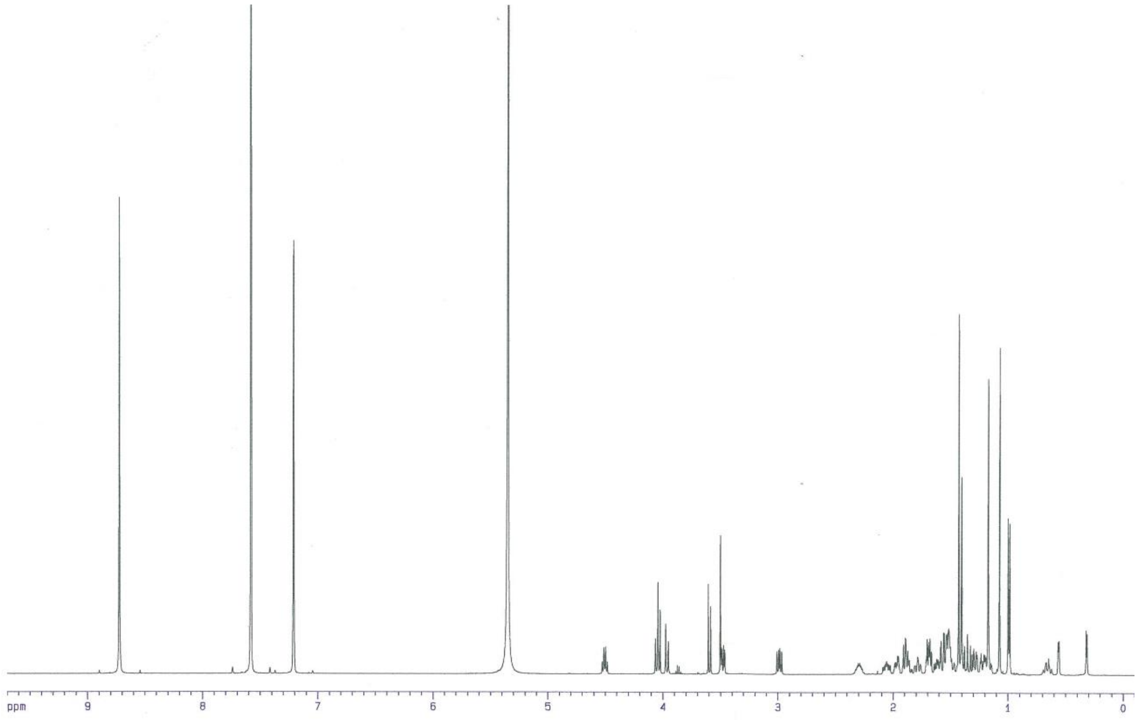

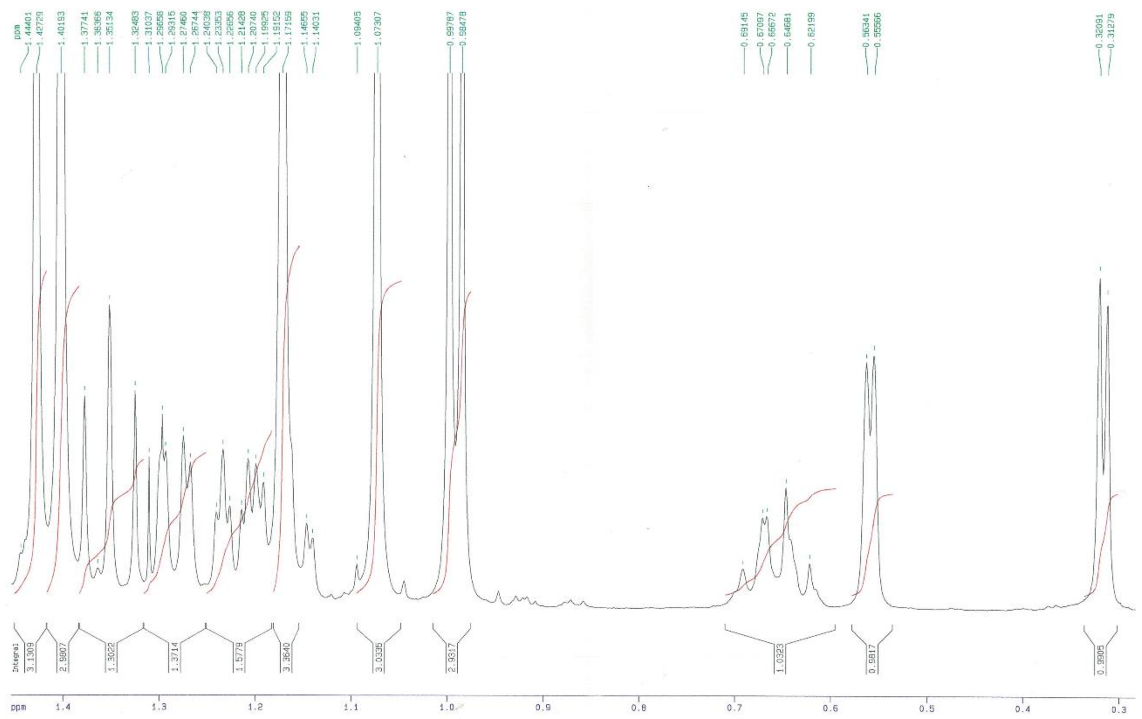

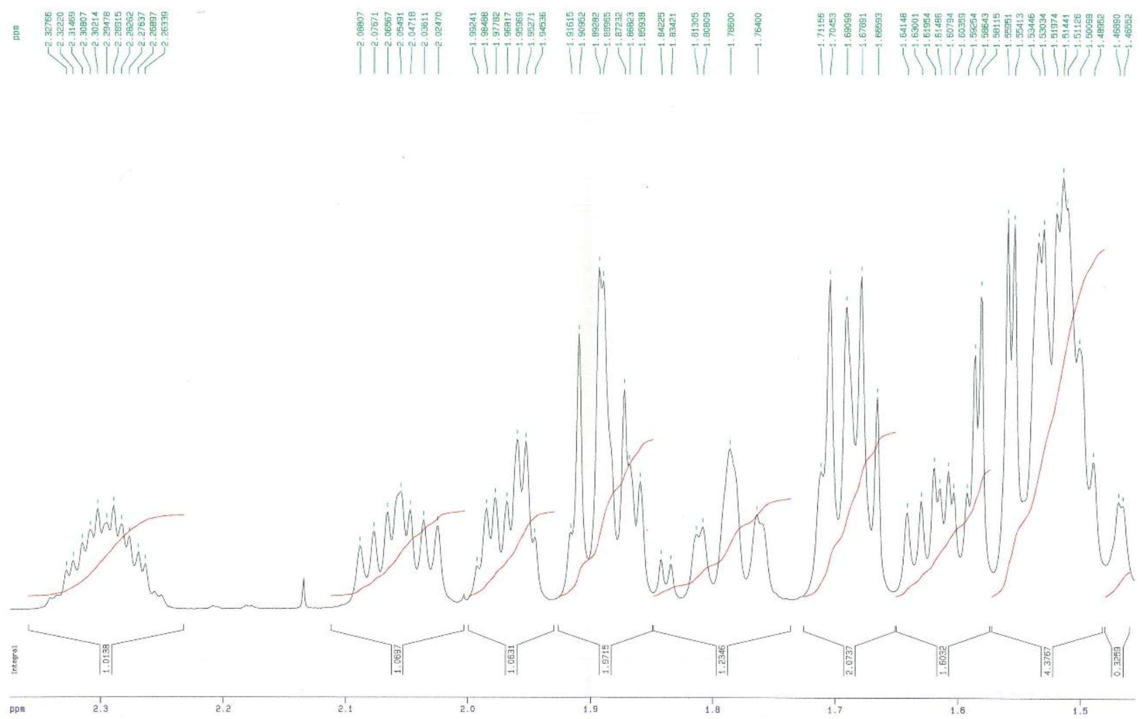

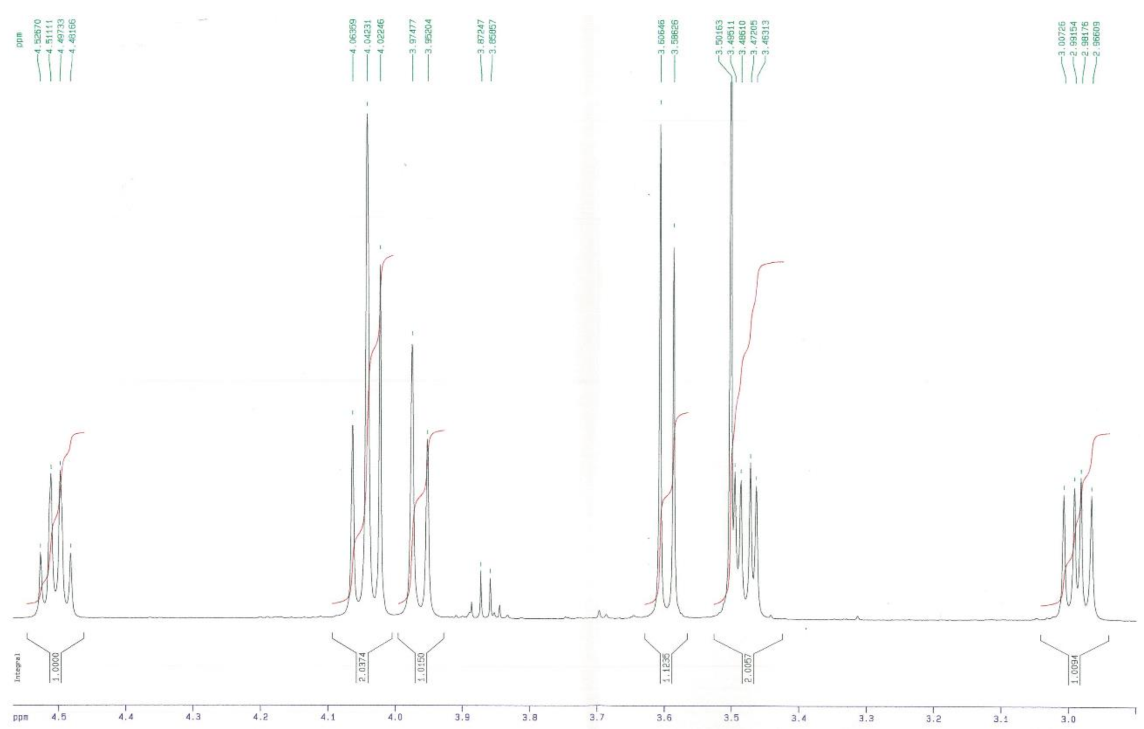

^{13}C-NMR spectrum of **1a** in C_5D_5N

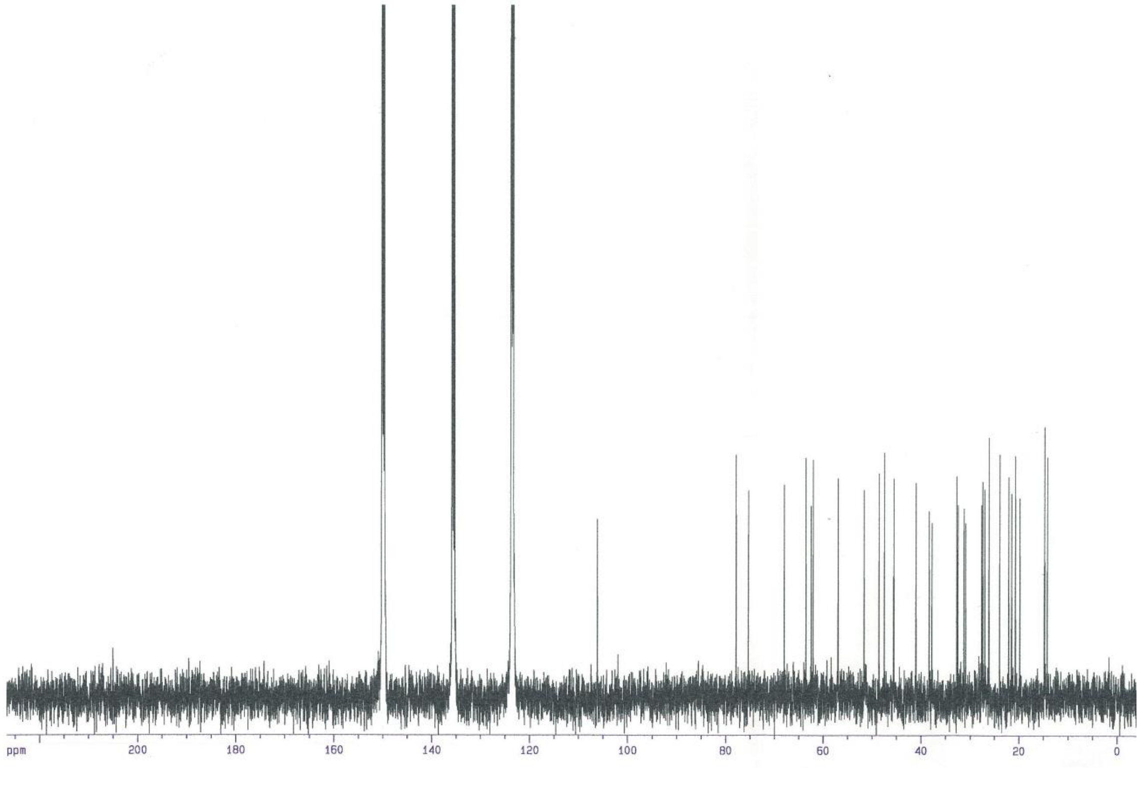

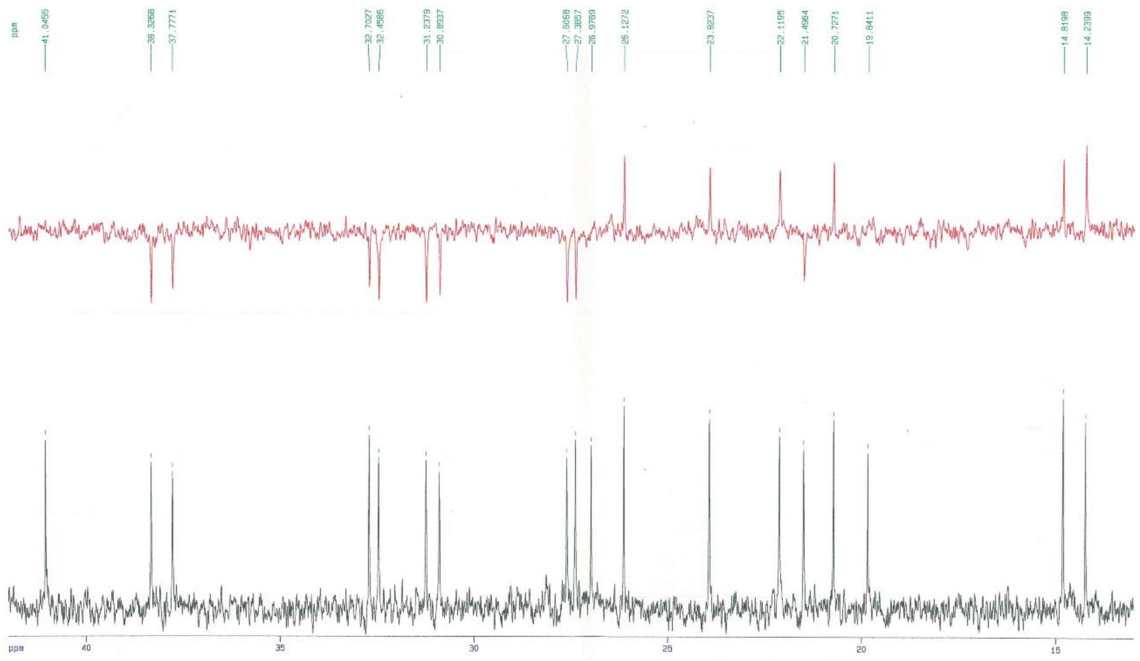
ppm
41.0455
38.3268
37.7771
32.7027
32.4586
31.2379
30.8937
27.6068
27.3857
26.9769
26.1272
23.8237
22.1195
21.4964
20.7271
19.8411
14.8198
14.2399
ppm 40 35 30 25 20 15

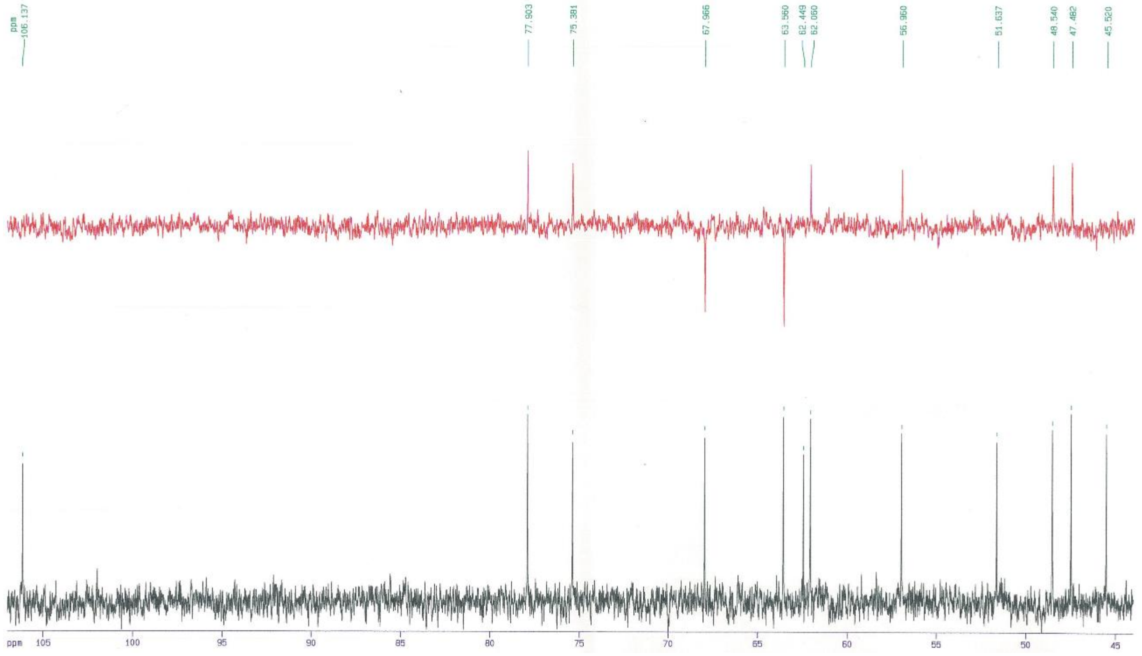
ppm
106.137
77.903
75.381
67.966
63.560
62.449
62.060
56.960
51.637
48.540
47.482
45.520
ppm 105 100 95 90 85 80 75 70 65 60 55 50 45

COSY spectrum of **1a** in C_5D_5N

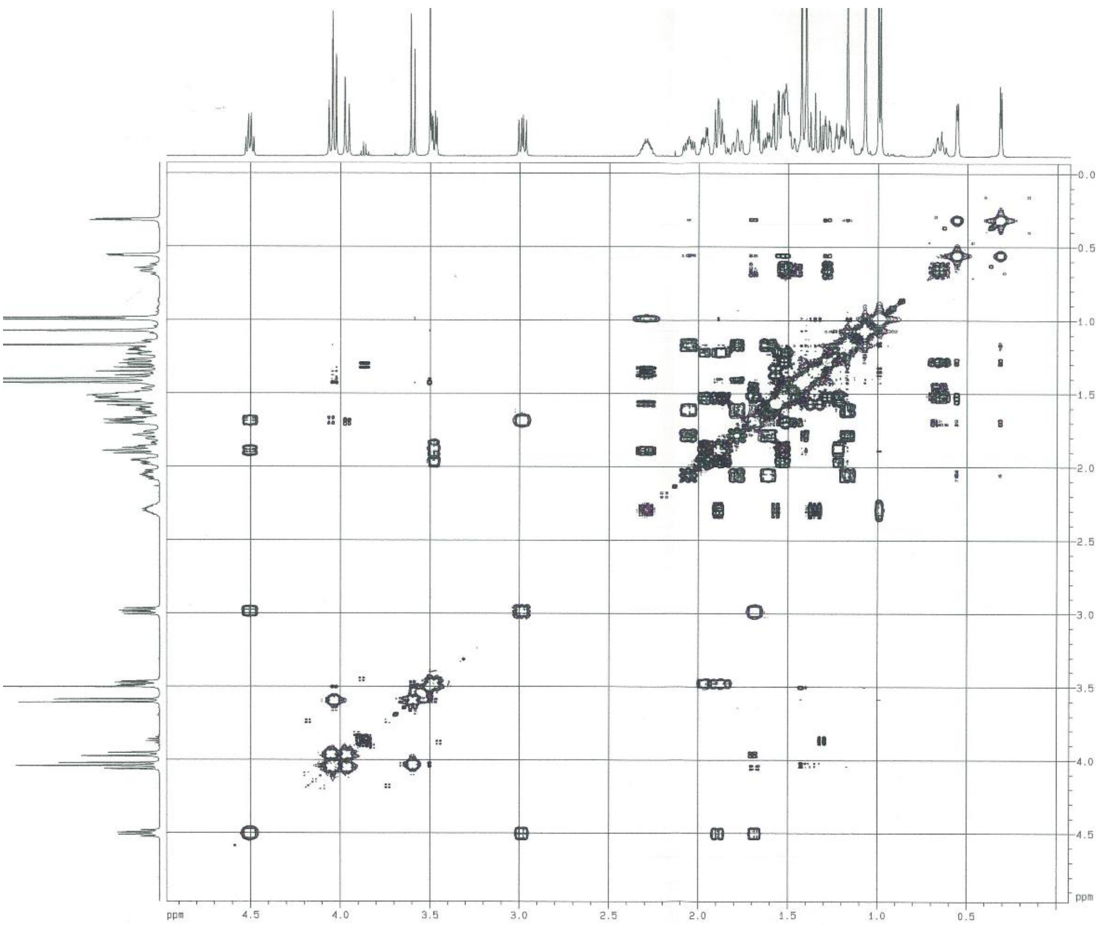

HMQC spectrum of **1a** in C_5D_5N

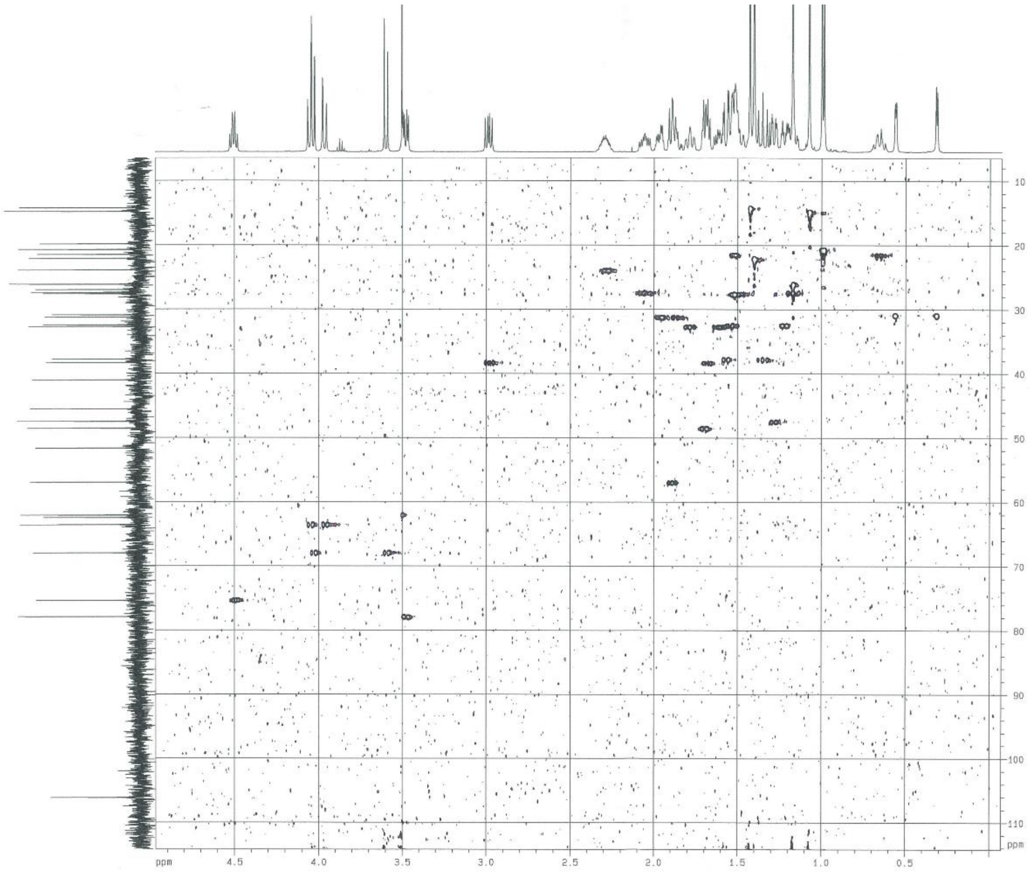

HMBC spectrum of **1a** in C_5D_5N

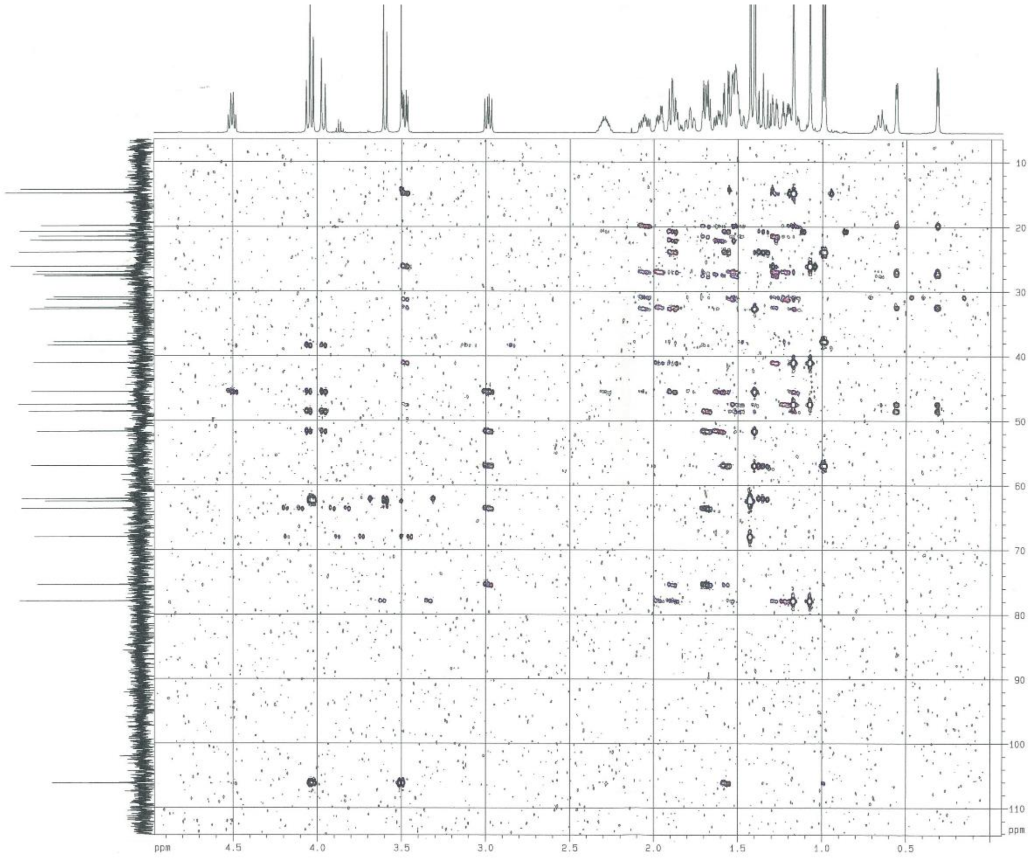

NOESY spectrum of **1a** in C_5D_5N

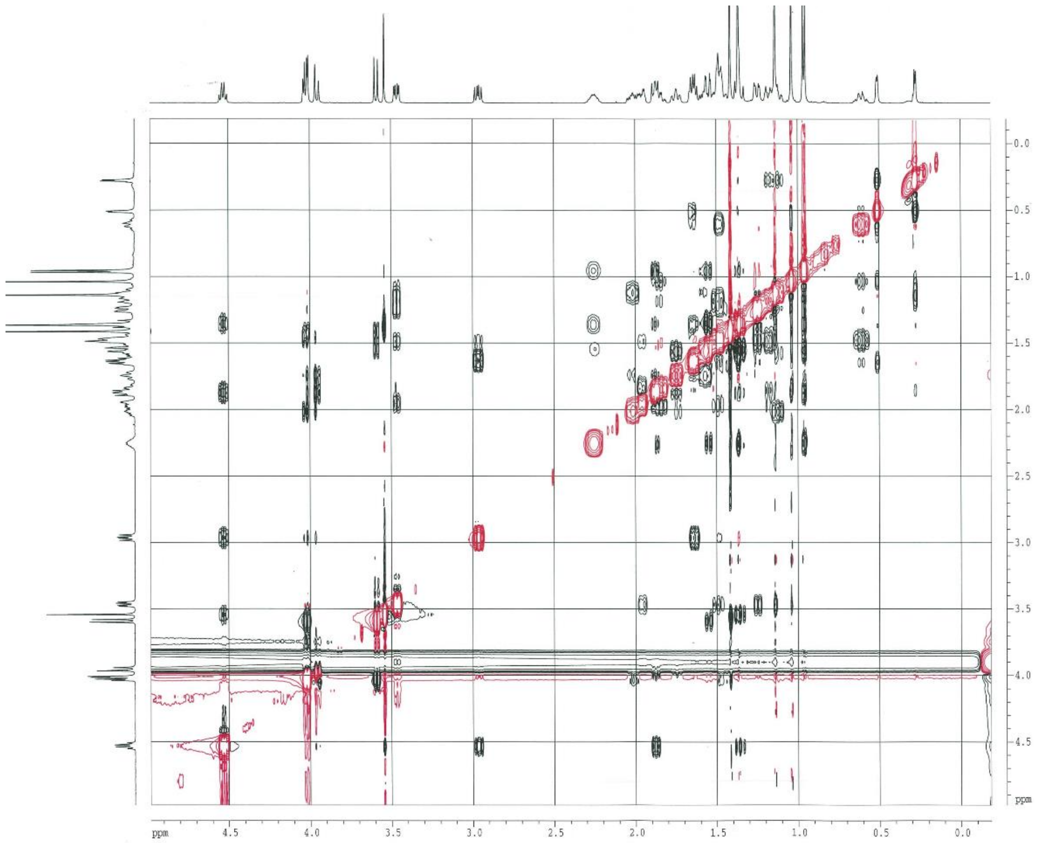

^{1}H-NMR spectrum of **2** in C_5D_5N

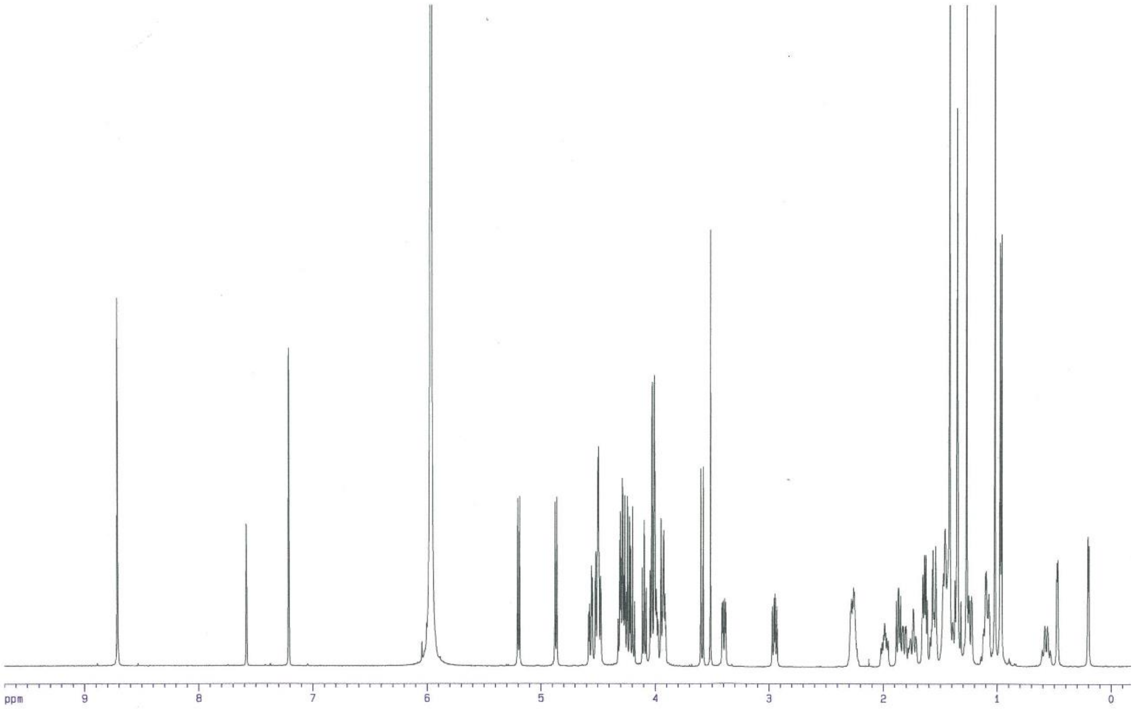

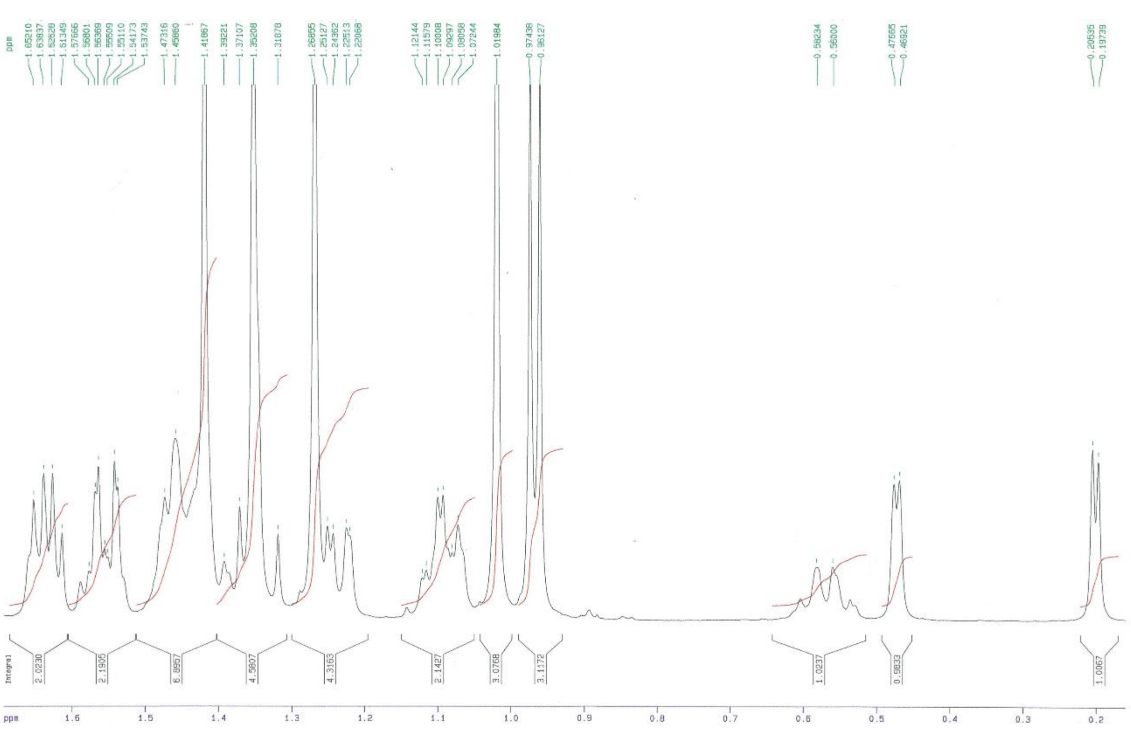

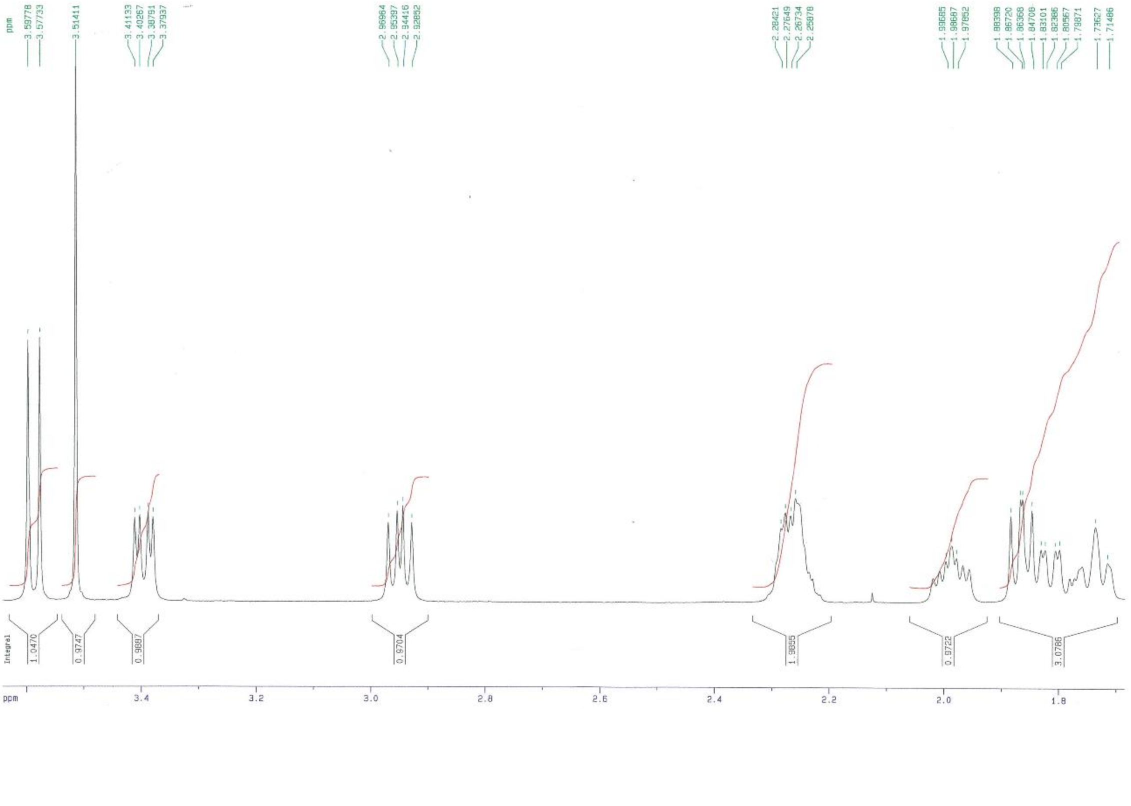

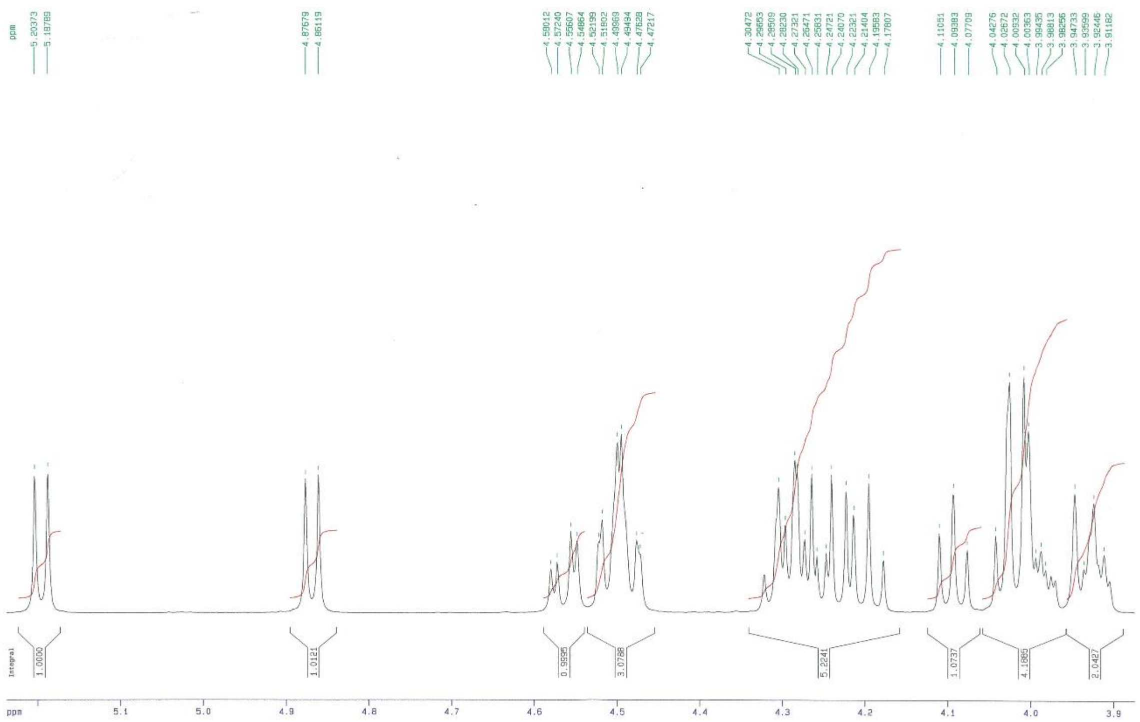

^{13}C-NMR spectrum of **2** in C_5D_5N

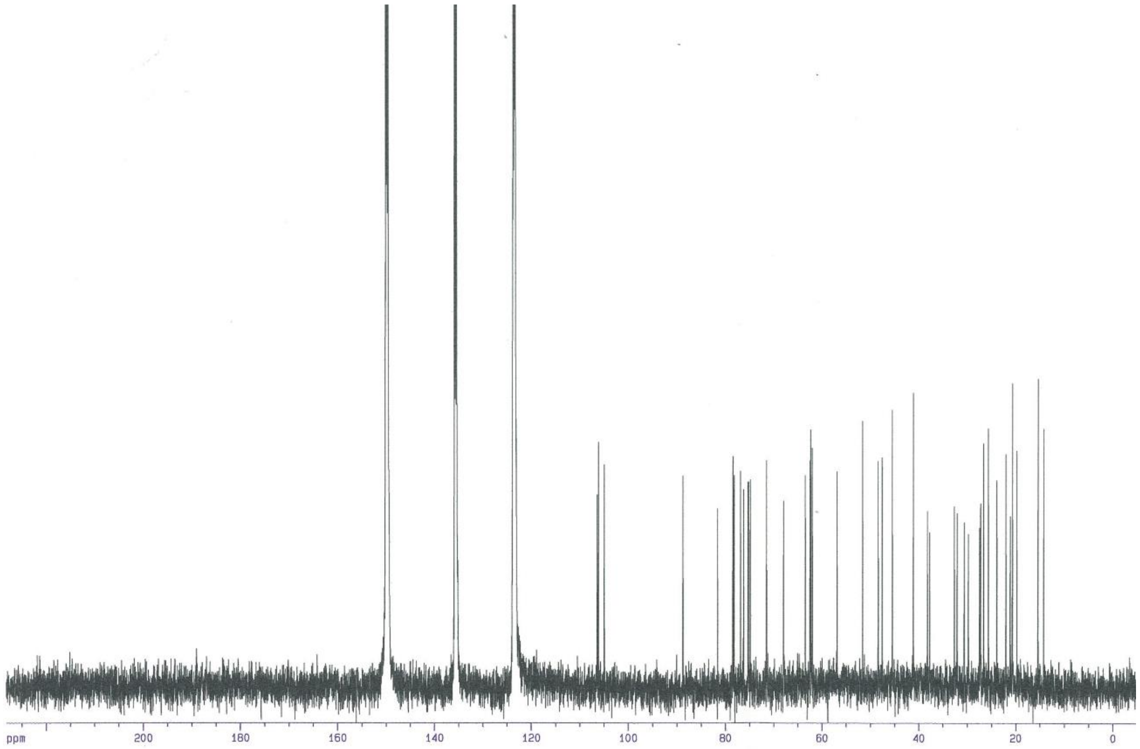

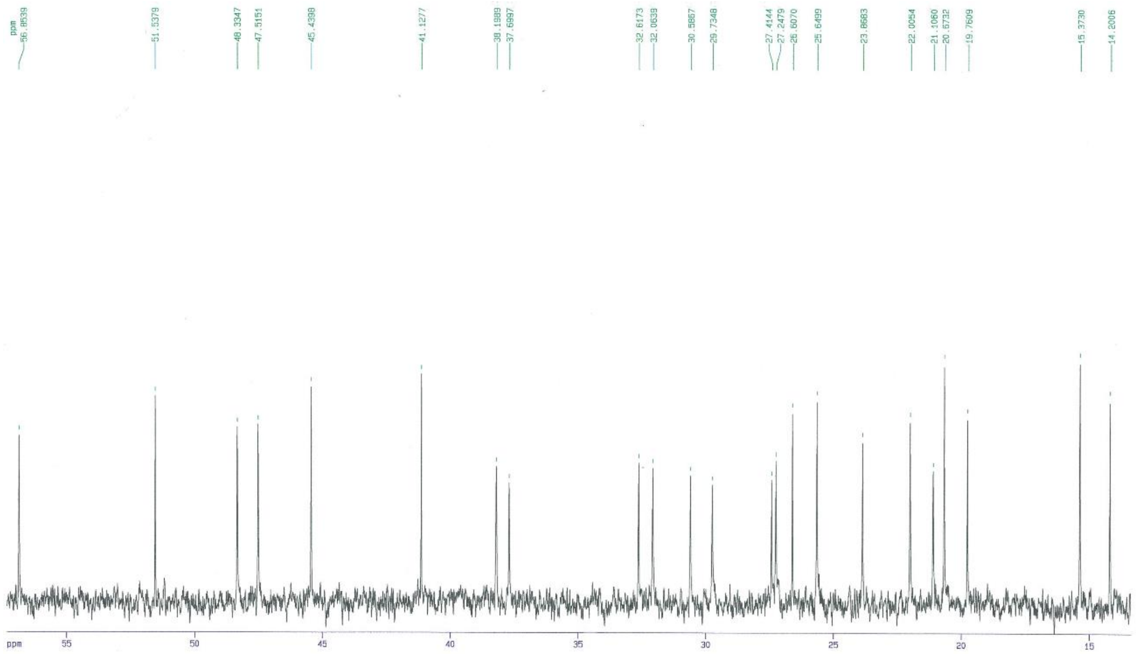

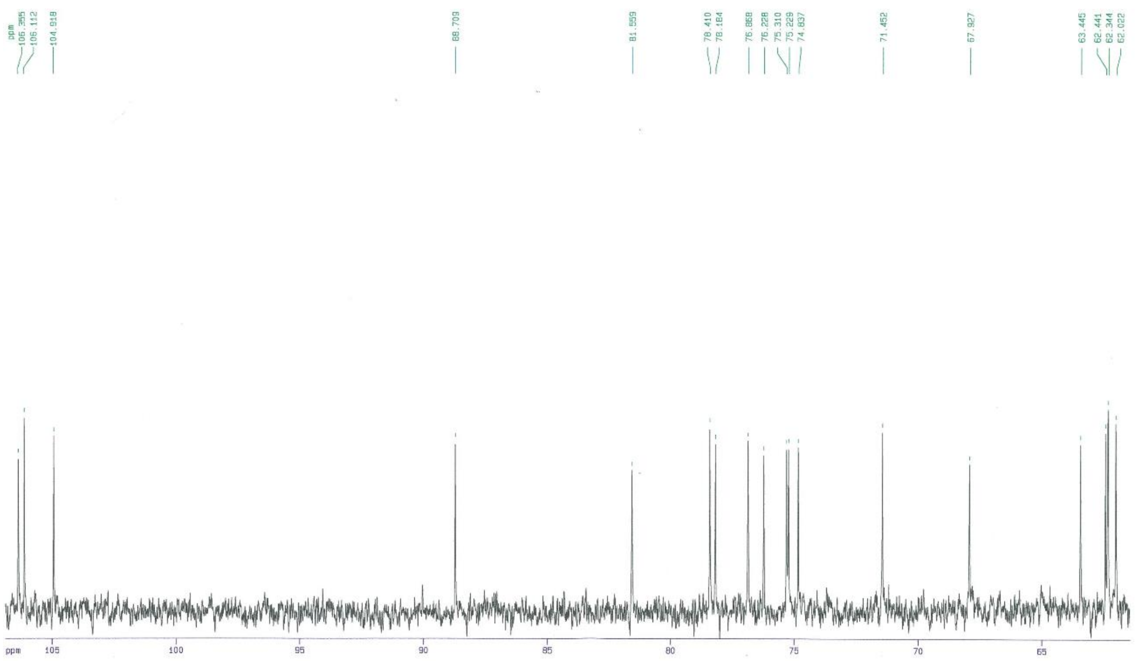

COSY spectrum of **2** in C_5D_5N

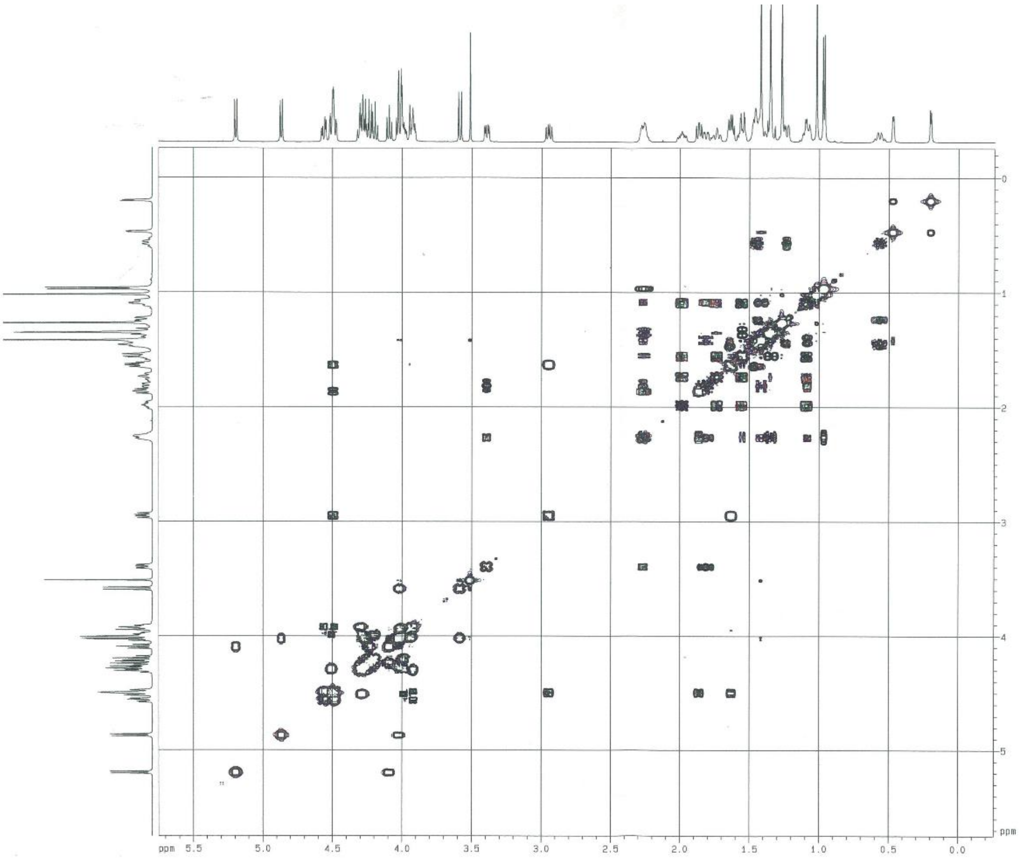

HMQC spectrum of **2** in C_5D_5N

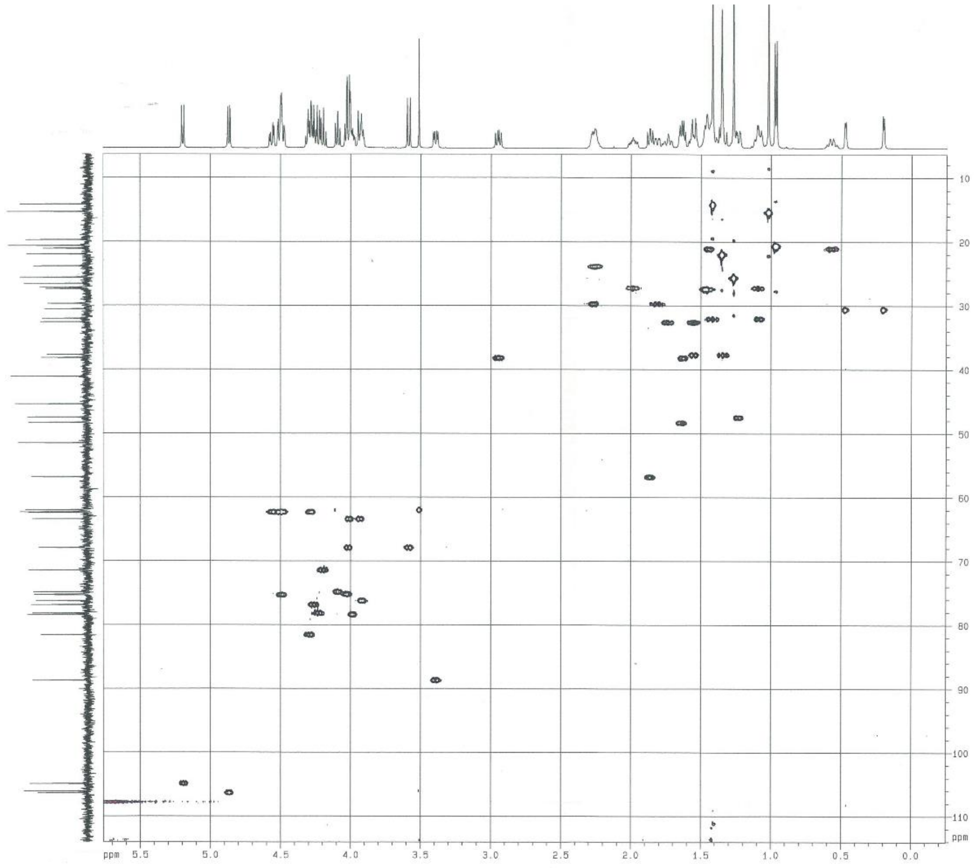

HMBC spectrum of **2** in C_5D_5N

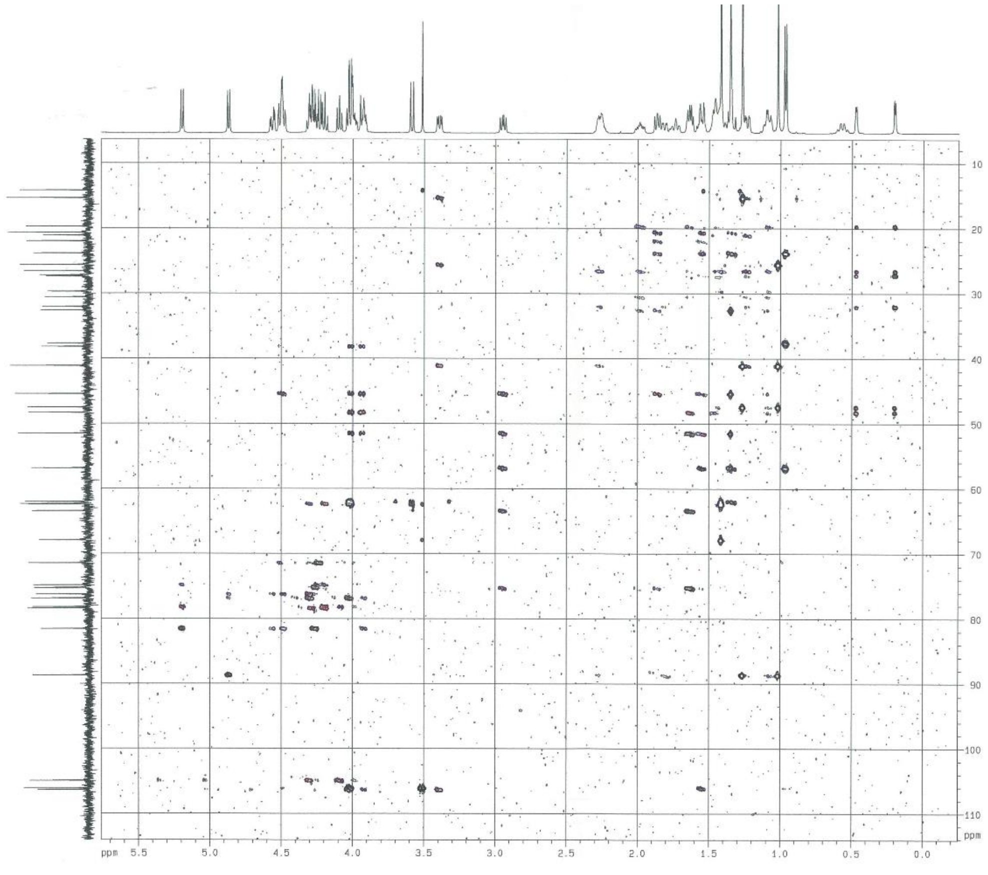

NOESY spectrum of **2** in C_5D_5N

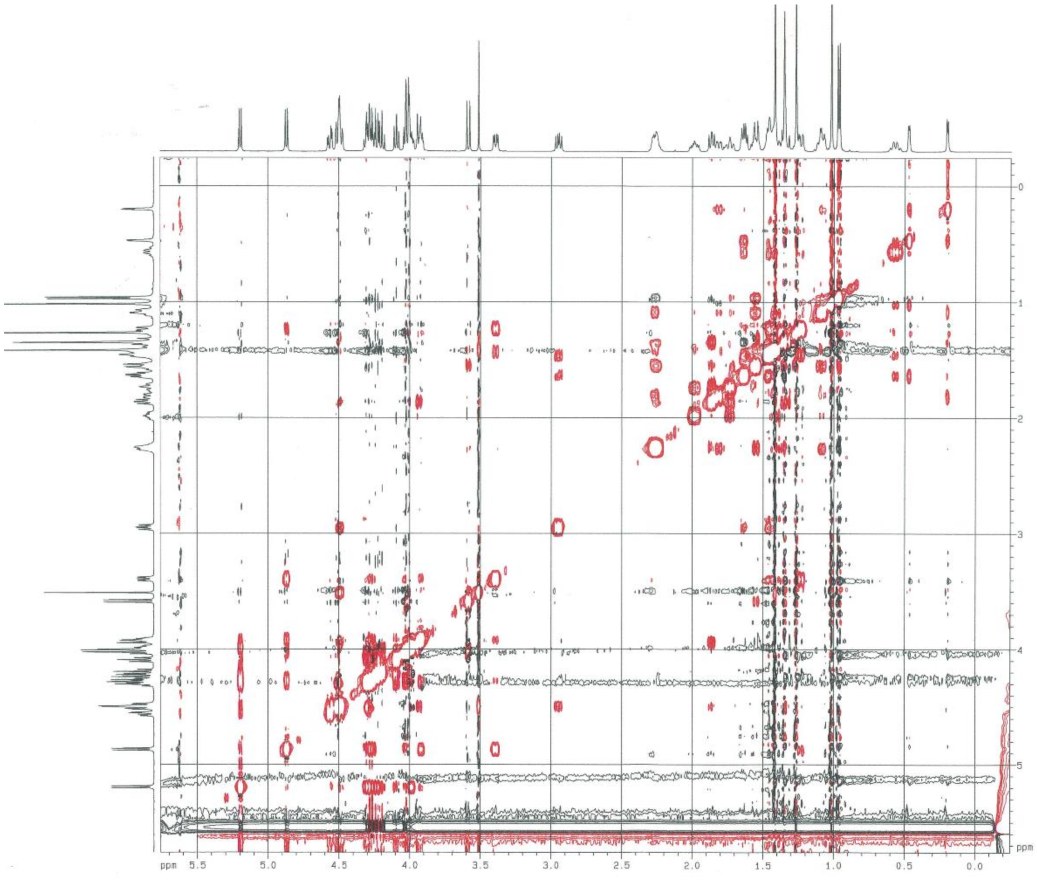

^{1}H-NMR spectrum of **3** in C_5D_5N

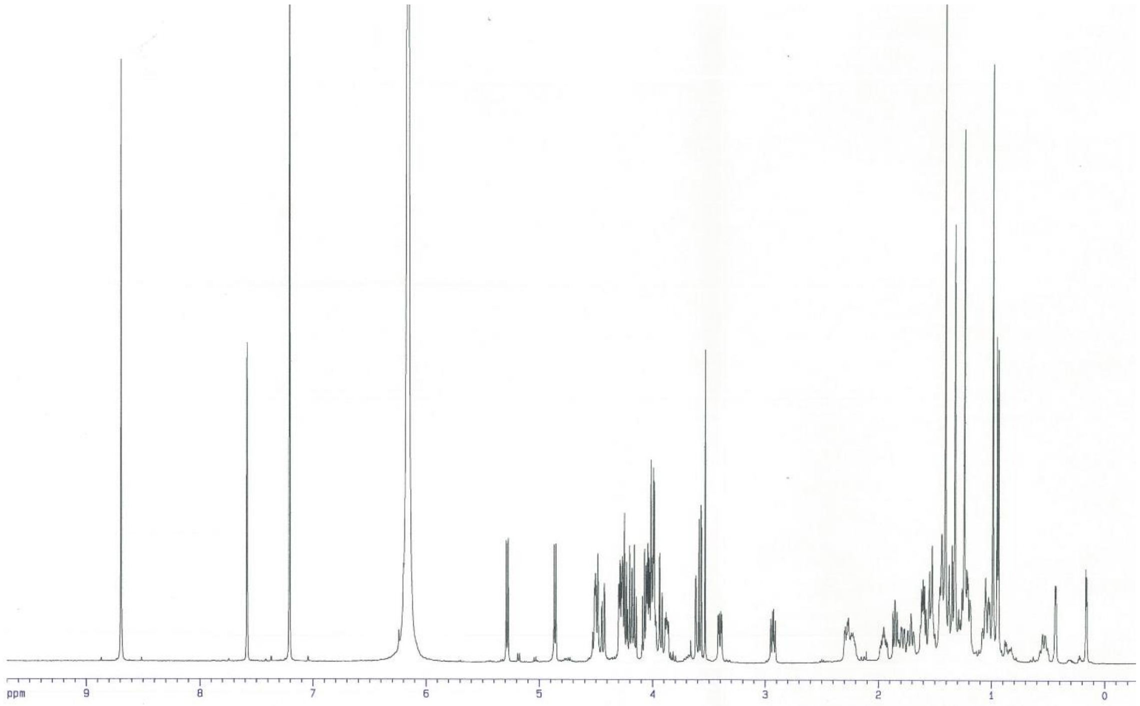

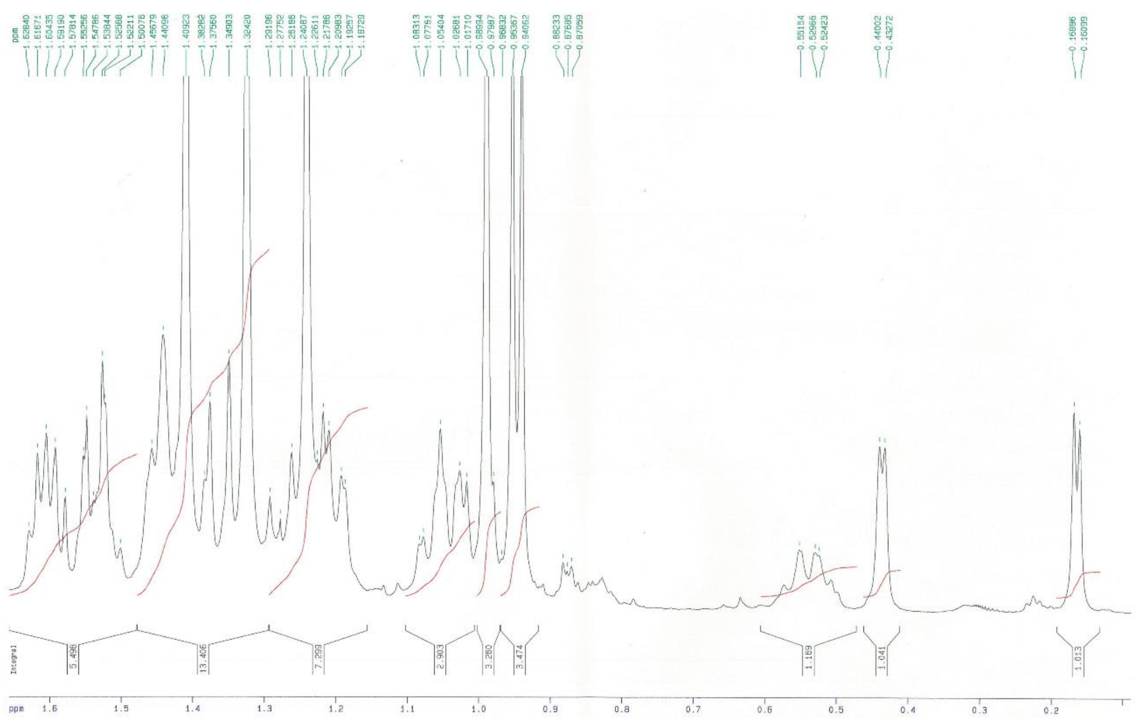

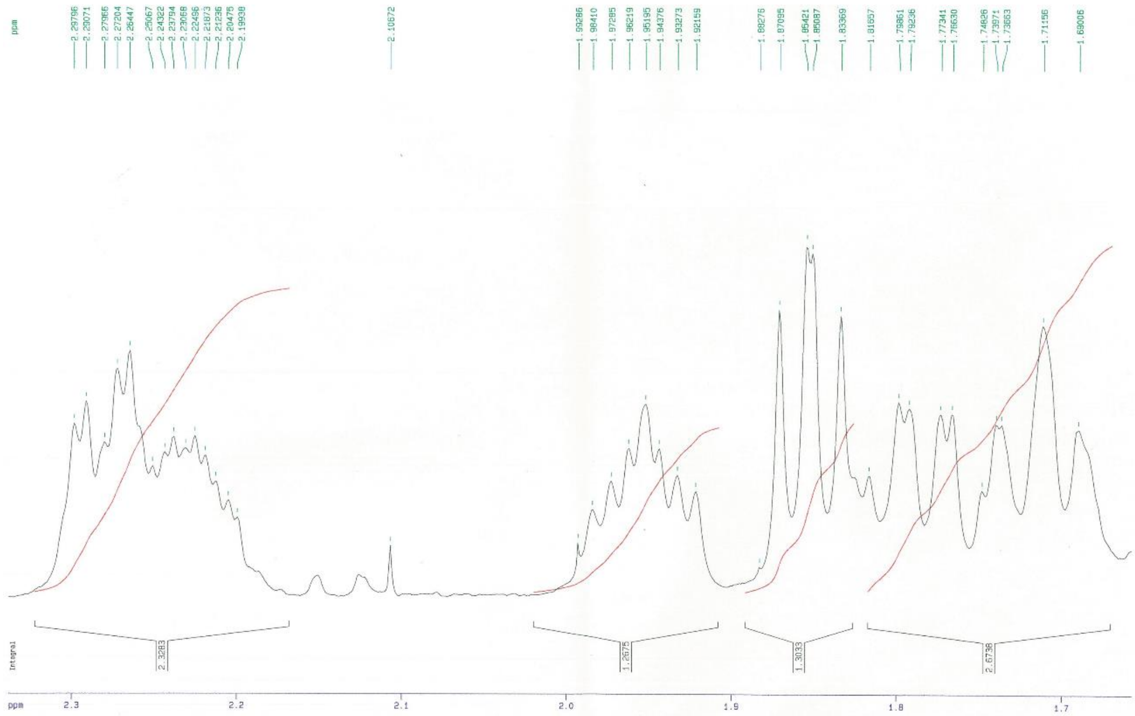

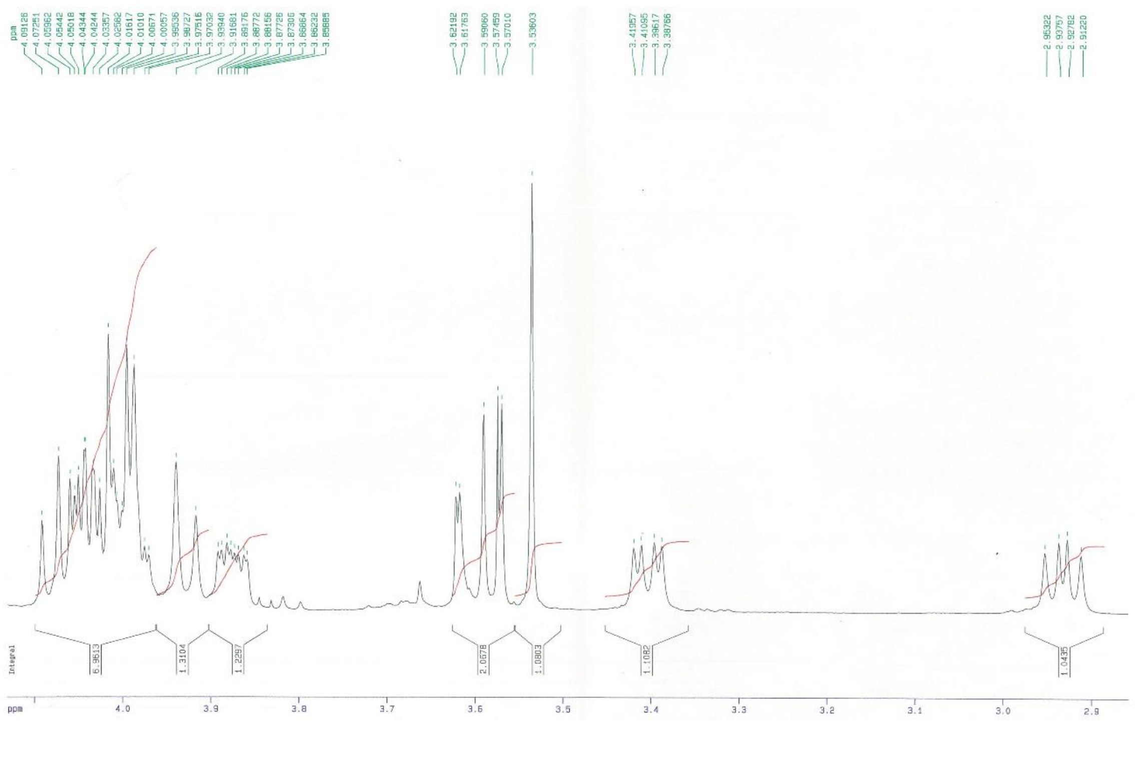
ppm
4.0
3.9
3.8
3.7
3.6
3.5
3.4
3.3
3.2
3.1
3.0
2.9
Integral
6.9613
1.3104
1.2297
2.0578
1.0803
1.1082
1.0435

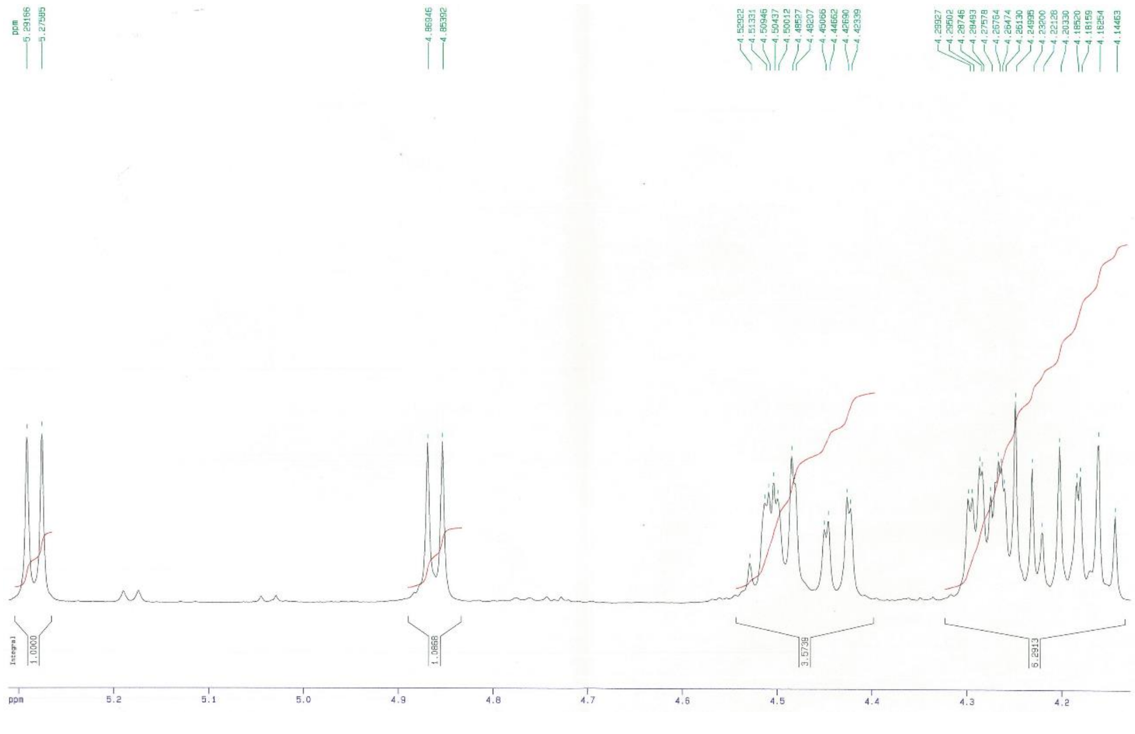
ppm
5.2
5.1
5.0
4.9
4.8
4.7
4.6
4.5
4.4
4.3
4.2
Integral
1.0000
1.0868
3.5736
6.2913

13C-NMR spectrum of **3** in C_5D_5N

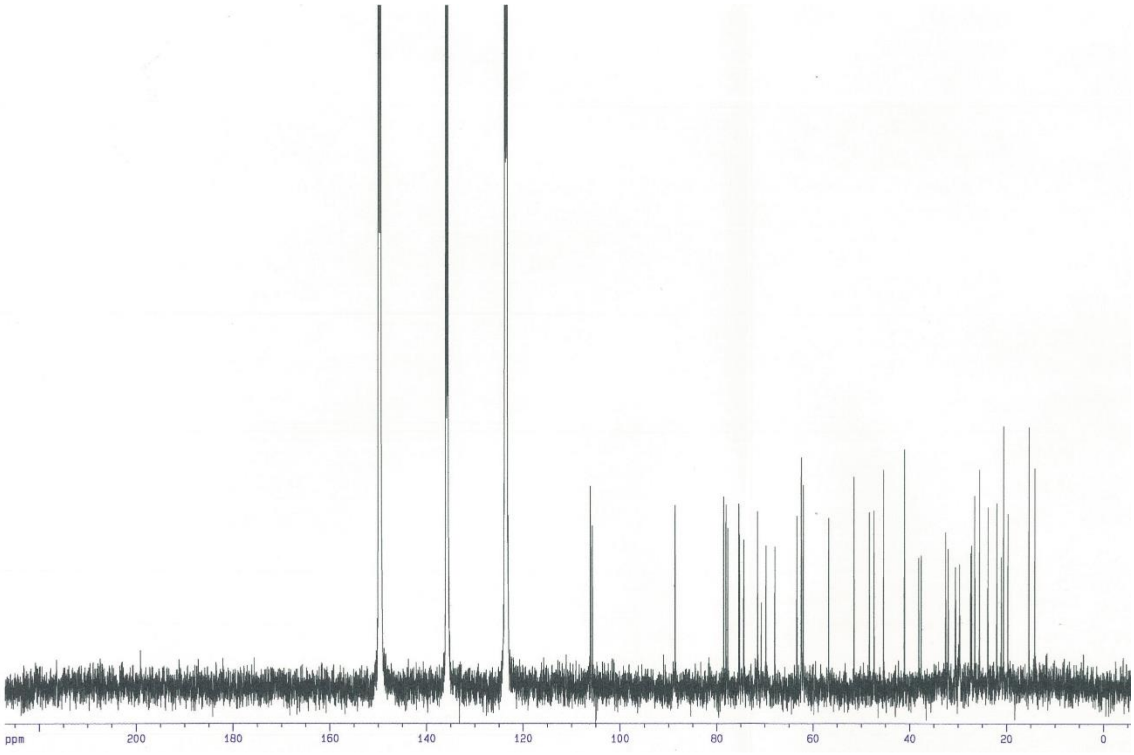

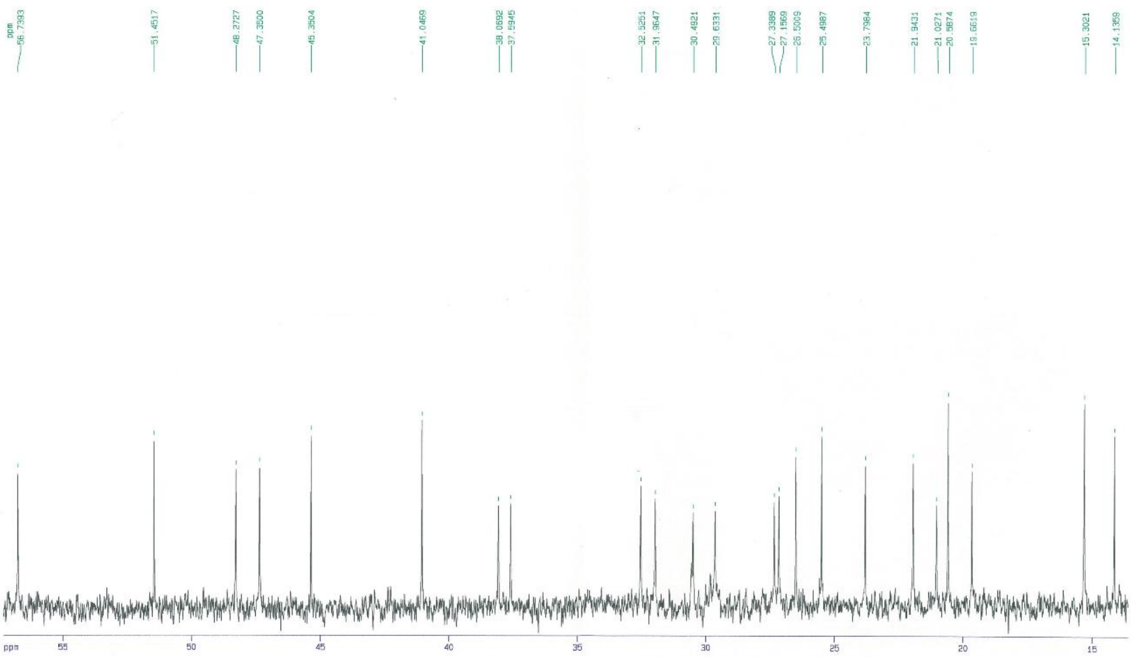

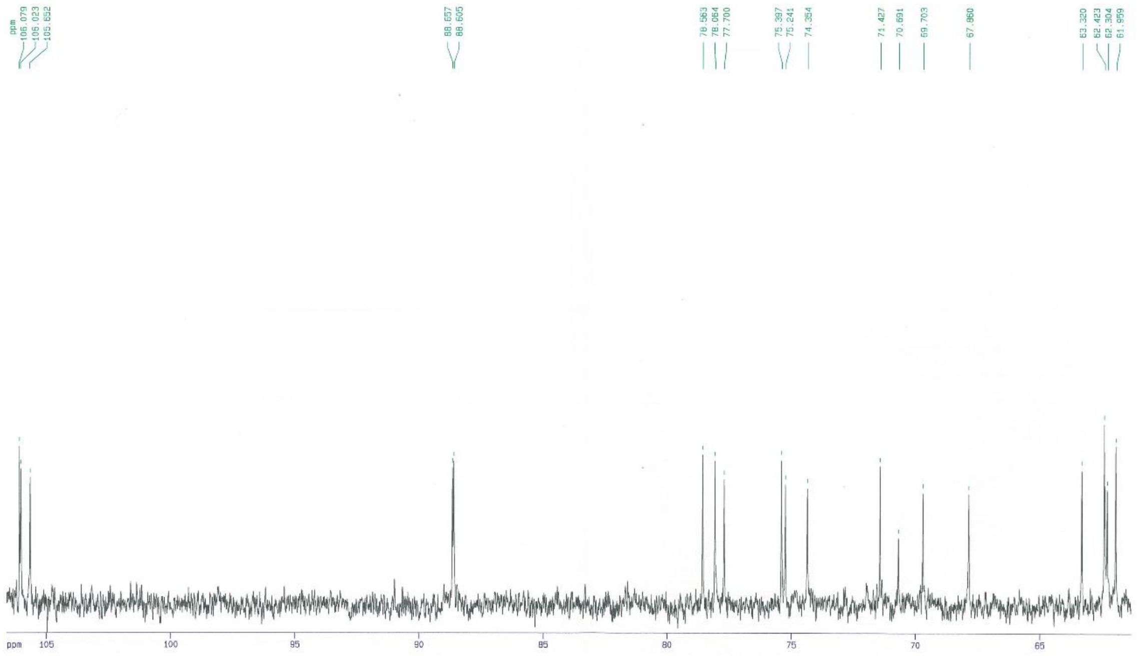

COSY spectrum of **3** in C_5D_5N

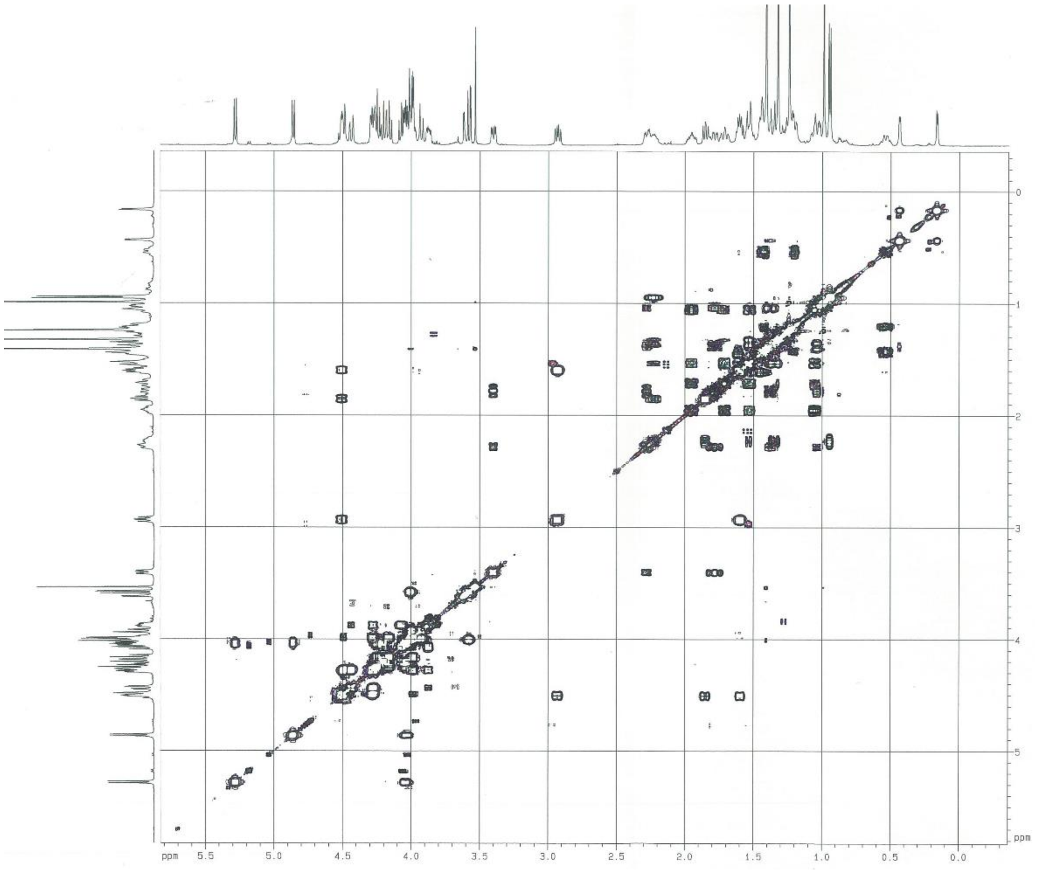

HMQC spectrum of **3** in C_5D_5N

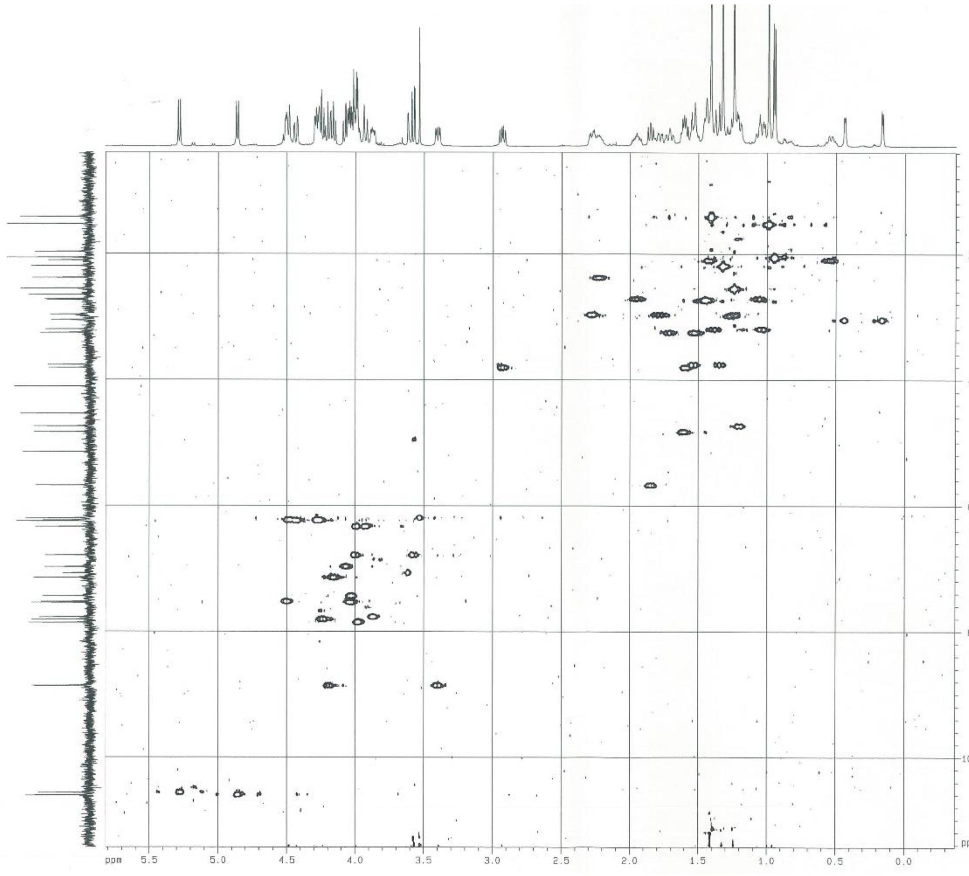

HMBC spectrum of **3** in C_5D_5N

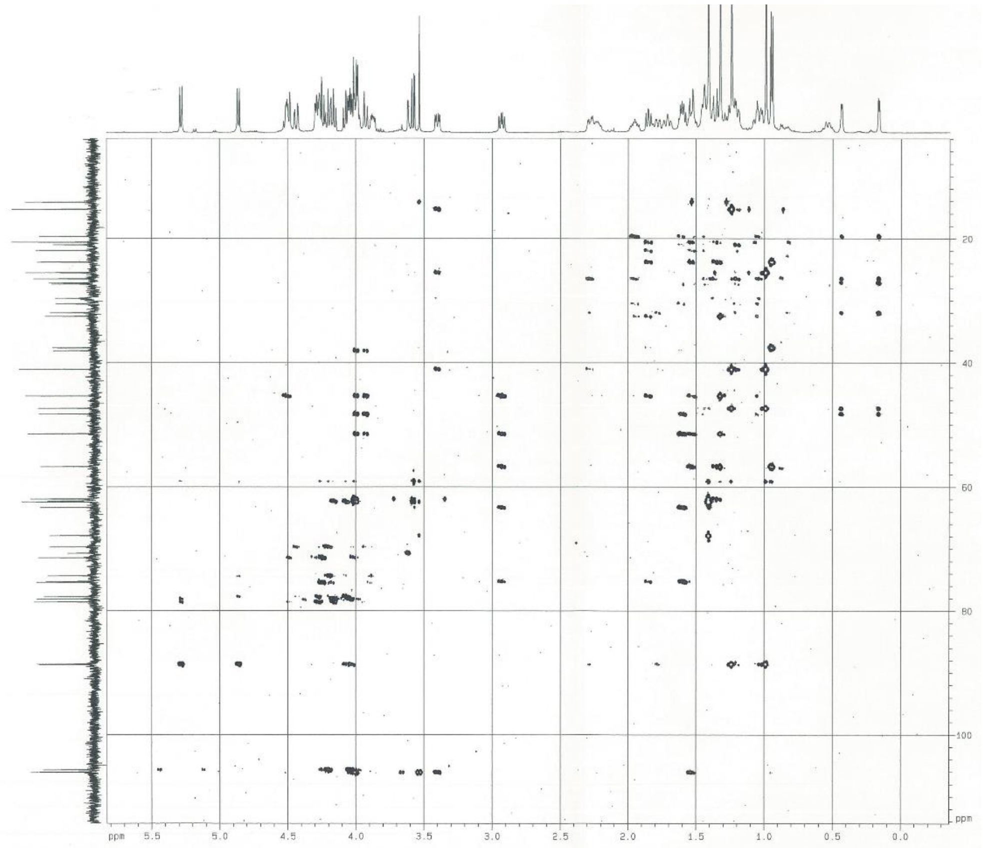

NOESY spectrum of **3** in C_5D_5N

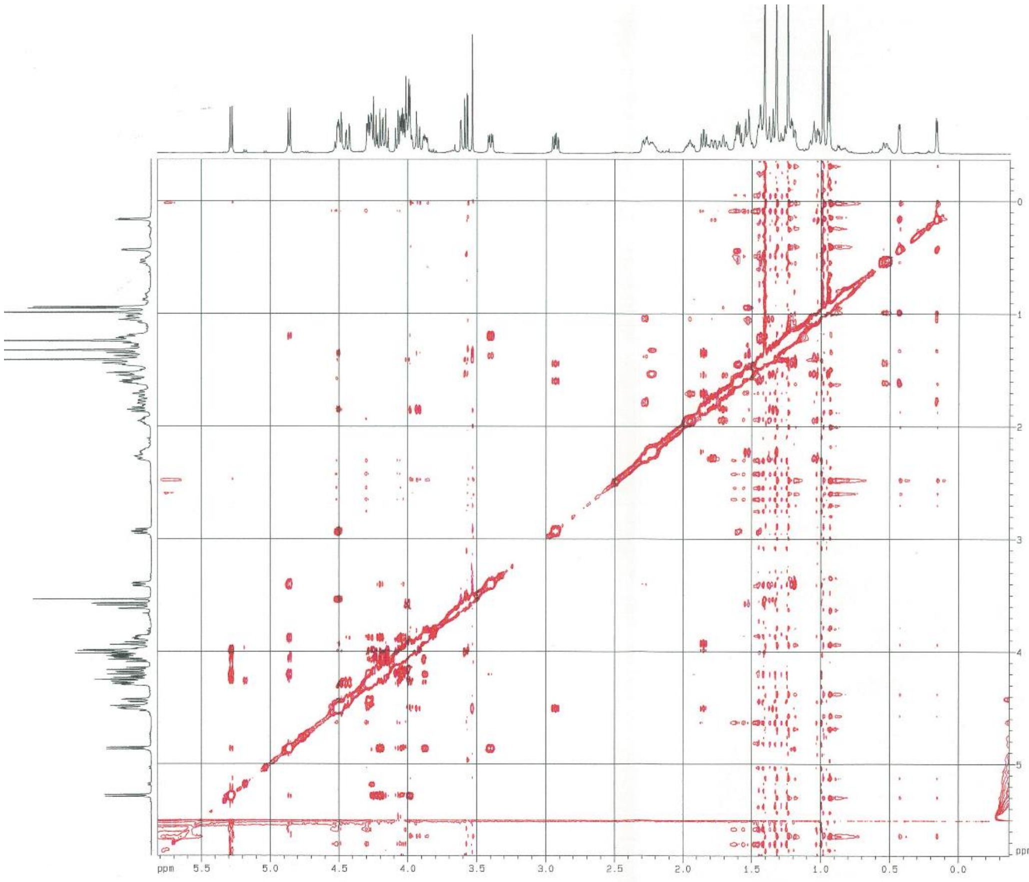

^{1}H-NMR spectrum of **4** in C_5D_5N

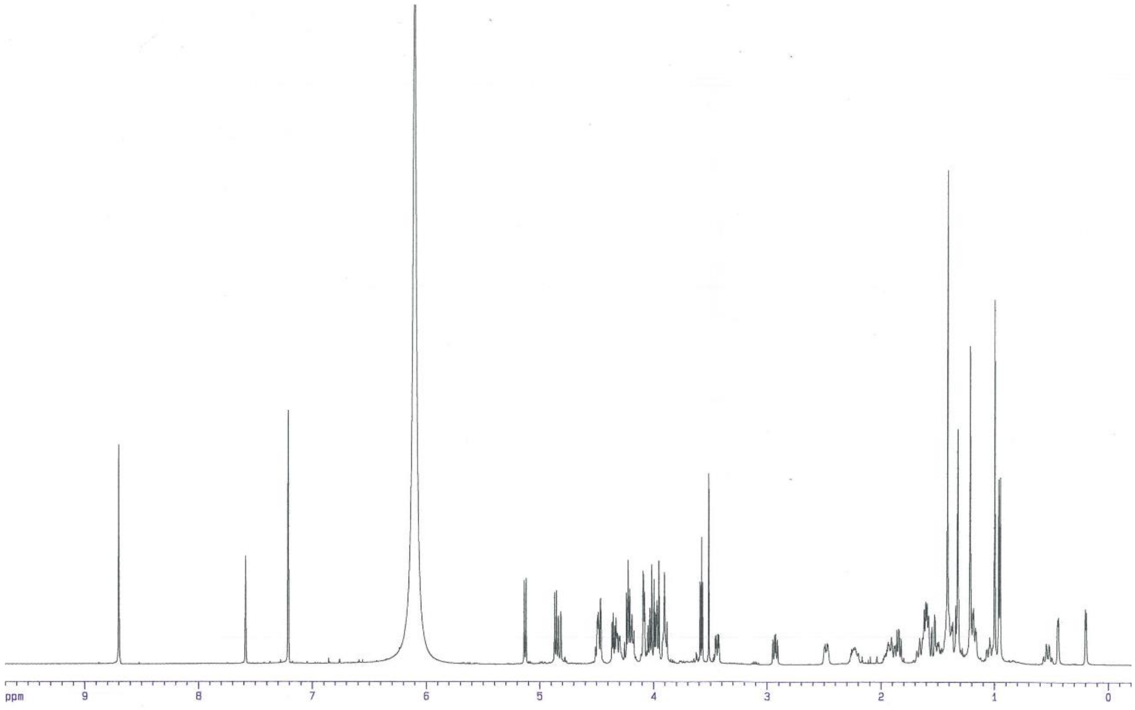

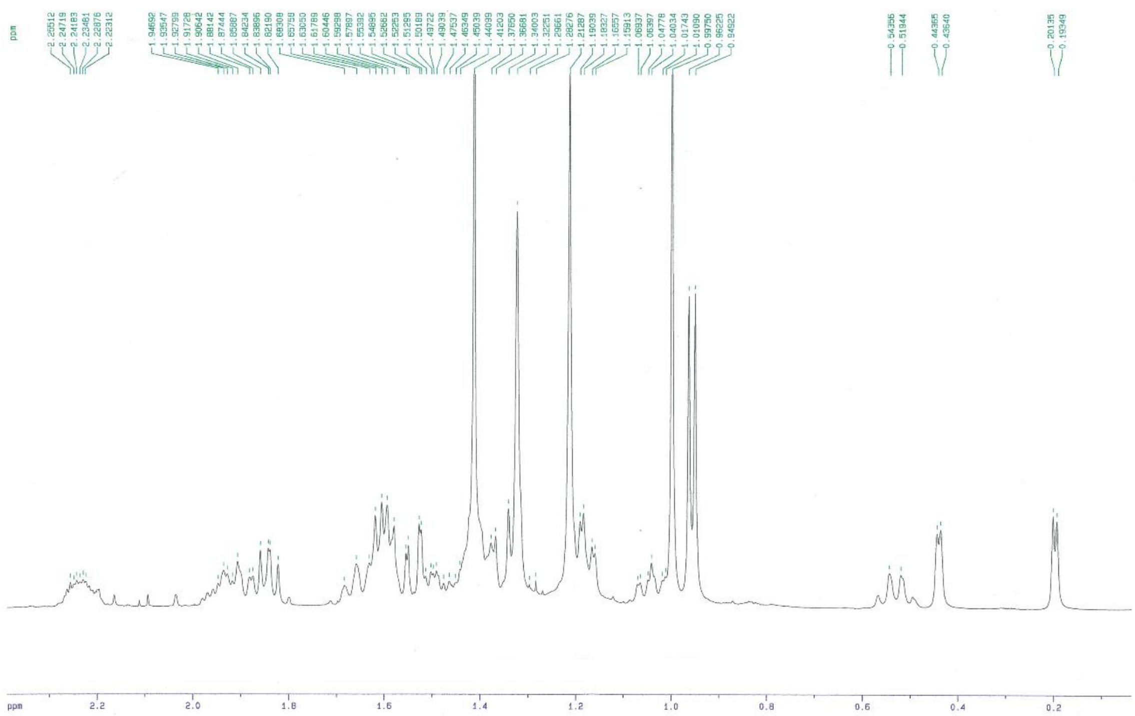

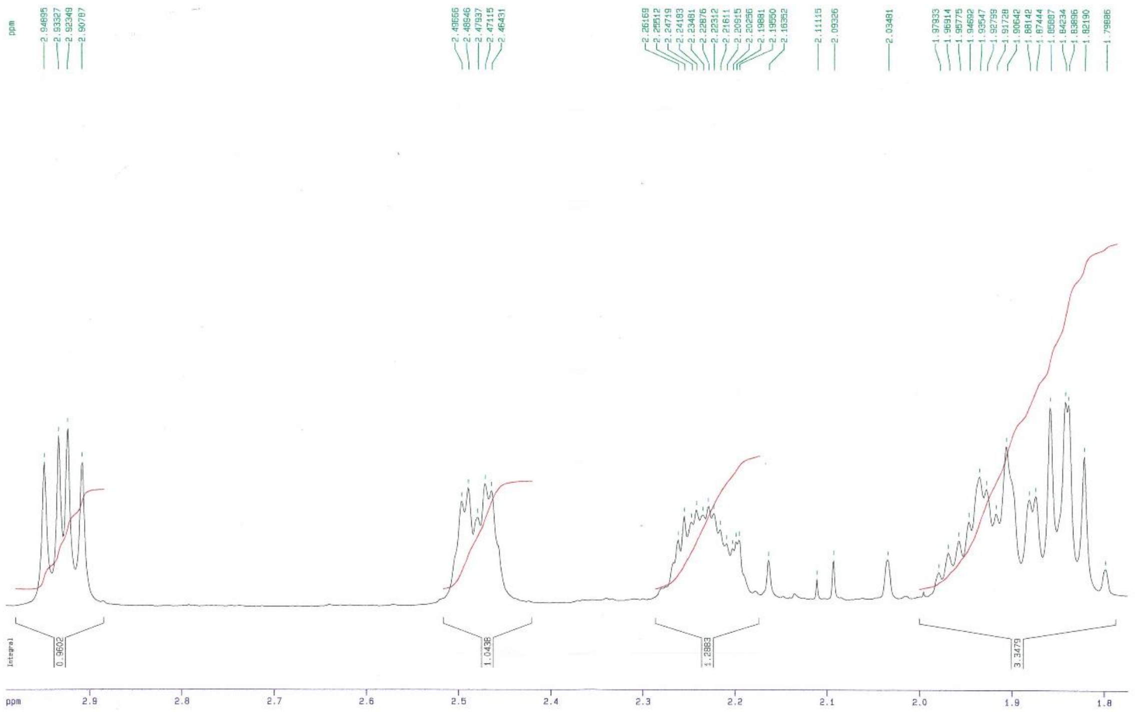

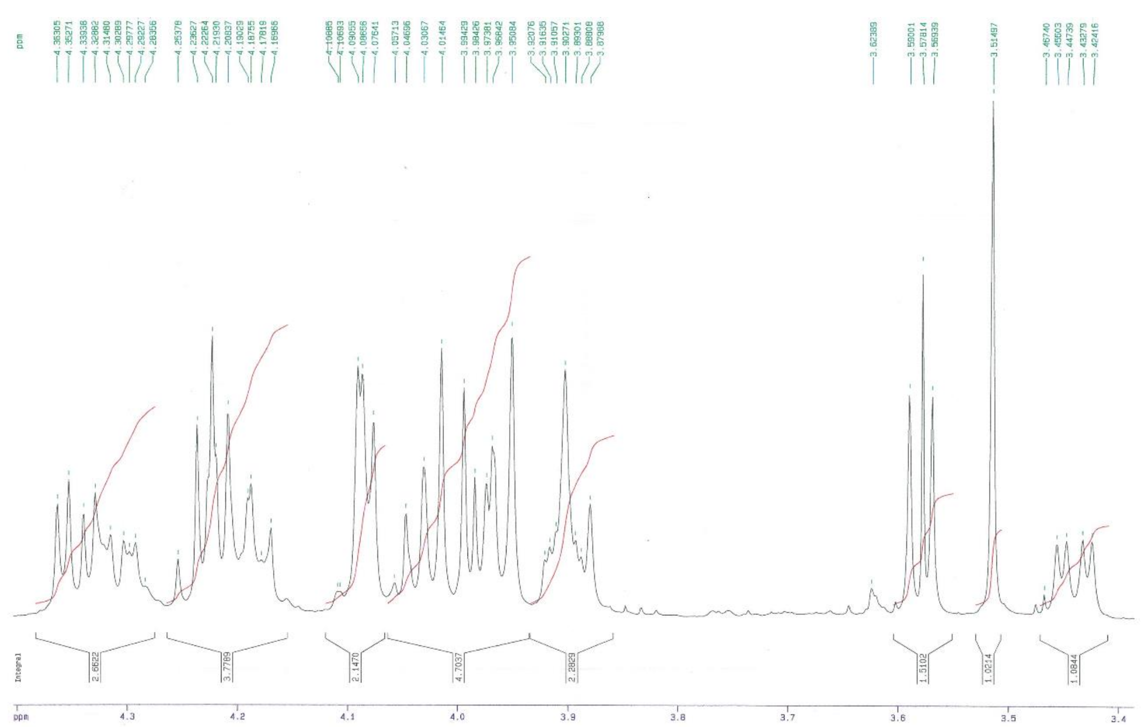

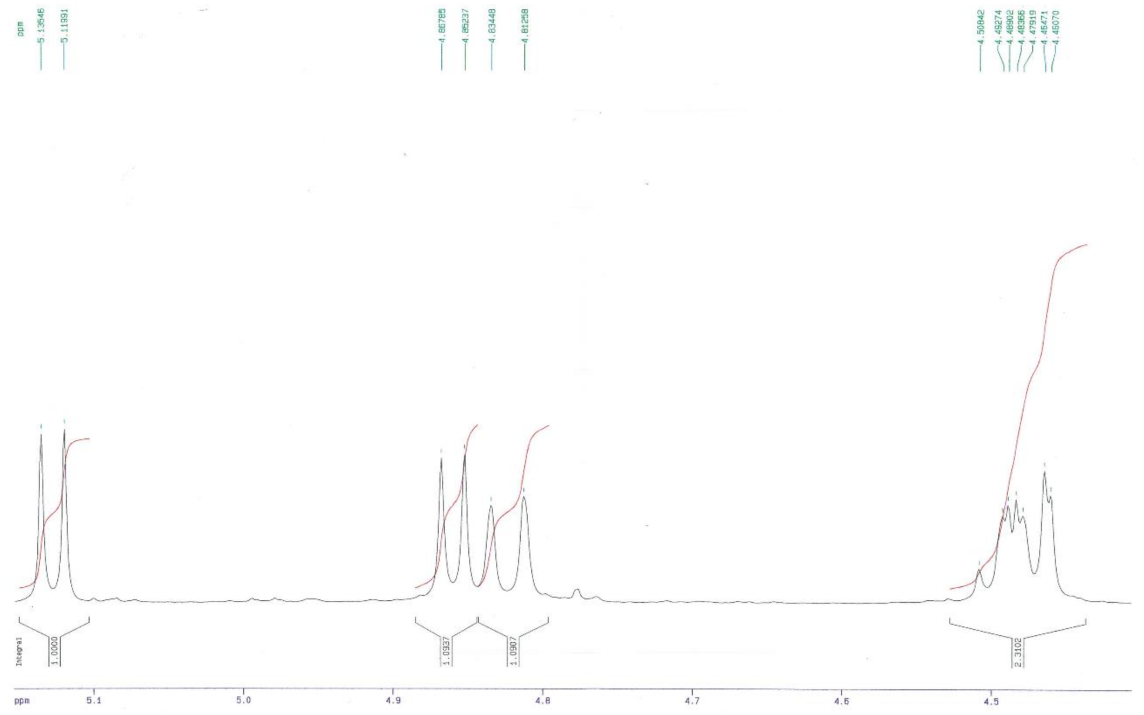

^{13}C-NMR spectrum of **4** in C_5D_5N

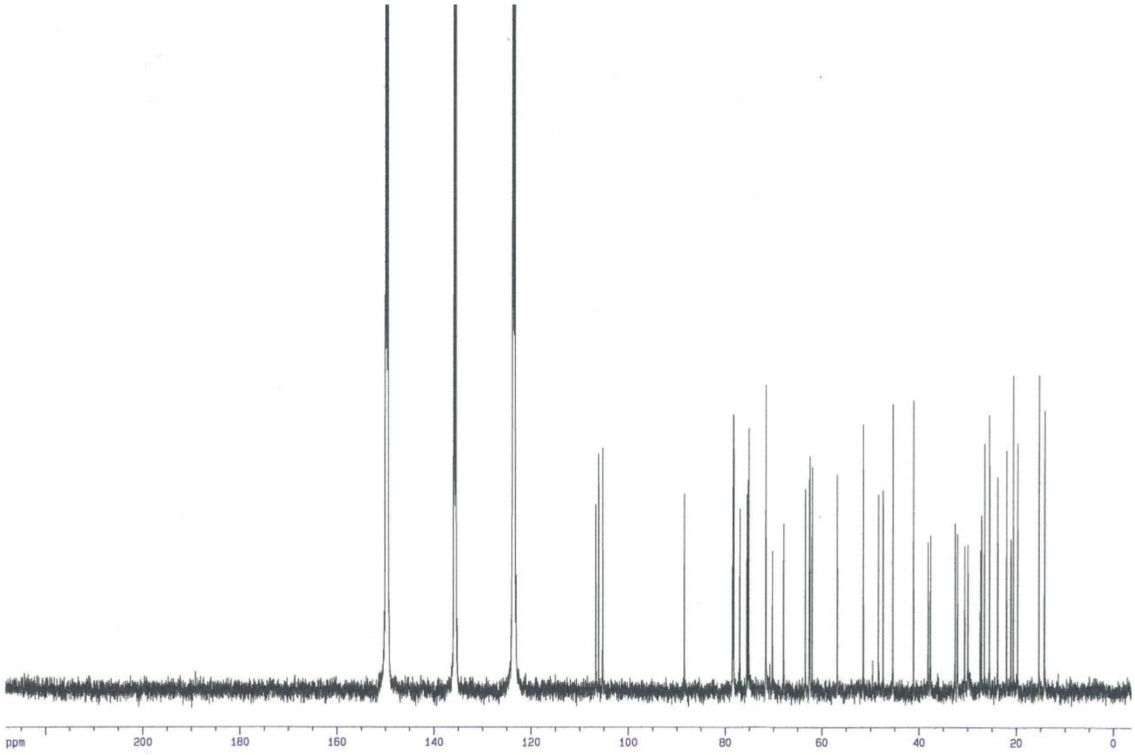

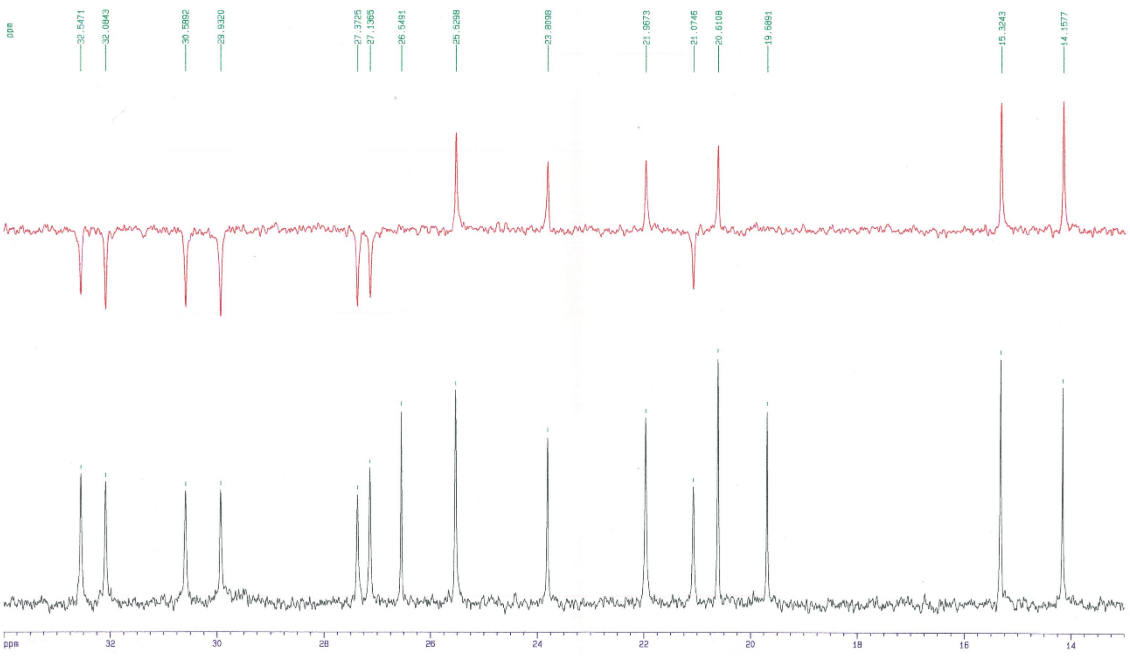

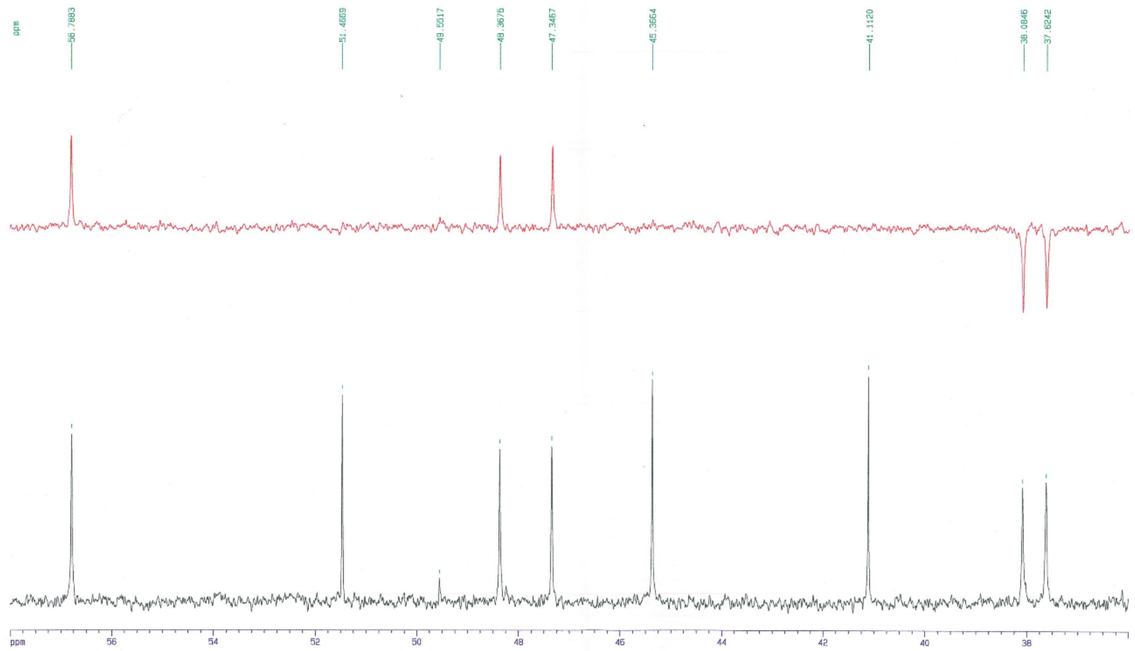

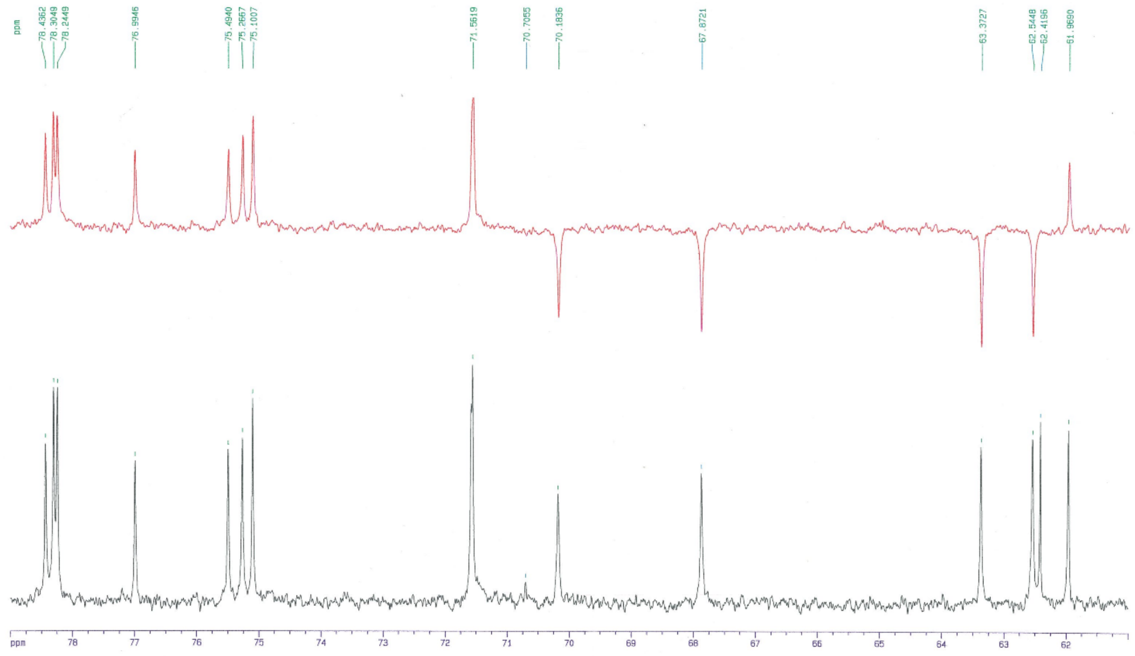

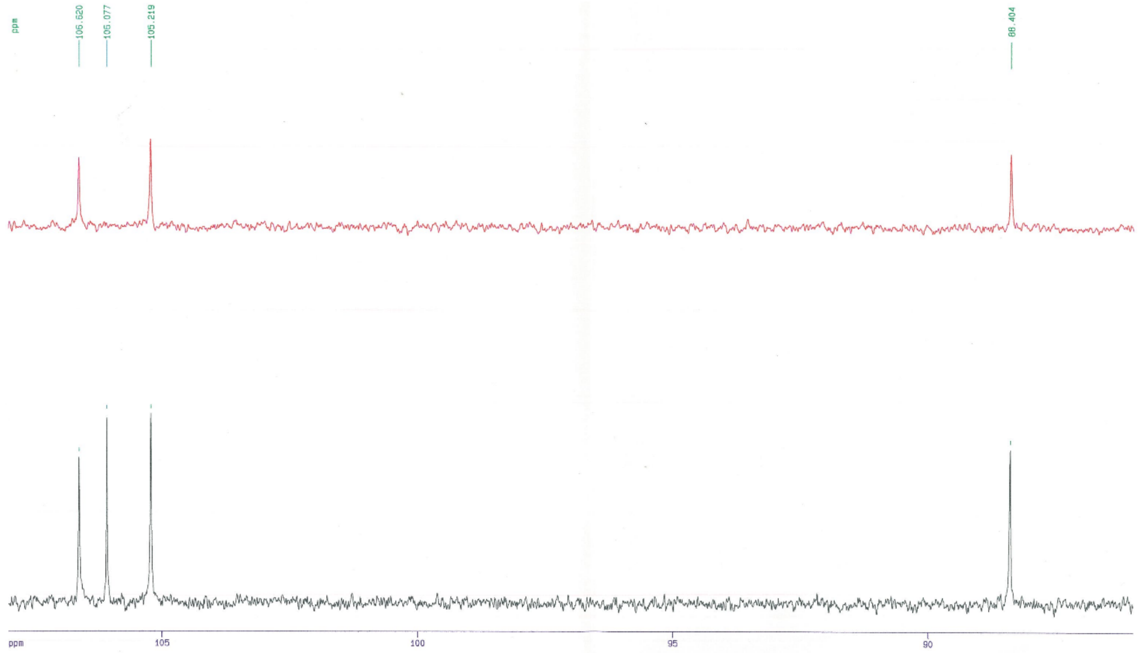
ppm
106.620
106.077
105.219
88.404
ppm
105
100
95
90

COSY spectrum of **4** in C_5D_5N

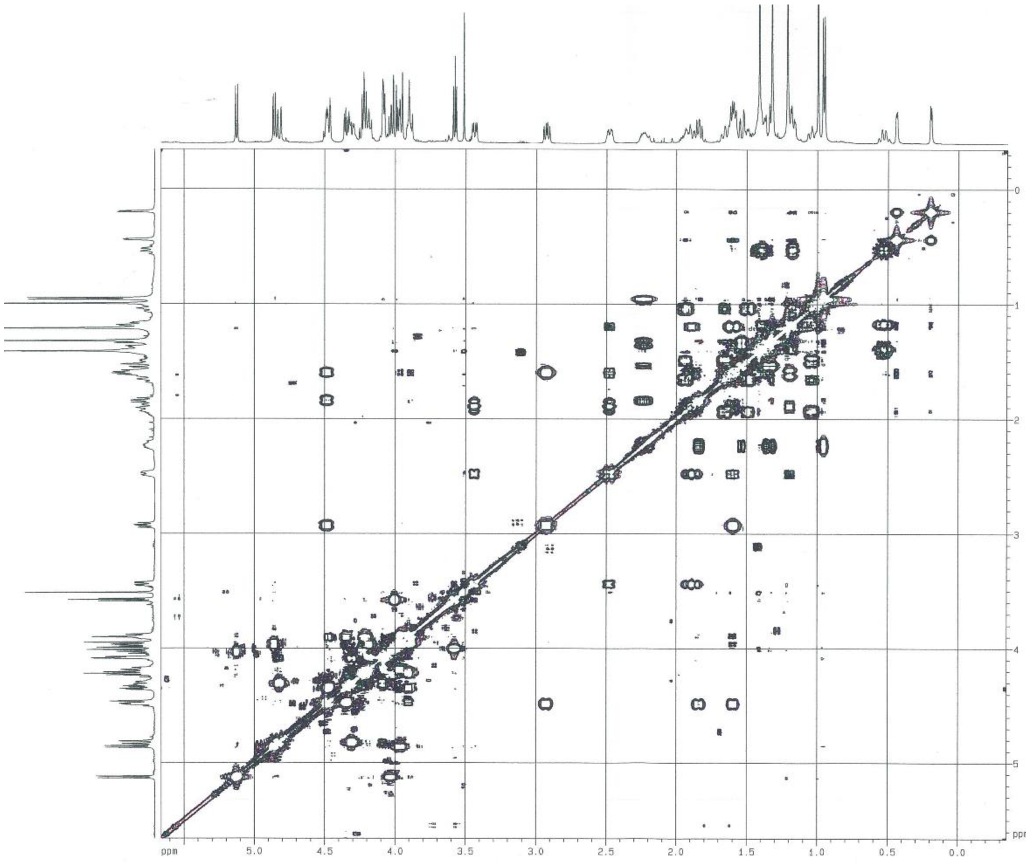

HMQC spectrum of **4** in C_5D_5N

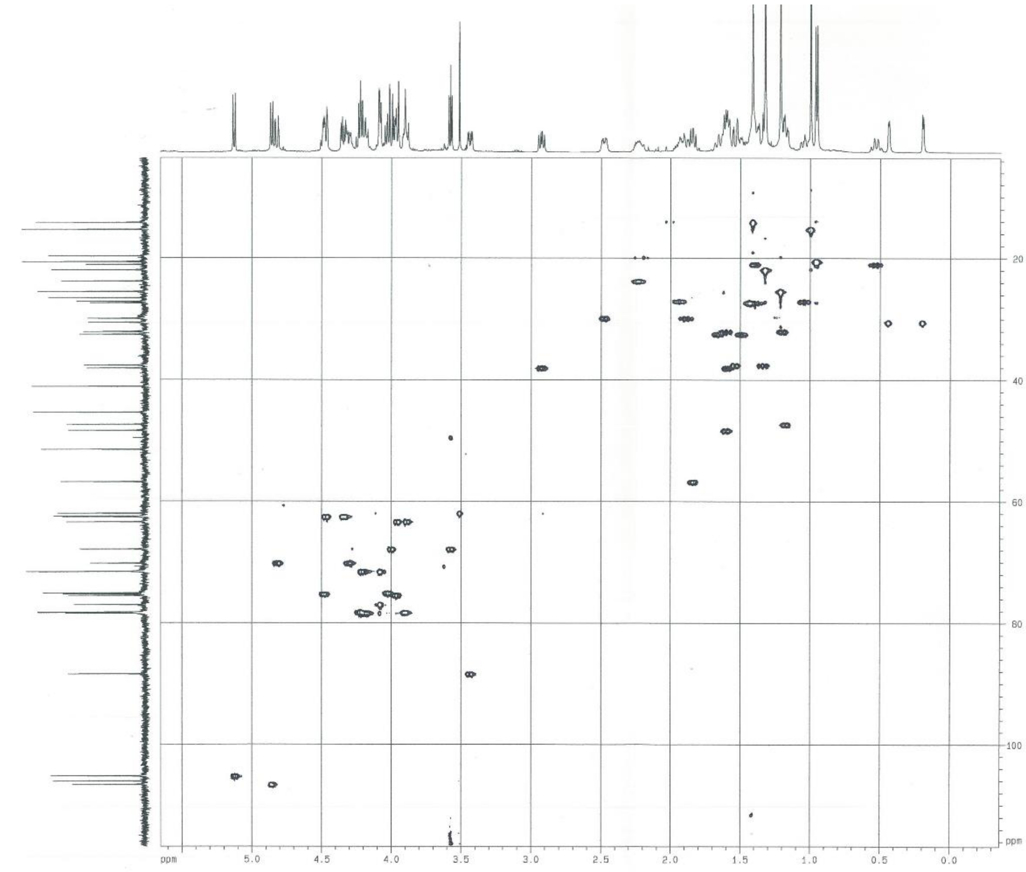

HMBC spectrum of **4** in C_5D_5N

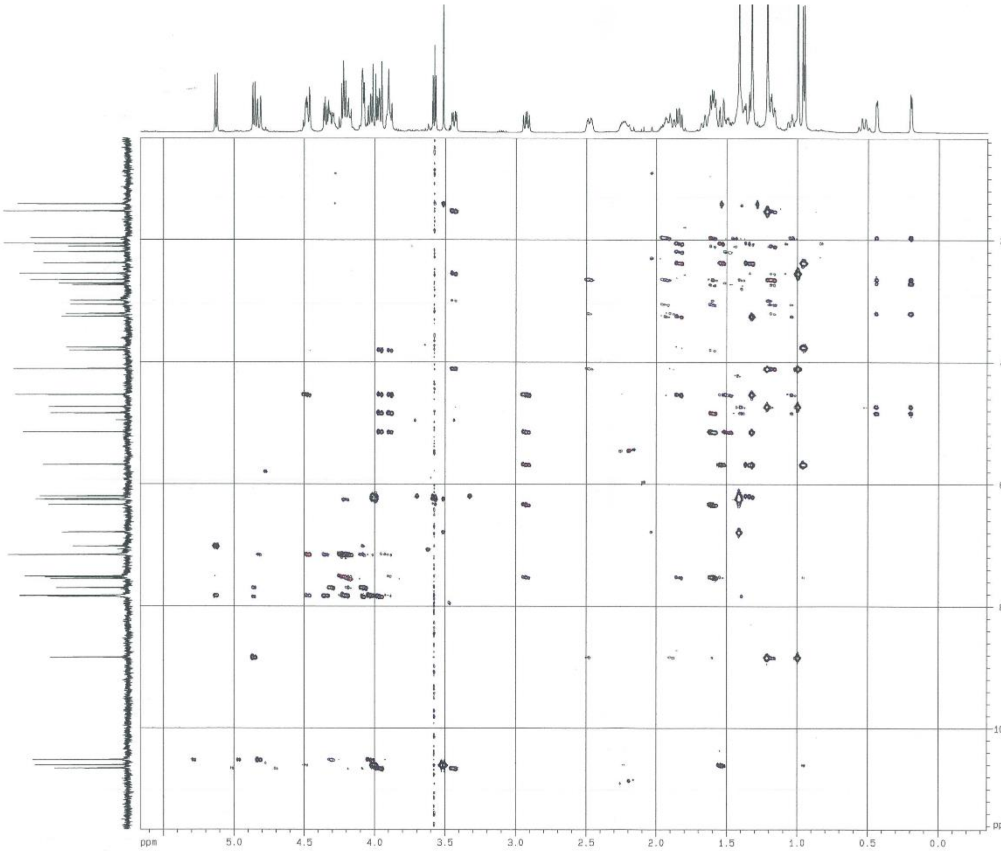

^{1}H-NMR spectrum of **5** in C_5D_5N

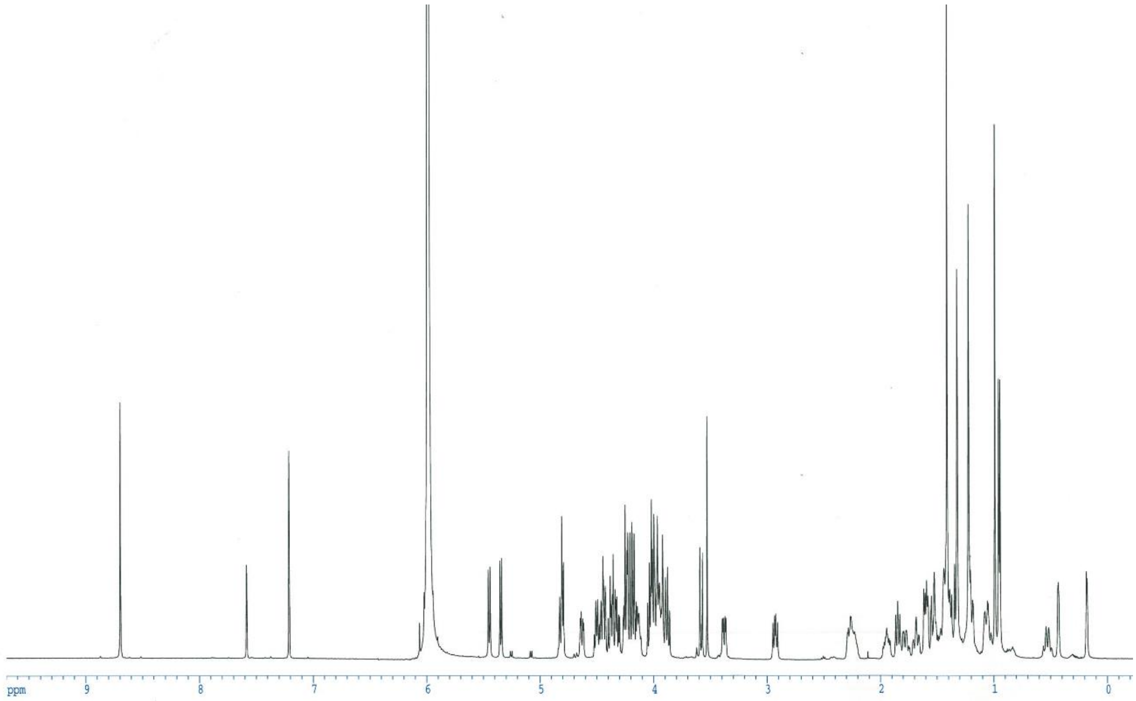

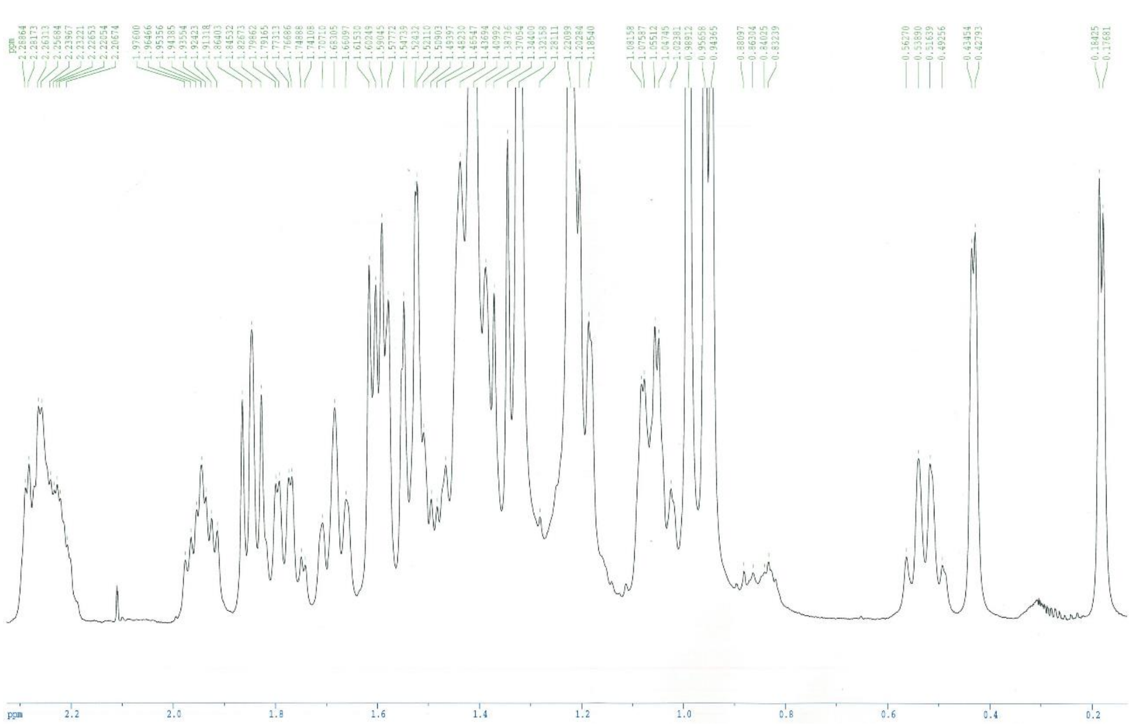

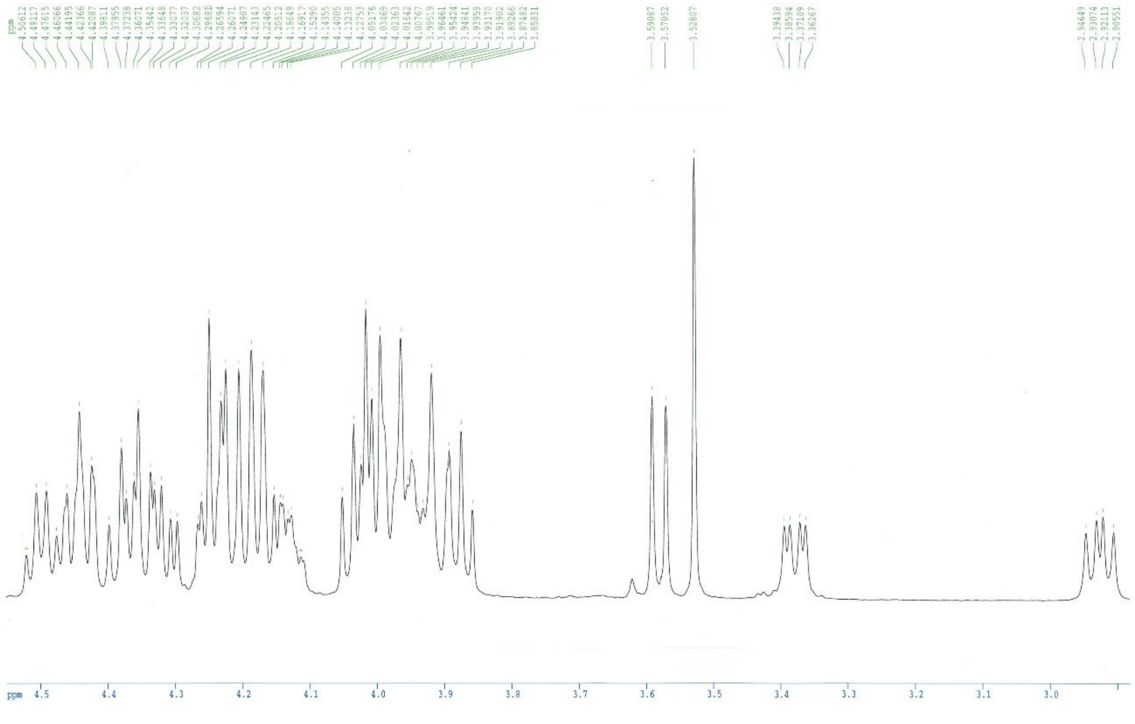

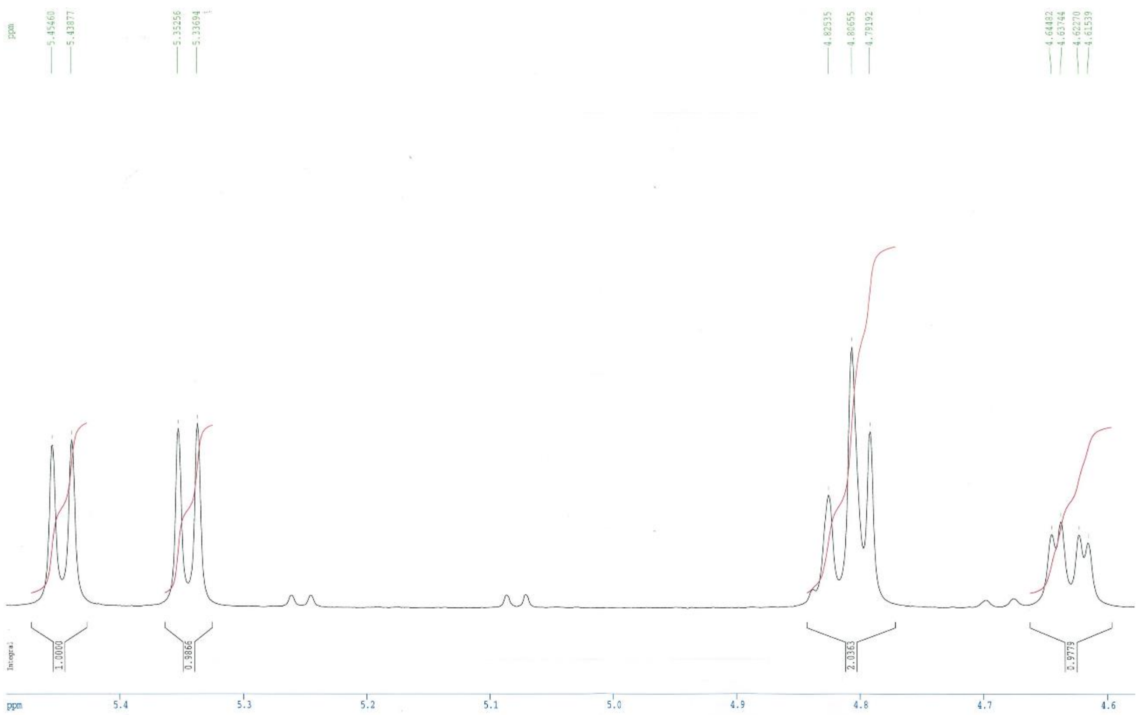

^{13}C-NMR spectrum of **5** in C_5D_5N

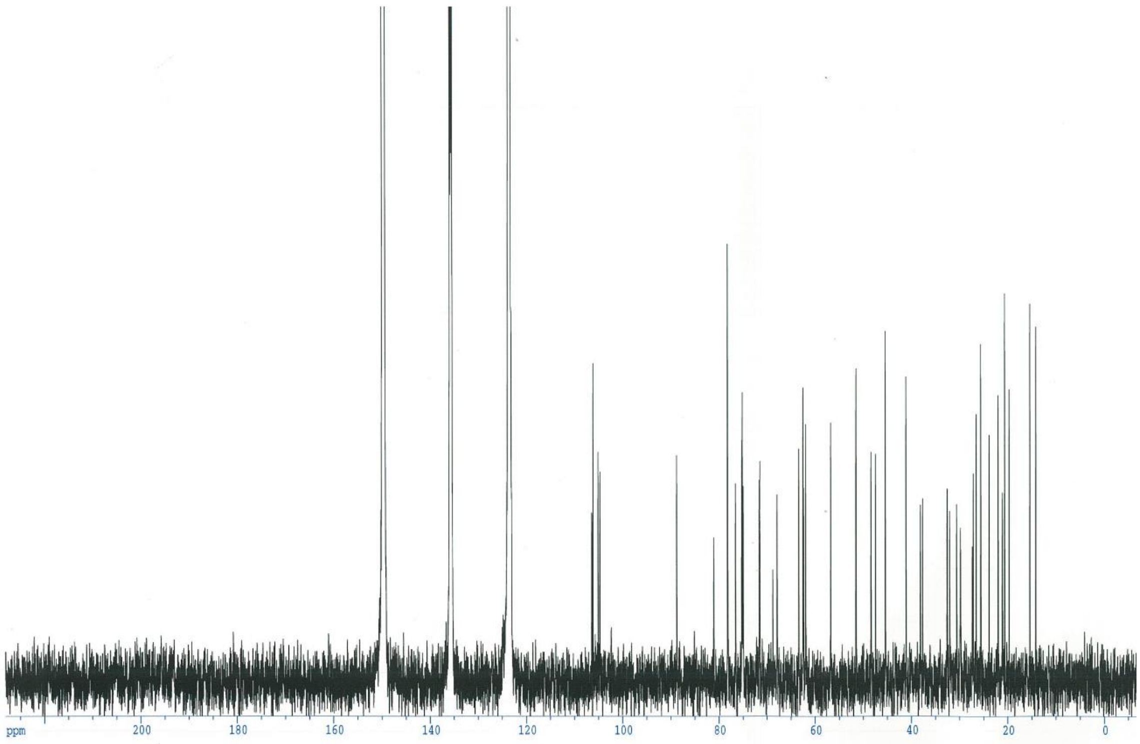

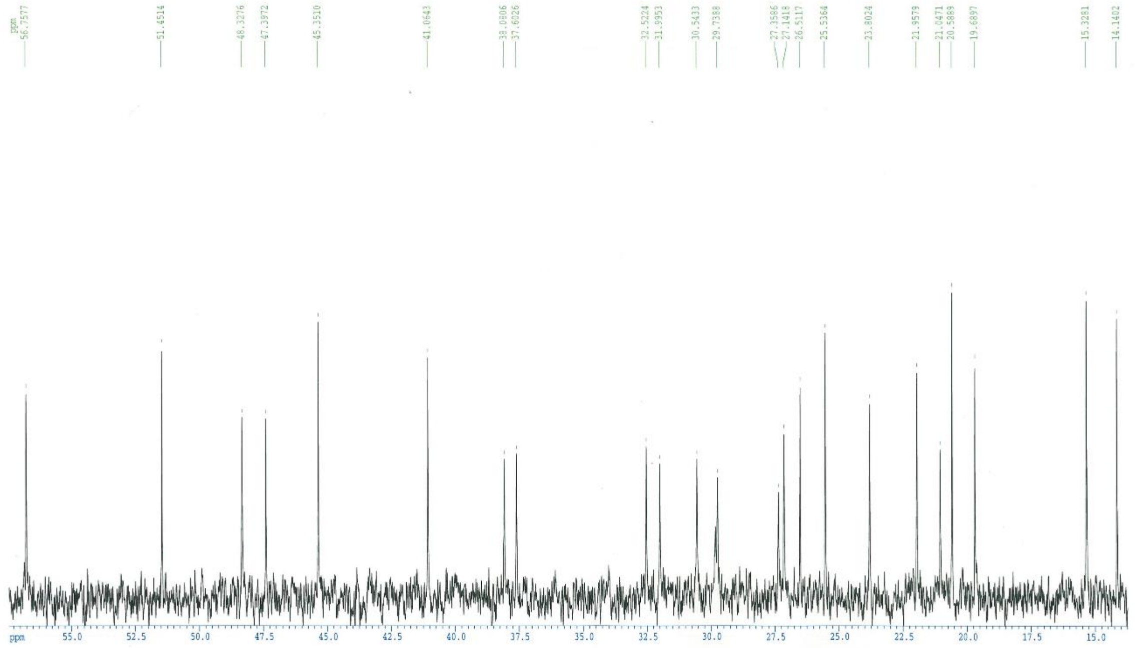
ppm
56.7577
51.4514
48.3276
47.3972
45.3510
41.0643
38.0806
37.6026
32.5224
31.9953
30.5433
29.7388
27.3586
27.1418
26.5117
25.5364
23.8024
21.9579
21.0471
20.5889
19.6897
15.3281
14.1482
ppm 55.0 52.5 50.0 47.5 45.0 42.5 40.0 37.5 35.0 32.5 30.0 27.5 25.0 22.5 20.0 17.5 15.0

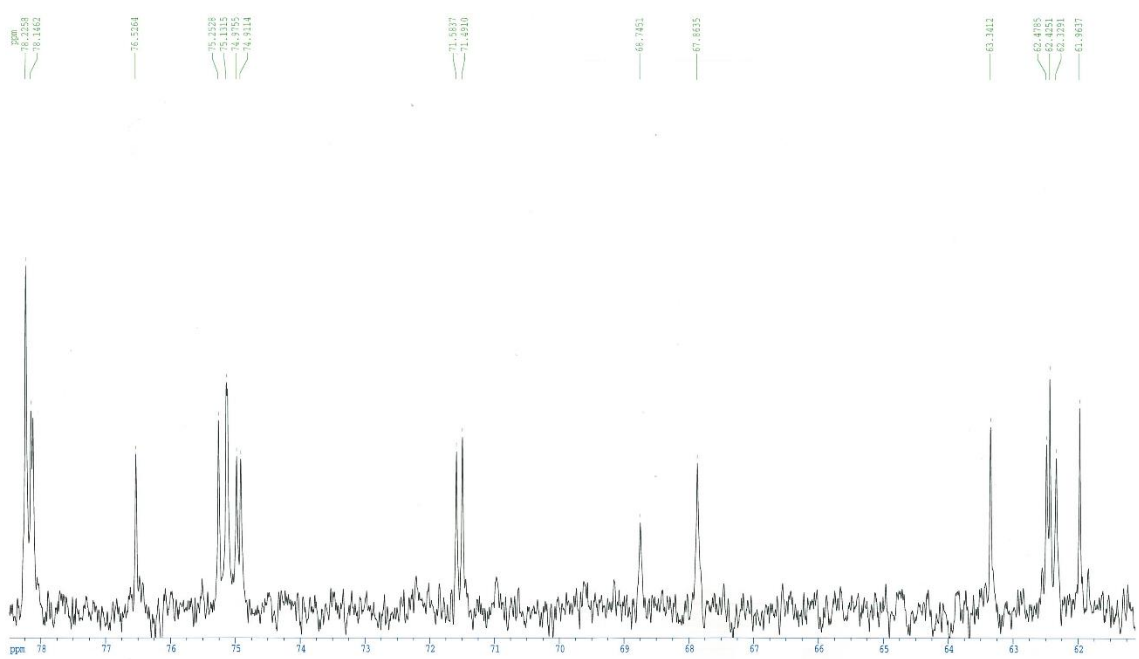
ppm
78.2258
78.1462
76.5264
75.2528
75.1315
74.9755
74.9114
71.5837
71.4910
68.7451
67.8635
63.3412
62.4785
62.4251
62.3291
61.9637
ppm 78 77 76 75 74 73 72 71 70 69 68 67 66 65 64 63 62

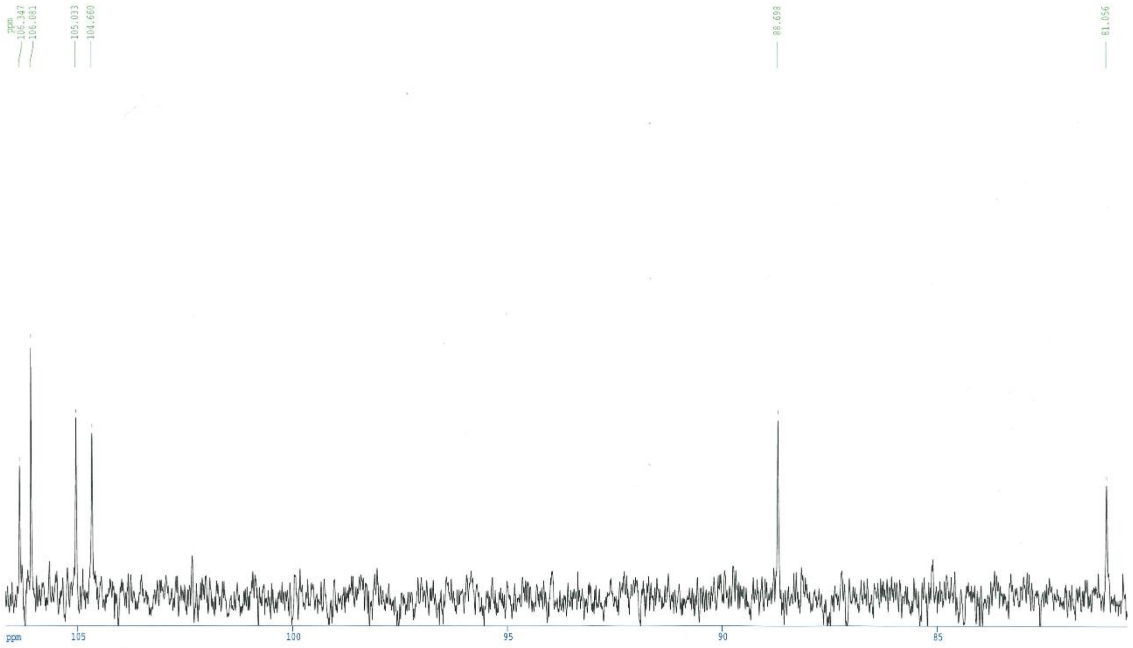

COSY spectrum of **5** in C_5D_5N

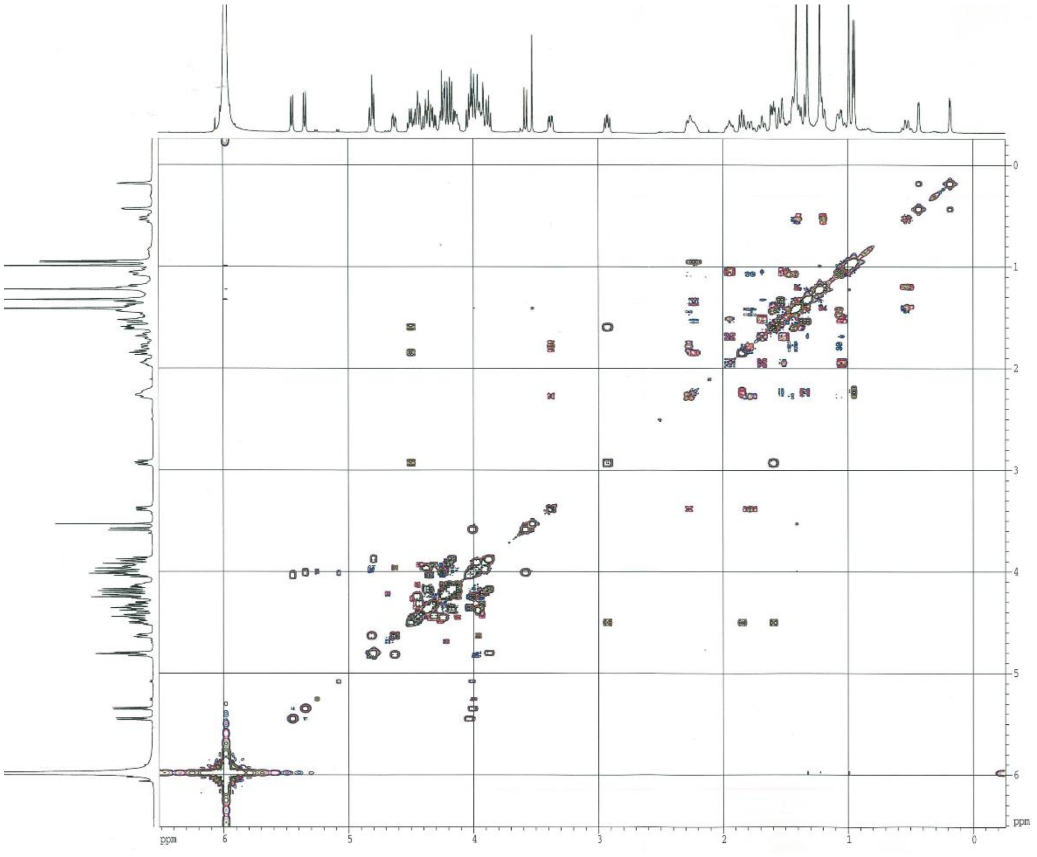

HMQC spectrum of **5** in C_5D_5N

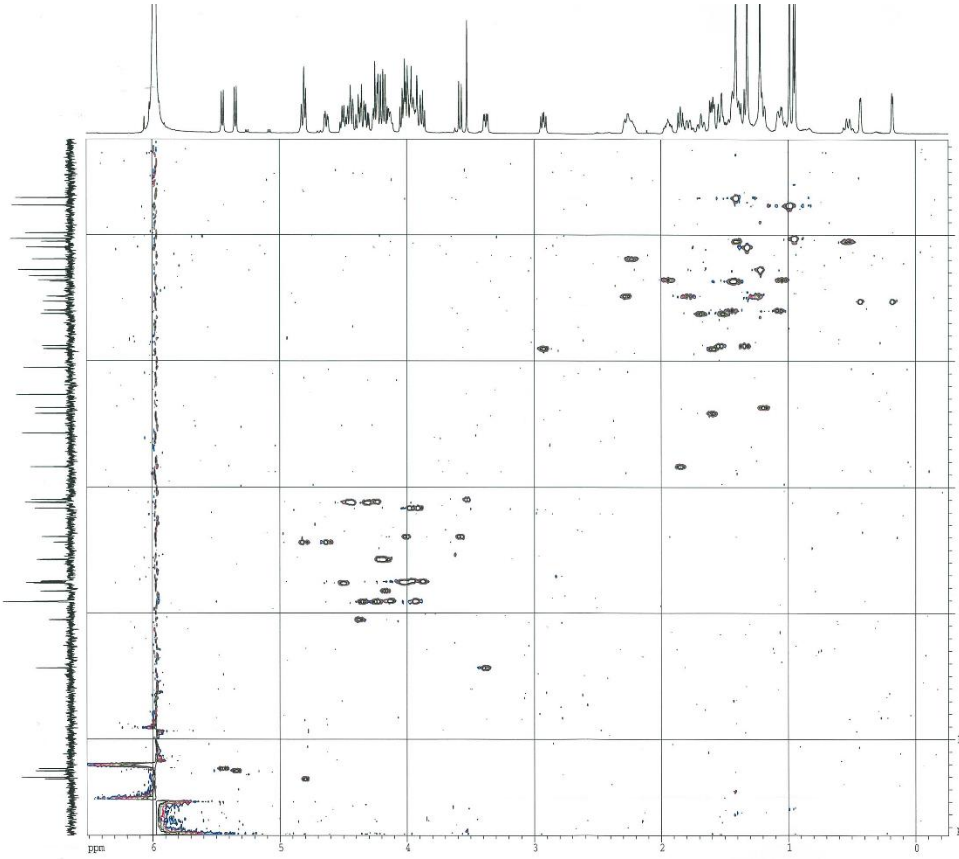

HMBC spectrum of **5** in C_5D_5N

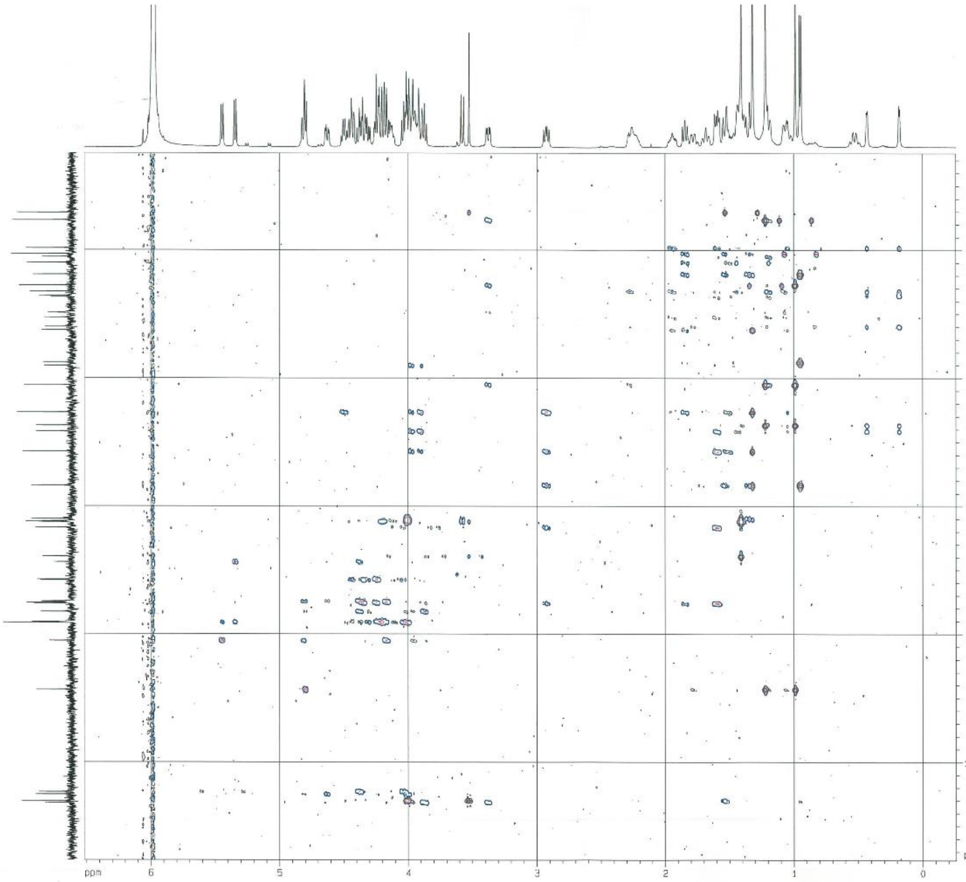

^{1}H-NMR spectrum of **6** in C_5D_5N

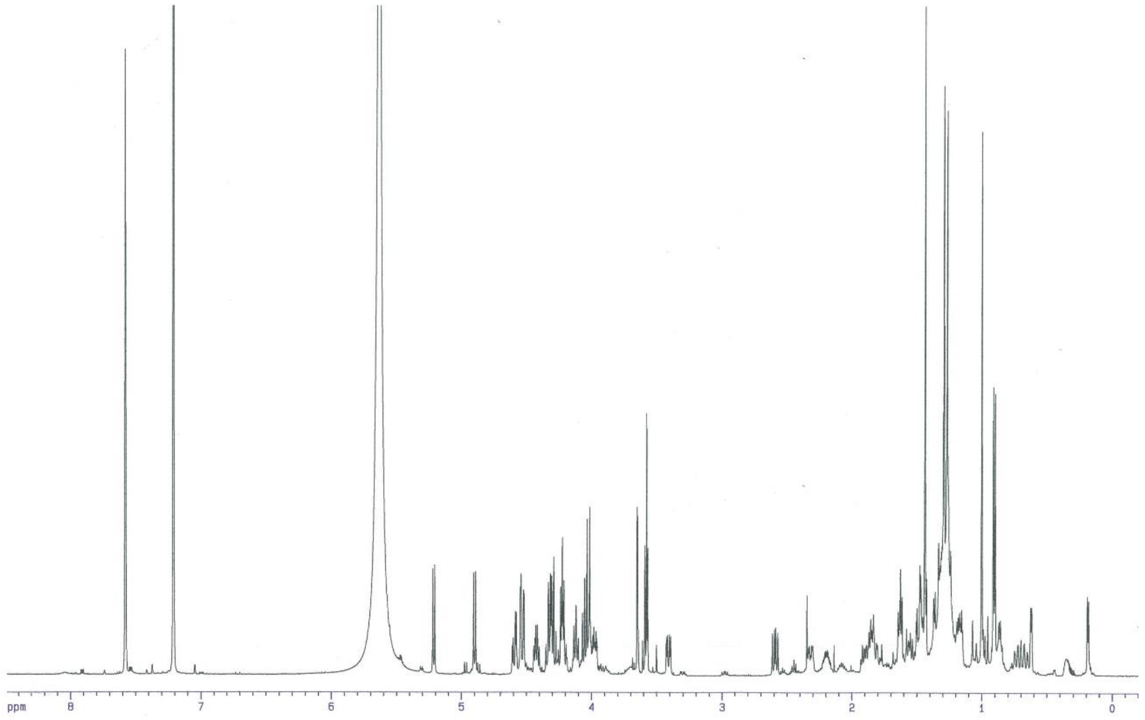

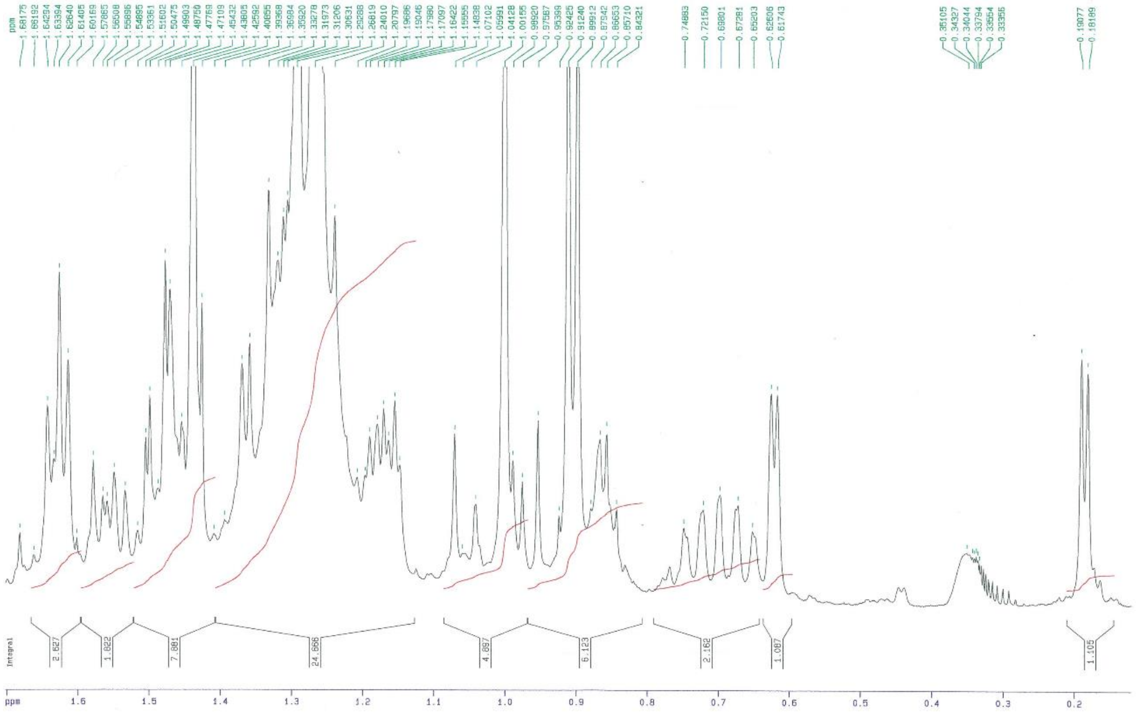

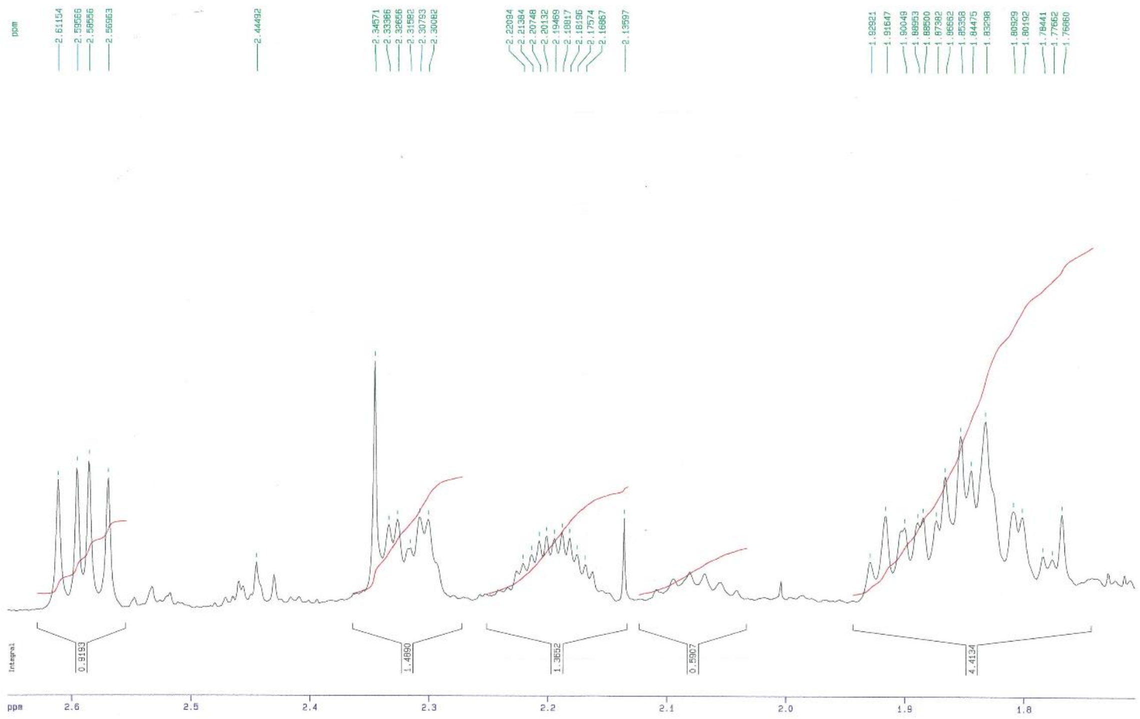

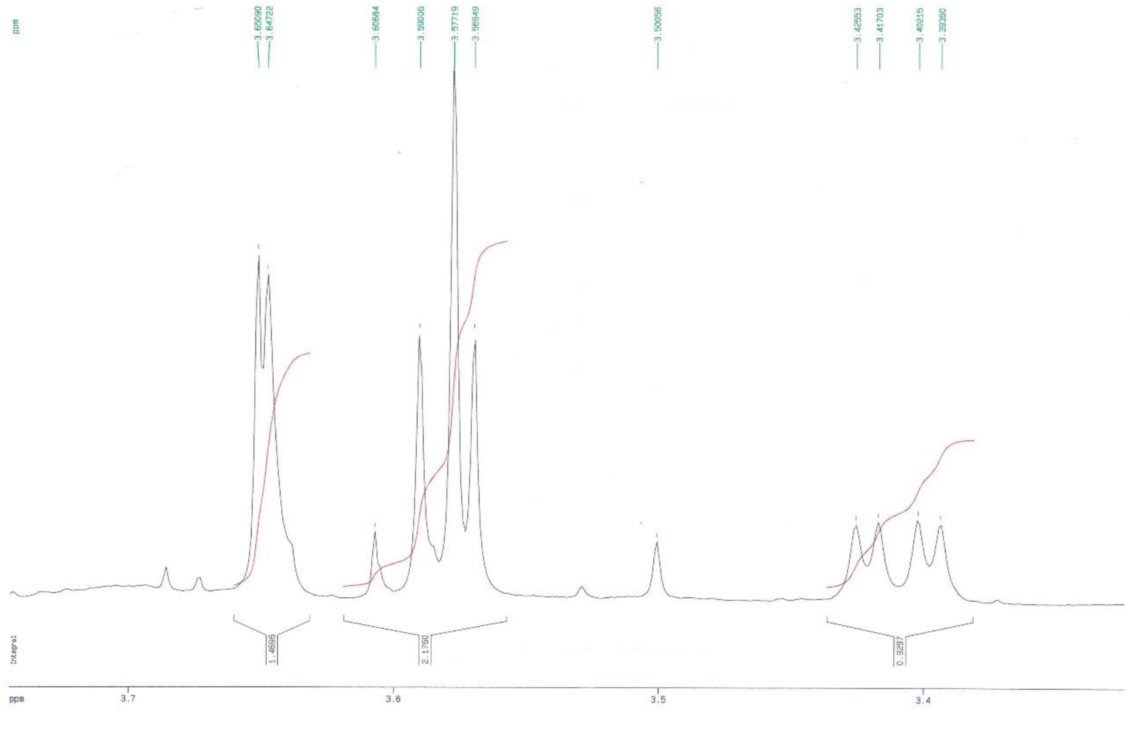

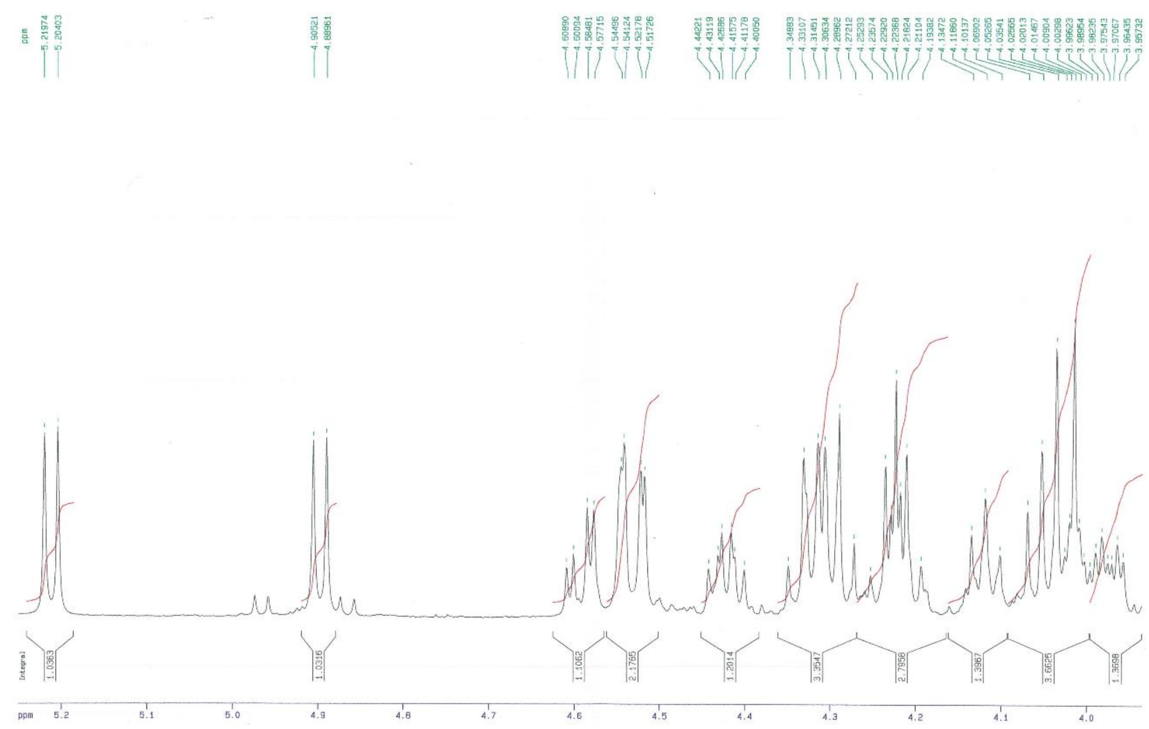

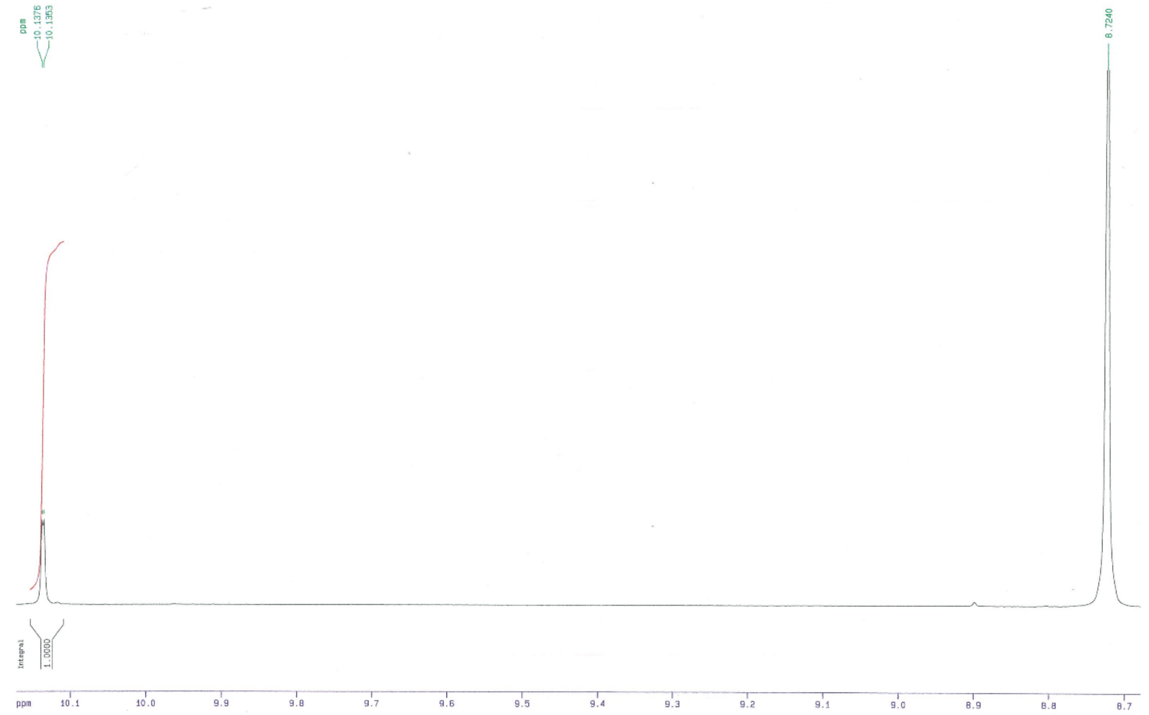

^{13}C-NMR spectrum of **6** in C_5D_5N

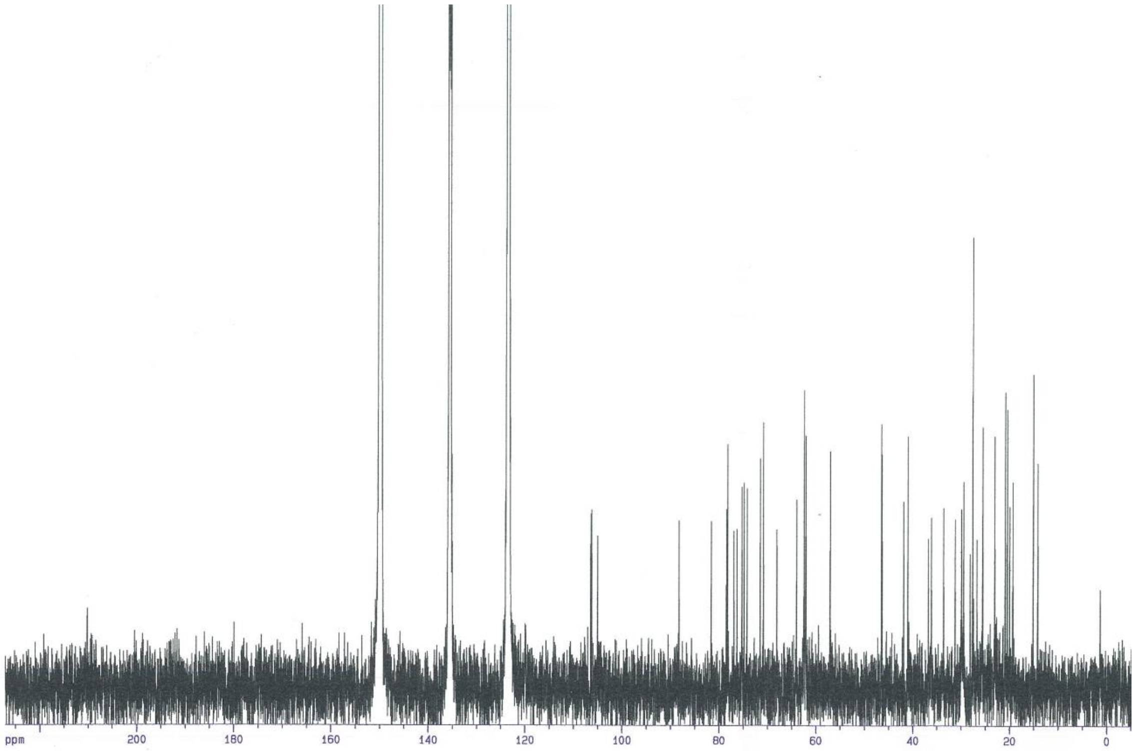

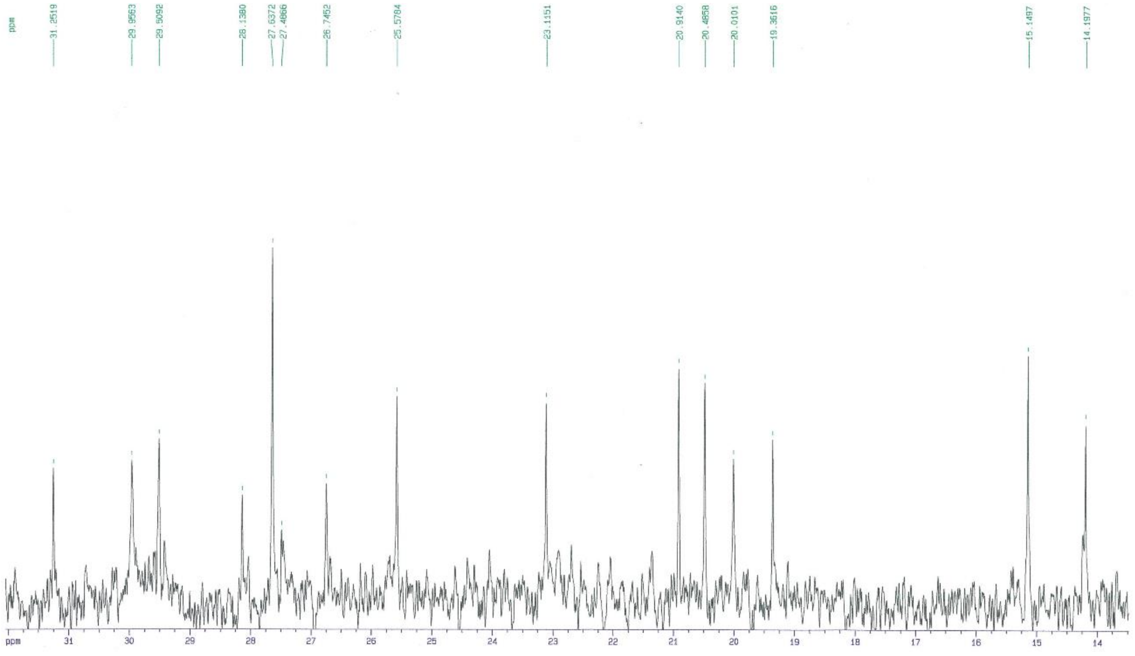

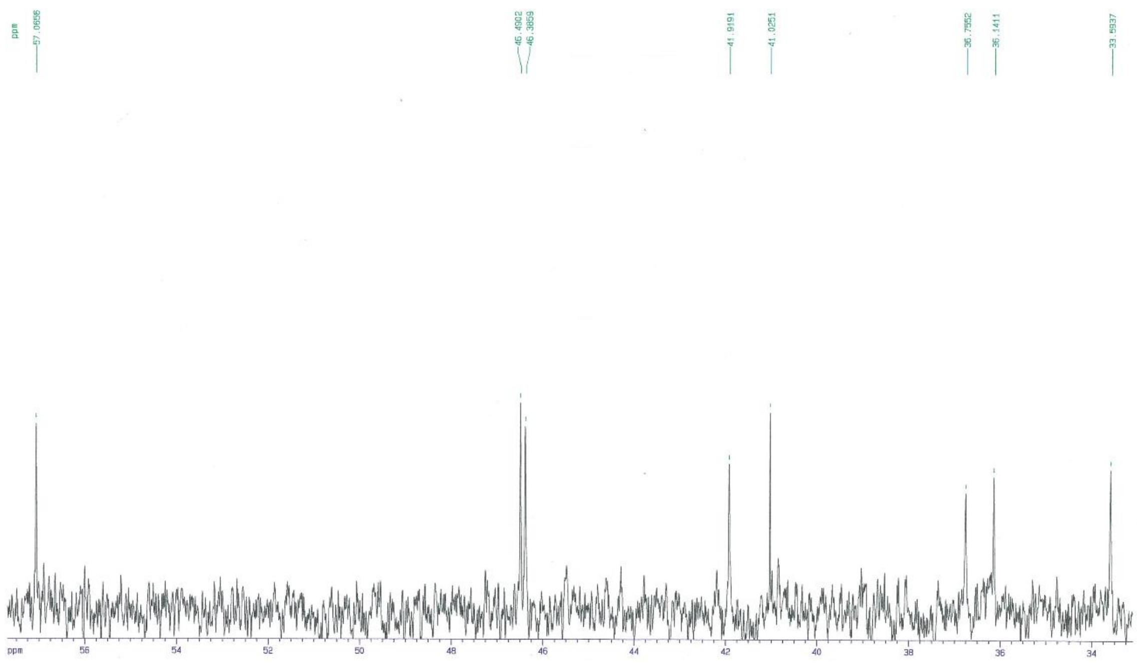

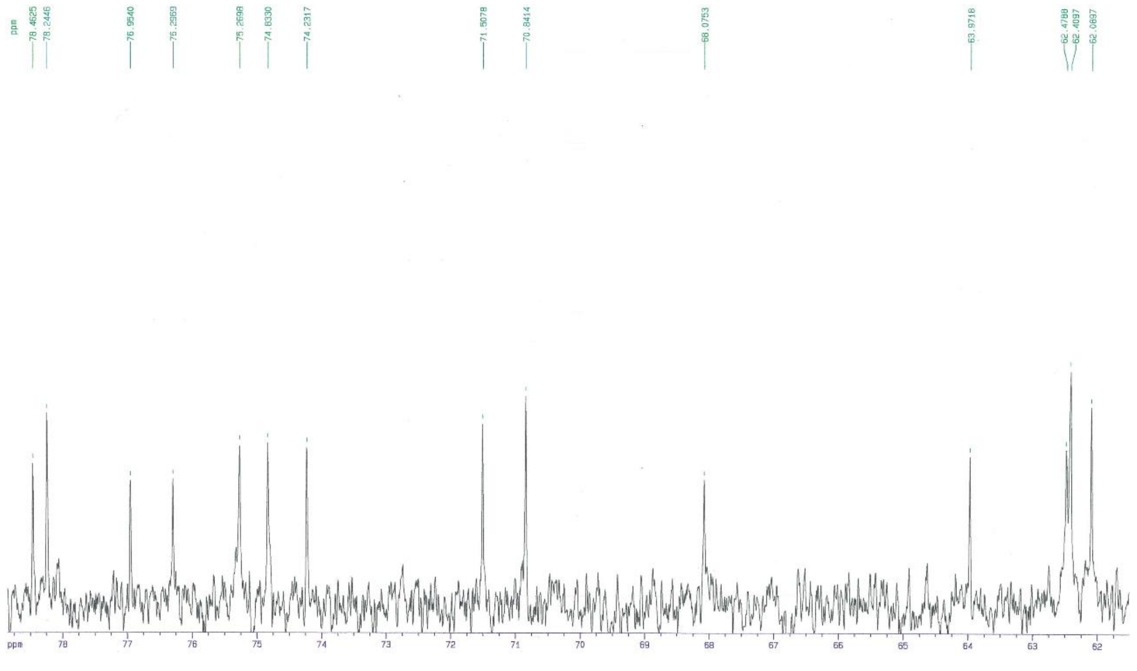

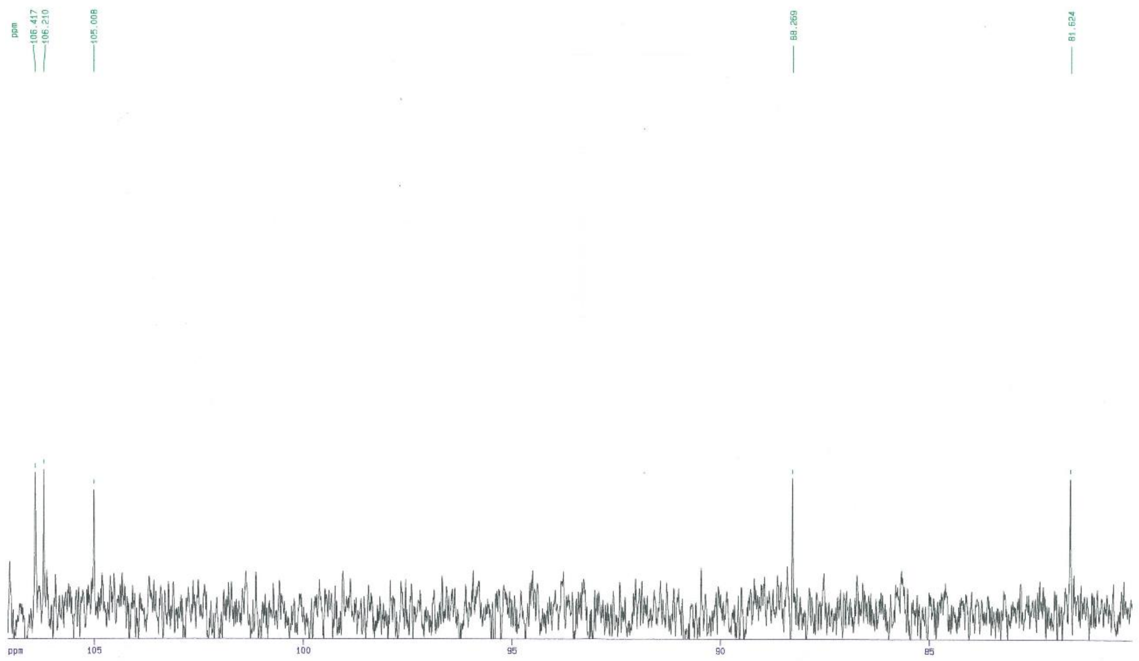

COSY spectrum of **6** in C_5D_5N

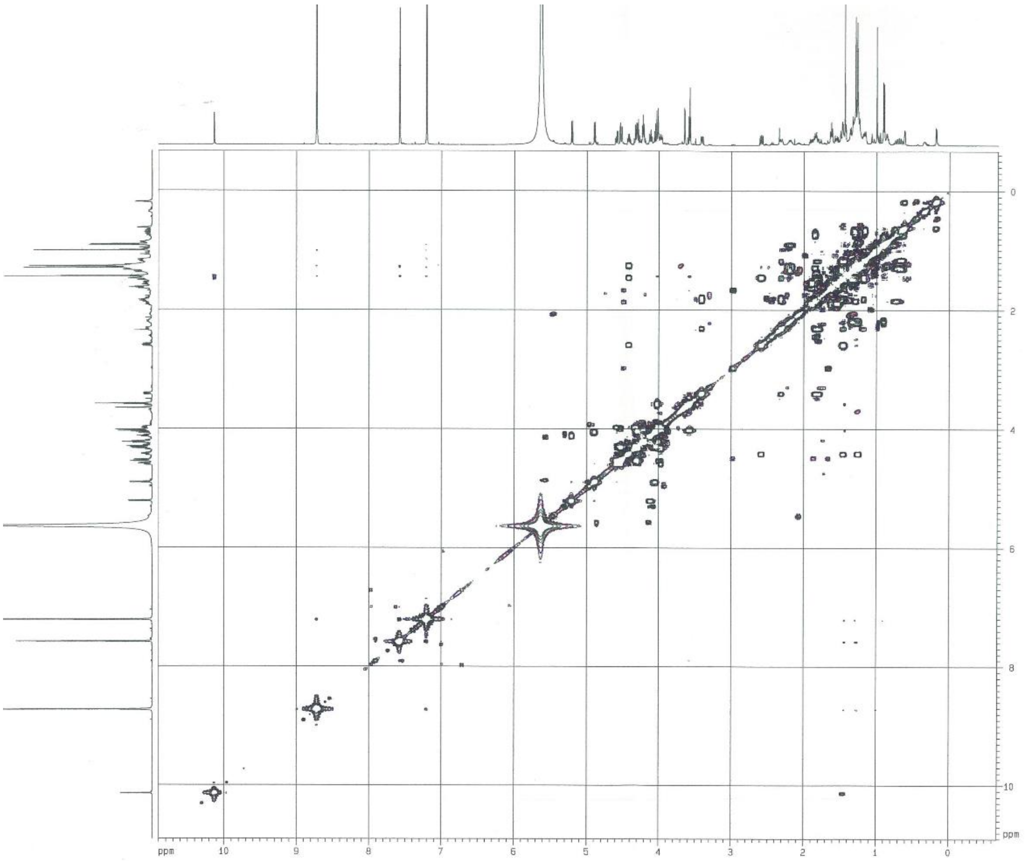

HMQC spectrum of **6** in C_5D_5N

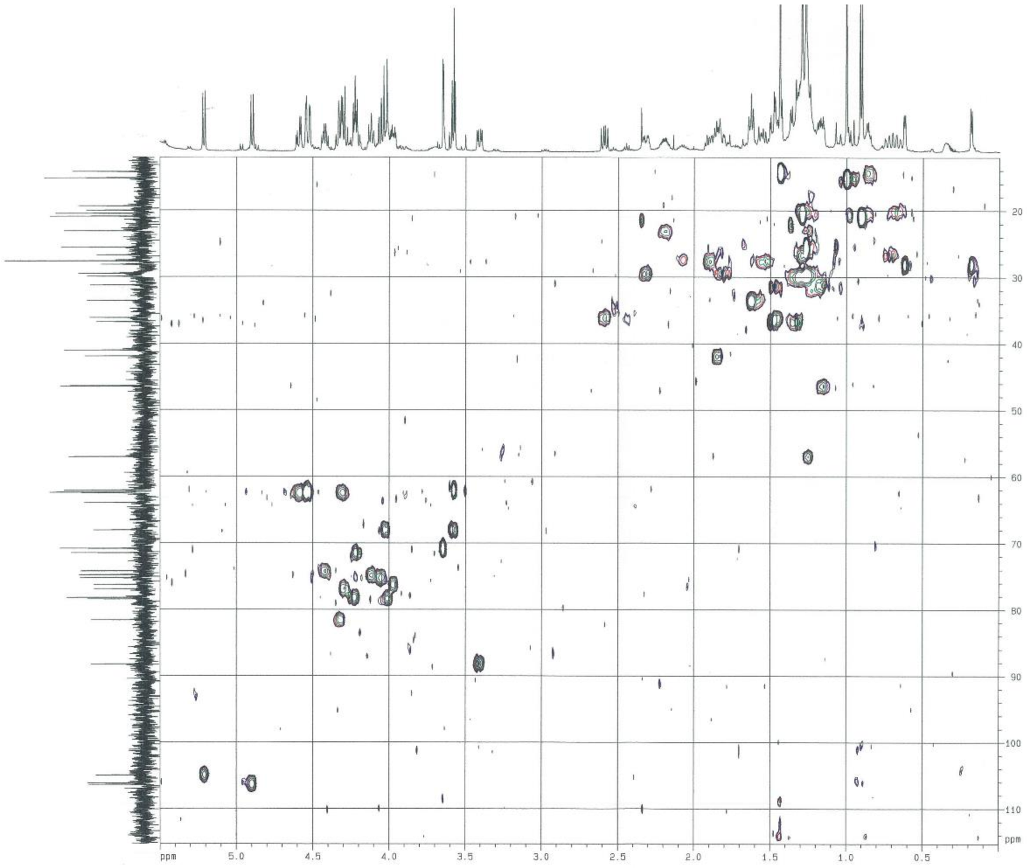

HMBC spectrum of **6** in C_5D_5N

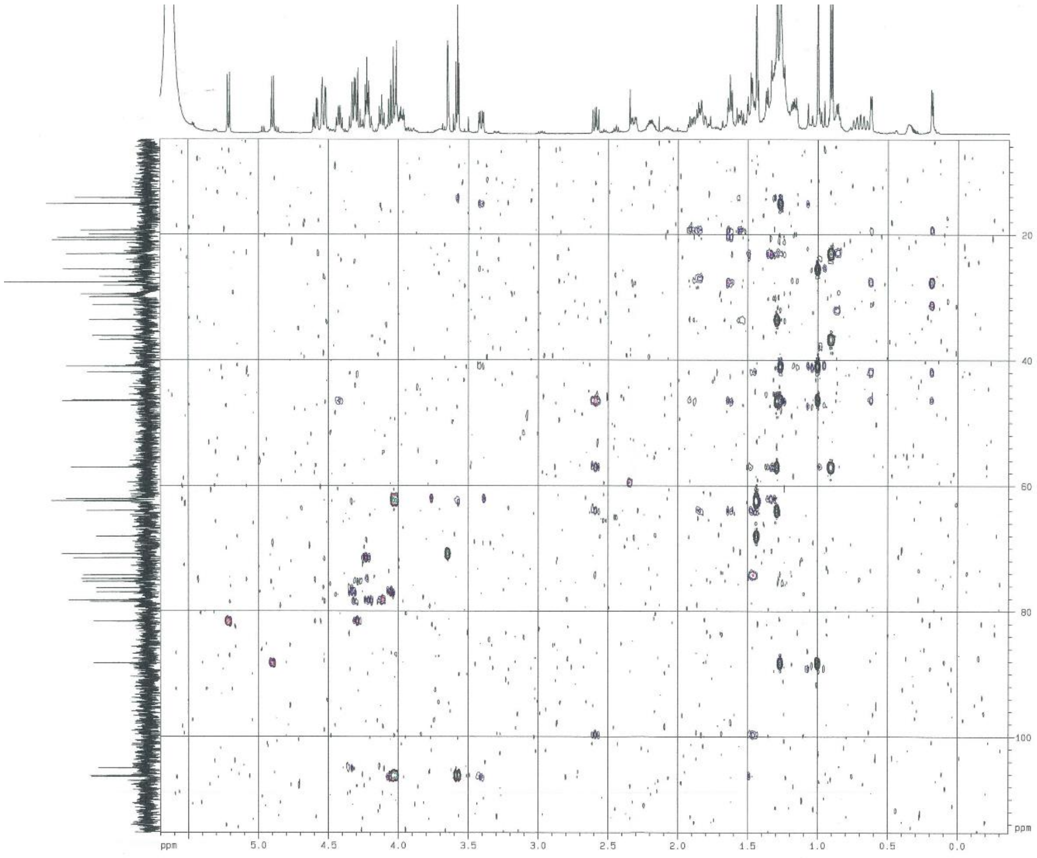

^{1}H-NMR spectrum of **7** in C_5D_5N

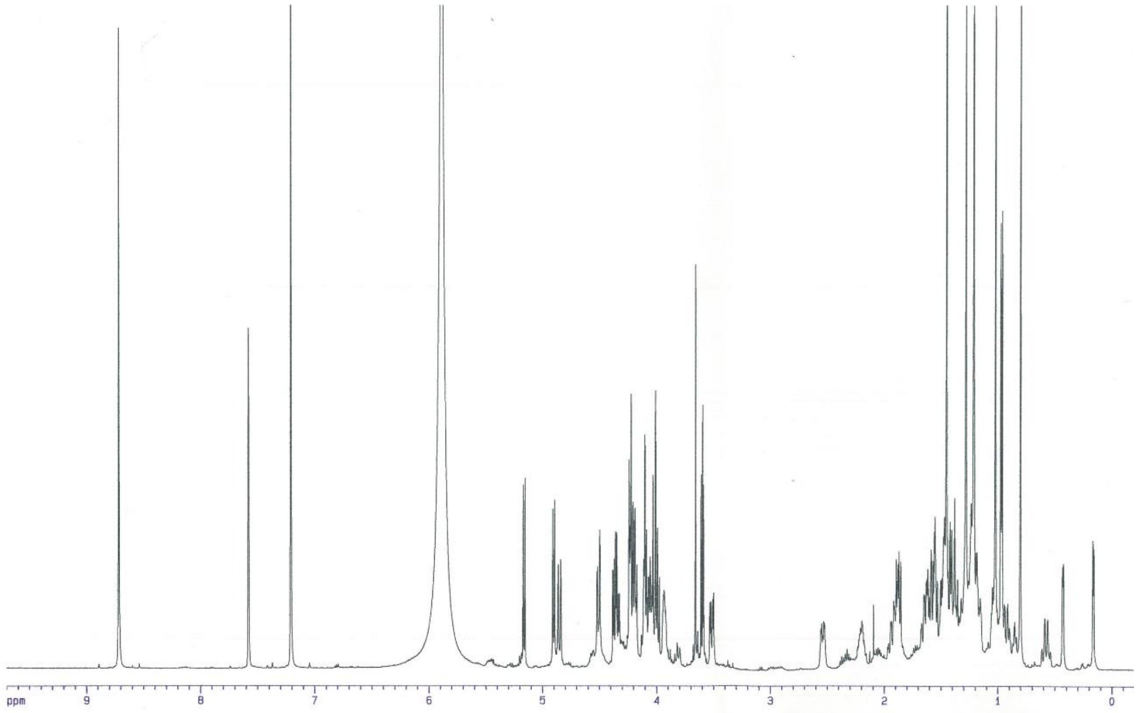

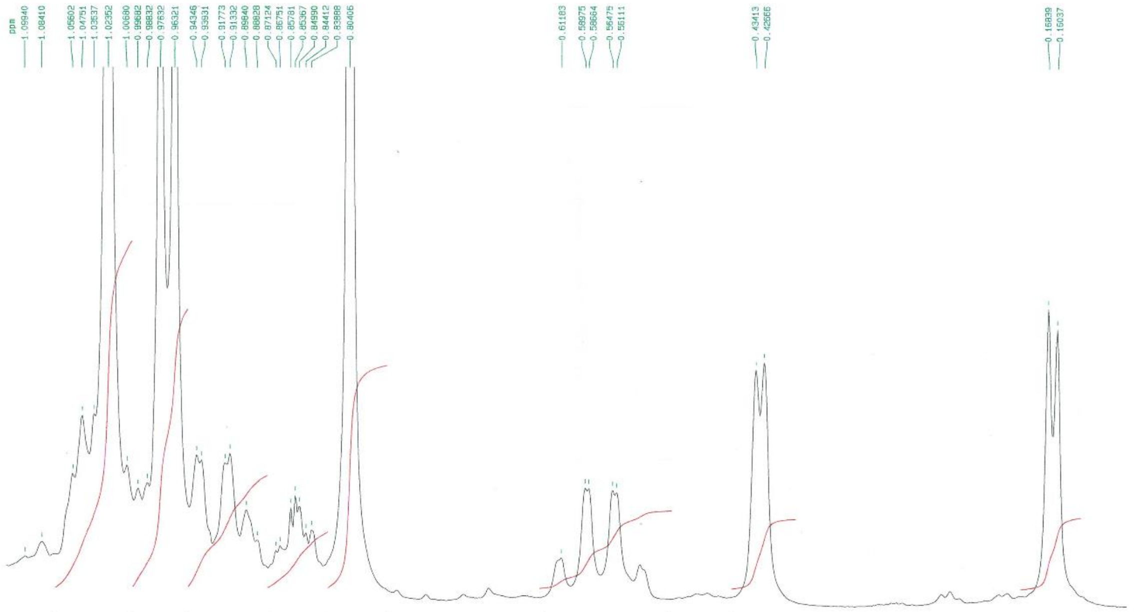

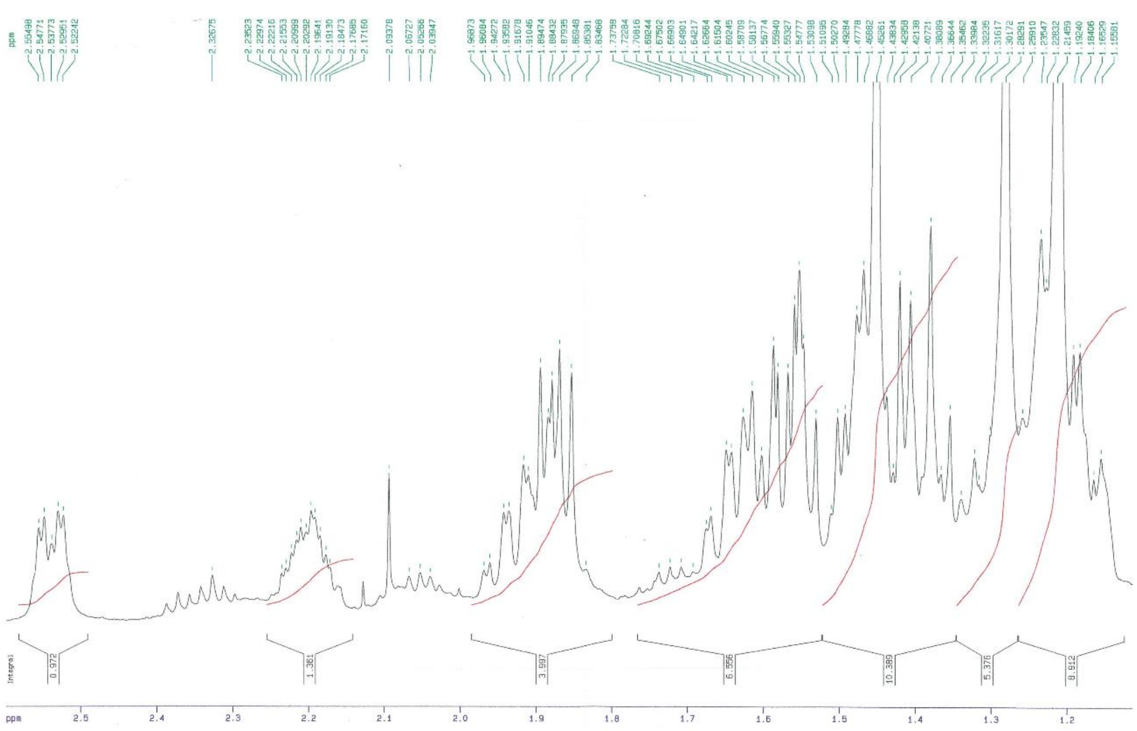

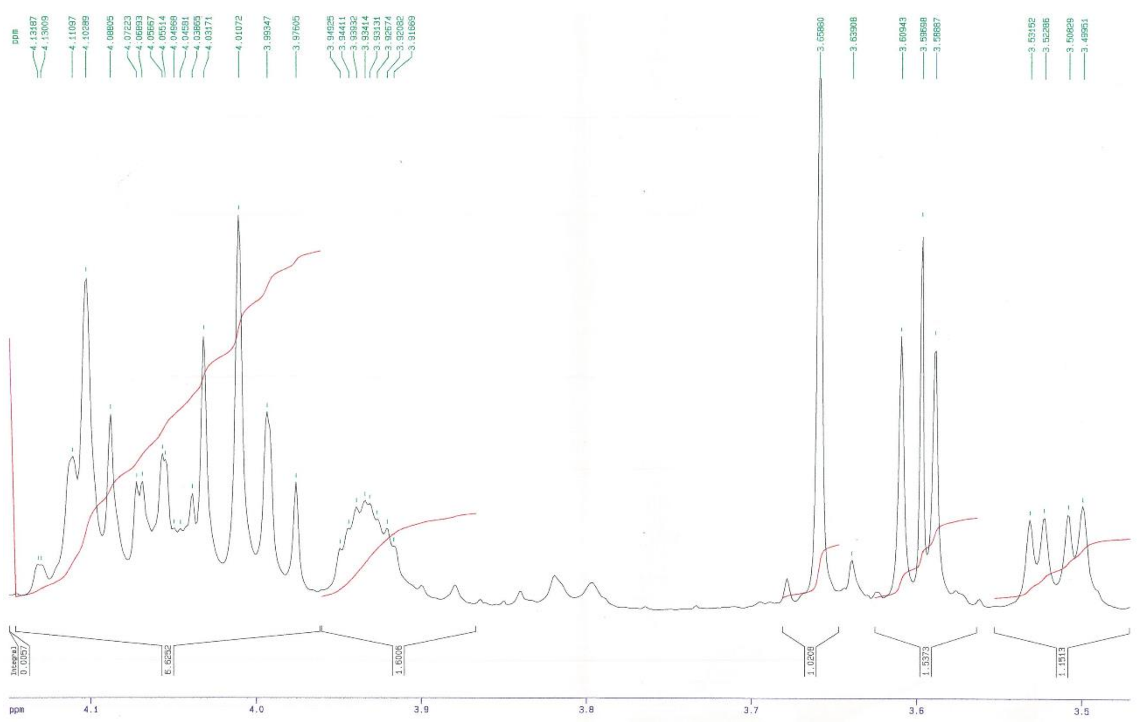

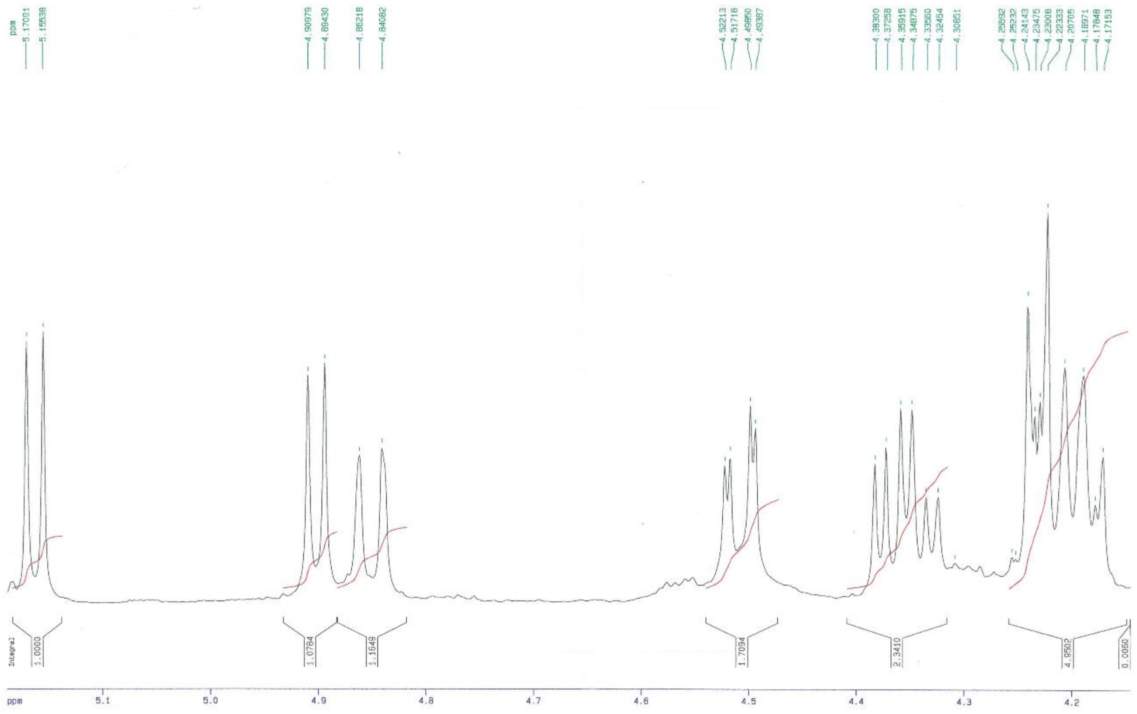

^{13}C-NMR spectrum of **7** in C_5D_5N

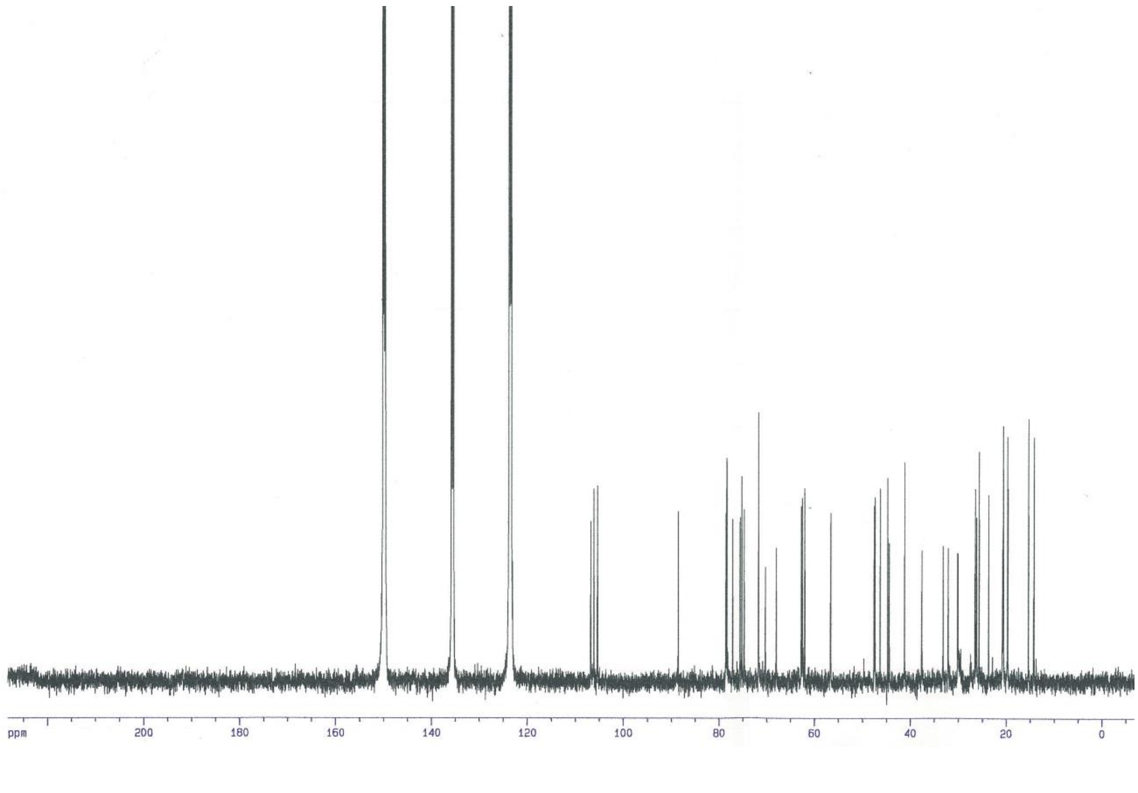

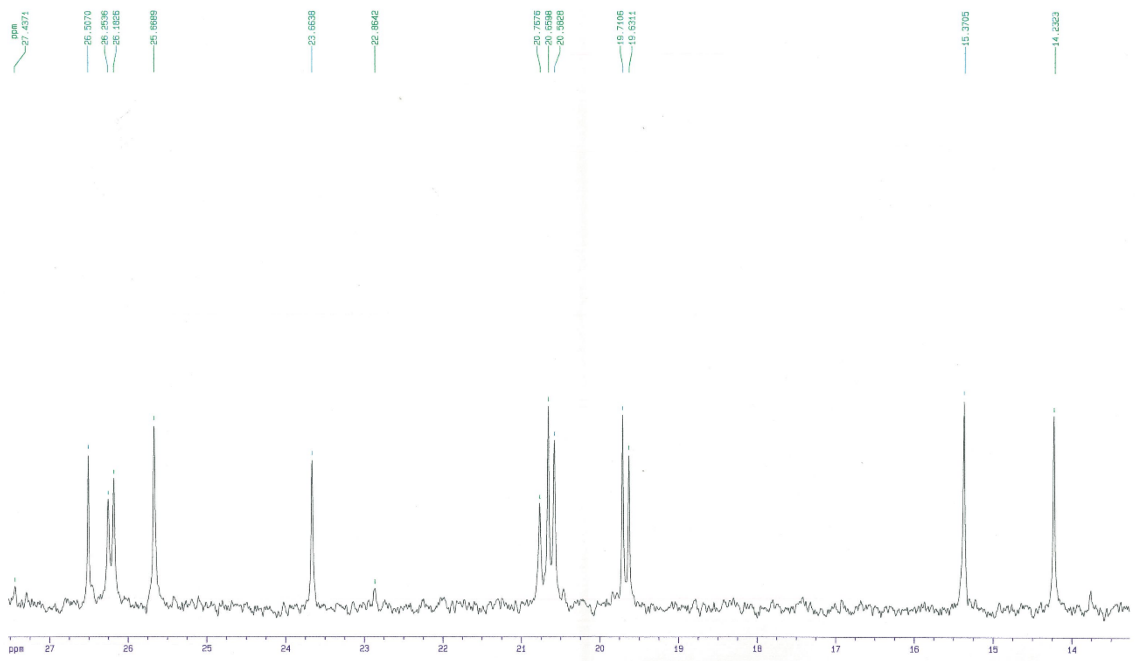
ppm
27.4371
26.5070
26.2536
26.1826
25.6689
23.6638
22.8642
20.7676
20.6598
20.5828
19.7106
19.6311
15.3705
14.2323
ppm 27 26 25 24 23 22 21 20 19 18 17 16 15 14

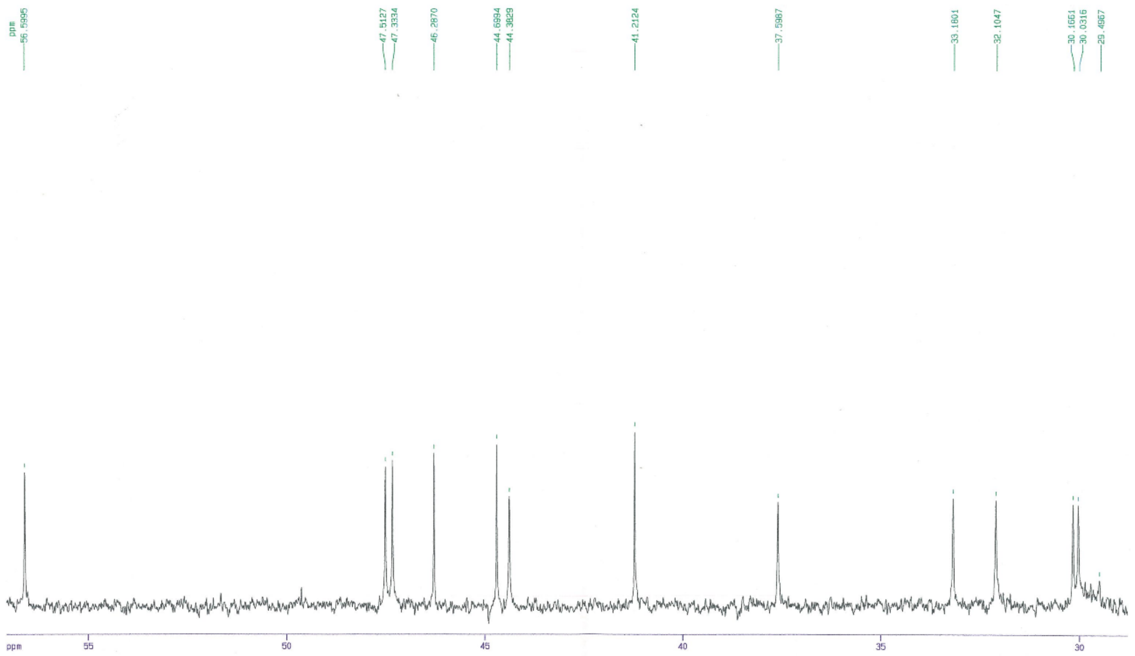
ppm
55.5995
47.5127
47.3334
46.2870
44.6994
44.3829
41.2124
37.5987
33.1801
32.1047
30.1651
30.0316
29.4967
ppm 55 50 45 40 35 30

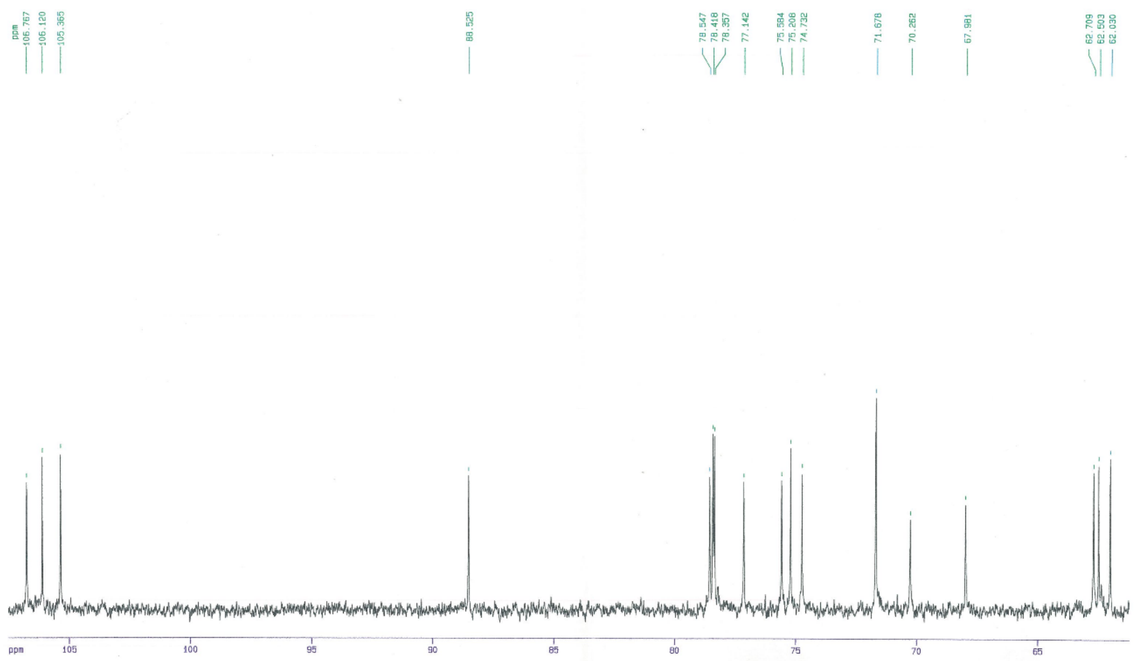

COSY spectrum of **7** in C_5D_5N

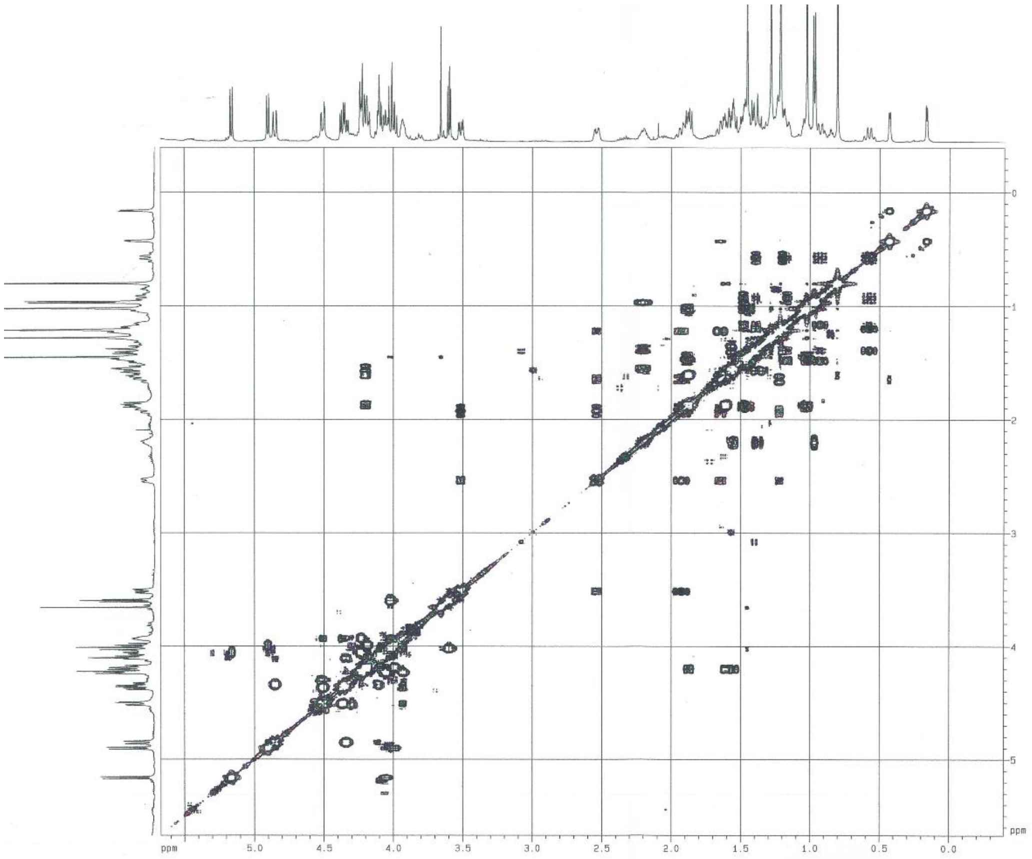

HMQC spectrum of **7** in C_5D_5N

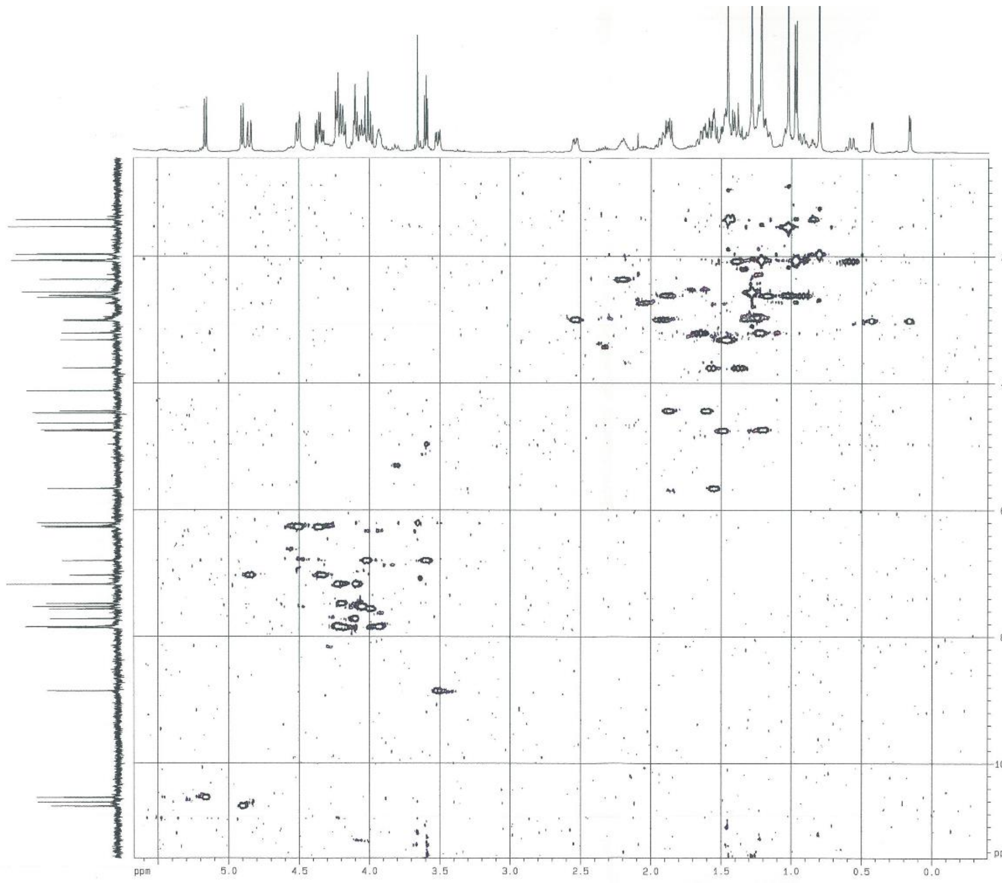

HMBC spectrum of **7** in C_5D_5N

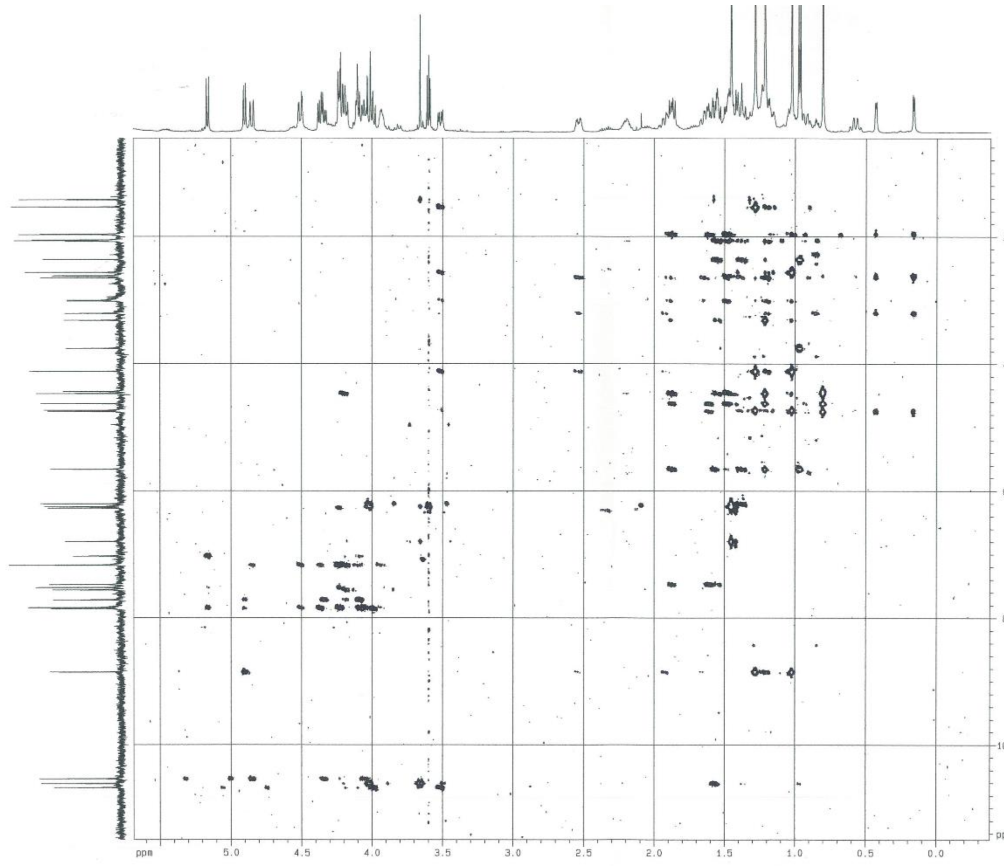

NOESY spectrum of **7** in C_5D_5N

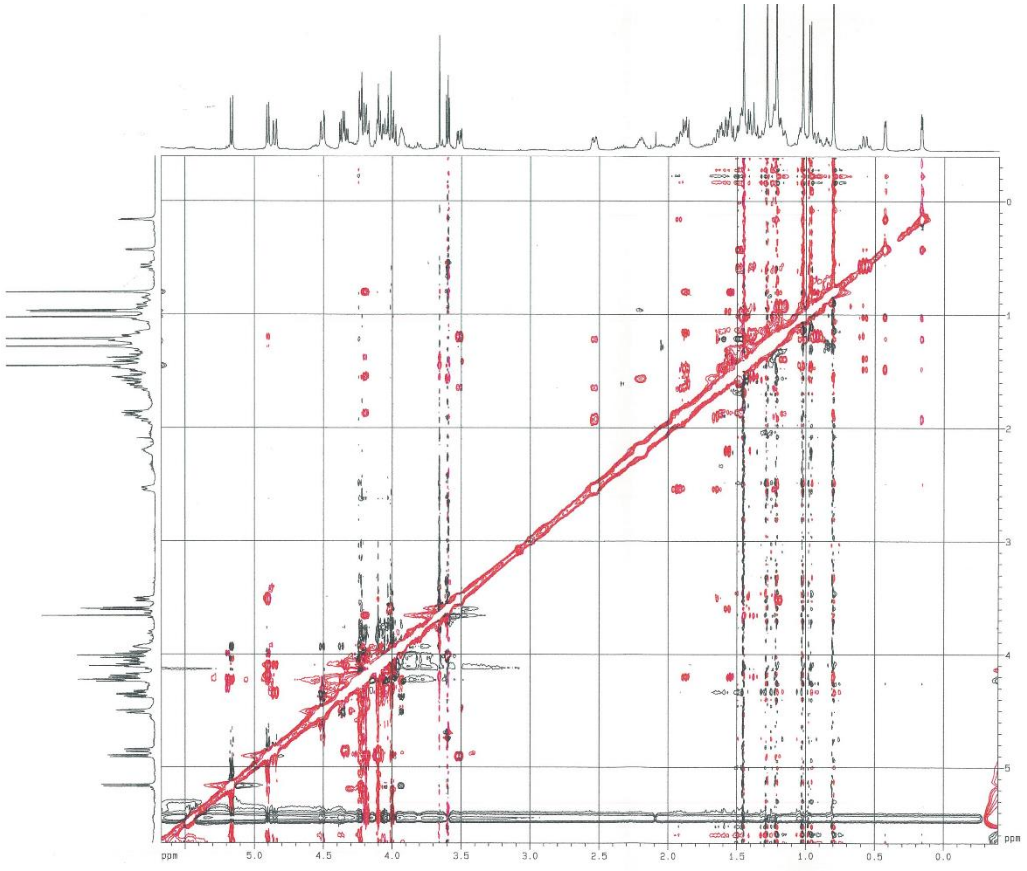

^{1}H-NMR spectrum of **8** in C_5D_5N

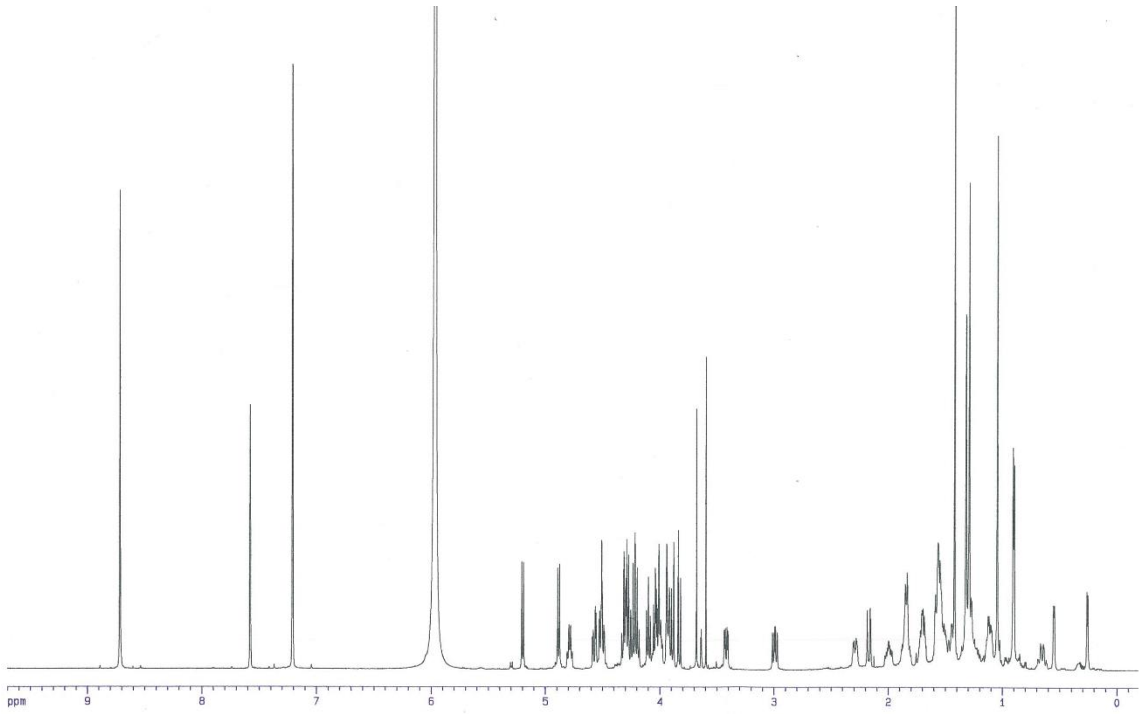

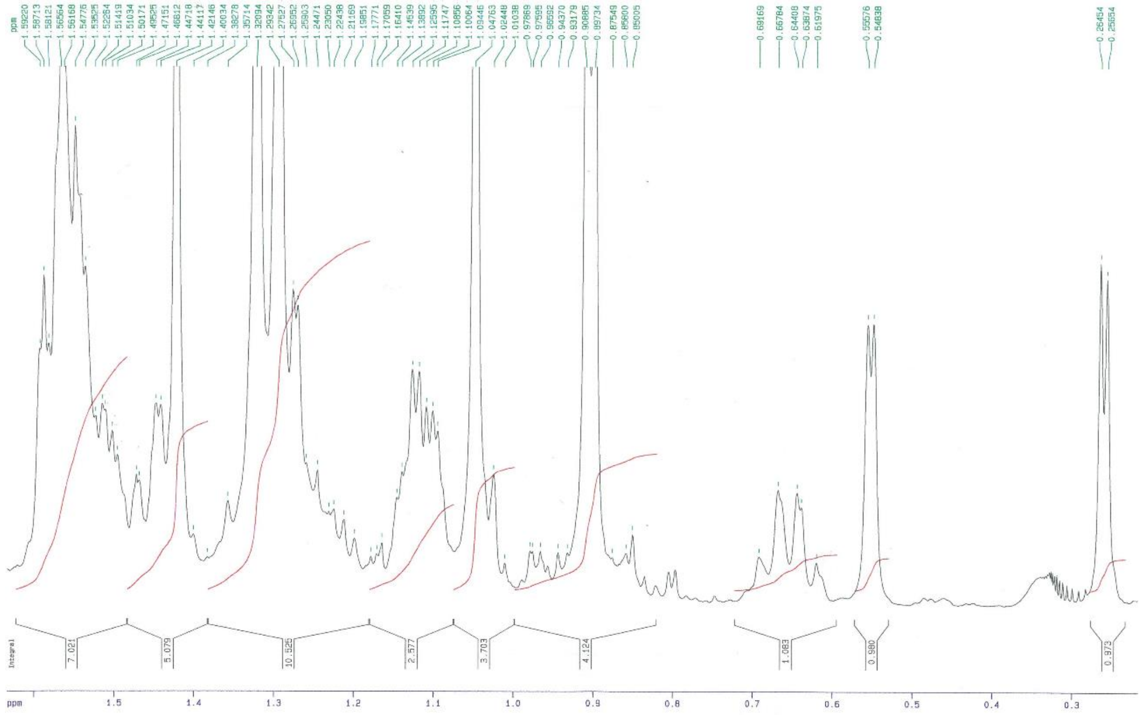
Integral
7.021
5.079
10.526
2.577
3.703
4.124
1.083
0.980
0.973
ppm

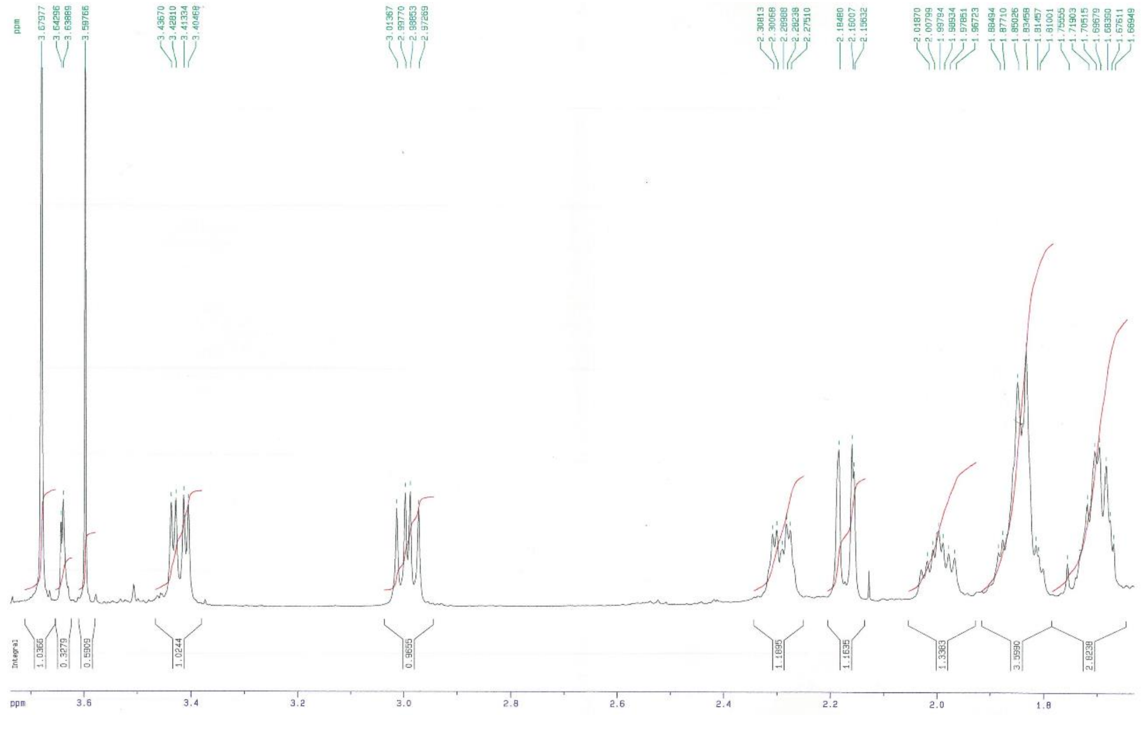
Integral
1.0366
0.3279
0.5909
1.0244
0.9855
1.1895
1.1636
1.3383
3.3990
2.8238
ppm

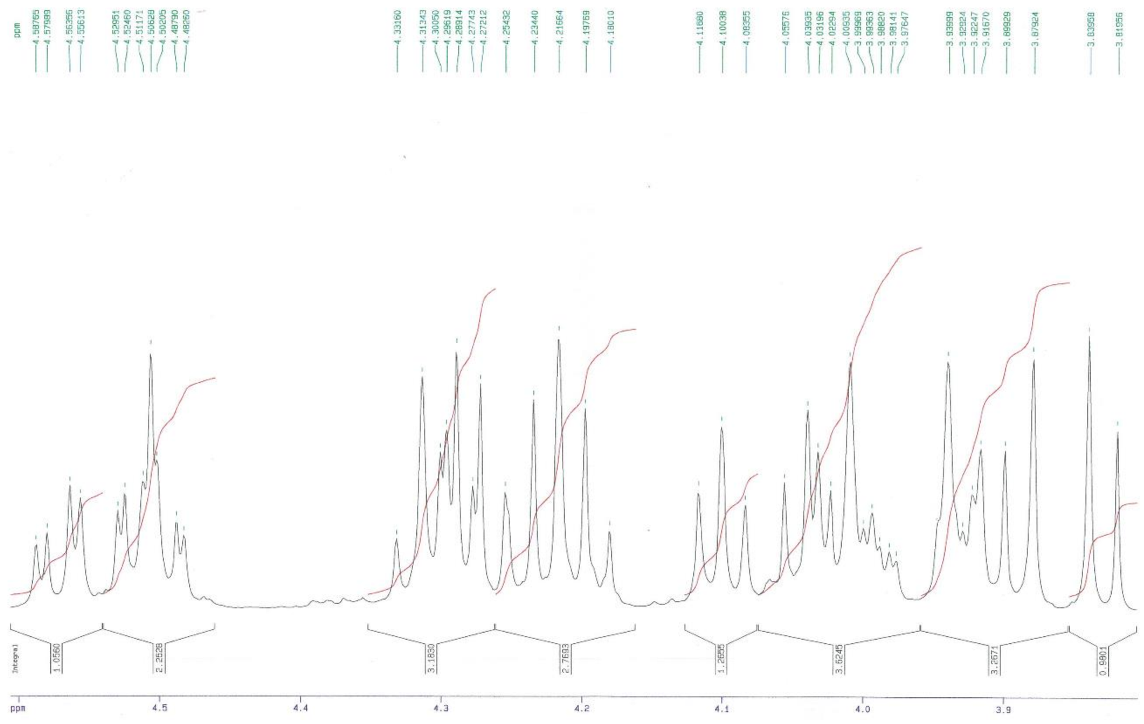

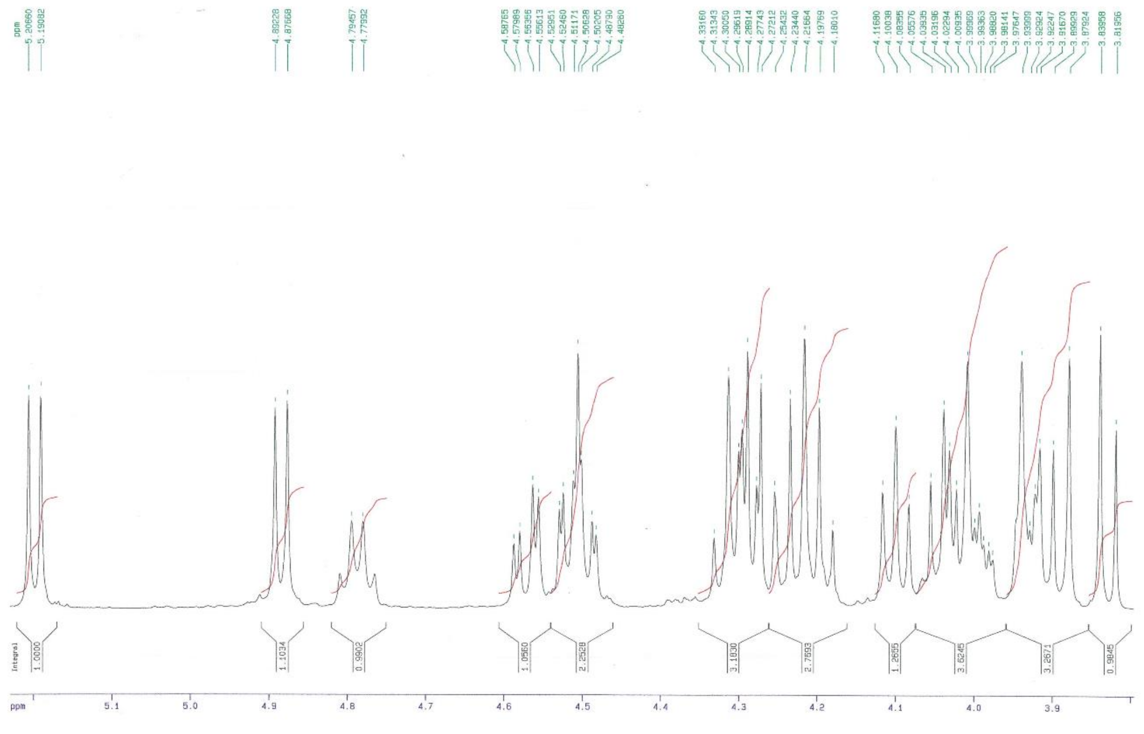

^{13}C-NMR spectrum of **8** in C_5D_5N

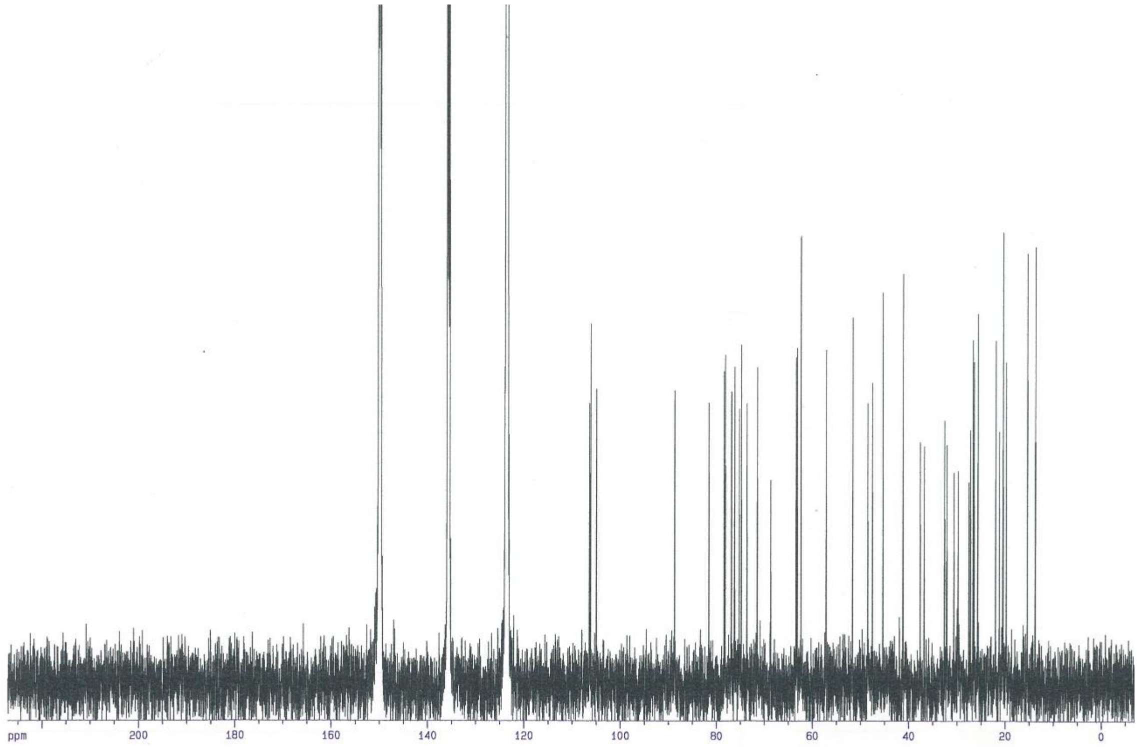

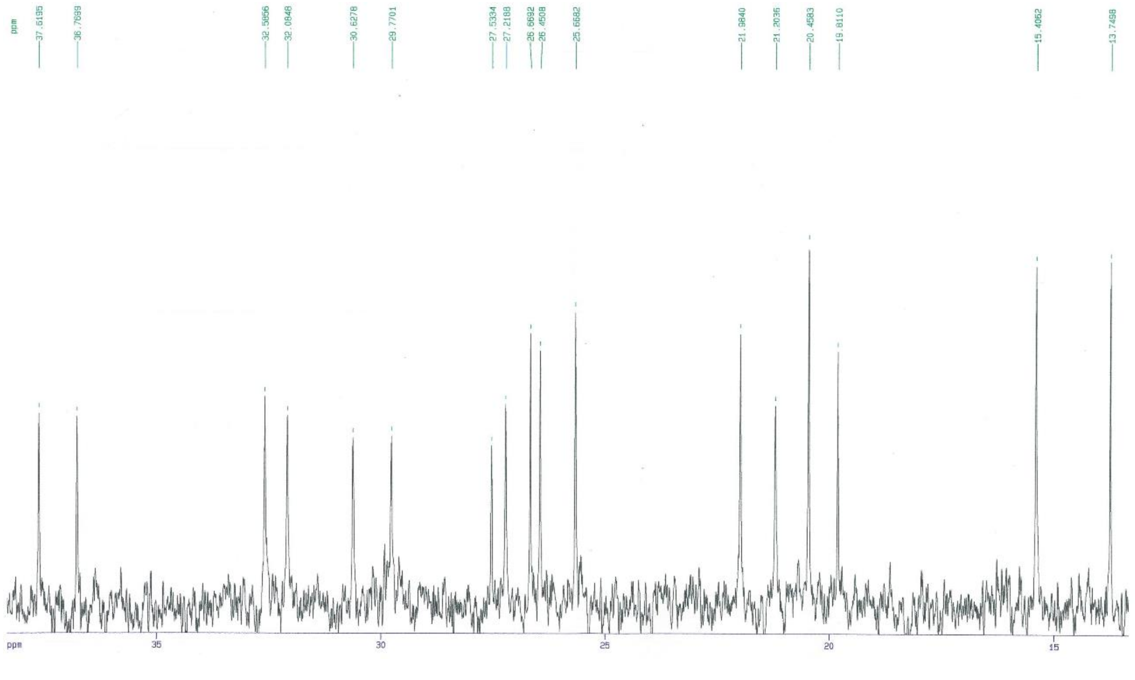

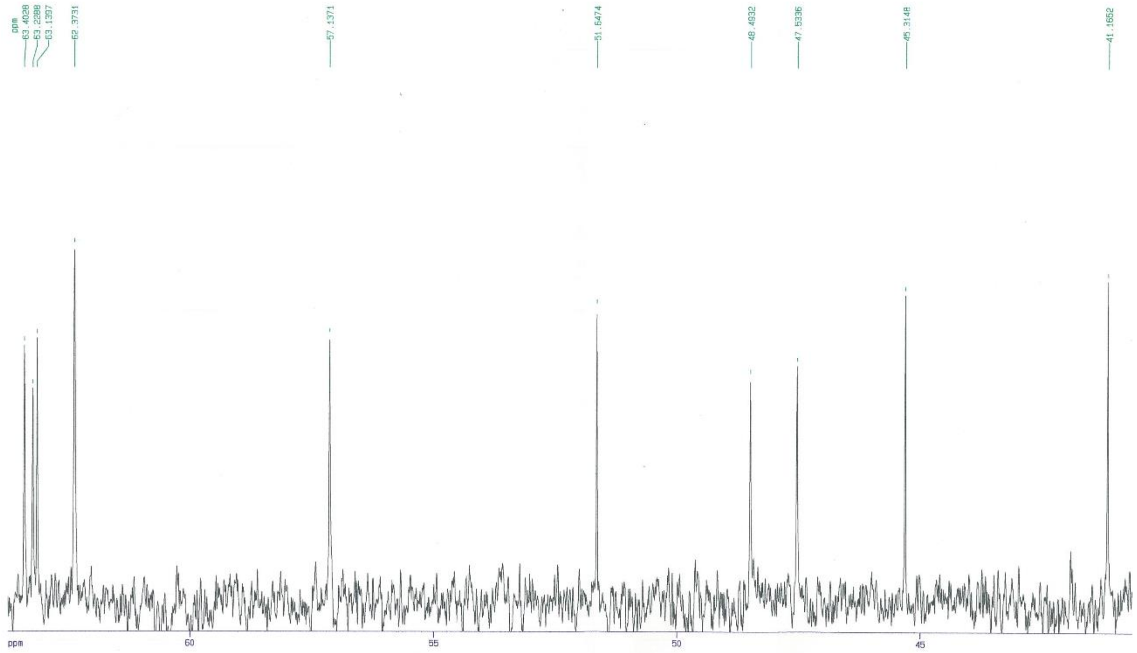

ppm
63.4028
63.2880
63.1397
62.3731
57.1371
51.5474
48.4932
47.5336
45.3148
41.1652
ppm
60
55
50
45

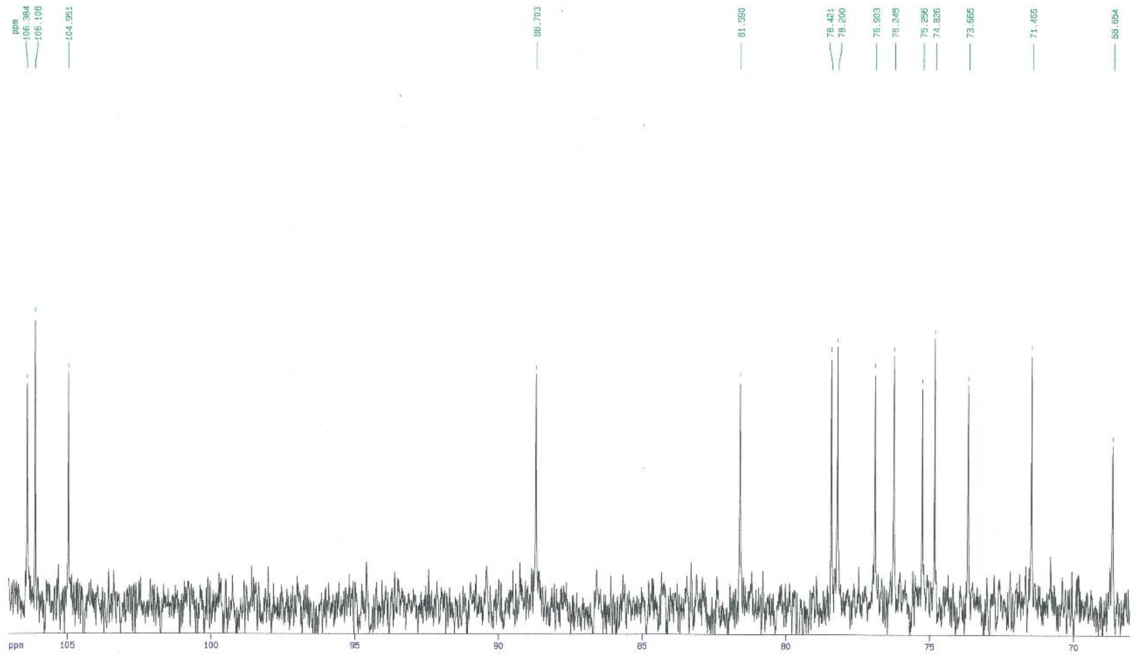

ppm
106.384
106.109
104.951
88.703
81.590
78.421
78.200
76.903
76.245
75.256
74.826
73.665
71.465
68.654
ppm
105
100
95
90
85
80
75
70

COSY spectrum of **8** in C_5D_5N

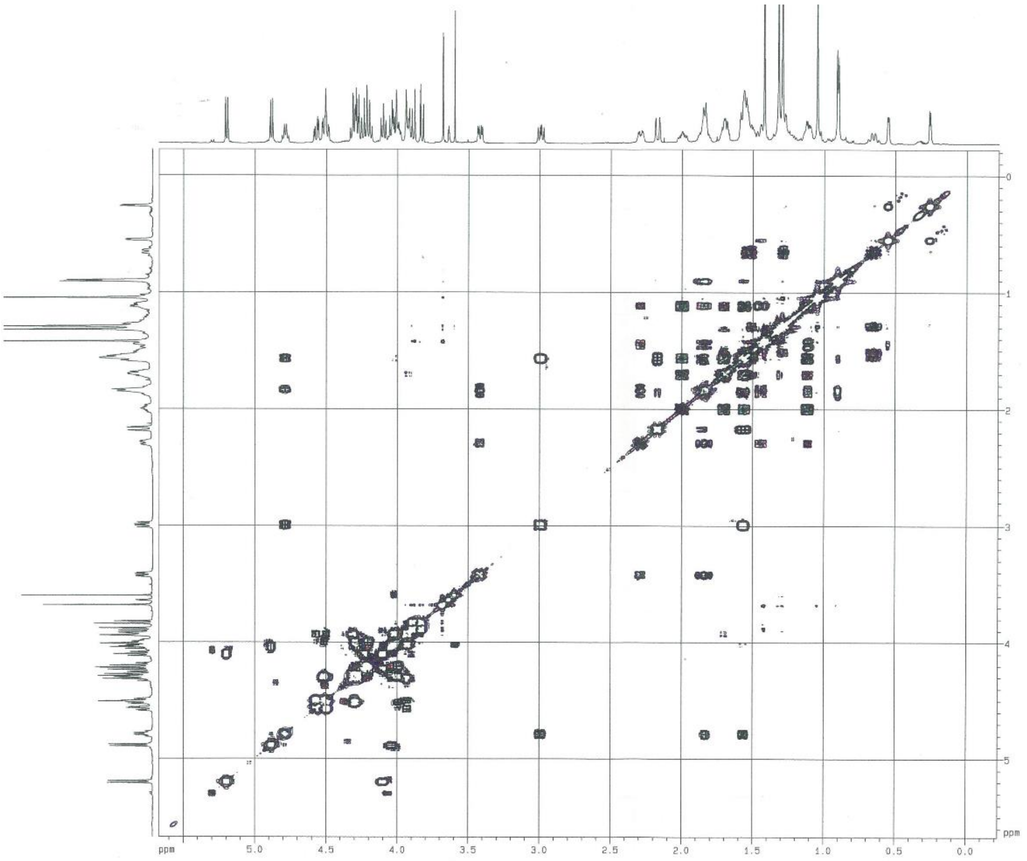

HMQC spectrum of **8** in C_5D_5N

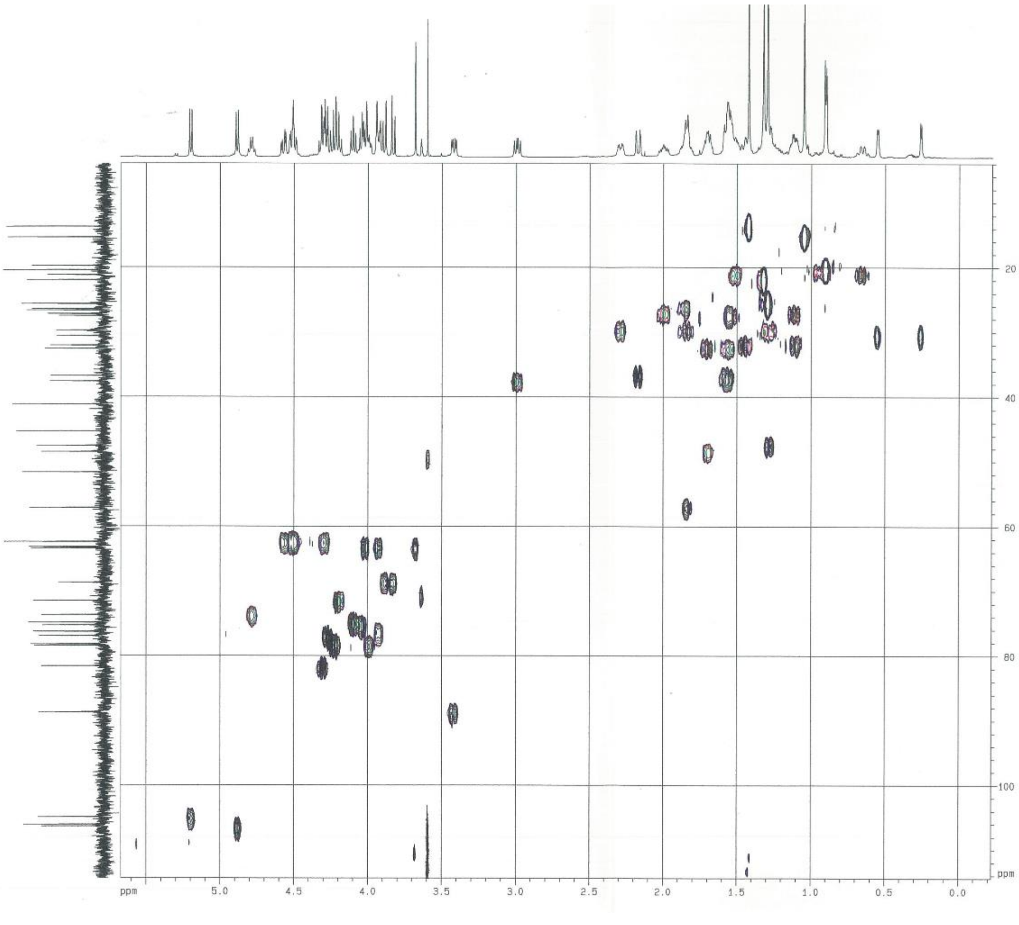

HMBC spectrum of **8** in C_5D_5N

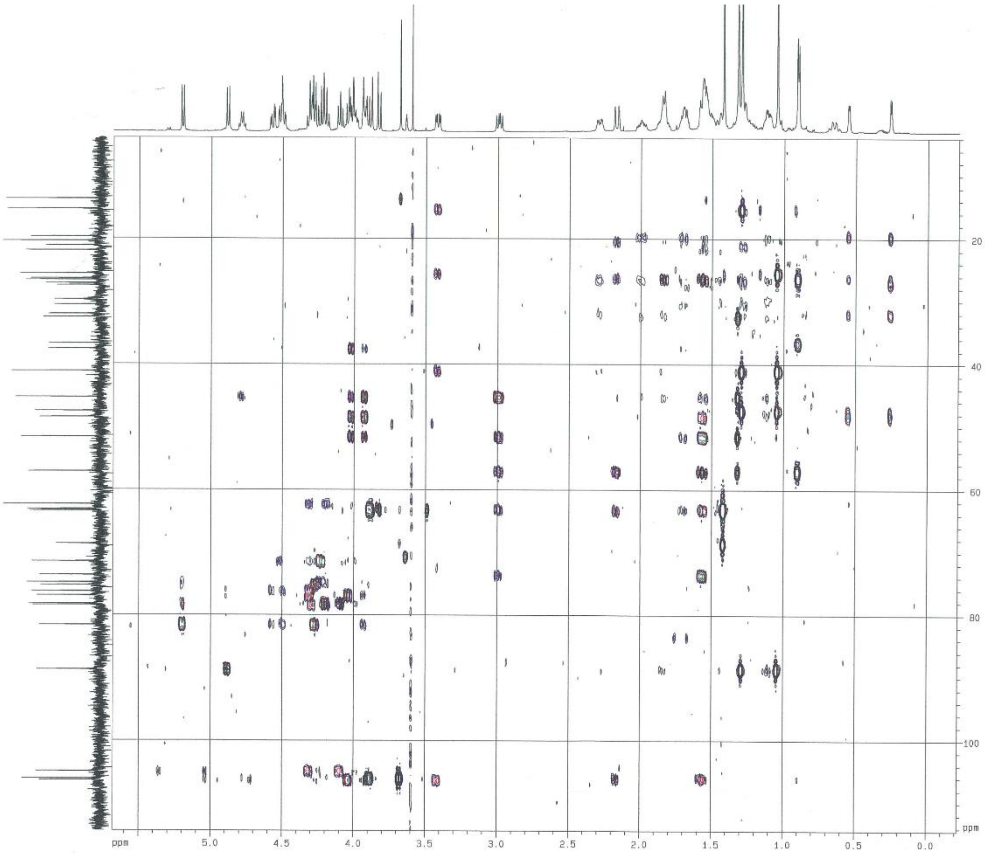

^{1}H-NMR spectrum of **8a** in C_5D_5N

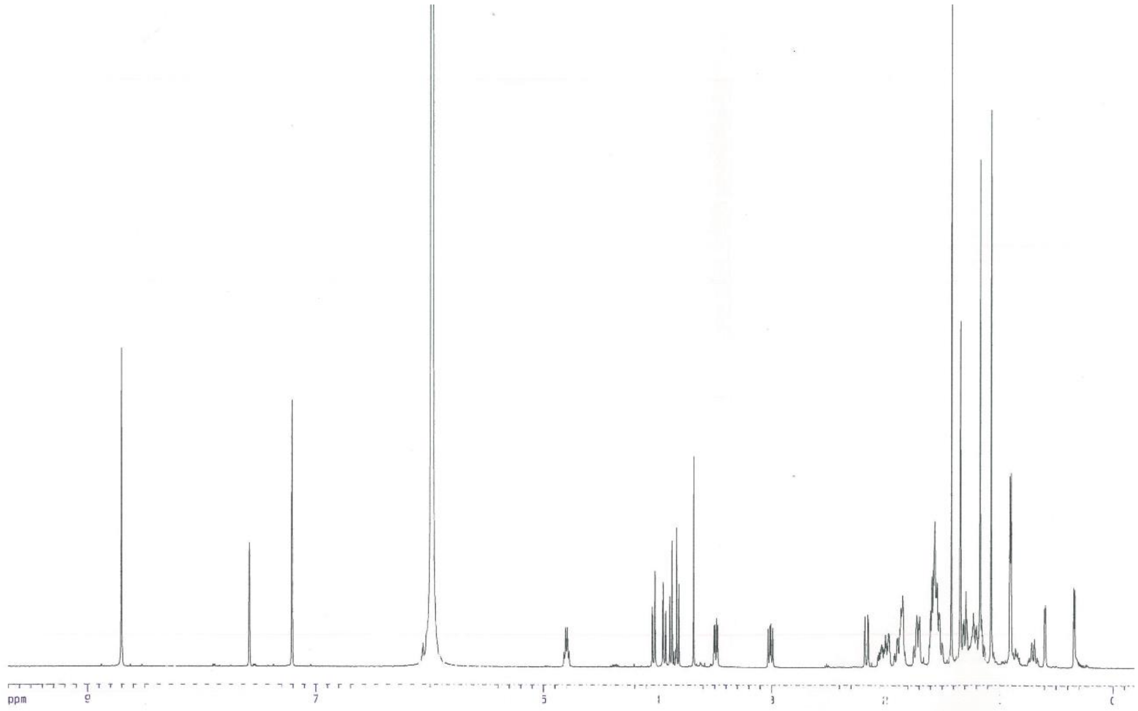

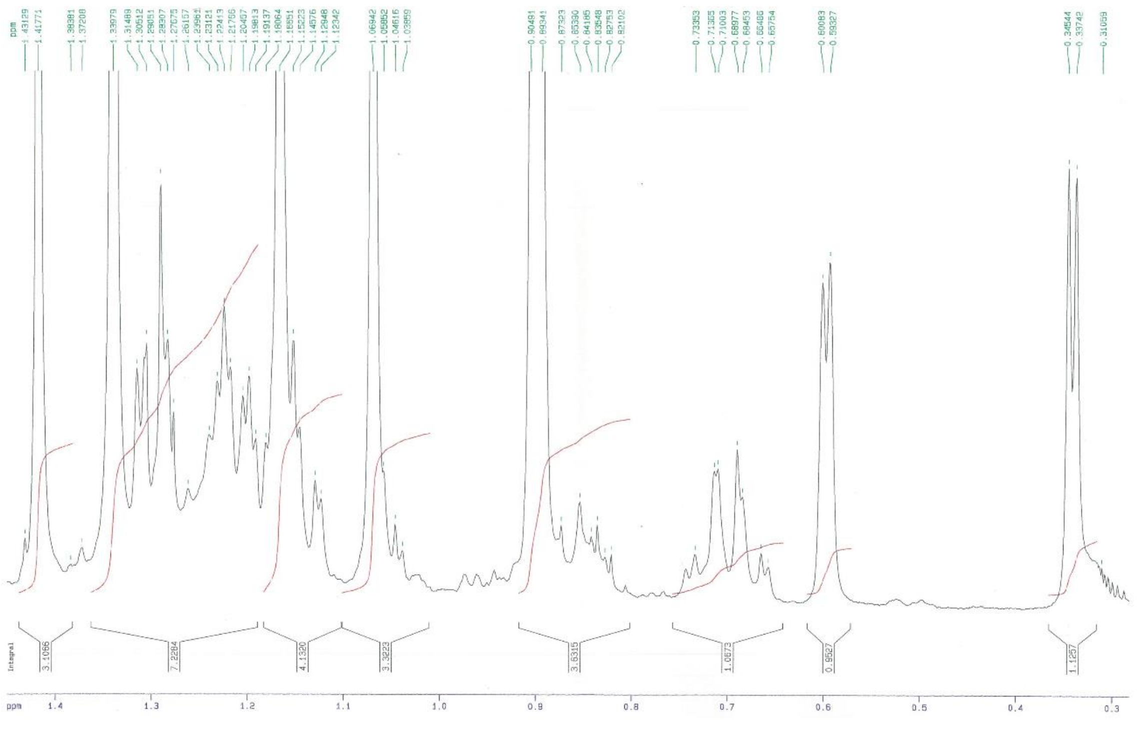

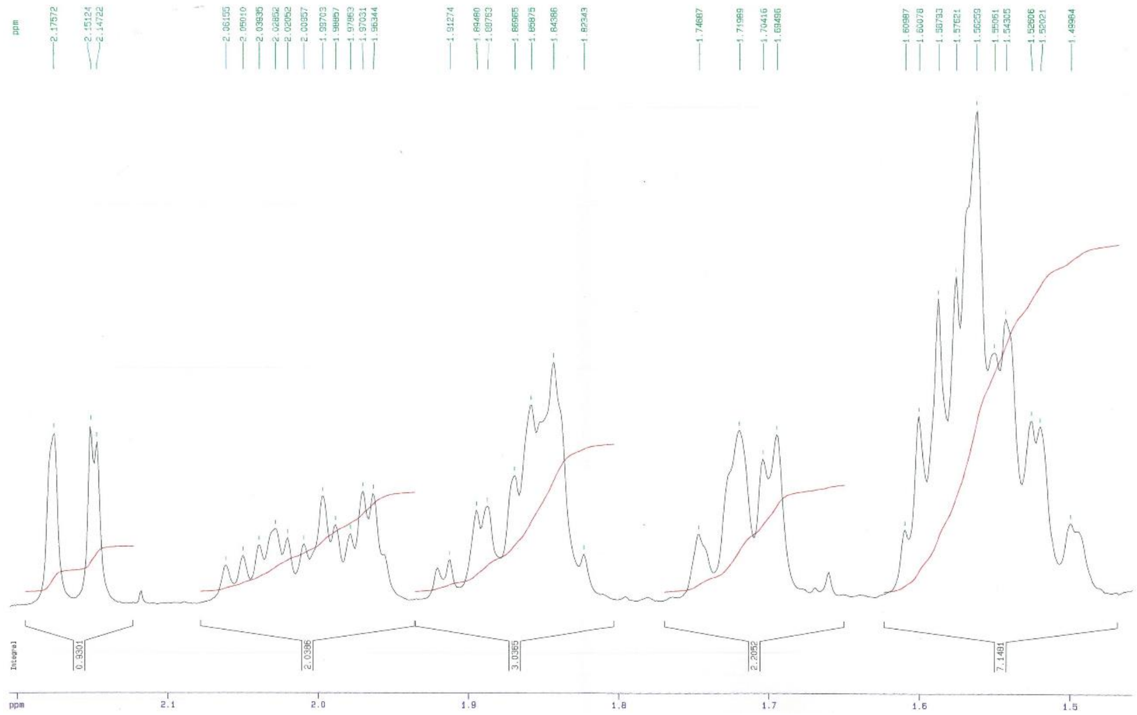

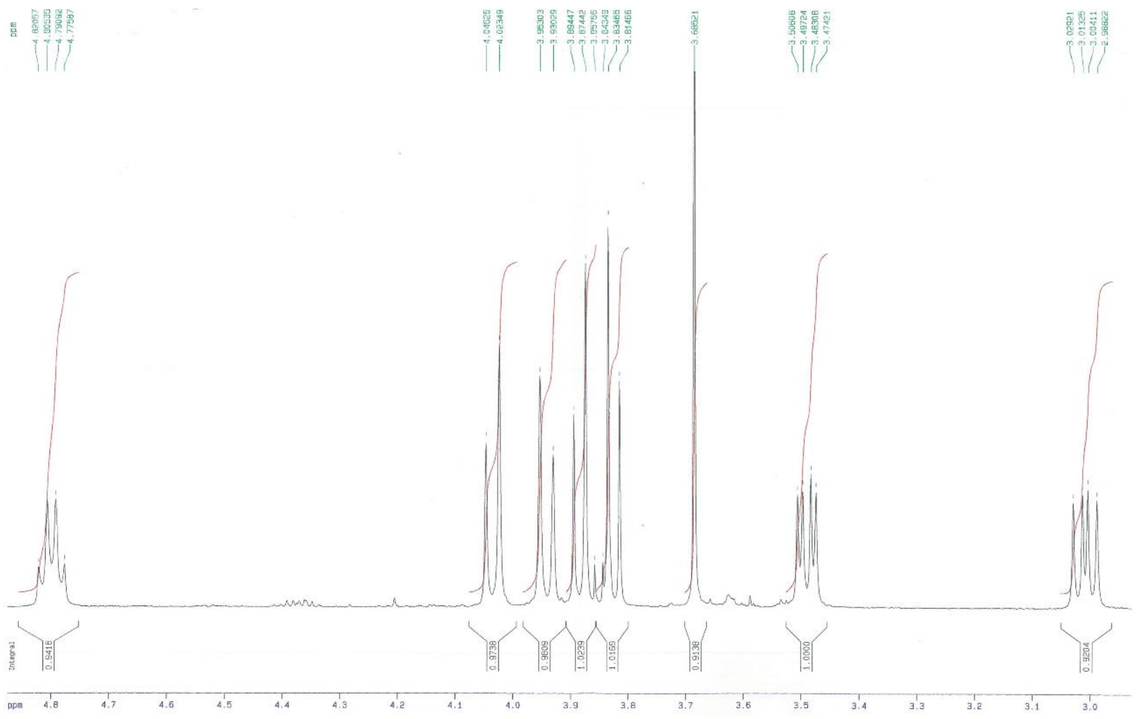

^{13}C-NMR spectrum of **8a** in C_5D_5N

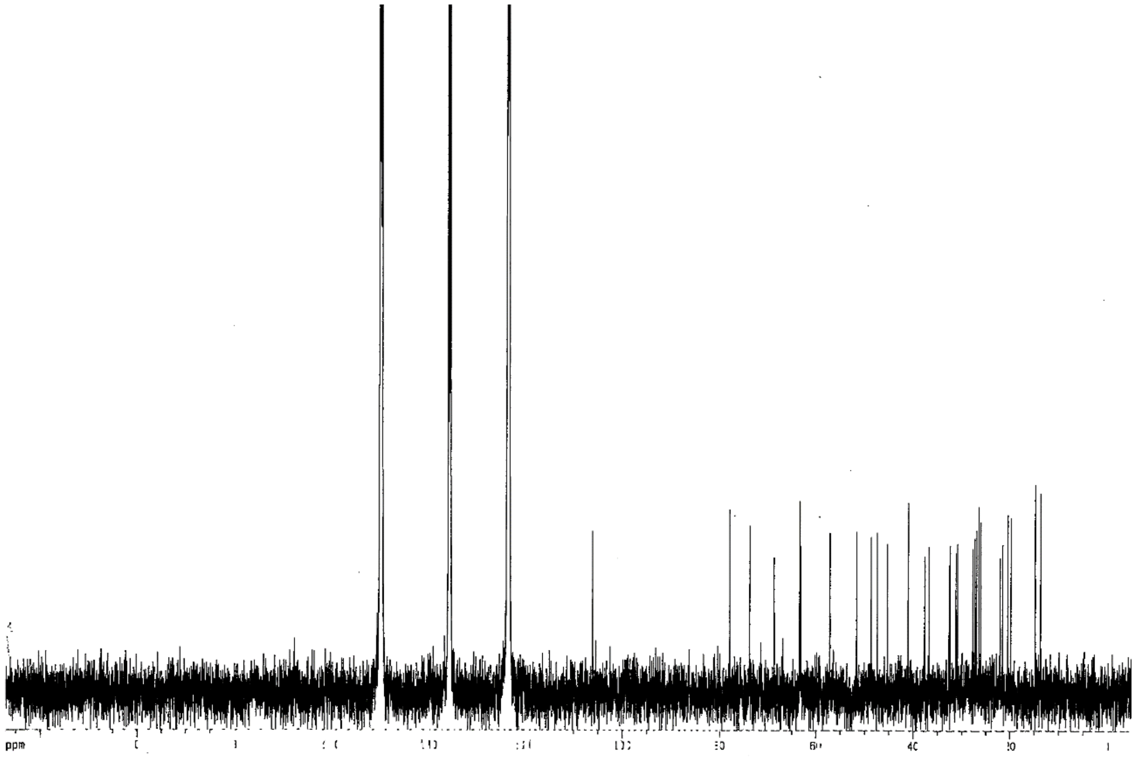

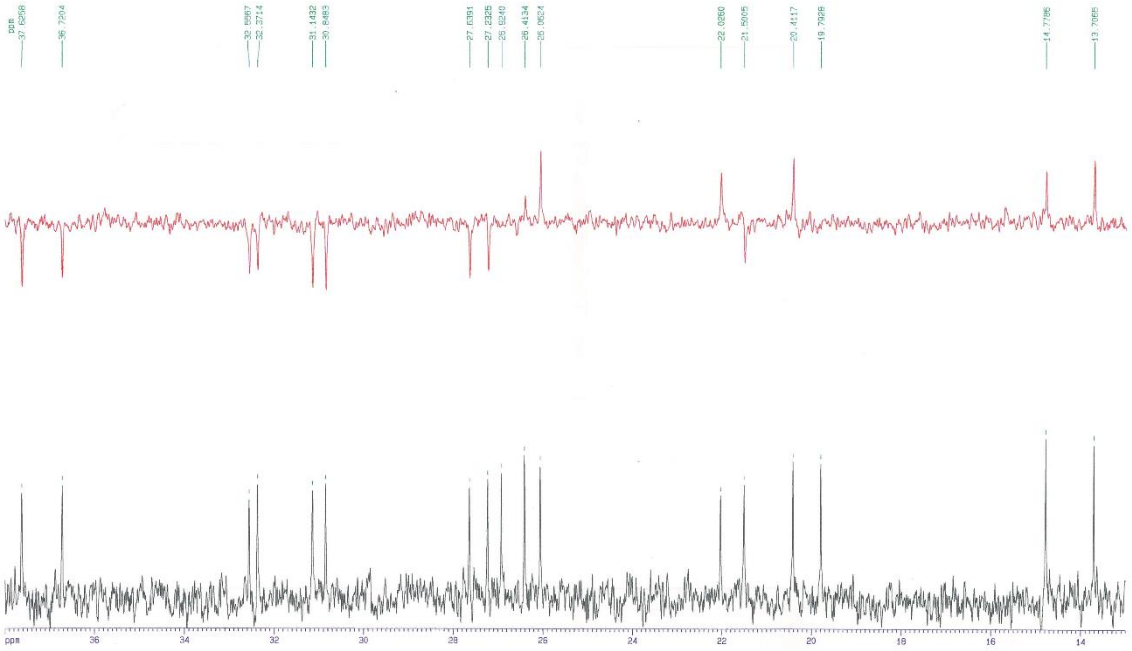

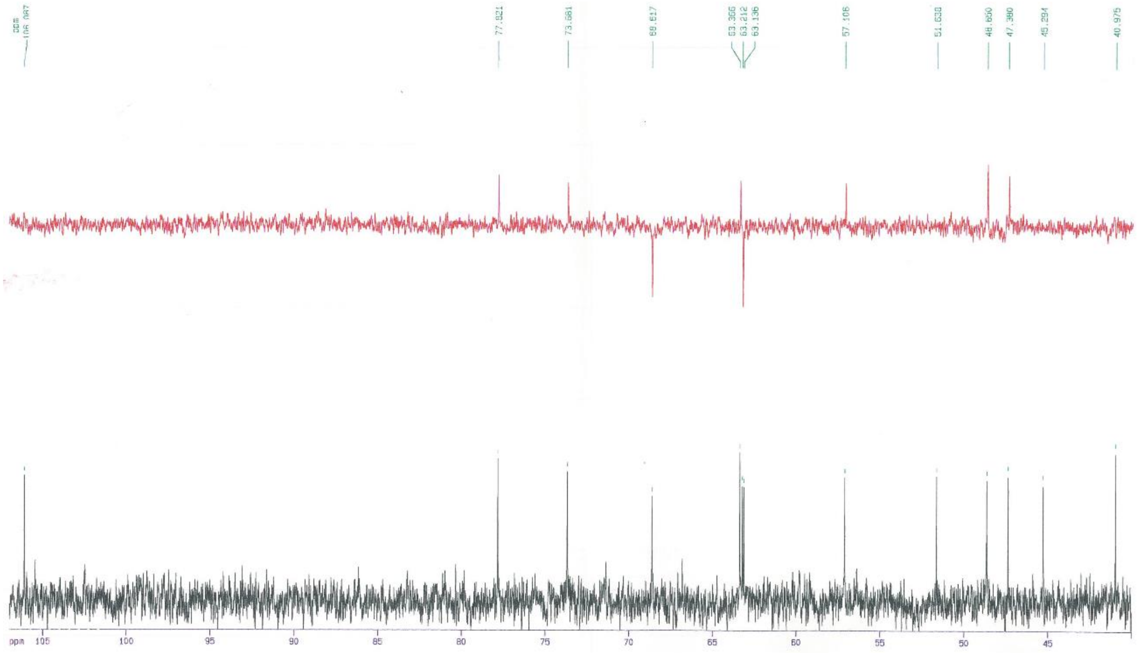

COSY spectrum of **8a** in C_5D_5N

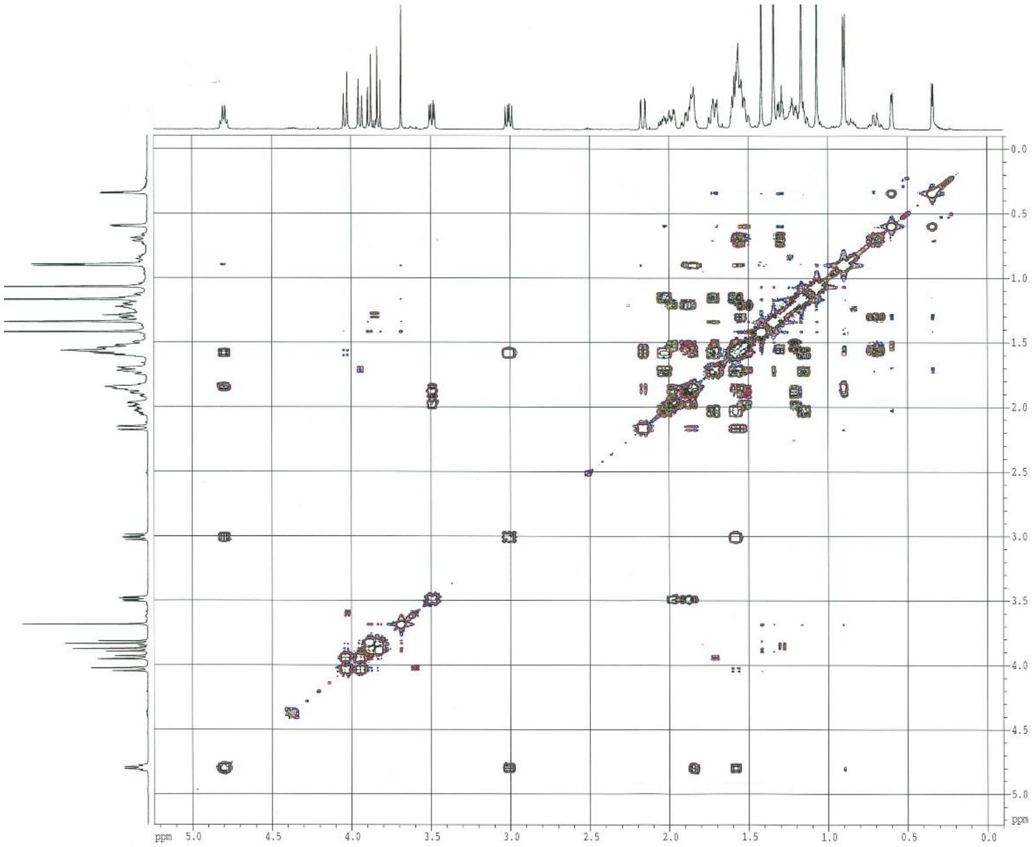

HMQC spectrum of **8a** in C_5D_5N

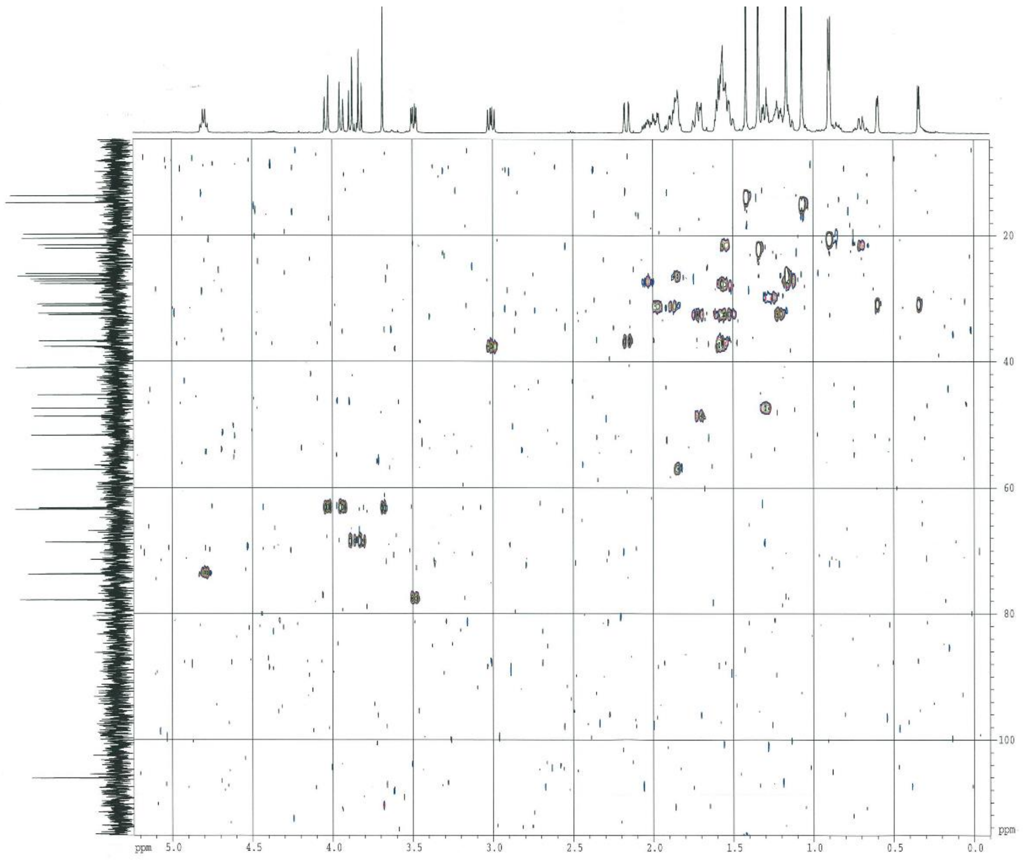

HMBC spectrum of **8a** in C_5D_5N

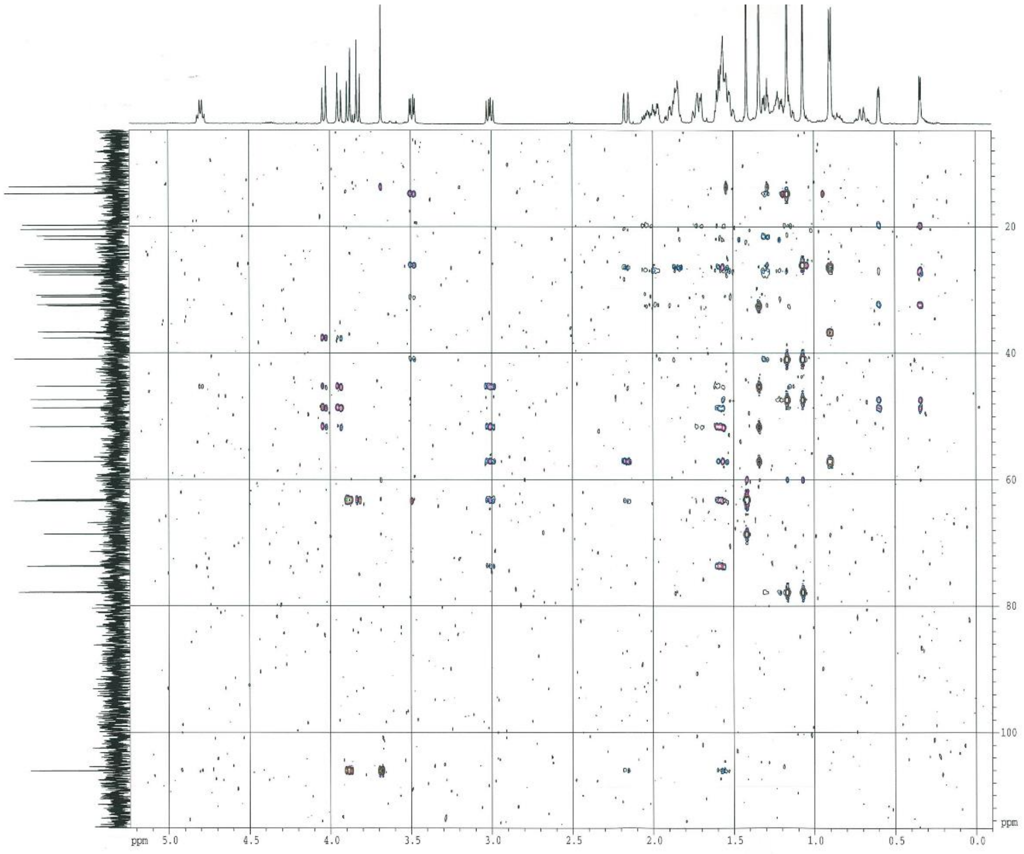

NOESY spectrum of **8a** in C_5D_5N

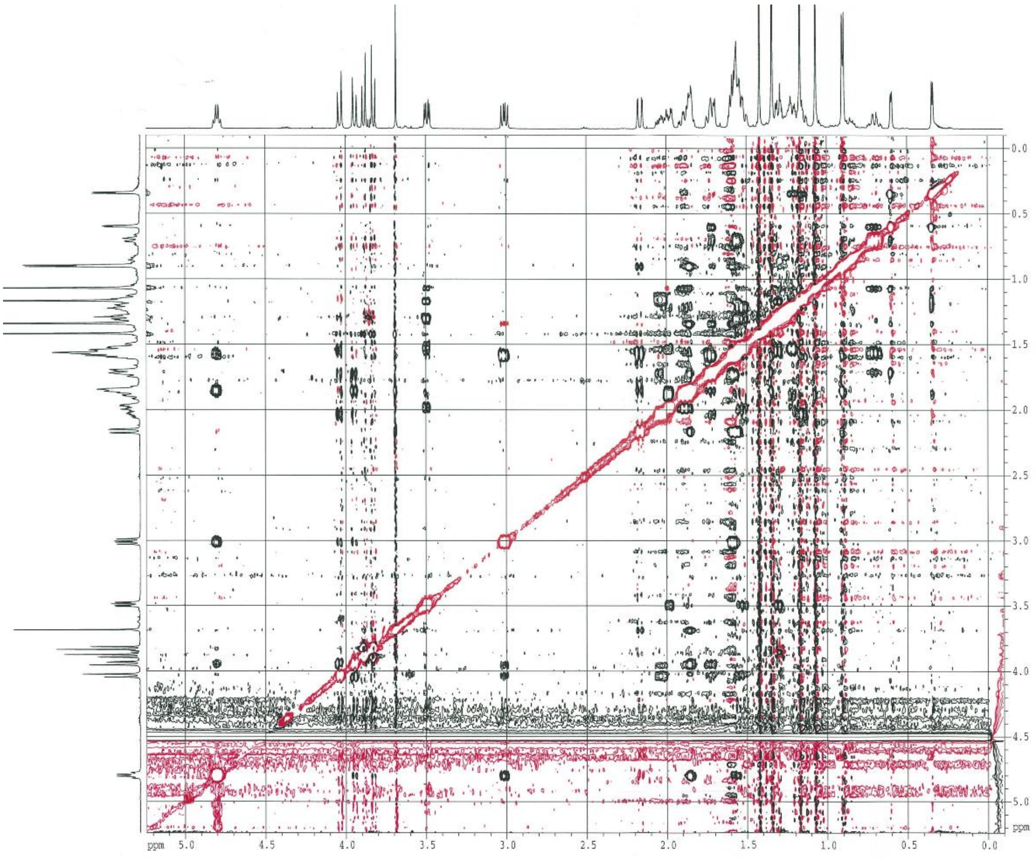

^{1}H-NMR spectrum of **9** in C_5D_5N

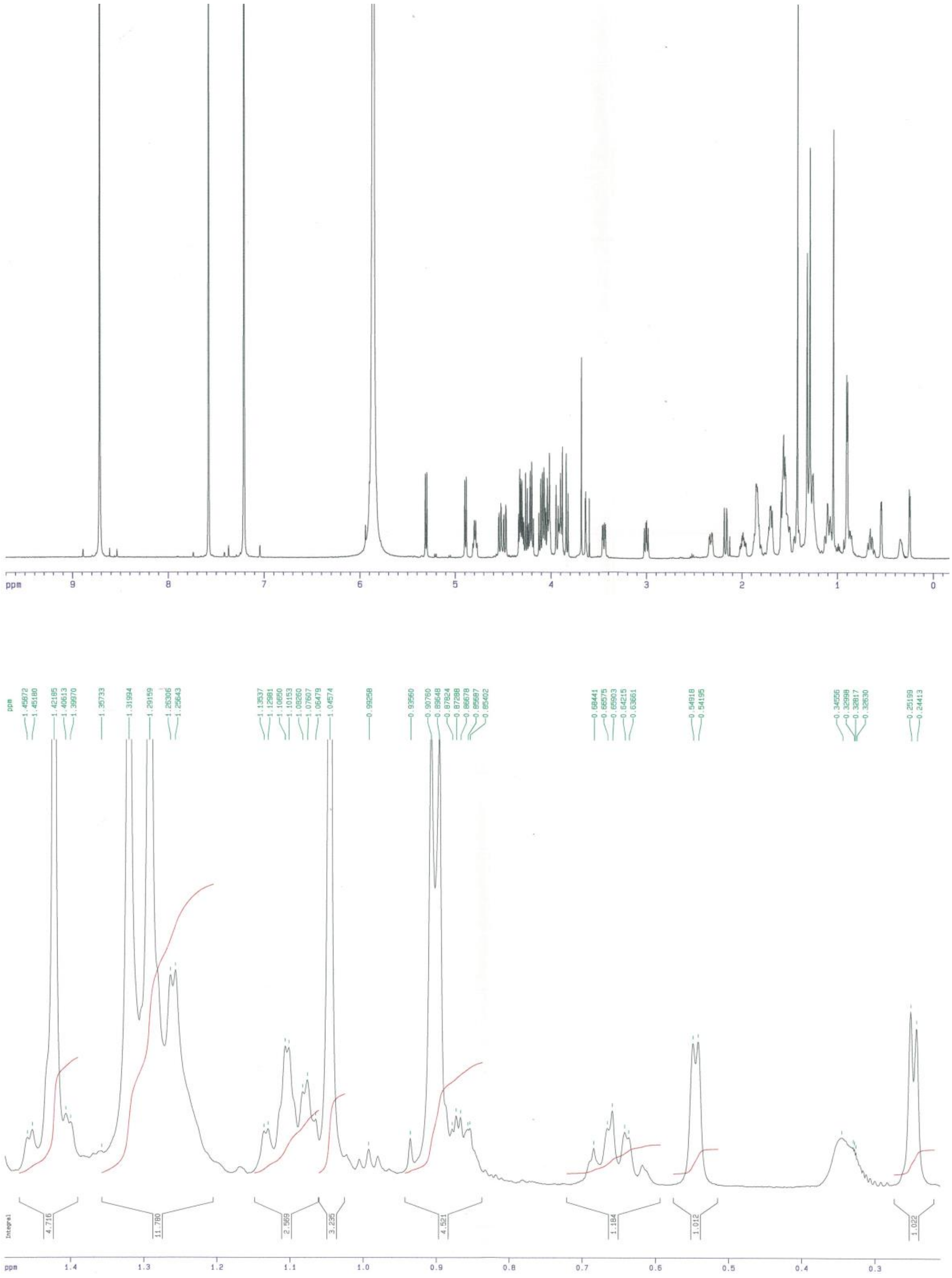

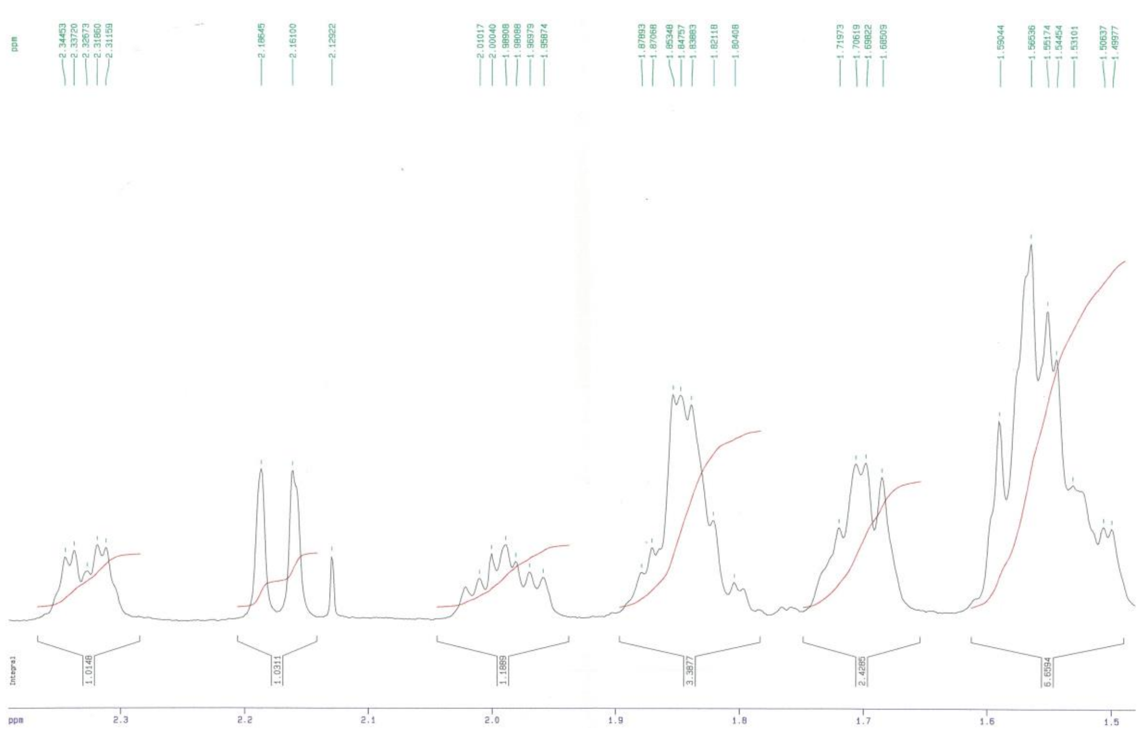

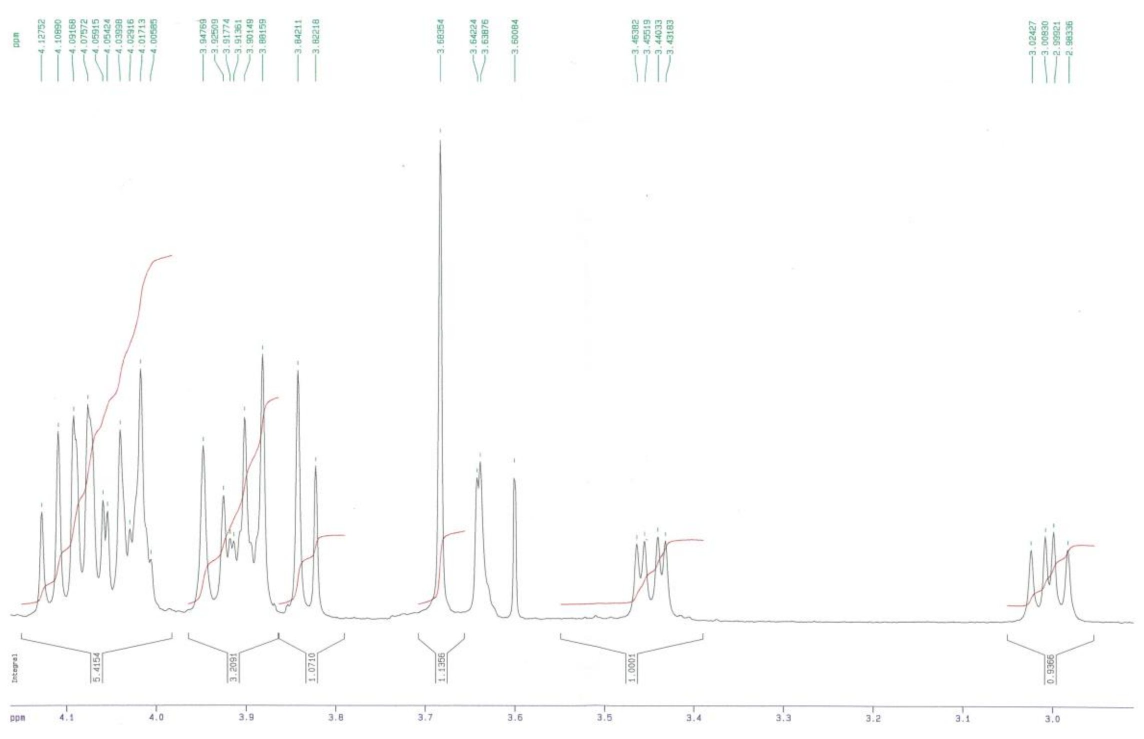

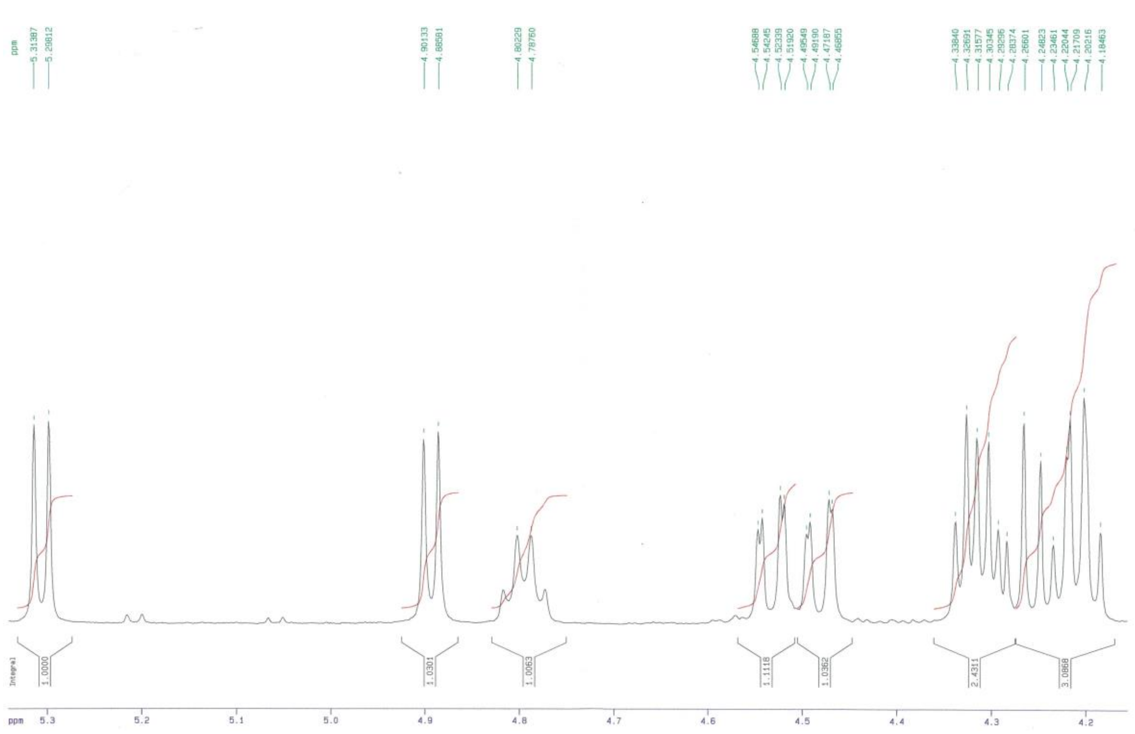

^{13}C-NMR spectrum of **9** in C_5D_5N

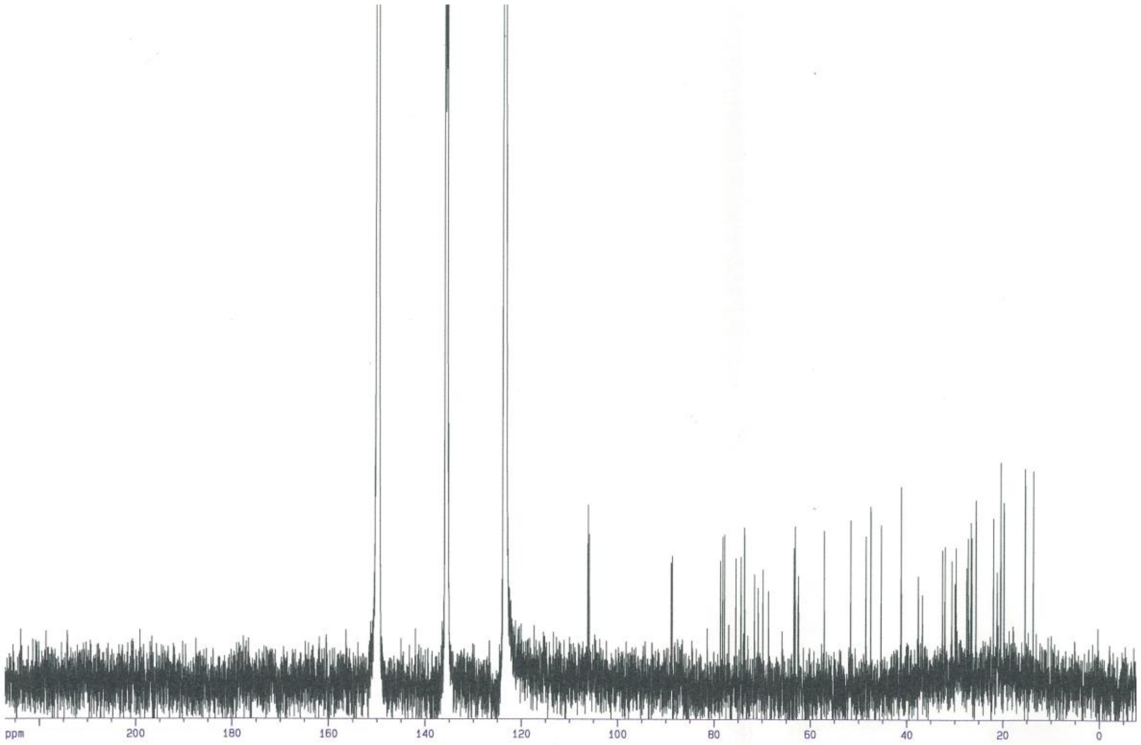

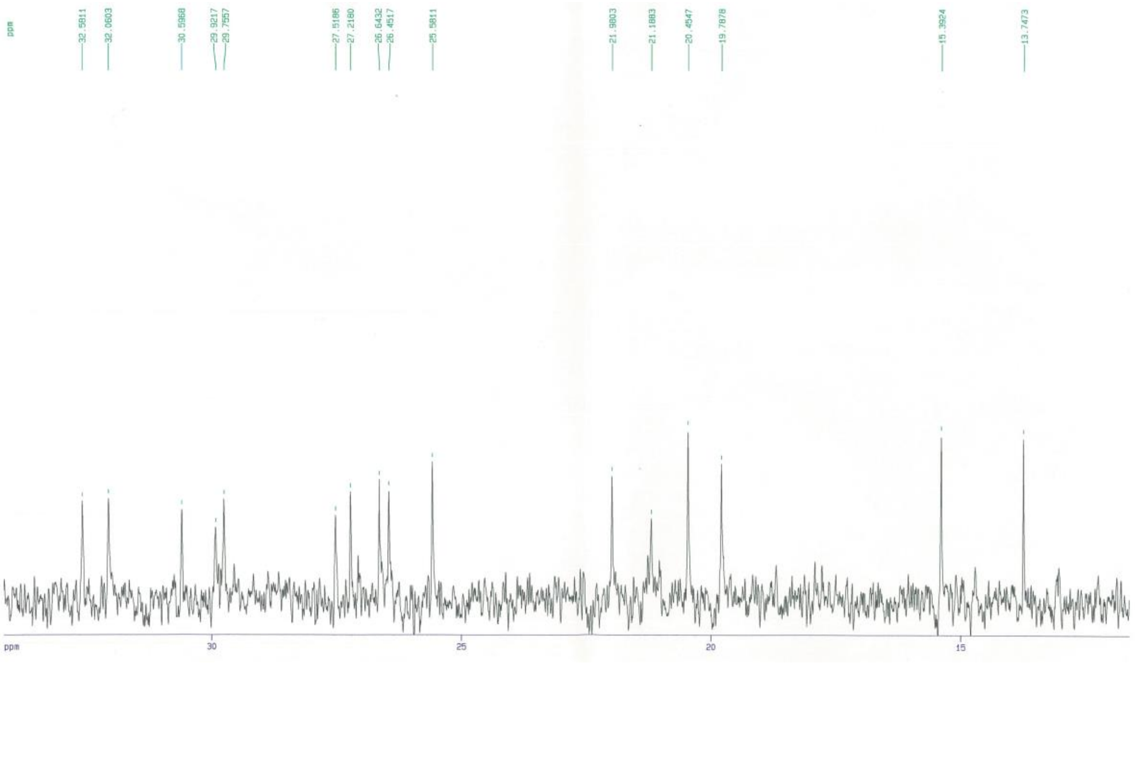

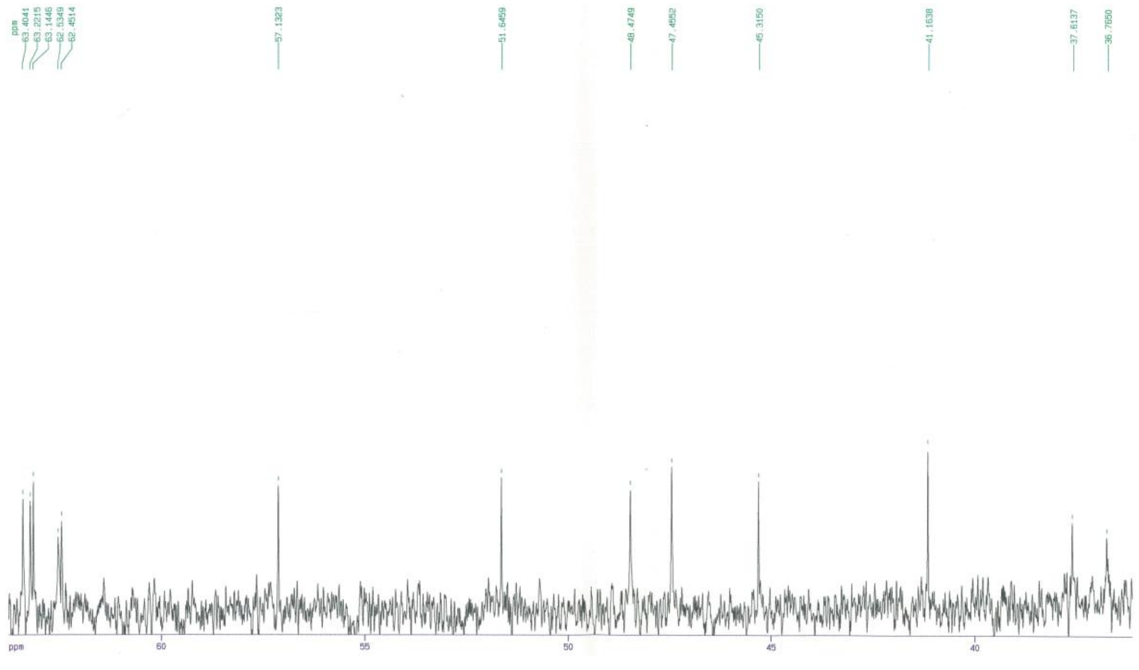

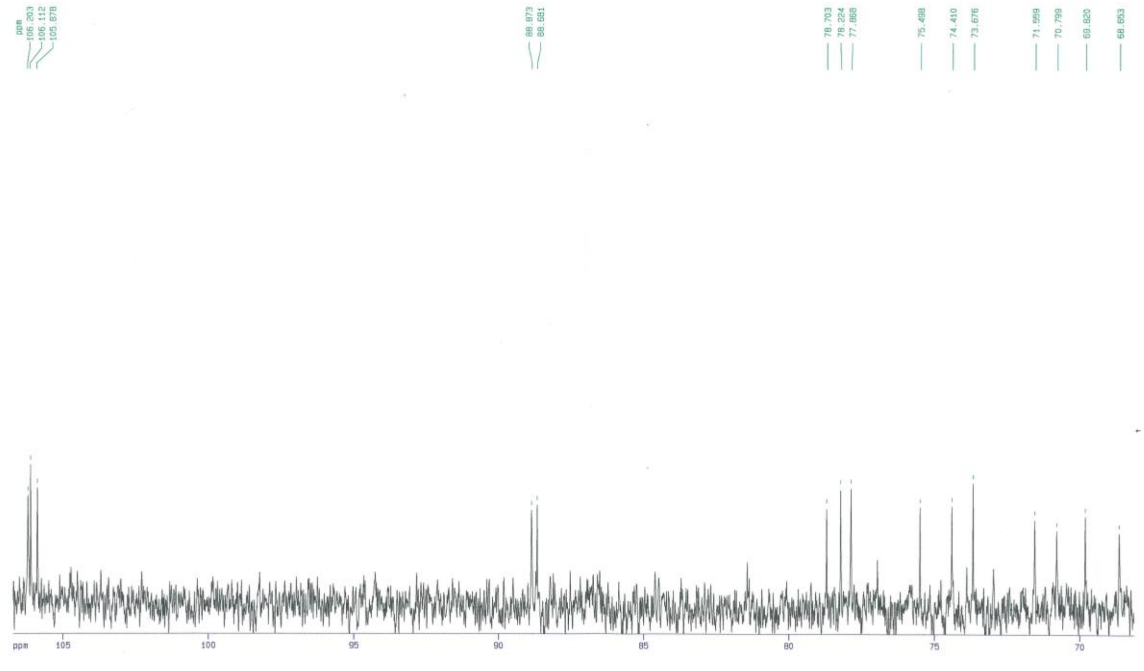

COSY spectrum of **9** in C_5D_5N

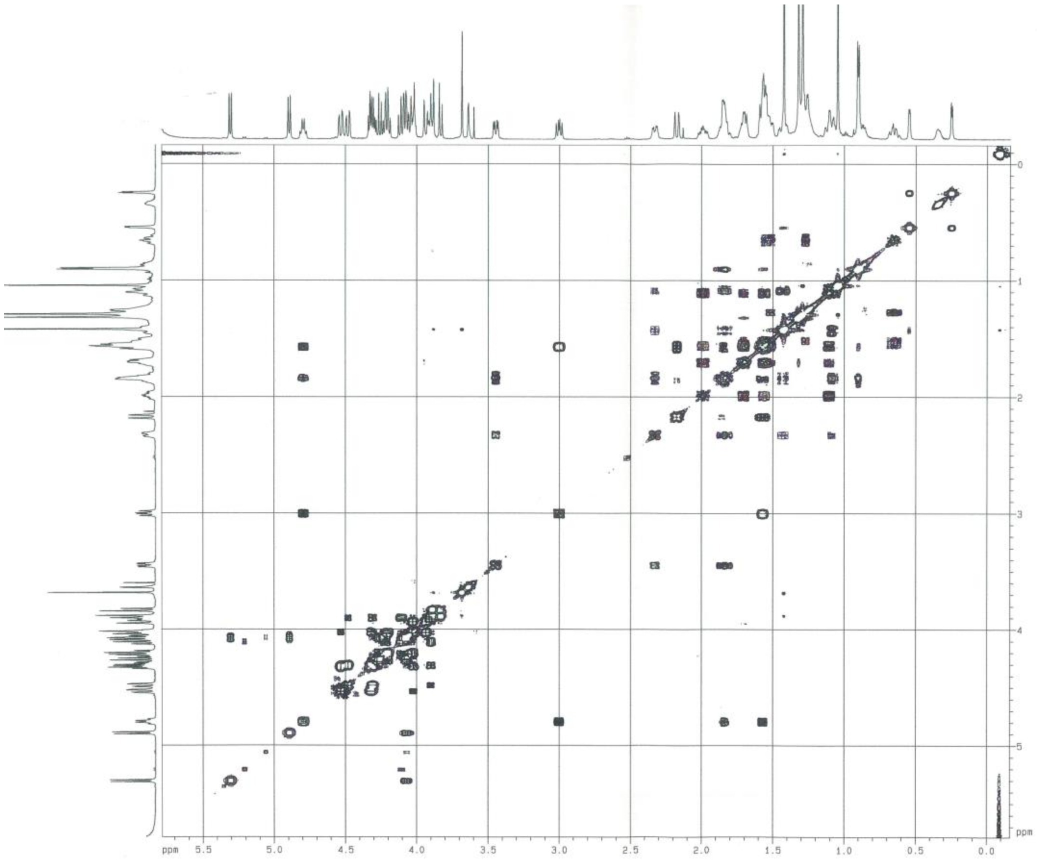

HMQC spectrum of **9** in C_5D_5N

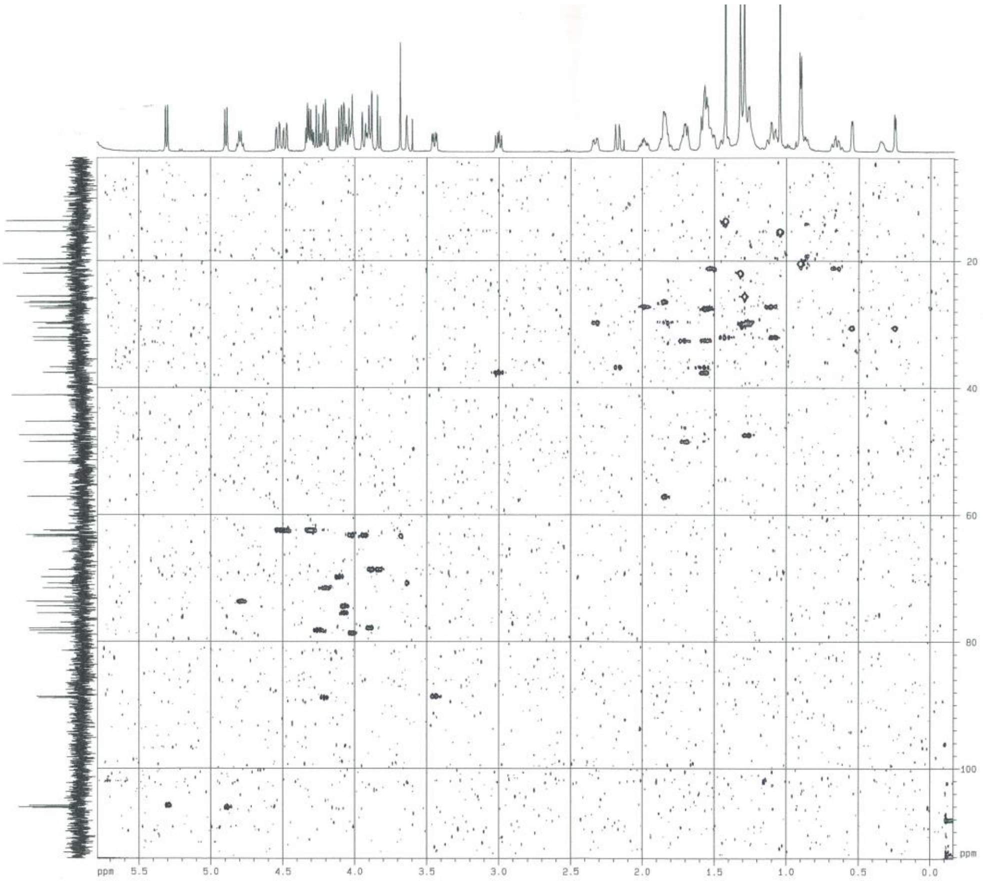

HMBC spectrum of **9** in C_5D_5N

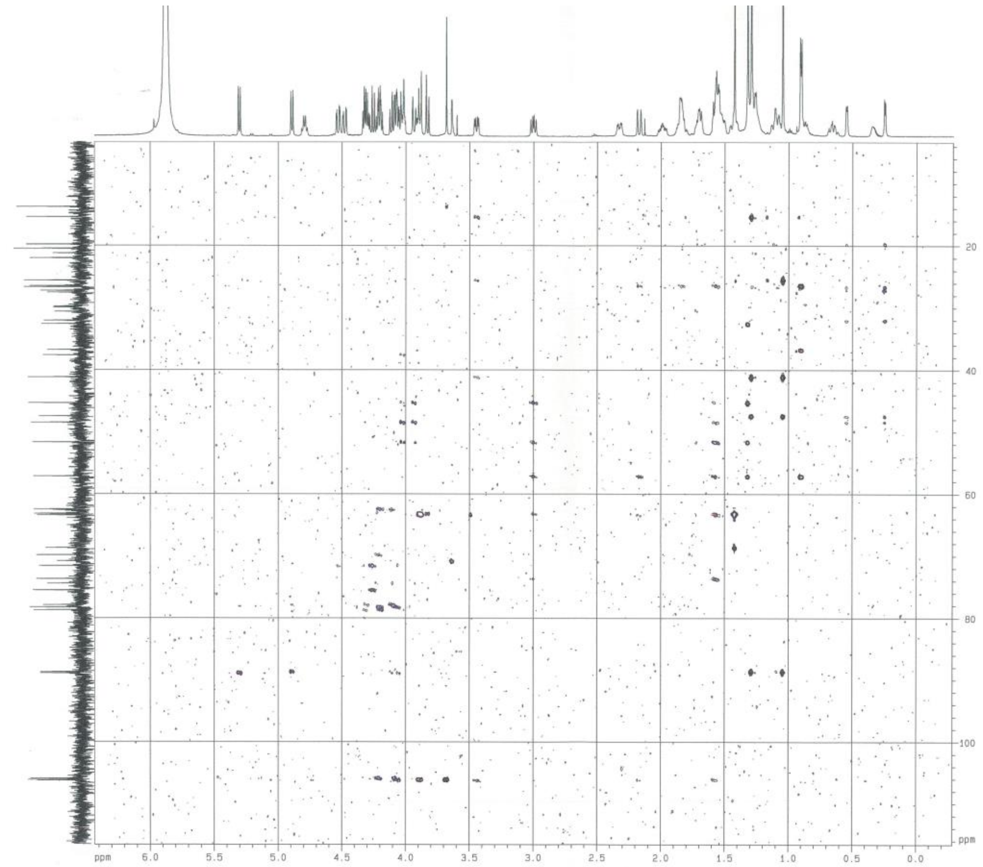

NOESY spectrum of **9** in C_5D_5N

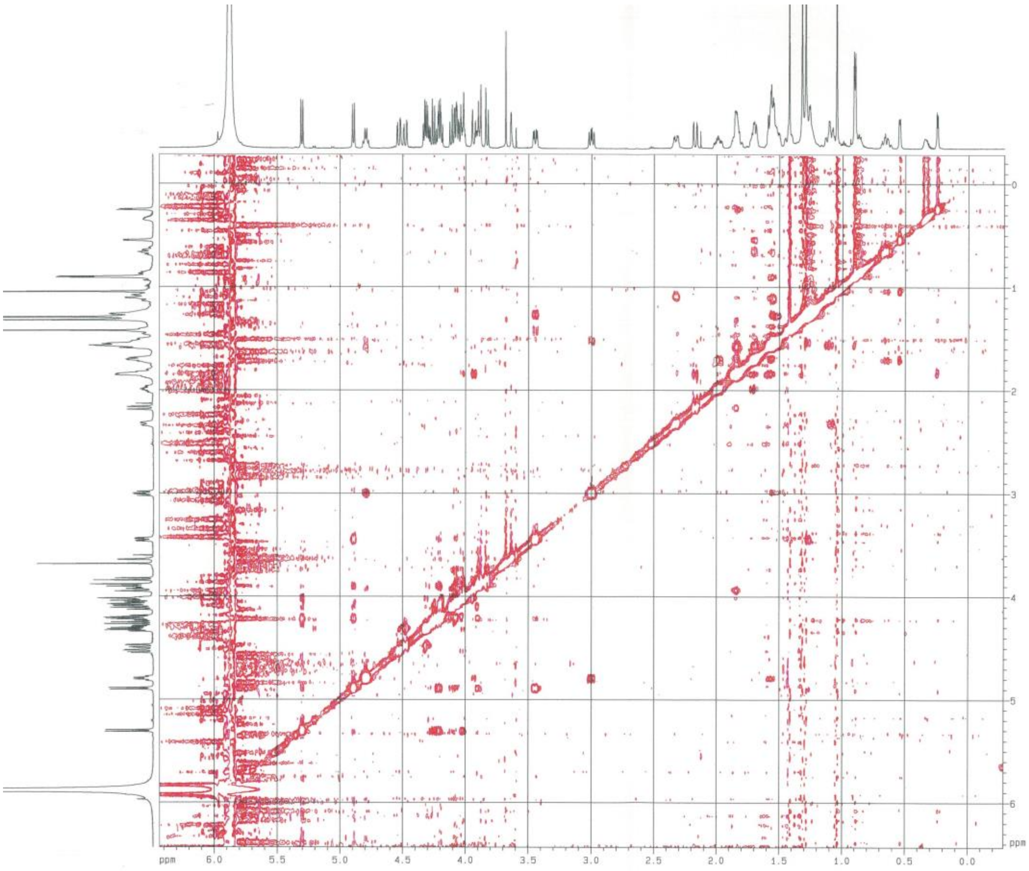

^{1}H-NMR spectrum of **10** in C_5D_5N

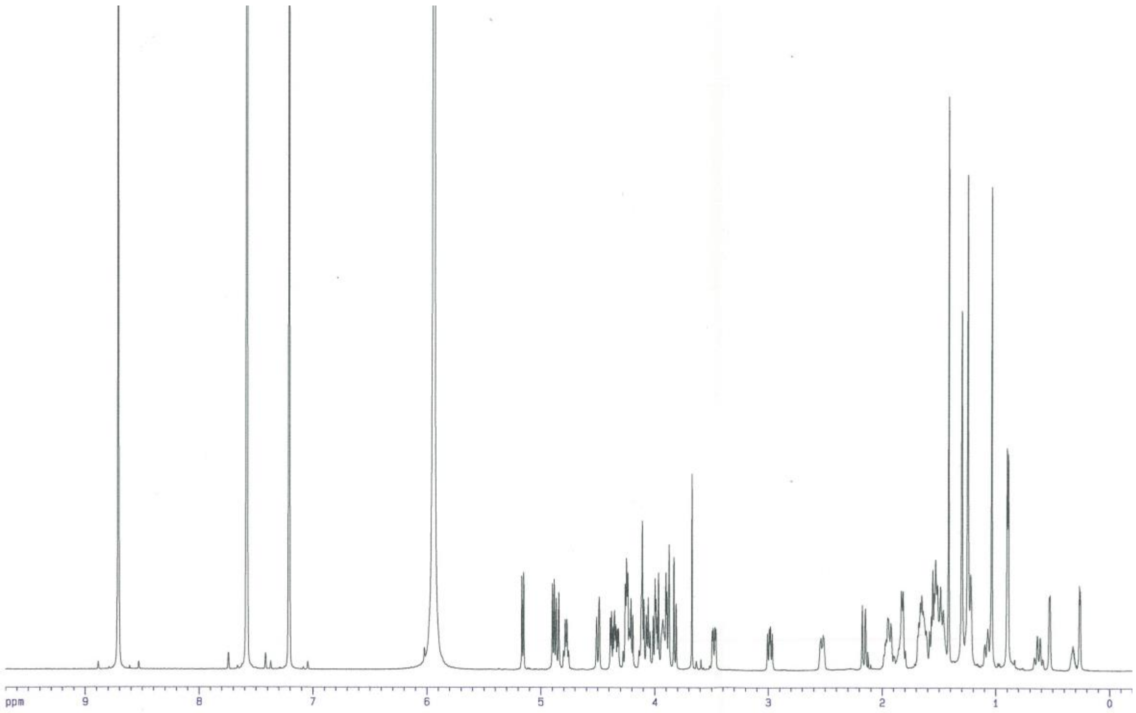

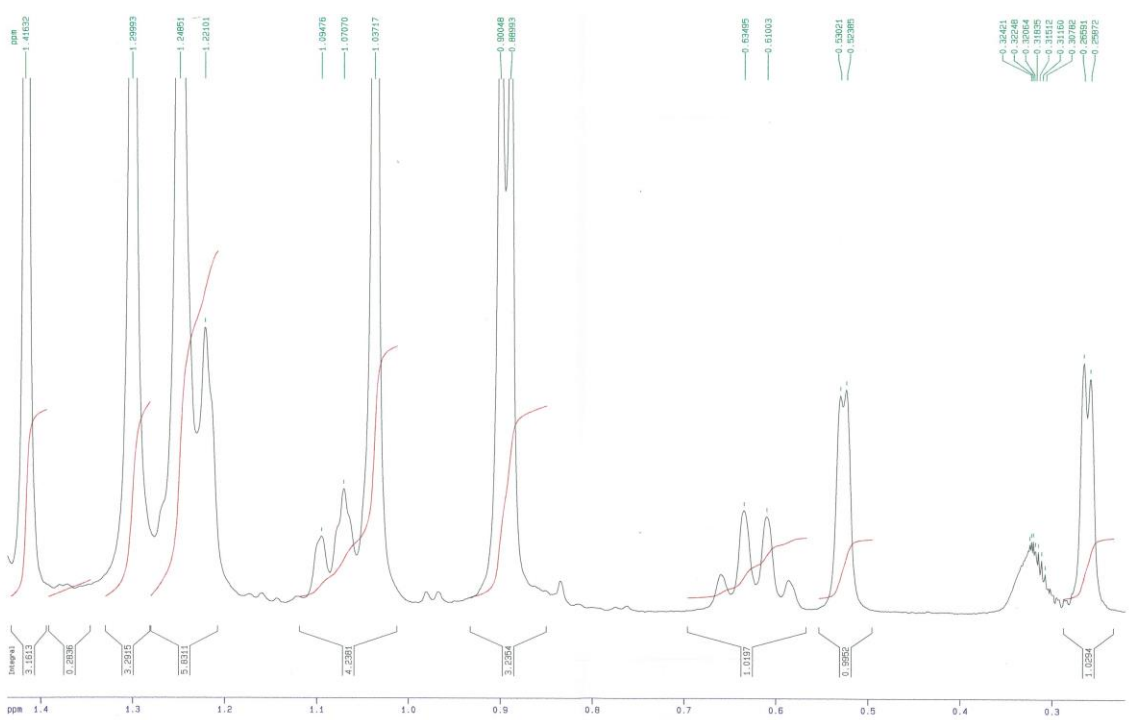

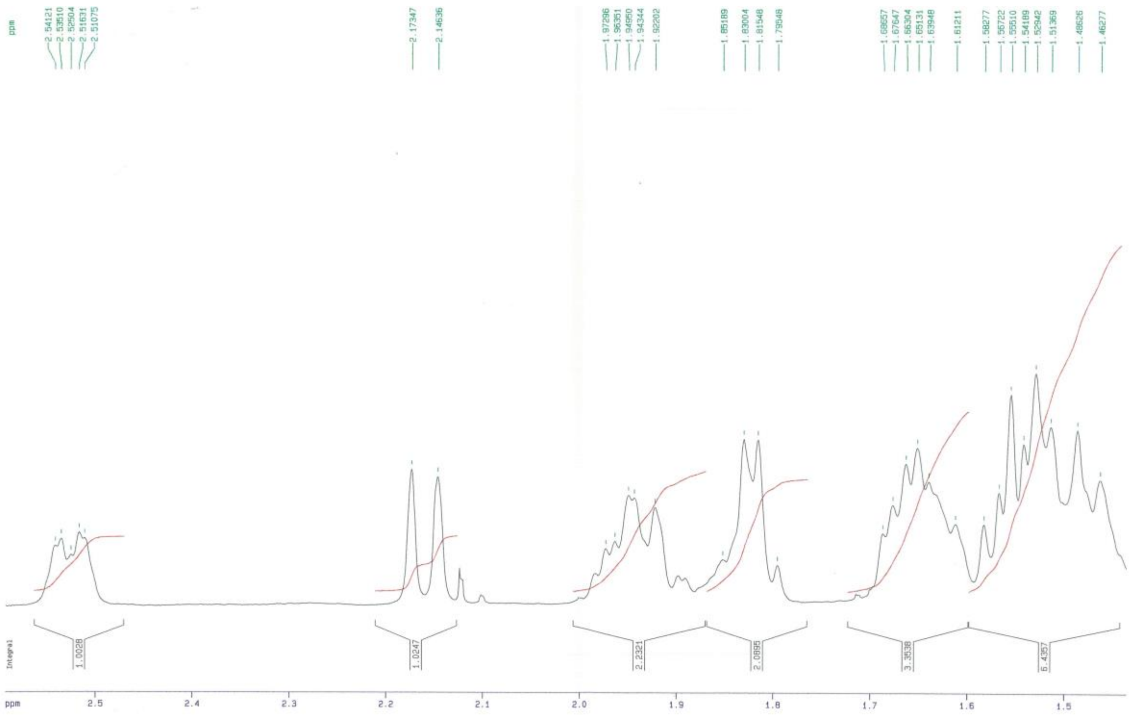

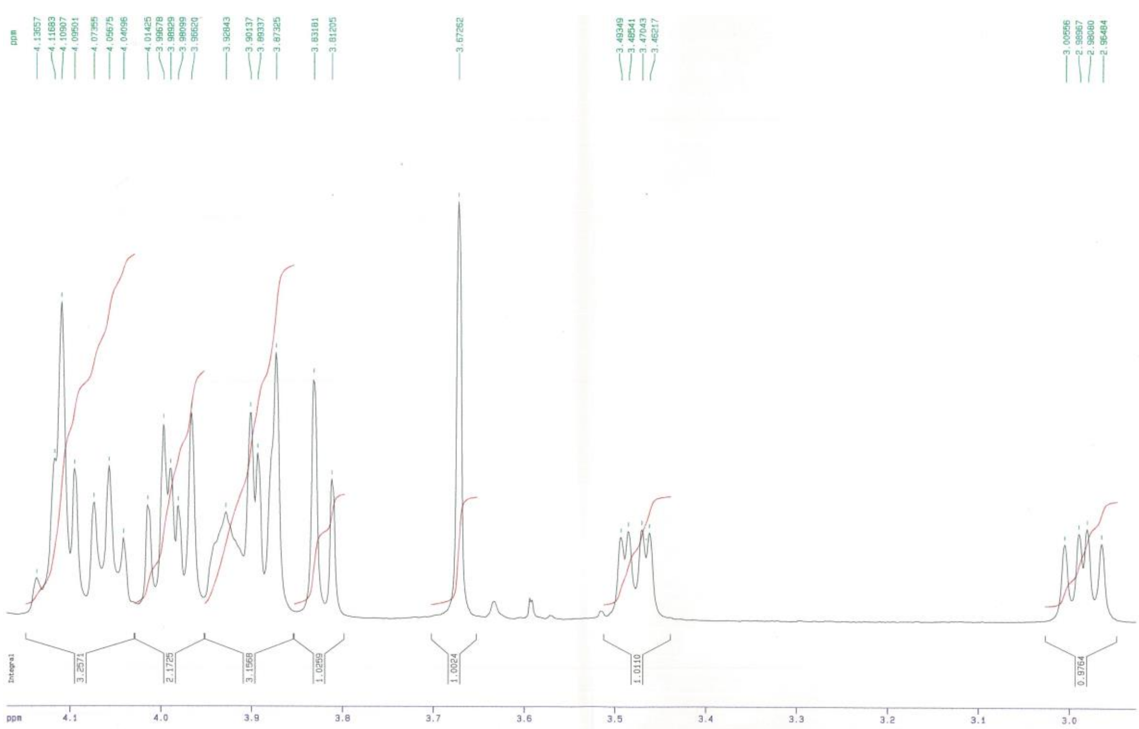

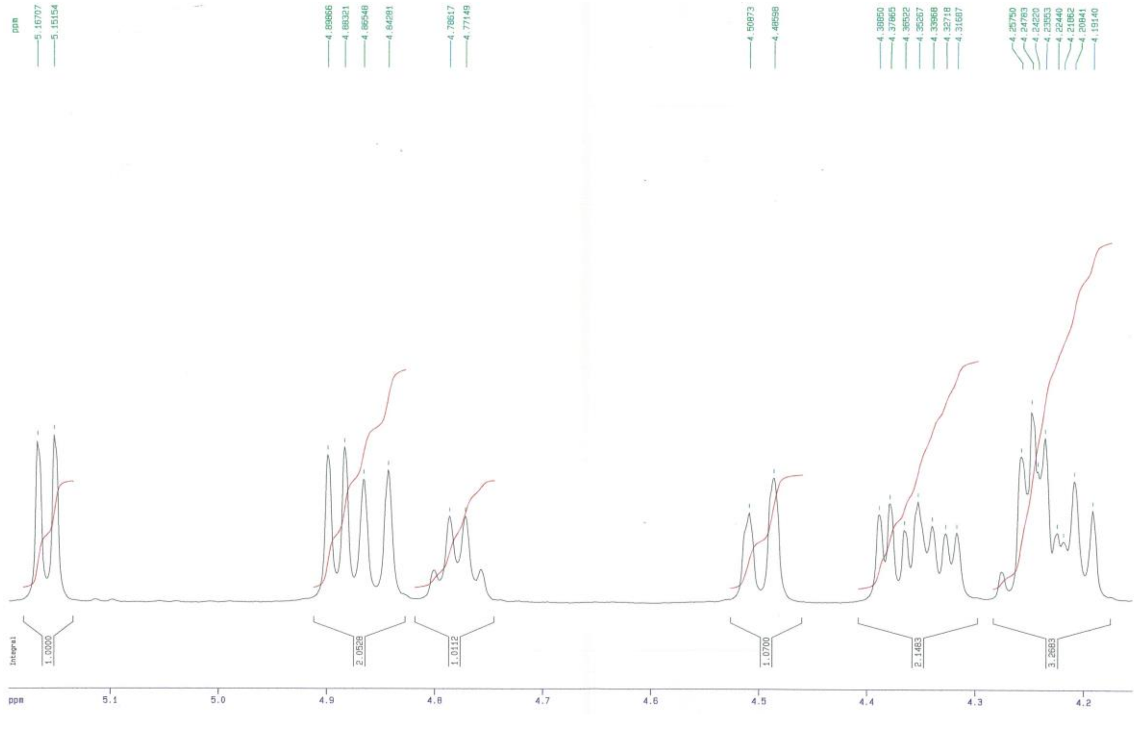

^{13}C-NMR spectrum of **10** in C_5D_5N

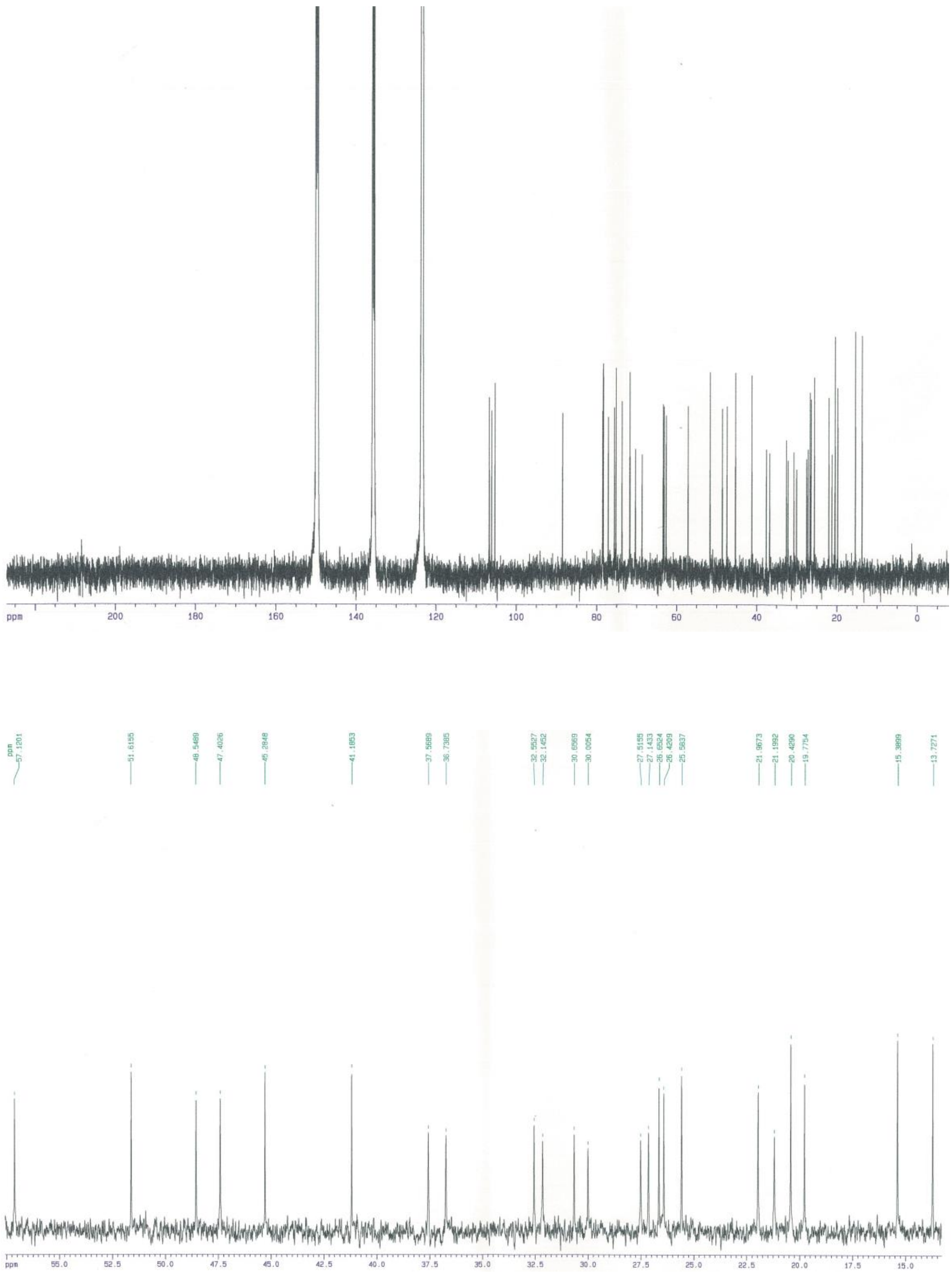

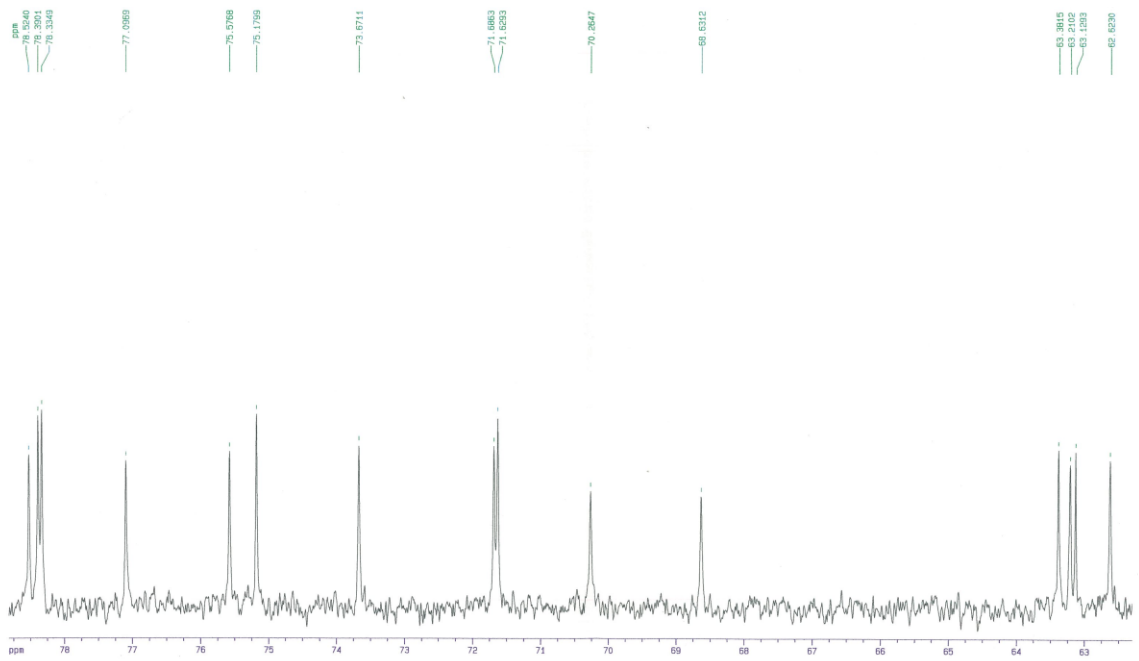

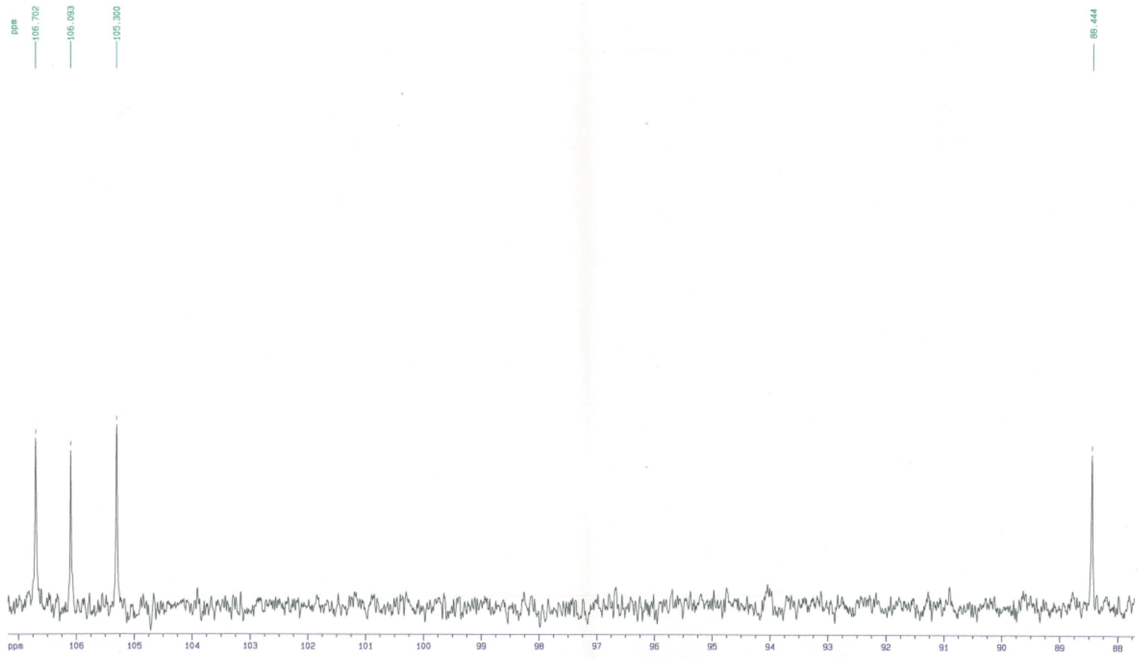

COSY spectrum of **10** in C_5D_5N

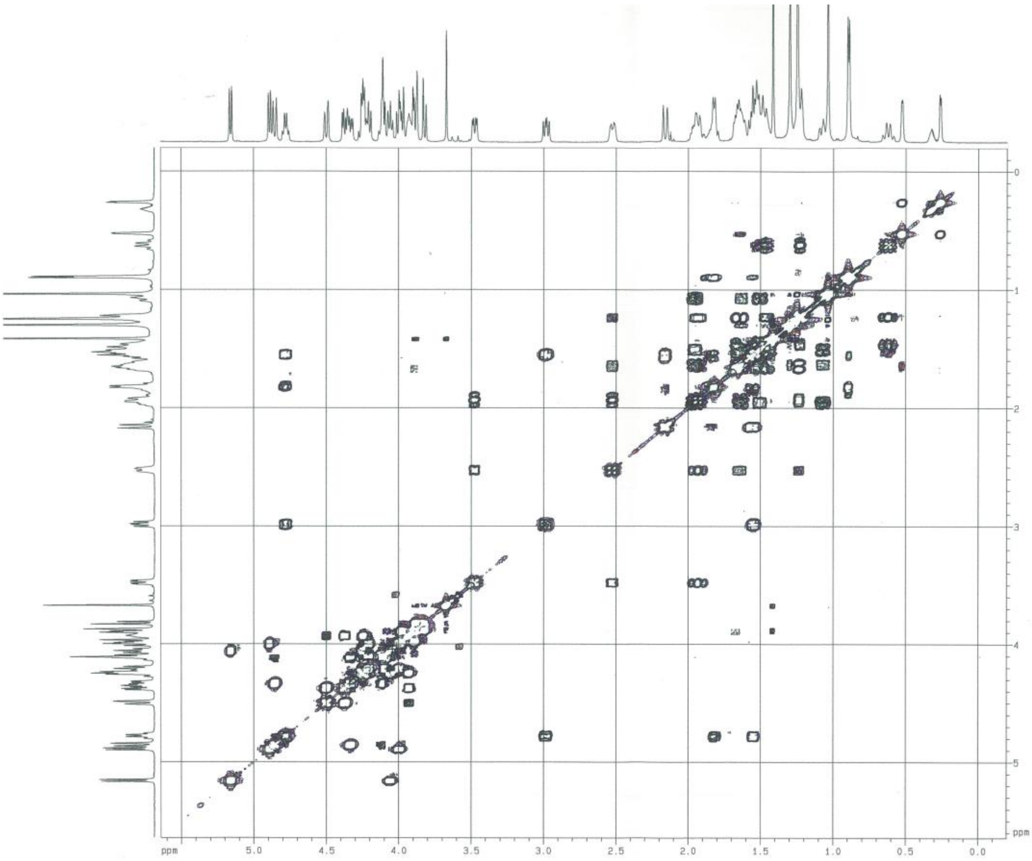

HMQC spectrum of **10** in C_5D_5N

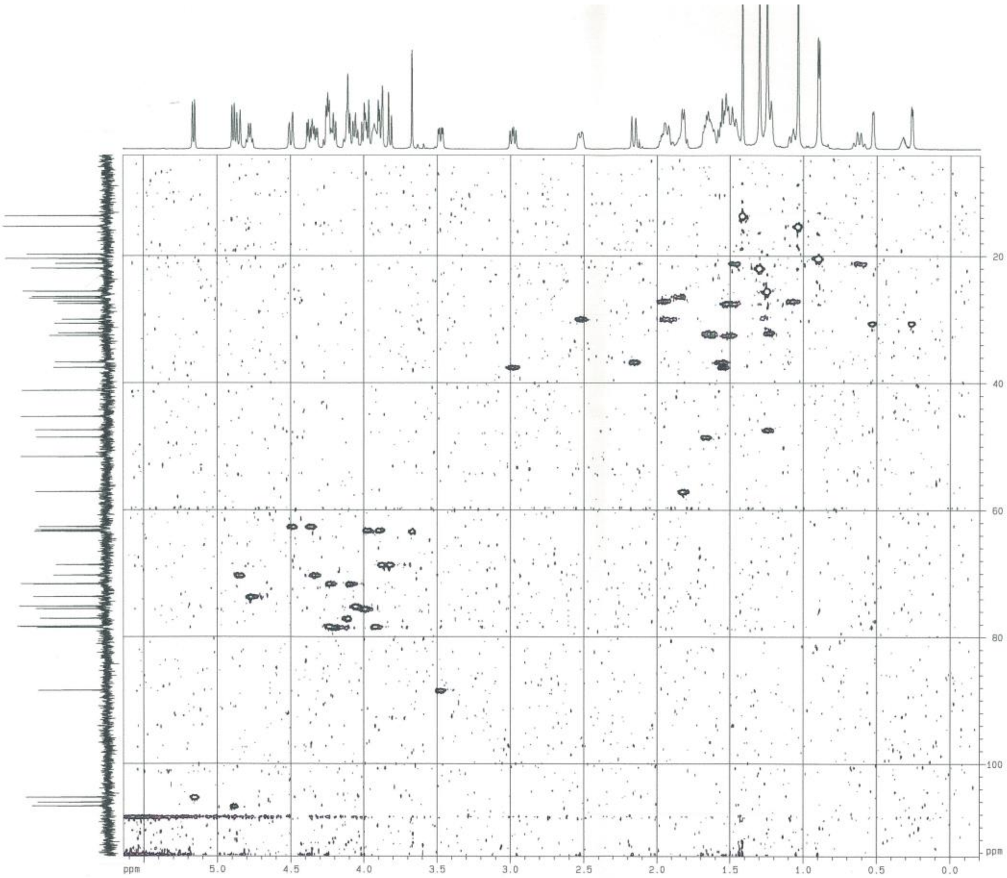

HMBC spectrum of **10** in C_5D_5N

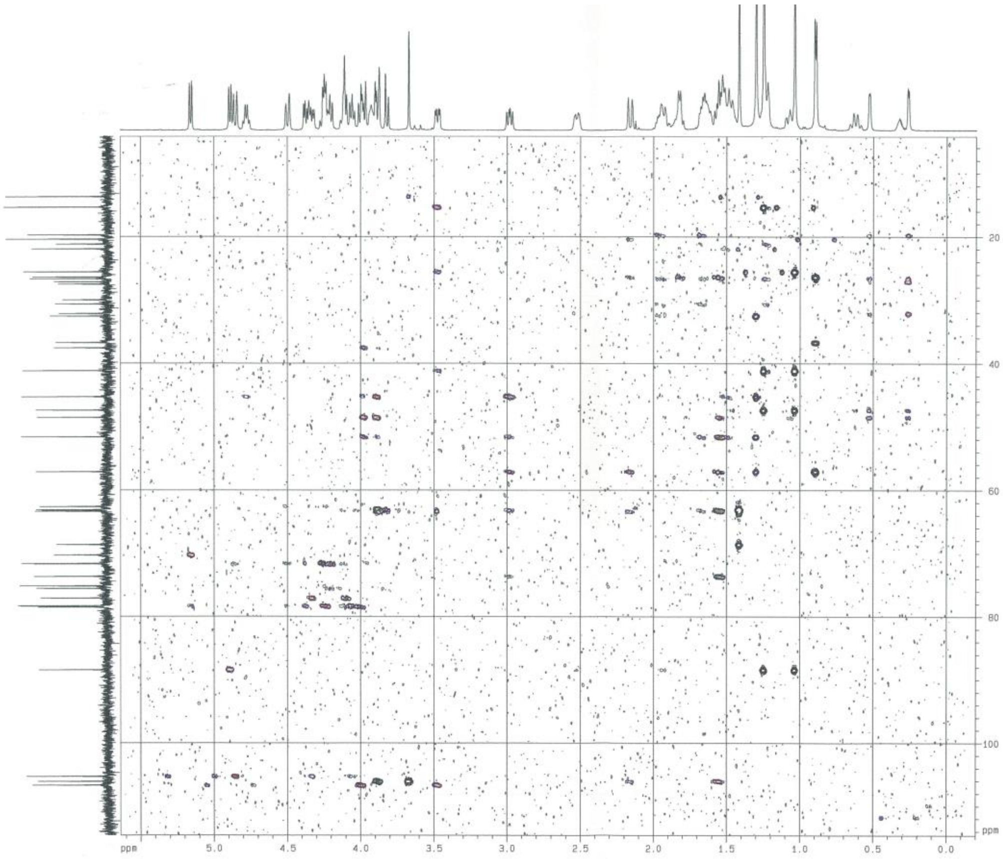

^{1}H-NMR spectrum of **11** in C_5D_5N

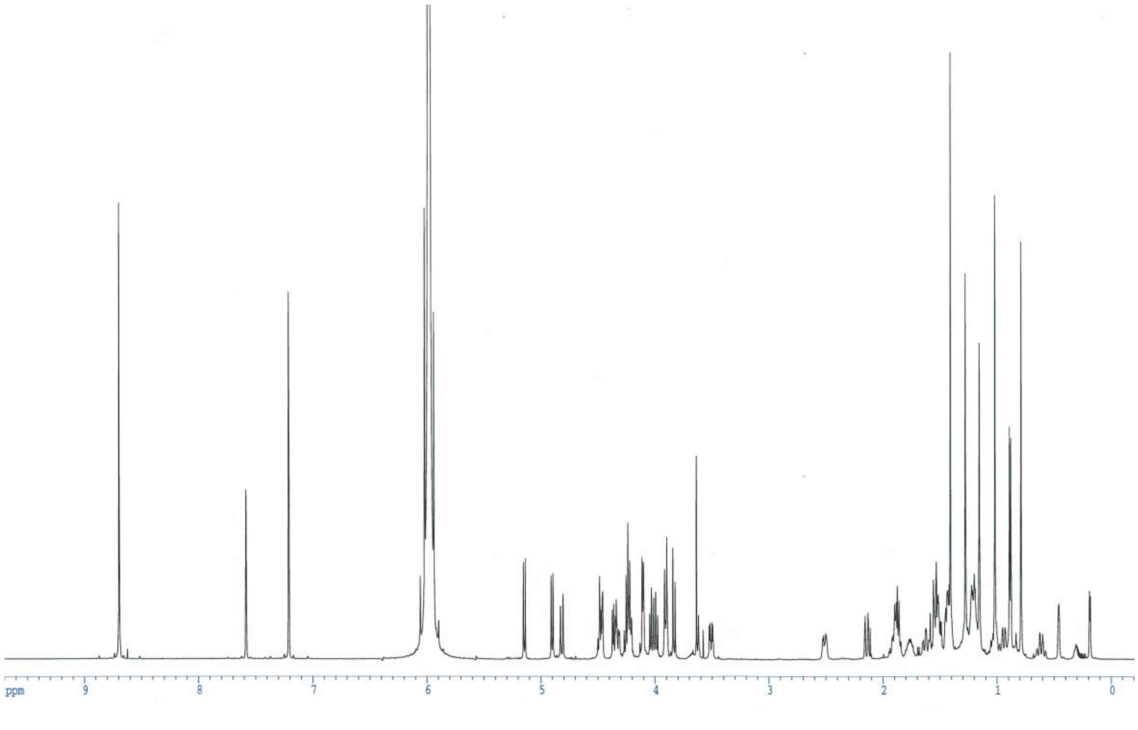

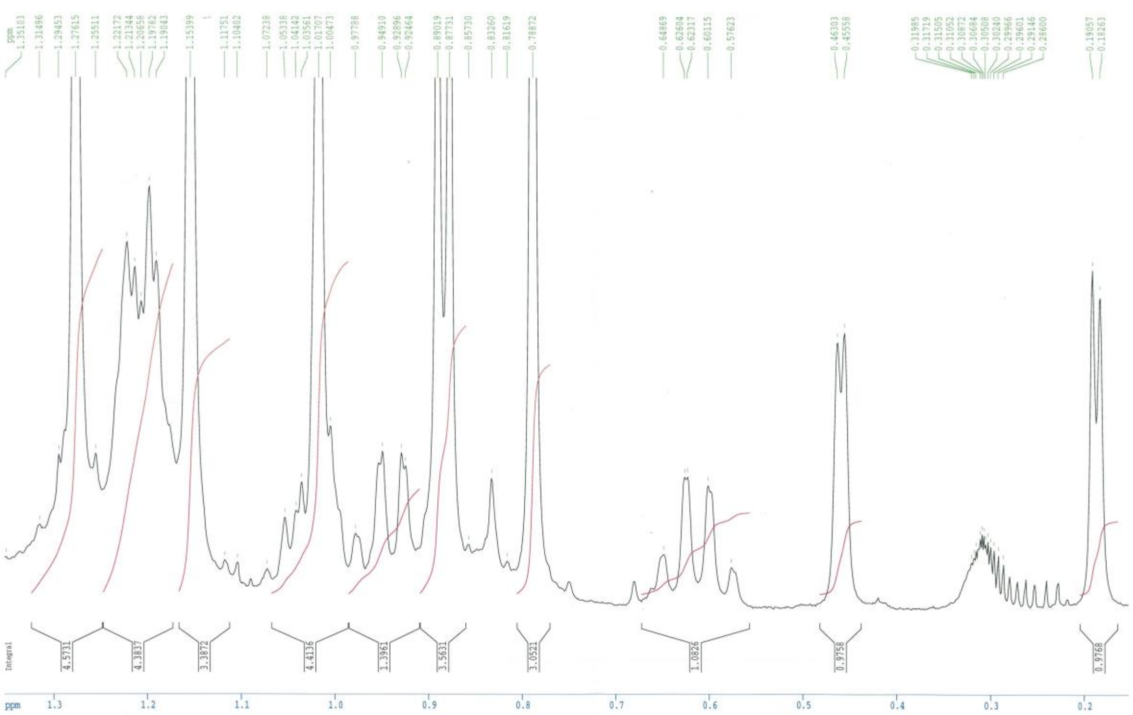
Integral
4.5731
4.3837
3.3872
4.4136
1.3961
3.5631
3.0521
1.0826
0.9758
0.9768
ppm
1.3
1.2
1.1
1.0
0.9
0.8
0.7
0.6
0.5
0.4
0.3
0.2

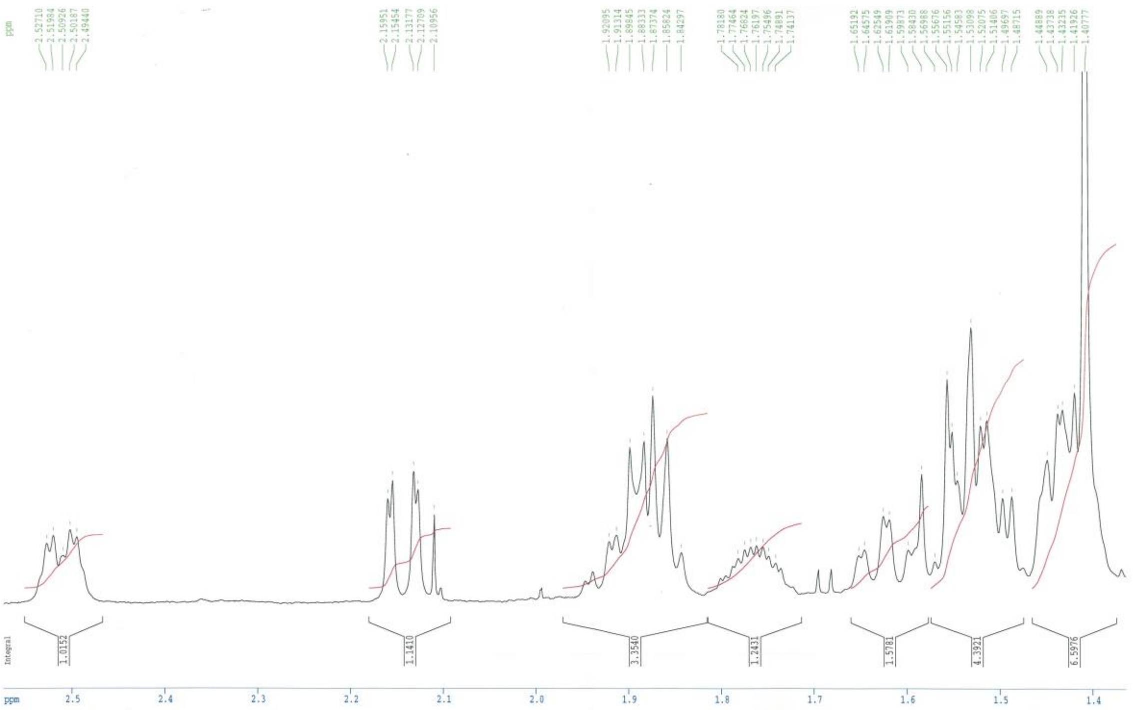
Integral
1.0152
1.1410
3.3540
1.2431
1.5781
4.3921
6.5976
ppm
2.5
2.4
2.3
2.2
2.1
2.0
1.9
1.8
1.7
1.6
1.5
1.4

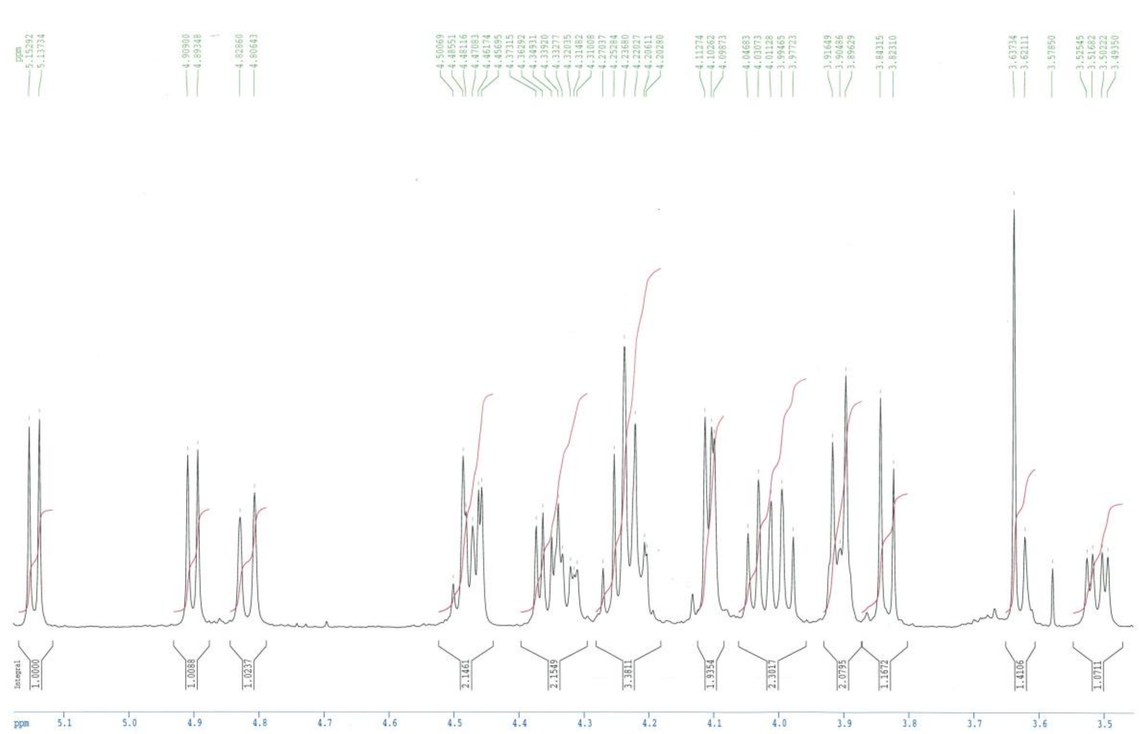

^{13}C-NMR spectrum of **11** in C_5D_5N

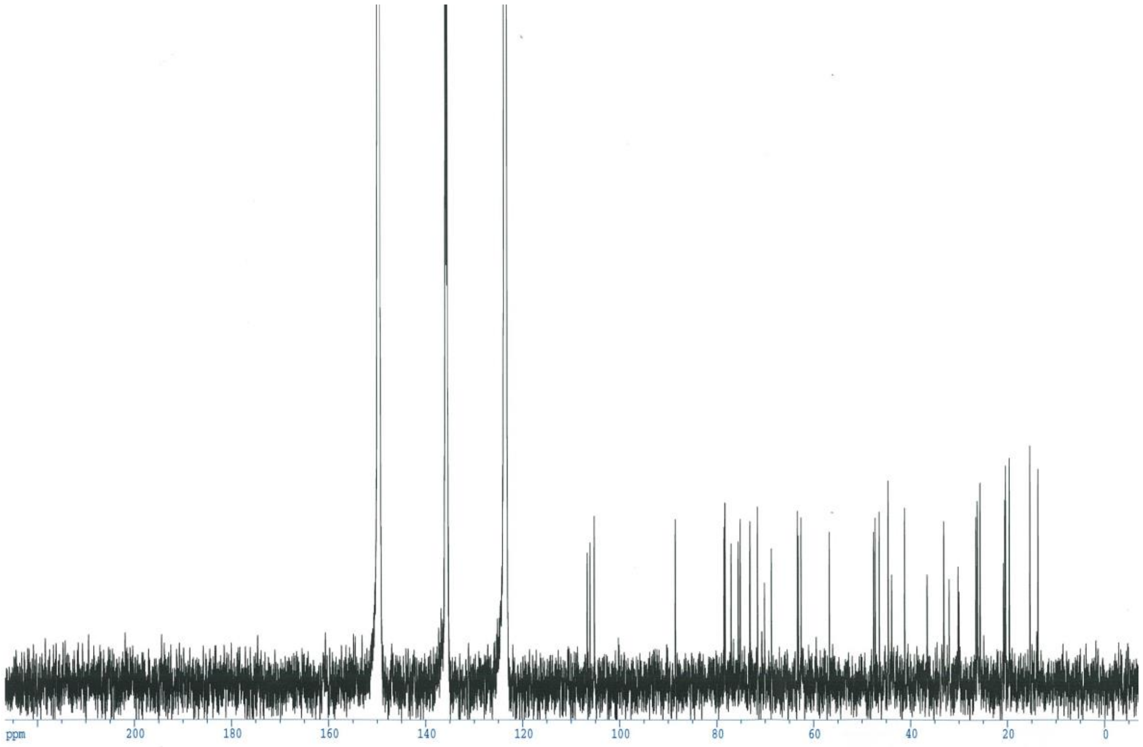

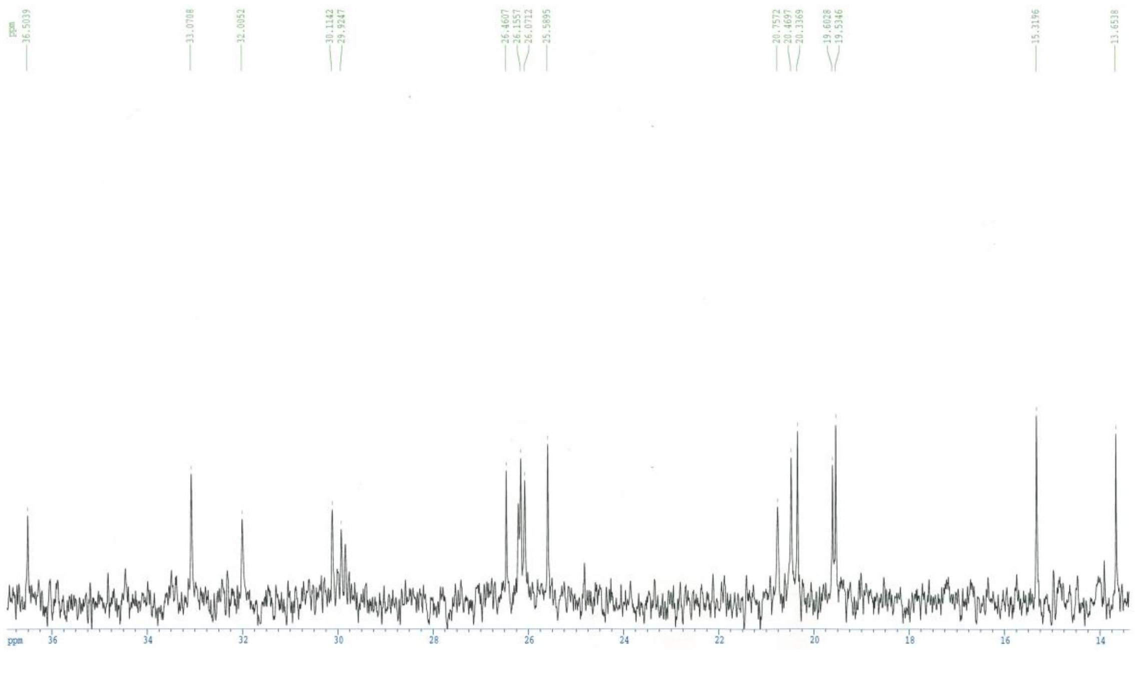

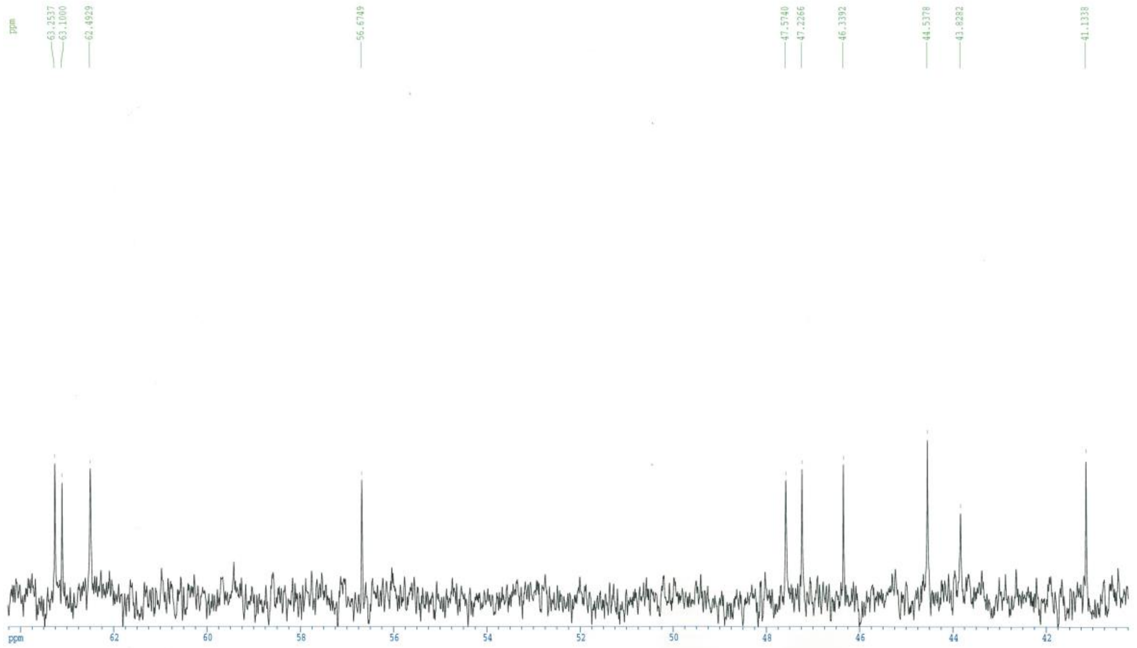
ppm
63.2537
63.1000
62.4929
56.6749
47.5740
47.2266
46.3392
44.5378
43.8282
41.1338
ppm 62 60 58 56 54 52 50 48 46 44 42

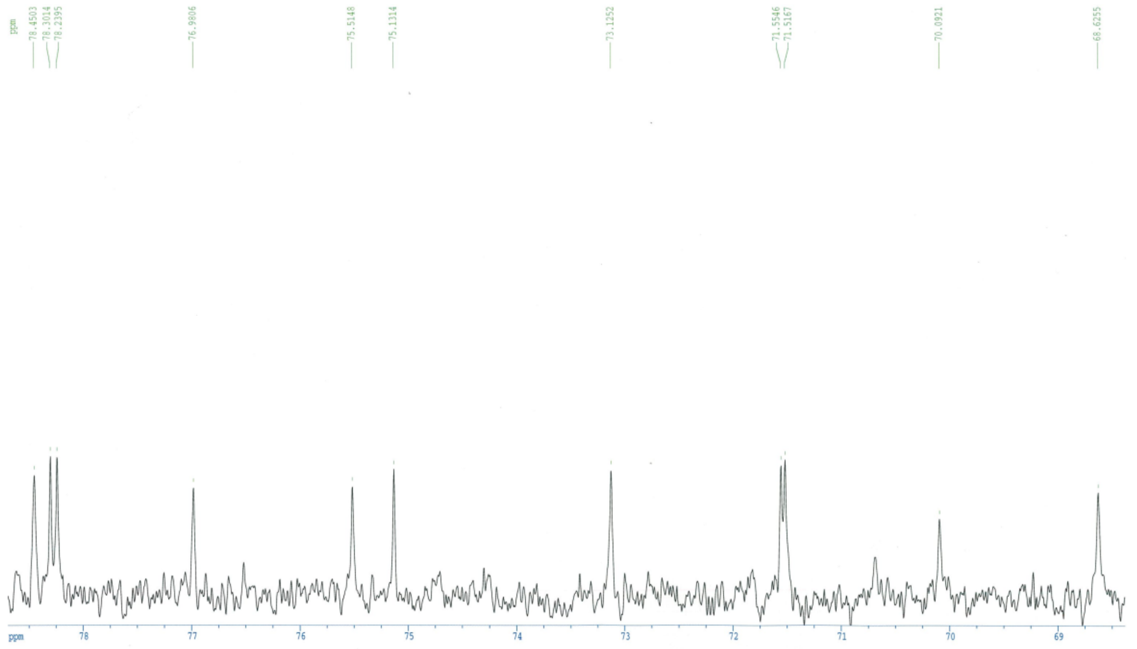
ppm
78.4503
78.3014
78.2395
76.9806
75.5148
75.1314
73.1252
71.5546
71.5167
70.0921
68.6255
ppm 78 77 76 75 74 73 72 71 70 69

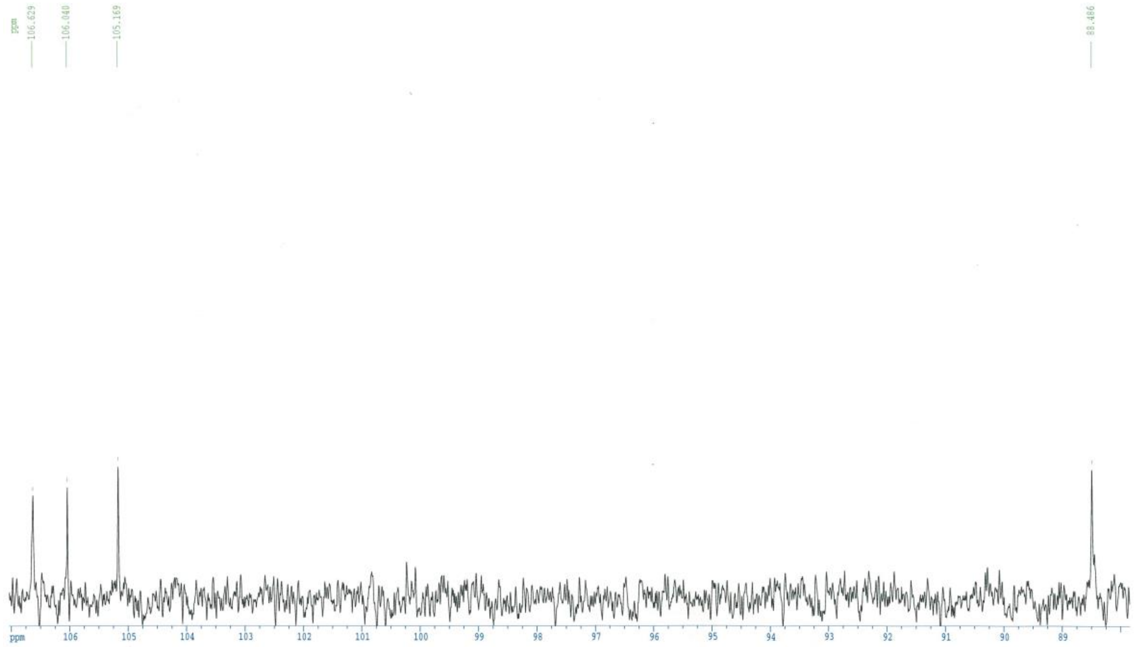

COSY spectrum of **11** in C_5D_5N

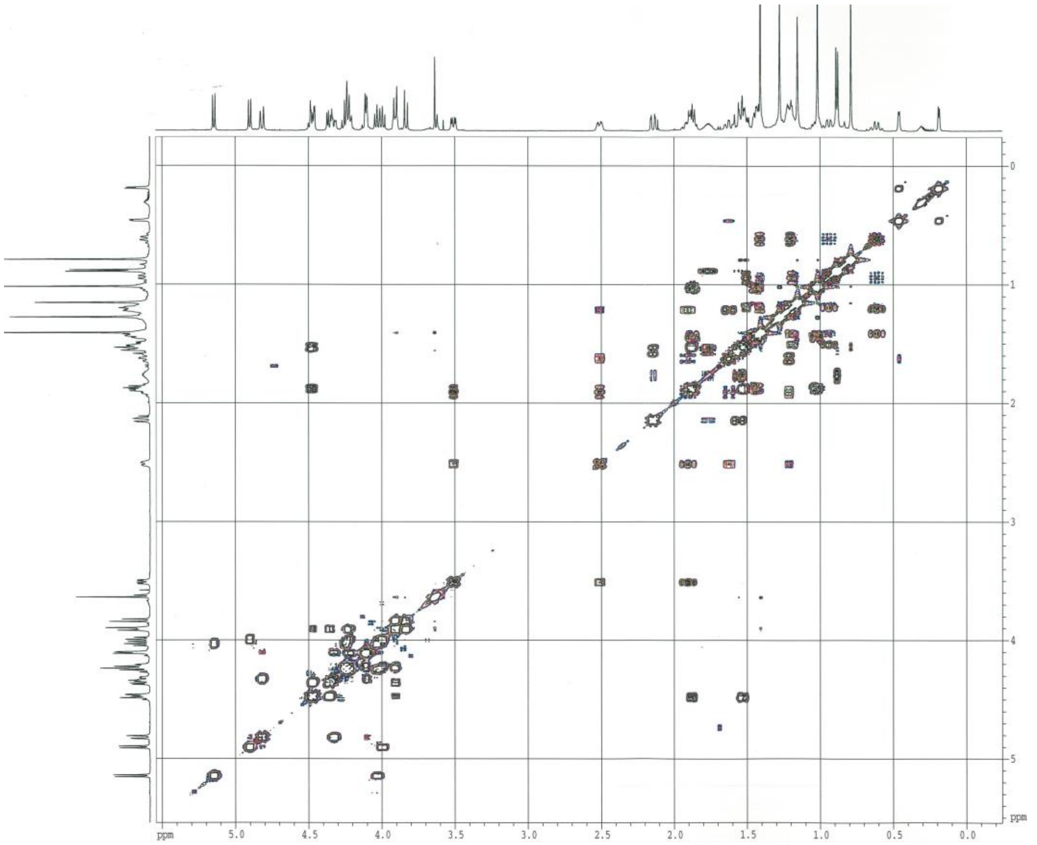

HMQC spectrum of **11** in C_5D_5N

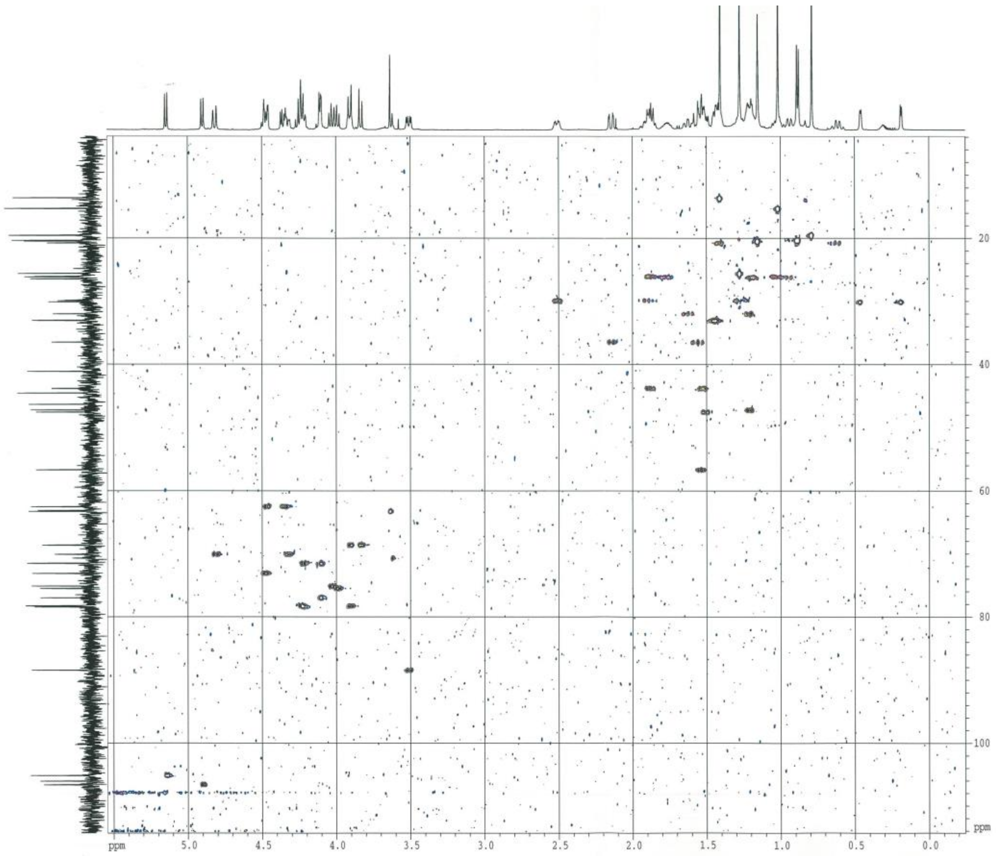

HMBC spectrum of **11** in C_5D_5N

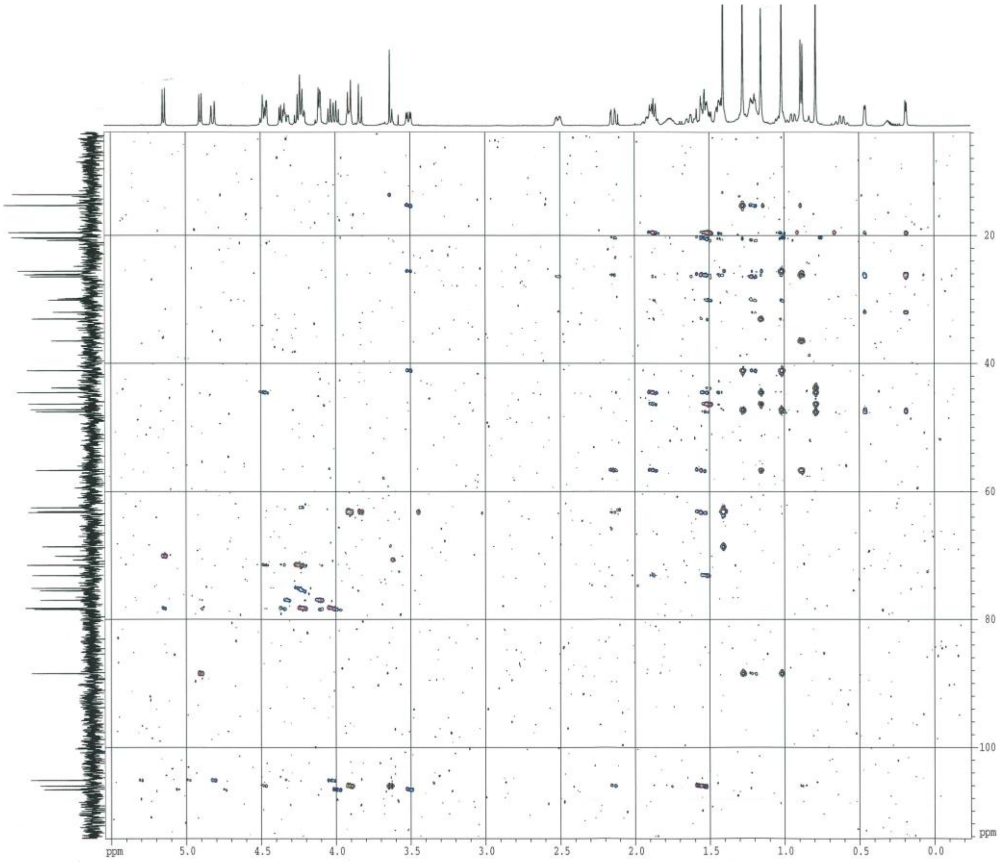

^{1}H-NMR spectrum of **13** in C_5D_5N

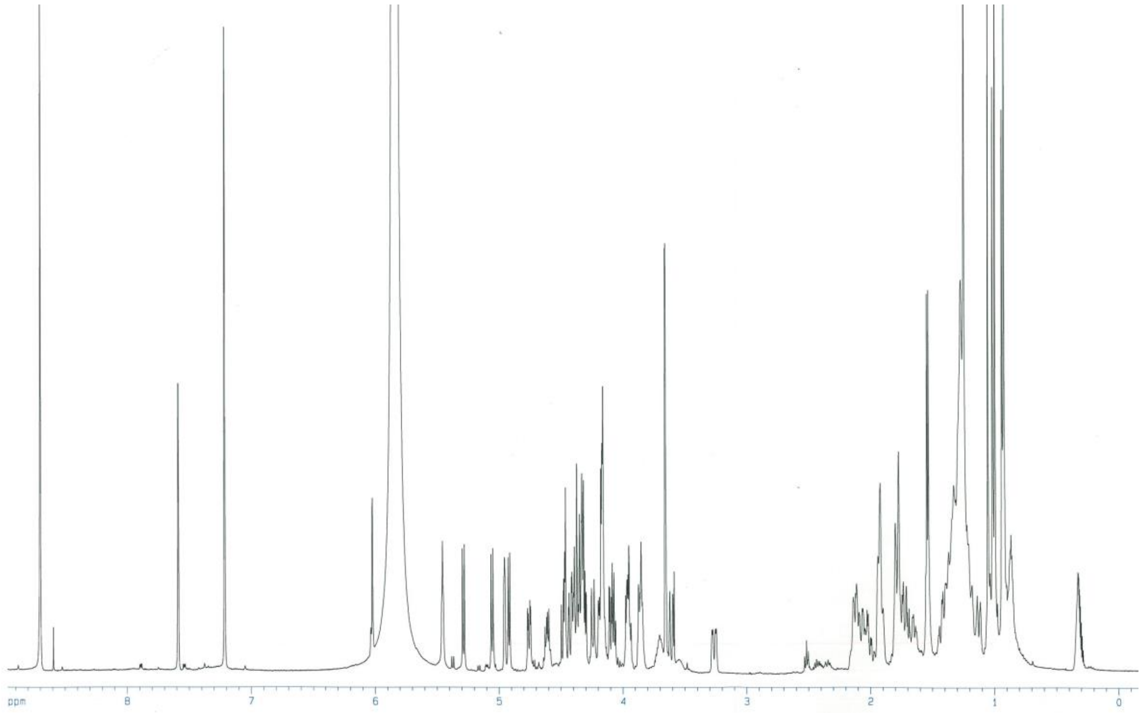

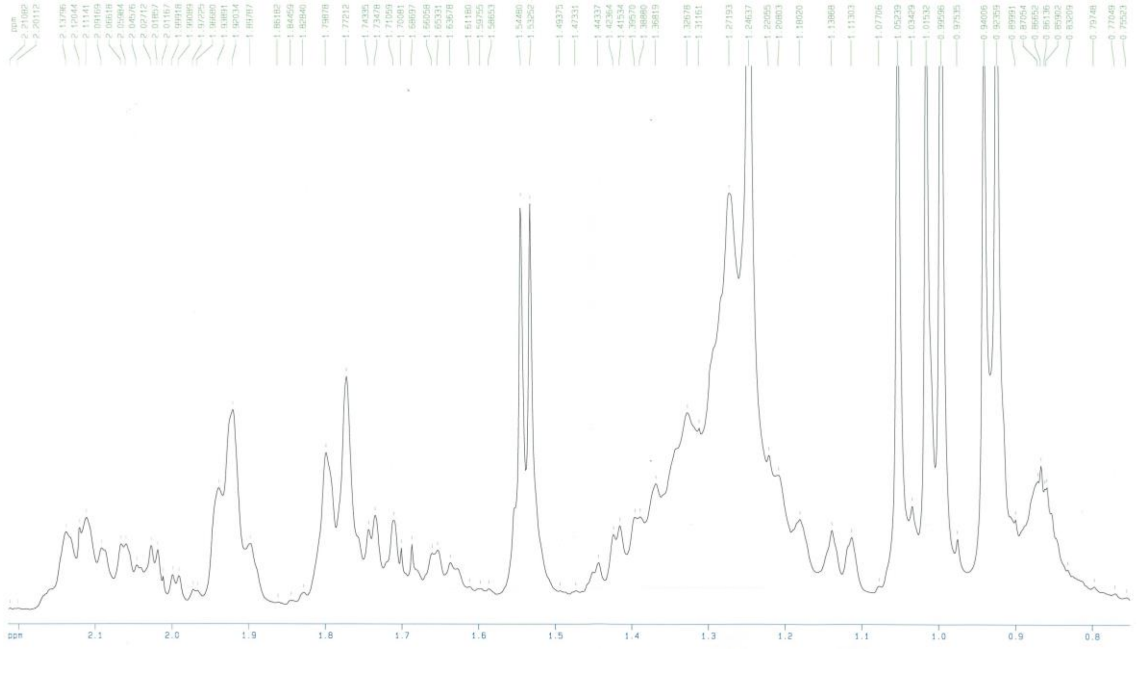

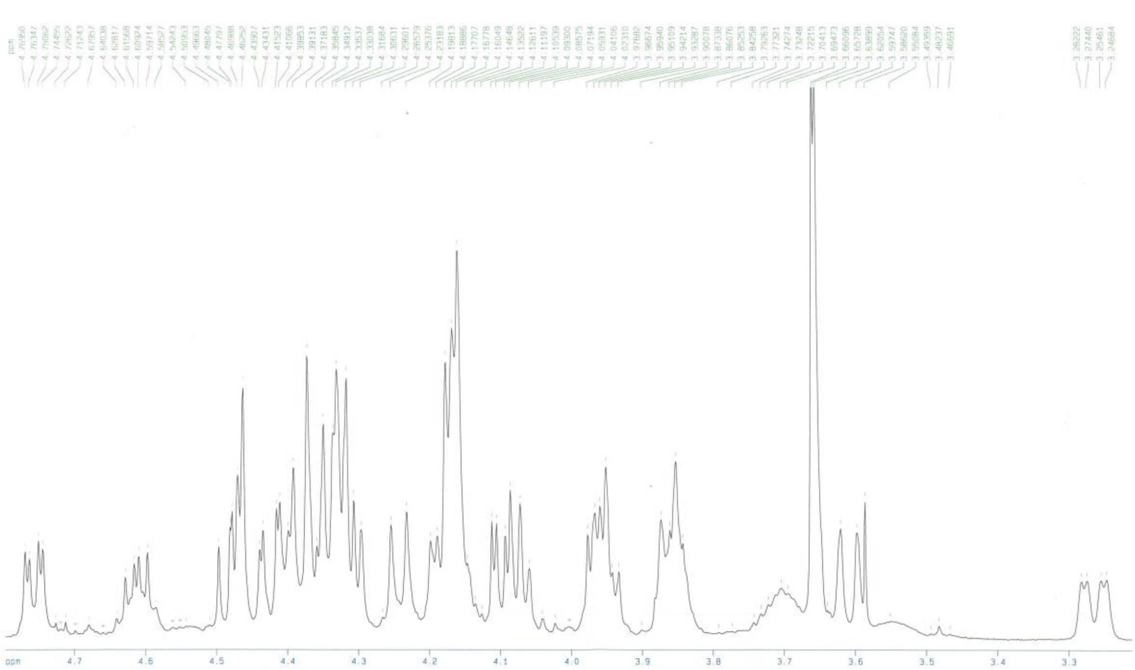
ppm
4.7
4.6
4.5
4.4
4.3
4.2
4.1
4.0
3.9
3.8
3.7
3.6
3.5
3.4
3.3

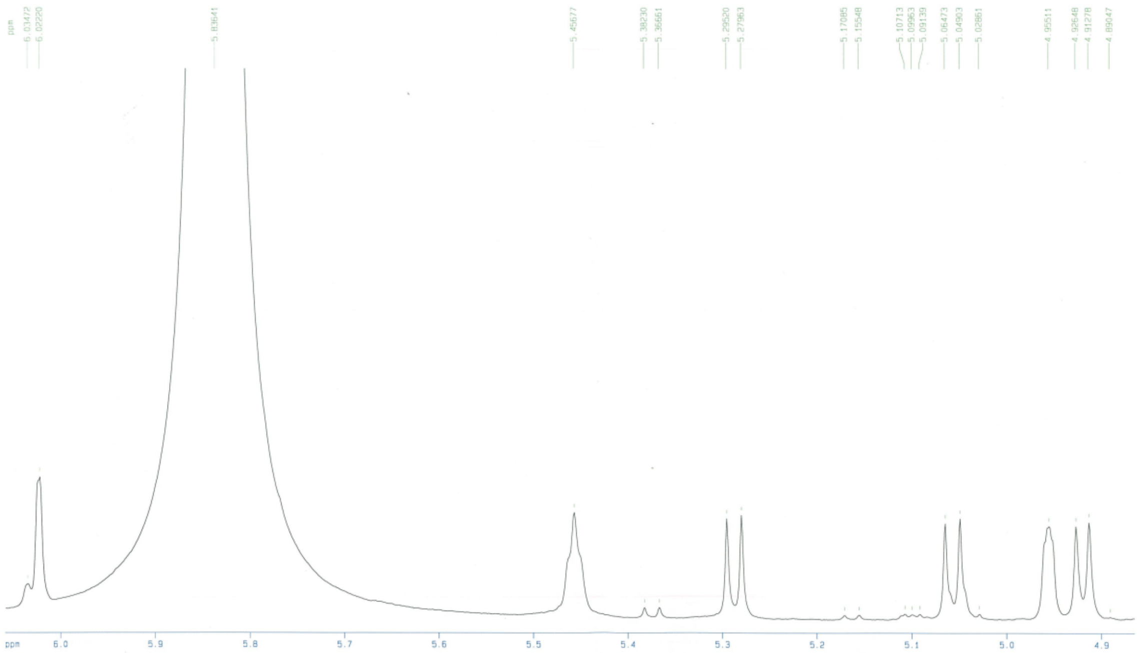
ppm
6.03472
6.02220
5.83641
5.45677
5.38230
5.36661
5.29520
5.27963
5.17085
5.15548
5.10713
5.09963
5.09139
5.06473
5.04903
5.02861
4.95511
4.92648
4.91278
4.89047
6.0
5.9
5.8
5.7
5.6
5.5
5.4
5.3
5.2
5.1
5.0
4.9

^{13}C-NMR spectrum of **13** in C_5D_5N

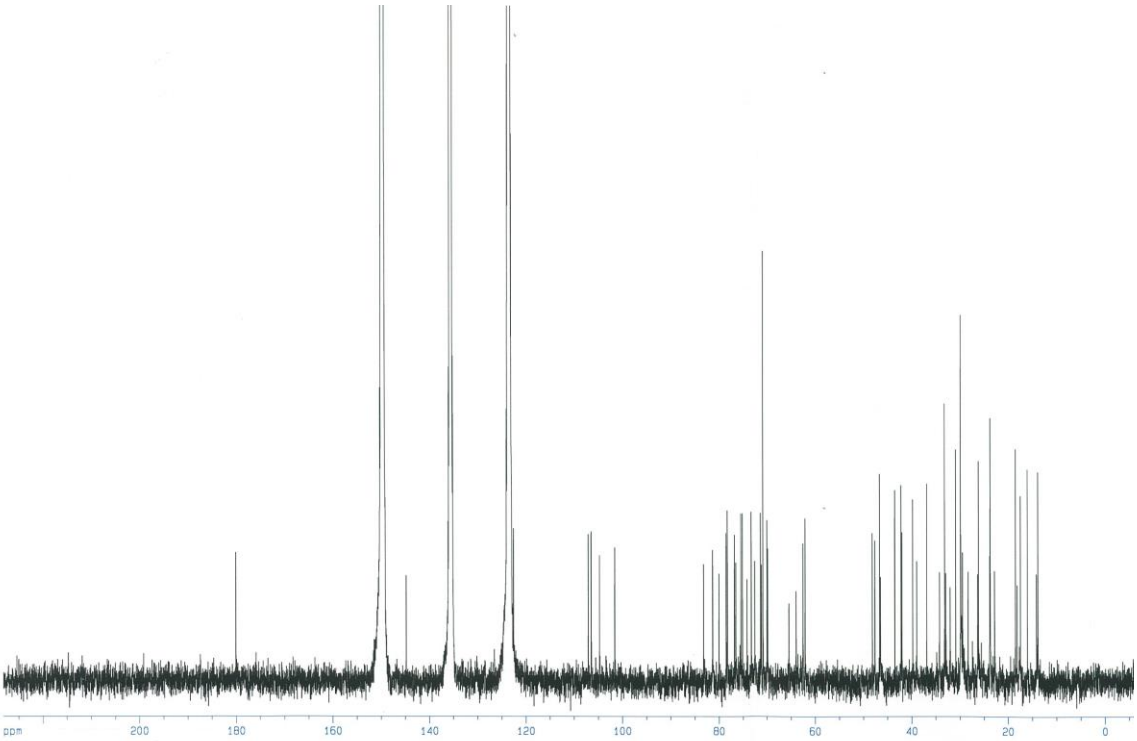

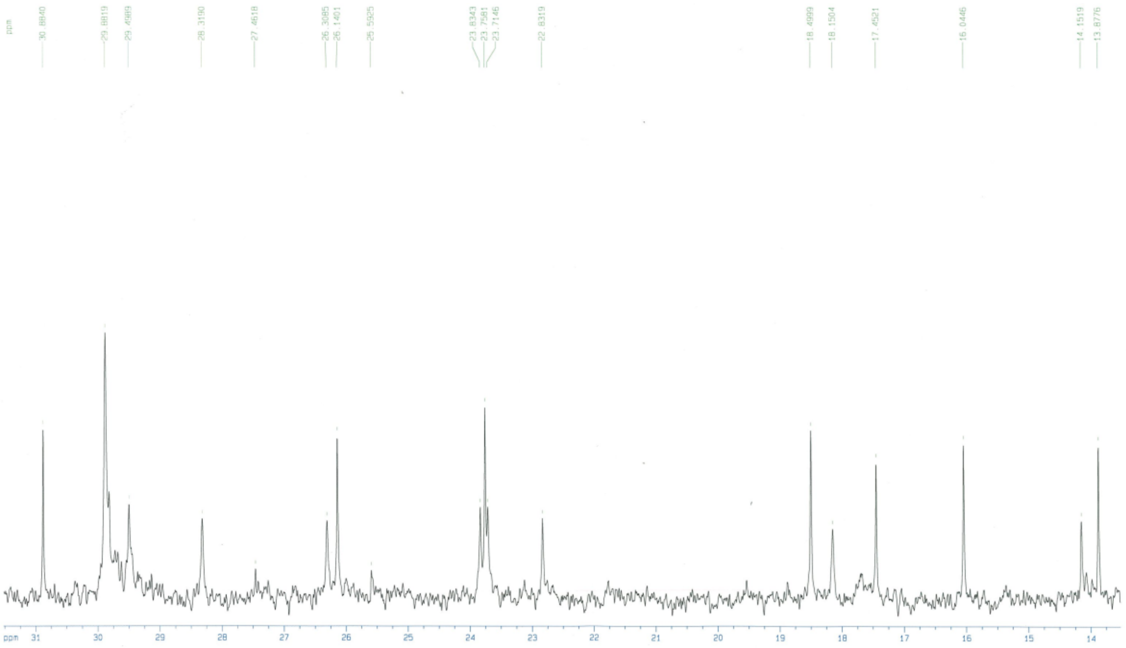

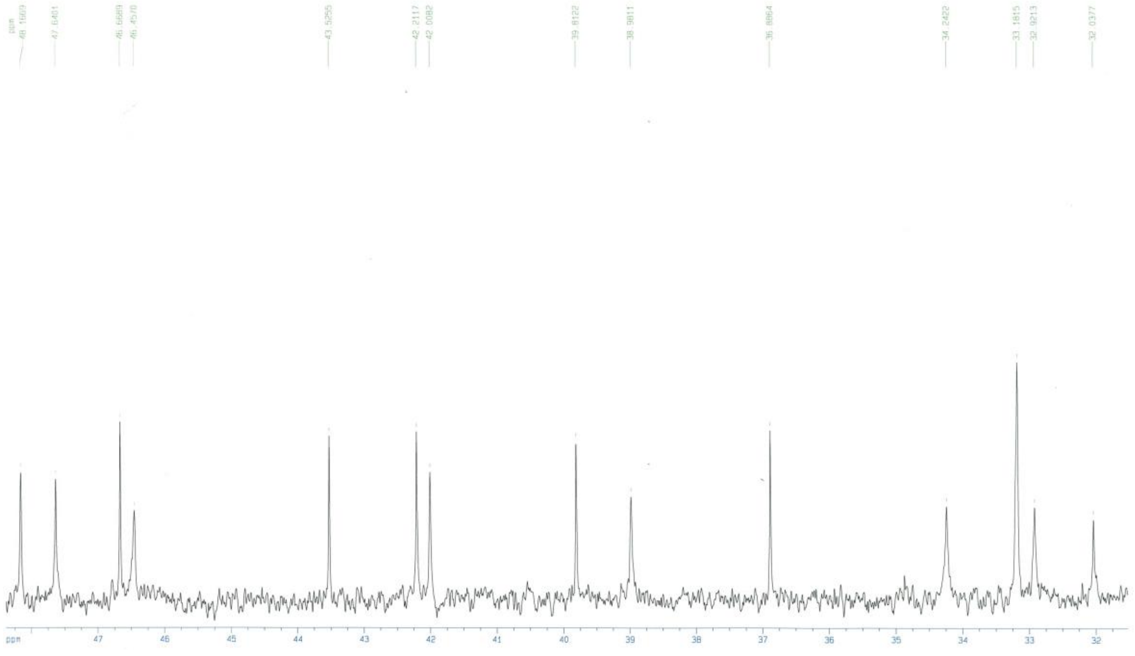

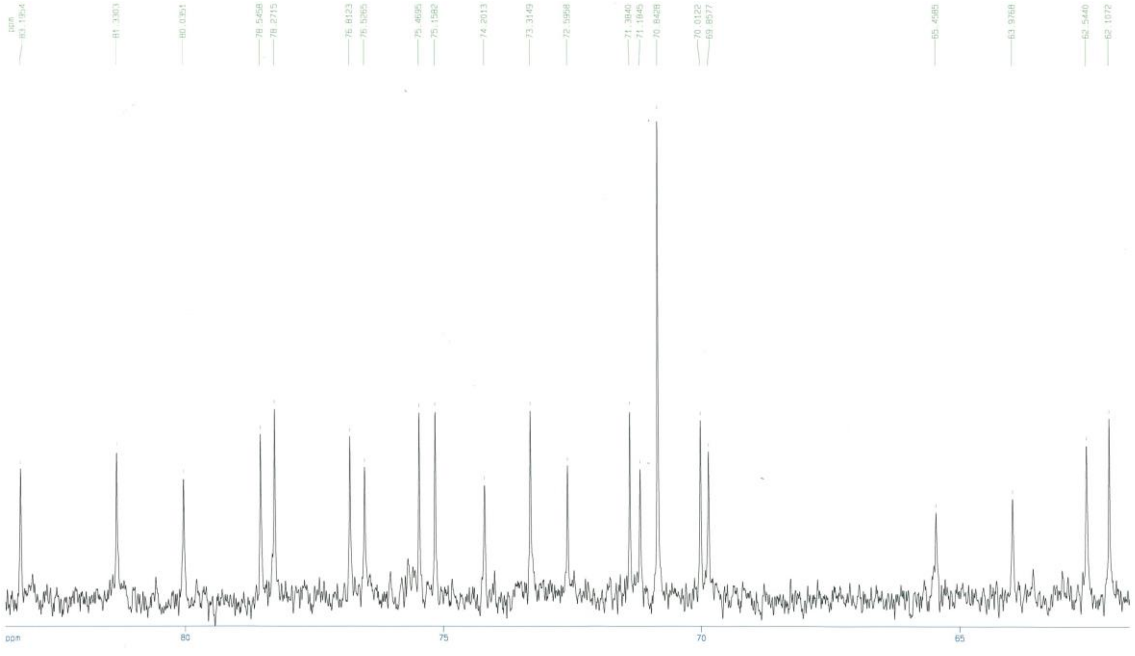

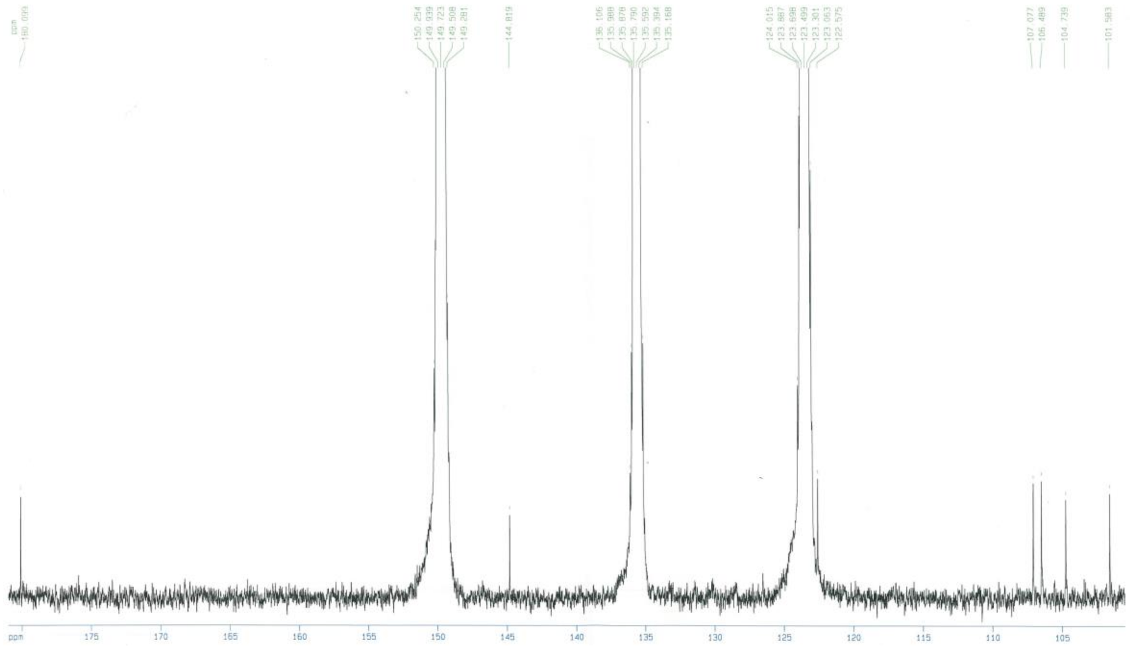

COSY spectrum of **13** in C_5D_5N

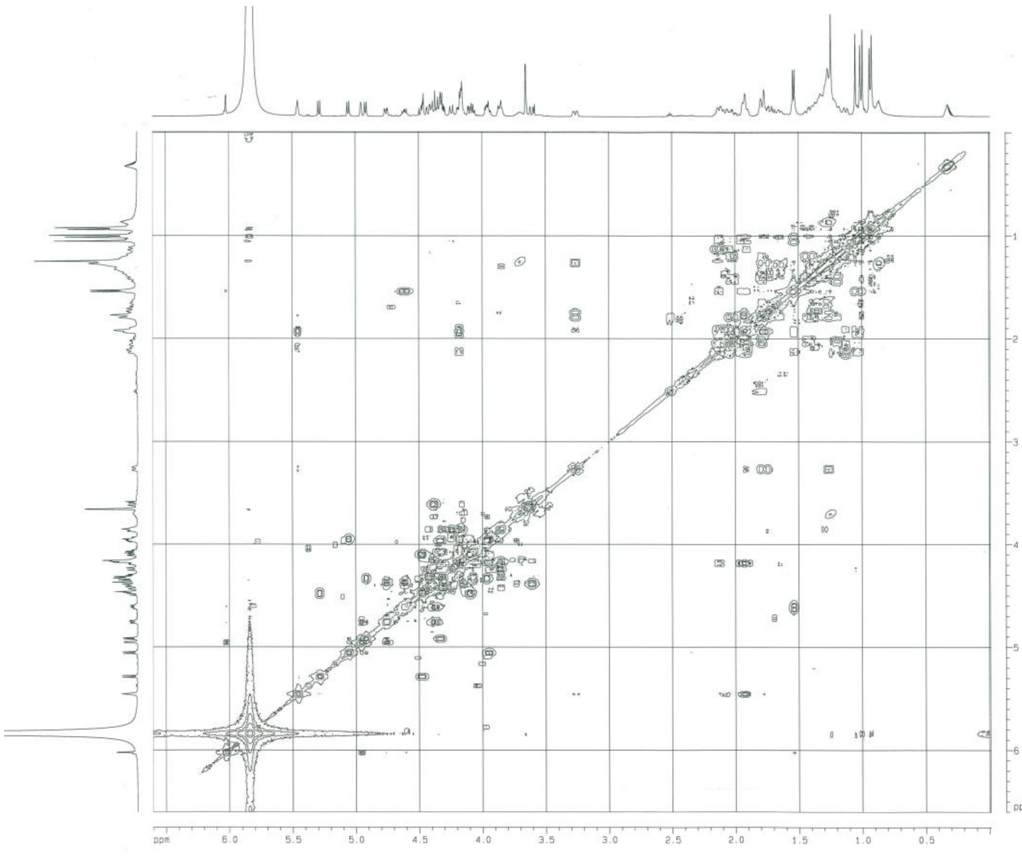

HMQC spectrum of **13** in C_5D_5N

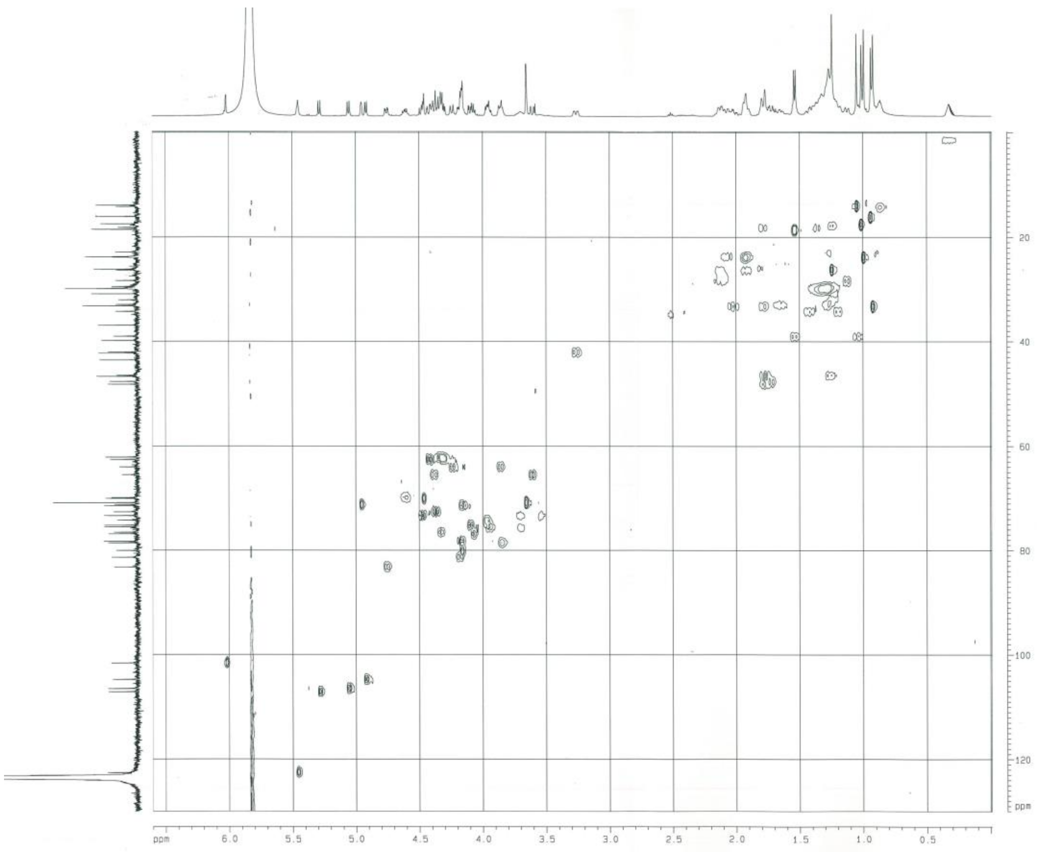

HMBC spectrum of **13** in C_5D_5N

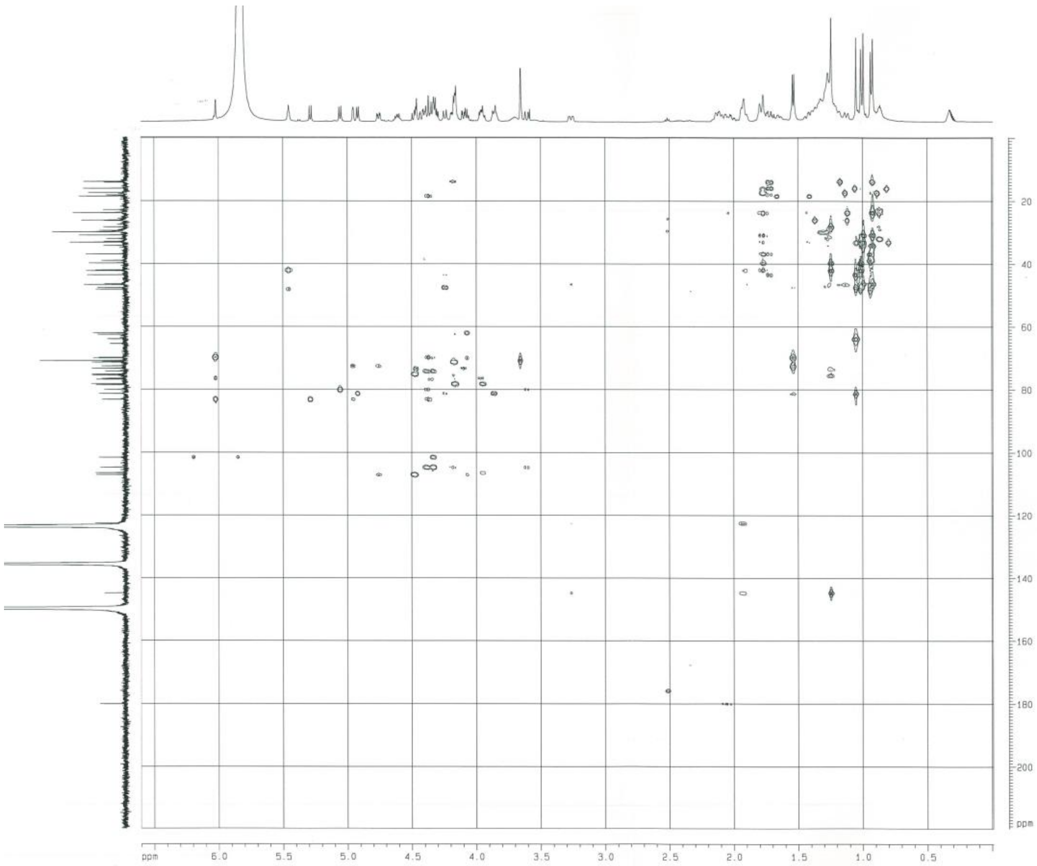

NOESY spectrum of **13** in C_5D_5N

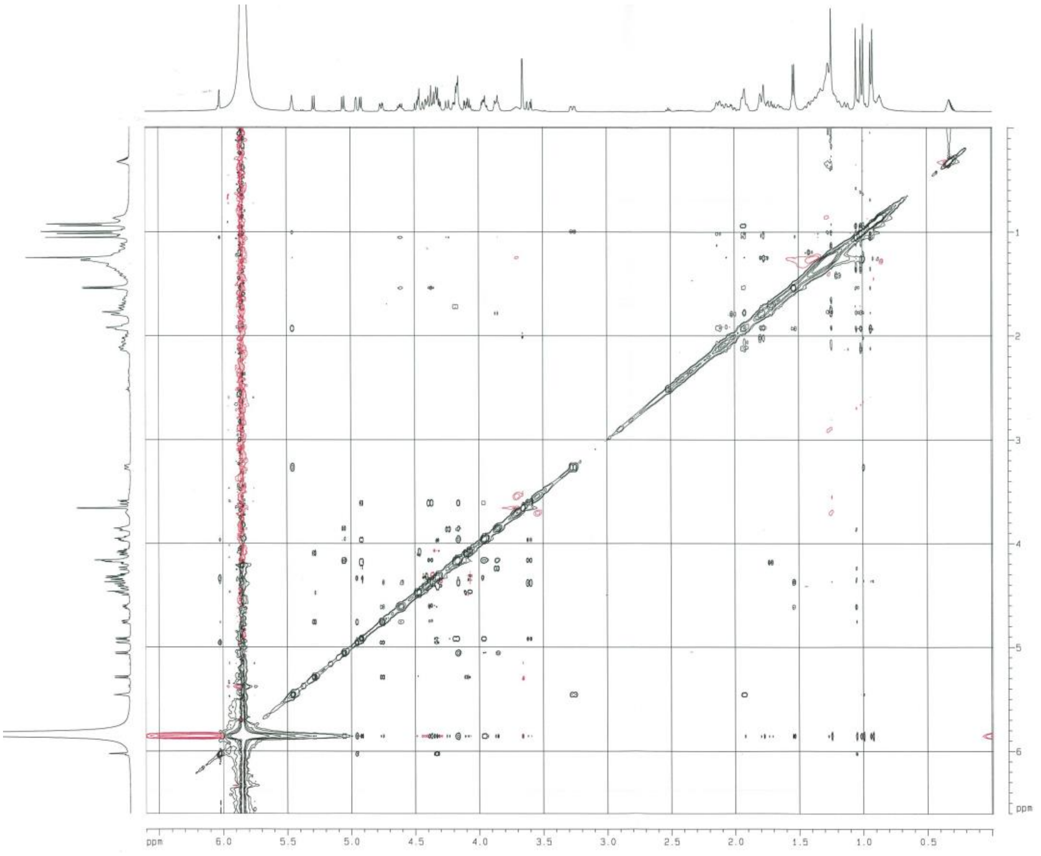

Supplement: Supplementary file 1 [file molecules-24-00069-s001.zip › E.cilicica NMR spectral data.pdf]
